# *SNCA* correlates with immune infiltration and serves as a prognostic biomarker in lung adenocarcinoma

**DOI:** 10.1186/s12885-022-09289-7

**Published:** 2022-04-14

**Authors:** Xiuao Zhang, Zhengcun Wu, Kaili Ma

**Affiliations:** 1 Institute of Medical Biology, Chinese Academy of Medical Sciences and Peking Union Medical College, Kunming, 650118 China; 2 Neuroscience Center, Chinese Academy of Medical Sciences and Peking Union Medical College, Beijing, 100005 China; 3 Yunnan Key Laboratory of Vaccine Research Development on Severe Infectious Diseases, Kunming, 650118 China

**Keywords:** *SNCA*, Lung adenocarcinoma, Survival prognosis, Immune infiltration, Immune checkpoints, Methylation

## Abstract

**Background:**

The *SNCA* gene is a critical gene in Parkinson’s disease (PD) pathology. Accumulating evidence indicates that *SNCA* is involved in tumorigenesis; however, the role of *SNCA* in lung adenocarcinoma (LUAD) remains unclear. This study aimed to explore the potential value of *SNCA* as a prognostic and diagnostic molecular marker in LUAD.

**Methods:**

In this study, we explored the expression pattern, prognostic value, and promoter methylation status of *SNCA* in LUAD based on Oncomine, UALCAN, and Kaplan–Meier Plotter. Then, using TIMER, we investigated the correlation between *SNCA* expression and immune infiltration. And cBioPortal were used to analysis the correlation between *SNCA* expression and immune checkpoint. The transcriptome data of A549 cells overexpressing *SNCA* were used to further study the potential immune role of *SNCA* in LUAD. The effect of *SNCA* on proliferation of A549 cells were evaluated by CCK-8, EdU and colony formation. Finally, LUAD cell lines treated with 5-aza-dC were used to explore the correlation between increased promoter methylation and downregulated mRNA expression of *SNCA*.

**Results:**

In general, the expression level of *SNCA* in LUAD tissue was lower than that in normal tissue, and high expression of *SNCA* was related to better prognosis.

There were significant positive correlations between *SNCA* expression and immune infiltrations, including CD8+ T cells, macrophages, neutrophils, dendritic cells, B cells, and CD4+ T cells, and immune checkpoints, suggesting that immune infiltration was one of the reasons for the influence of *SNCA* on prognosis in LUAD. The transcriptome data of A549 cells overexpressing *SNCA* were further used to screen the relevant immune-related genes regulated by *SNCA*. Enrichment analysis confirmed that *SNCA* participates in the PI3K-AKT signaling pathway and other key tumor signaling pathways and regulates the expression of *MAPK3*, *SRC*, *PLCG*1, and *SHC1*. Cellular proliferation assay showed that *SNCA* could inhabit the growth of A549 cells via inhibiting activity of PI3K/AKT/ mTOR pathway. Finally, analysis of the methylation level of *SNCA* promoter showed that the promoter methylation negatively correlated with mRNA level. The expression of *SNCA* in LUAD cell lines was significantly upregulated by treatment with 5-aza-dC.

**Conclusion:**

High methylation of *SNCA* promoter in LUAD is one of the reasons for the downregulation of *SNCA* mRNA level. Given that *SNCA* could inhibit the proliferation of A549 cells and correlates with immune infiltrates, it may serve as a prognostic biomarker in LUAD.

**Supplementary Information:**

The online version contains supplementary material available at 10.1186/s12885-022-09289-7.

## Introduction

Lung cancer has the highest morbidity and mortality among all cancer types worldwide [1]. According to statistics for 2020, there were 2.2 million new cases of lung cancer and about 1.8 million lung cancer-related deaths worldwide [2]. About 85% of lung cancer patients are non-small-cell lung cancer (NSCLC). Lung adenocarcinoma (LUAD) is the main histological type of NSCLC, accounting for about 50% of all NSCLC cases, and its overall survival rate is low [3]. Although there has been much progress in early detection and standard treatment, LUAD is usually diagnosed at a late stage with poor prognosis. Therefore, new therapeutic methods and effective molecular targets for LUAD are urgently needed.

Interestingly, a large number of epidemiological studies have shown a significant reduction in the risk of cancer in patients with Parkinson’s disease (PD) [4]. A meta-analysis of 15 studies, involving 348,780 patients with PD, showed a significant negative correlation between PD and lung cancer risk (RR: 0.53, 95%CI: 0.41–0.70, *P* < 0.001) [5]. Both PD and lung cancer are multifactorial disorders involving genes and the environment. It is speculated that there is a reverse molecular link between PD and lung cancer, such that PD is the ‘harmful culprit’ and lung cancer is the “intentional participant”.

*SNCA* is the first gene reported to be involved in the pathological process of spontaneous PD [6], and the accumulation of alpha-synuclein (α-syn) encoded by *SNCA* is one of the inducers of PD. α-syn is mainly located in presynaptic nerve terminals and plays important regulatory roles in synapse maintenance, mitochondrial homeostasis, proteasome function, dopamine metabolism, and molecular chaperone activity [7]. A number of studies have shown that *SNCA* plays a different role in different cancers. In the SKCM cell line SK-Mel-28, the lack of *SNCA* is associated with dysregulated iron metabolism, leading to ferritin-ferric iron accumulation and cell apoptosis [8]. According to Pan et al., *SNCA* may be involved in the regulation of melanin synthesis [9]. In addition, it has been expected that *SNCA* methylation in human feces may become a biological diagnostic marker for colorectal cancer [10]. Overexpression of *SNCA* in medulloblastoma can inhibit tumor invasion and induce cell apoptosis [11]. Through microarray analysis of drug resistance-related microRNAs, Zou et al. proved that the downregulation of *SNCA* was significantly related to the multidrug resistance of ovarian cancer [12]. In a study about lymphoma, it has been reported that hypermethylation of the promoter of *SNCA* can be used as an early diagnostic marker [13]. Early research has predicted that *SNCA* is a biomarker of LUAD and a potential therapeutic target [14, 15] . However, the biological functions of *SNCA* in LUAD have not been further explored.

In this study, we first explored the expression of *SNCA* in LUAD and its impact on prognosis through Oncomine and UALCAN databases. In addition, due to the key role of the immune microenvironment in tumor development, we evaluated the potential correlation between *SNCA* and immune infiltration through TIMER database and cBioPortal. Combined with the transcriptome data of the A549 cells overexpressing *SNCA* constructed by our laboratory, immune-related differential genes regulated by *SNCA* were screened, and the related pathways and key genes that may be involved in LUAD regulation were analyzed. Finally, UALCAN, Wanderer, and DiseaseMeth databases were used to explore the methylation changes of *SNCA* promotel. Hence, our study revealed the clinical significance of *SNCA* in LUAD. Moreover, it may provide a new perspective for the prognostic evaluation of LUAD, suggesting that *SNCA* combined with immune checkpoint may be a new diagnosis or treatment strategy for LUAD.

## Materials and methods

### Oncomine database analysis

The mRNA expression of *SNCA* in different cancer types and meta-analysis of five LUAD studies were analyzed by Oncomine database (https://www.oncomine.org/resource/main.html) [16]. The threshold was set at a *P*-value of 0.01 and a fold change of 1.5.

### TIMER database analysis

The TIMER database (https://cistrome.shinyapps.io/timer/) was used to analyze the differences in gene expression and the level of immune infiltration in different tumors and adjacent tissues [17]. We used the TIMER database to confirm the expression levels of *SNCA* in various tumors and correlation between *SNCA* expression and immune infiltration (B cells, CD4 + T cells, CD8 + T cells, neutrophils, macrophages, and dendritic cells) in LUAD. Finally, the correlation between the markers in dendritic cells, CD8 + T cells, macrophages, NK cells, Treg cells, B-cells, monocytes, and neutrophils and *SNCA* in LUAD was verified. The *P* value was corrected for tumor purity. The Spearman method was used to determine the correlation coefficient.

### Survival analysis in TCGA and Kaplan–Meier plotter databases

The clinical information of 594 LUAD samples from TCGA database (https://tcga-data.nci.nih.gov/tcga/) was analyzed for overall survival and prognosis, and the patient’s genders, ages, smoking history, and TNM tumor staging were assessed using hierarchical analysis. The ‘survival’ package in R software was used to perform the analysis. The Kaplan–Meier plotter (http://kmplot.com/) is an online database of published microarray data sets [18]. It was used to predict the relationship between *SNCA* expression and prognosis in LUAD-related immune subgroups. Survival was assessed by hazard ratio (HR) and its 95% confidence interval and corresponding *P* value. *P* < 0.05 was considered statistically significant.

### cBioPortal database analysis

cBioPortal database (www.cbioportal.org) is a comprehensive network resource that can visualize and analyze multi-dimensional cancer genome data [19]. The overall situation of *SNCA* and immune checkpoint changes in LUAD were analyzed via cBioPortal database.

### Cell culture and transfection

A549 and H1299 cells purchased from ATCC (LGC Standards GmbH, Wesel, Germany) were cultured in DMEM (Gibco, USA) containing 10% fetal bovine serum (Sigma-Aldrich, USA) at 37 °C in a humidified atmosphere containing 5% CO_2_. The cells were treated with 5 μM 5-aza-dC (Sigma-Aldrich, USA) for 72 h. Then, they were collected for RNA extraction. To obtain the cells overexpressing *SNCA*, a plasmid overexpressing *SNCA* constructed by our laboratory was transfected into A549 cells using EndoFectin-Lenti (GeneCopoeia, USA) in accordance with the manufacturer’s protocol. The cells were harvested for RNA extraction after 2 days of selection with 5 μg/ml puromycin (Solarbio, China).

### RNA sequencing and screening for differentially expressed genes (DEGs)

The total RNA from the A549 cells overexpressing *SNCA* and that from control cells (empty vectors) were isolated using TRIzol reagent (Sigma, USA). The concentration and integrity of the extracted total RNA were estimated by NanoPhotometer (IMPLEN, Germany). Next, the processed RNAs were subjected to deep sequencing with an Illumina HiSeq 3000 (Illumina, San Diego, CA). Mapping of paired-end reads was carried out as follows. Before read mapping, clean reads were obtained from the raw reads by removing the adaptor sequences, reads with > 5% ambiguous bases (noted as N), and low-quality reads containing more than 20% of bases with qualities of < 20. Differential gene expression analysis of RNA sequencing experiments was performed with TopHat and Cufflinks. Nat Protoc20. HTseq was used to count genes, and the RPKM method was used to determine the gene expression. We applied the DESeq 22 algorithm to identify the DEGs, after the significance, *P*-value, and FDR analyses, under the following criteria: (i) log2 (FC) > 1.5 or log2 (FC) < − 1.5 and (ii) FDR < 0.01. The immune-related DEGs regulated by *SNCA* were obtained through overlap analysis between the DEGs screened above and the genes from Immport Resource (https://www.immport.org/resources).

### GO and KEGG pathway enrichment analyses of DEGs

The DAVID Bioinformatics Resources 6.8 (https://david.ncifcrf.gov/) is a commonly used database for distinguish and enrich the biological attributes, such as biological processes, cellular components, molecular functions and pathways of important DEGs [20]. The Kyoto Encyclopedia of Genes and Genomes (KEGG) pathway (www.kegg.jp/kegg/kegg1.html) [21] and Gene Ontology (GO) enrichment analyses of the identified DEGs performed by DAVID were used to identify the significant pathways. *P* < 0.05 was set as the cutoff criterion for significant enrichment.

### Cellular proliferation

The transfected cells were seeded into 96-well plates at a density of 2 × 10^4^ cells per well. Cell proliferation was assessed using a CCK-8 kit (DOJINDO, Japan) and EdU assay (Cell-Light™ EdU Apollo®594, UE, China) according to the protocol of the manufacturer. For the CCK-8 assay, absorbance was read at 480 nm using a microplate reader (Tecan, Switzerland). EdU-positive cells were captured using a fluorescence microscope (Nikon, Japan), and percentages of EdU-positive cells were assessed using Image J. Clonony formation assay was performed in standard 6-well plates using a cell density of 1000 cells per well. After 7–10 days of culture, cells were fixed using 4% paraformaldehyde for 15 min and stained with 0.1% crystal violet (Biotime, USA) for 10 min. The numbers of the clones were analyzed to determine cellular proliferation capacity.

### Protein-protein interaction (PPI) network construction and analysis of modules

Search Tool for the Retrieval of Interacting Genes/Proteins (STRING) (https://string-db.org/) is a search tool that can analyze the interaction relationships between proteins. The use of STRING to analyze the PPI network of DEGs can help us to understand the relationships between different genes. Cytoscape software was used to screen for hub genes according to degrees. The modules in the PPI network were analyzed using the Cytohubba plug-in in Cytoscape software.

### RNA isolation and quantitative real-time PCR (RT-qPCR) analysis

Total RNA was extracted from the cells using TRIzol reagent (Sigma-Aldrich, USA) in accordance with the manufacturer’s instructions. Concentration and quality of the RNA were determined by a NanoPhotometer (IMPLEN, Germany). RNA was reverse-transcribed into cDNA using Reverse Transcription Kit (Promega, USA). The qRT-PCR was performed using Eastep qPCR Master Mix (Promega, USA) and a CFX96 Real-Time PCR Detection System (Bio-Rad, USA) in accordance with the manufacturer’s instructions. The primers’ sequences are listed in Supplementary Table S1. The relative expression of genes was calculated based on the 2^-ΔΔCt^ method with *GAPDH* as an internal reference.

### Western blotting

Western blotting was performed as described previously [22]. The cells were disrupted with ice-cold RIPA buffer with a final concentration of 2 mM PMSF and 1× protease inhibitor cocktail (Merck, USA). The protein concentration was determined using a BCA Protein Assay Kit (Cwbio, Beijing, China). Total protein (20 μg) was separated by 10% TGX Stain-Free gels (Bio-Rad, USA) and transferred to a nitrocellulose membrane (Millipore, USA). 5% skim milk was used to block the membranes for 1 h at room temperature. The membrane was cropped according to predicted position of target protein. Subsequently, blots was incubated with primary antibody at 4 °C overnight and then incubated with specific infrared labeled secondary antibodies ((Licor, USA)) after washing with PBST three times (all antibodies are listed in Supplementary Table S2). The bands were visualized using the LI-COR Odyssey system (Licor, USA). This system produces a signal number for each band identified on a western blot generated by the near-infrared fluorescent detection of secondary antibodies used. Infrared detection offers solutions to the problems associated with chemiluminescence and fluorescent detection methods including static detection, low autofluorescence, equivalent or higher sensitivity, higher signal-to-noise ratio, detection range, multiplex labeling, and greater efficiency [23]. The densitometric analyses of the blots were performed using Image J software. GAPDH was used as a loading control. The *t* test was used to estimate the significance of difference in gene expression levels between groups. * *p* < 0.05; ** *p* < 0.01.

### Immunohistochemical (IHC) staining

IHC was performed as described previously [22]. The slides were dried at 60 °C, dewaxed with methanol, and rehydrated with alcohol. The slides were incubated with sodium citrate buffer in a microwave oven for 15 min and then immersed in 3% hydrogen peroxide to block endogenous peroxidase. They were then incubated with the SNCA-antibody (Abcam, USA) at 4 °C overnight. Next, the slides were treated with secondary antibodies (Abcam, USA) for 1 h. Finally, they were stained with diaminobenzidine (DAB) until brown granules appeared. The slides were observed using a laser multicolor fluorescence scanning imager (GE, USA). The protein expression was analyzed using Image-Pro Plus 6.0 Software.

### Methylation analysis in UALCAN, wanderer, and DiseaseMeth databases

UALCAN database (http://ualcan.path.uab.edu/index.html) is an easy-to-use interactive web portal for in-depth analysis of TCGA gene expression data [24]. It can be used to evaluate the methylation level of genes in different tumors; it can also analyze the expression levels of mRNAs and proteins and perform stratified analysis according to the patient’s individual cancer stage and lymph node metastasis status. Wanderer (http://maplab.imppc.org/wanderer/) is generally used to explore DNA methylation and gene expression data in human cancer [25]. We used it to estimate the correlation between *SNCA* methylation level and mRNA expression level in LUAD. DiseaseMeth (http://bioinfo.hrbmu.edu.cn/diseasemeth/) is an interactive database that reflects the most complete collection and annotations of abnormal DNA methylation in human diseases, especially in various cancers [26]. We used it to evaluate the methylation level of four genomic fragments of *SNCA* promoter.

### Statistical analysis

The Student’s *t* test was used to examine differences between groups. *P*-values less than 0.05 were considered statistically significant. The survival analysis was obtained from a log-rank test, and the correlations of *SNCA* with immune infiltration and markers of immune cells were evaluated using Spearman’s correlation.

## Results

### Decreased expression of *SNCA* in LUAD

**Fig. 1:**
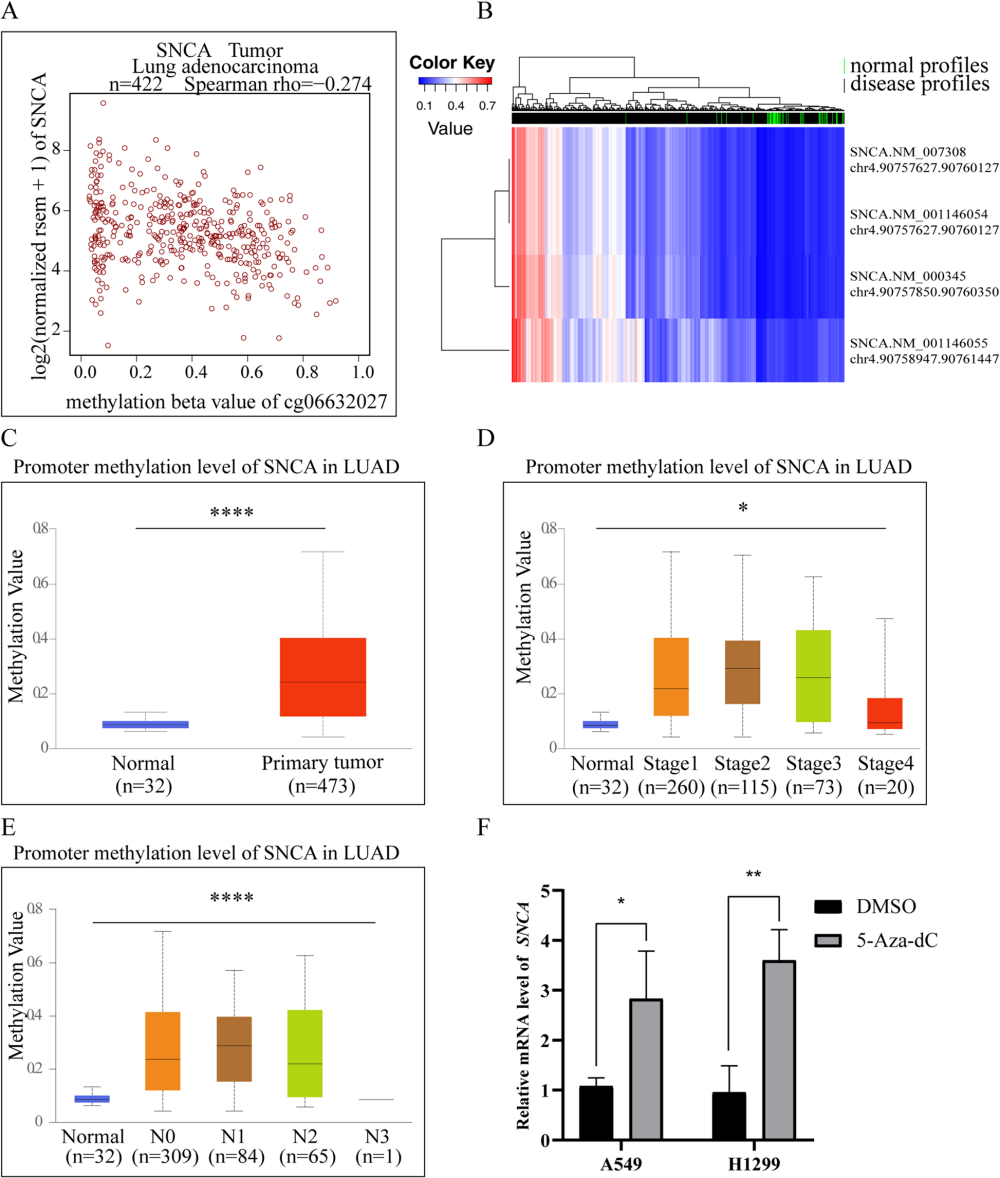
The expression of *SNCA* in different tumors was analyzed in Oncomine and TIMER databases. **A** Oncomine database was used to analyze the expression of *SNCA* gene in Pan-cancer species. Note: red represents upregulation of the target gene, while blue represents downregulation of the target gene. Threshold parameter: *p*-value is 0.01, fold change is 1.5. **b** The analysis of the expression level of *SNCA* in different tumors in TCGA through TIMER database. **C** Meta-analysis of *SNCA* expression was performed using five LUAD sequencing data sets through Oncomine database. **D** UALCAN database analysis of the mRNA expression level of *SNCA* in LUAD (**** *P* < 0.0001). **E** According to the analysis of UALCAN database, the protein expression level of *SNCA* in LUAD (**** *P* < 0.0001)

In order to investigate the changes of *SNCA* expression in tumor tissues, we evaluated the transcriptional level of *SNCA* expression in different tumors from Oncomine. It showed that *SNCA* was generally downregulated in bladder cancer, brain and CNS cancer, breast cancer, kidney cancer, lung cancer, ovarian cancer, and other tumors (Fig. 1A). Based on TIMER database, the expression of *SNCA* was significantly decreased in bladder urothelial carcinoma (BLCA), colon adenocarcinoma (COAD), glioblastoma multiforme (GBM), kidney renal clear cell carcinoma (KIRC), kidney renal papillary cell carcinoma (KIRP), thyroid carcinoma (THCA), and uterine corpus endometrial carcinoma (UCEC) (Fig. 1B). What attracted our attention was that the expression level of *SNCA* was also significantly reduced in LUAD (Fig. 1B). Further meta-analysis of five data sets of LUAD from Oncomine showed that the mRNA expression level of *SNCA* in LUAD was significantly lower than that in normal tissues (*P* < 0.001; Fig. 1C). Using UALCAN database to analyze 515 LUAD tissues and 59 normal samples from TCGA, we found that the mRNA level of *SNCA* was lower in LUAD tissues than in normal tissues (Fig. 1D). Analysis of CPTACS database in UALCAN showed that the protein expression level of *SNCA* in LUAD was also reduced (Fig. 1E).

### *SNCA* expression is associated with survival

**Fig. 2:**
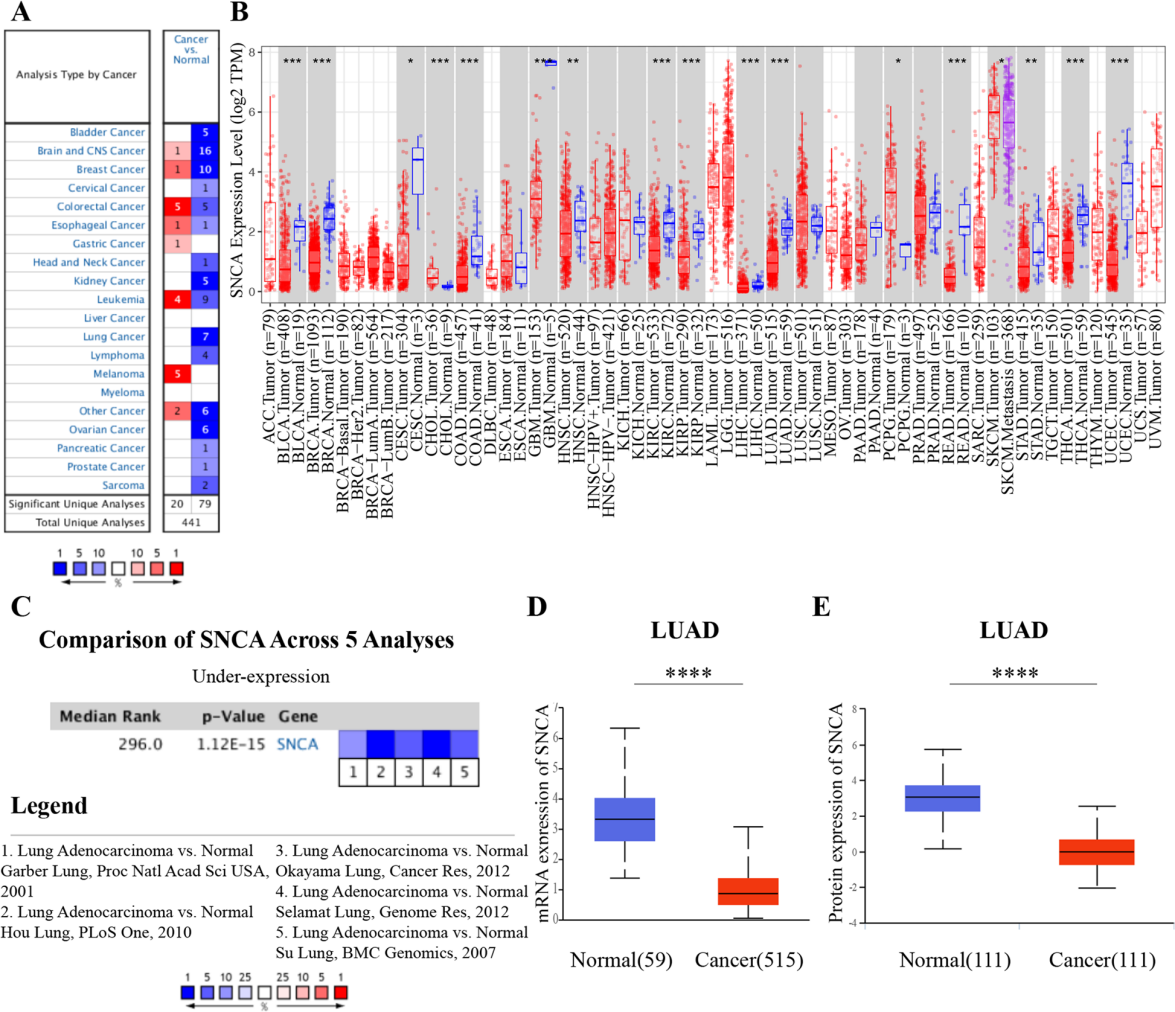
Kaplan–Meier curve analysis of the influence of *SNCA* expression level in LUAD on the overall survival rate. **A** Kaplan–Meier curves of *SNCA* in all tumor patients. **B**–**F** Subgroup analysis based on age (> 65), N2, M0, stage III, and R0

Next, in order to understand the correlation between *SNCA* expression and prognosis of patients, we used Kaplan–Meier curve to analyze the effect of *SNCA* expression level on survival of patients with LUAD from the TCGA. According to the median was based on *SNCA* expression, the patients were divided into a high-SNCA expression group and a low-SNCA expression group. As shown in Fig. 2A, patients with high expression of *SNCA* showed better overall survival rate than those with low expression of *SNCA* (*P* = 0.027). The same result was obtained in GSE31210 (*P* = 0.028)(Supplementary Fig. S1). Further subgroup analysis of multiple clinical features showed that the low expression of *SNCA* was significantly associated with poor prognosis in LUAD patients based on age (> 65 years) (*P* = 0.019), N2 (*P* = 0.011), M0 (*P* = 0.017), stage III (*P* = 0.01), and R0 (*P* = 0.021). The results showed that the high expression of *SNCA* in LUAD could improve the overall survival (OS) rate of LUAD, and the prognosis of patients with high *SNCA* expression was better than that of patients with low *SNCA* expression. Hence, *SNCA* is one of the good prognostic factors in LUAD.

### The transcription level of *SNCA* correlates with tumor immune infiltration in LUAD

**Fig. 3:**
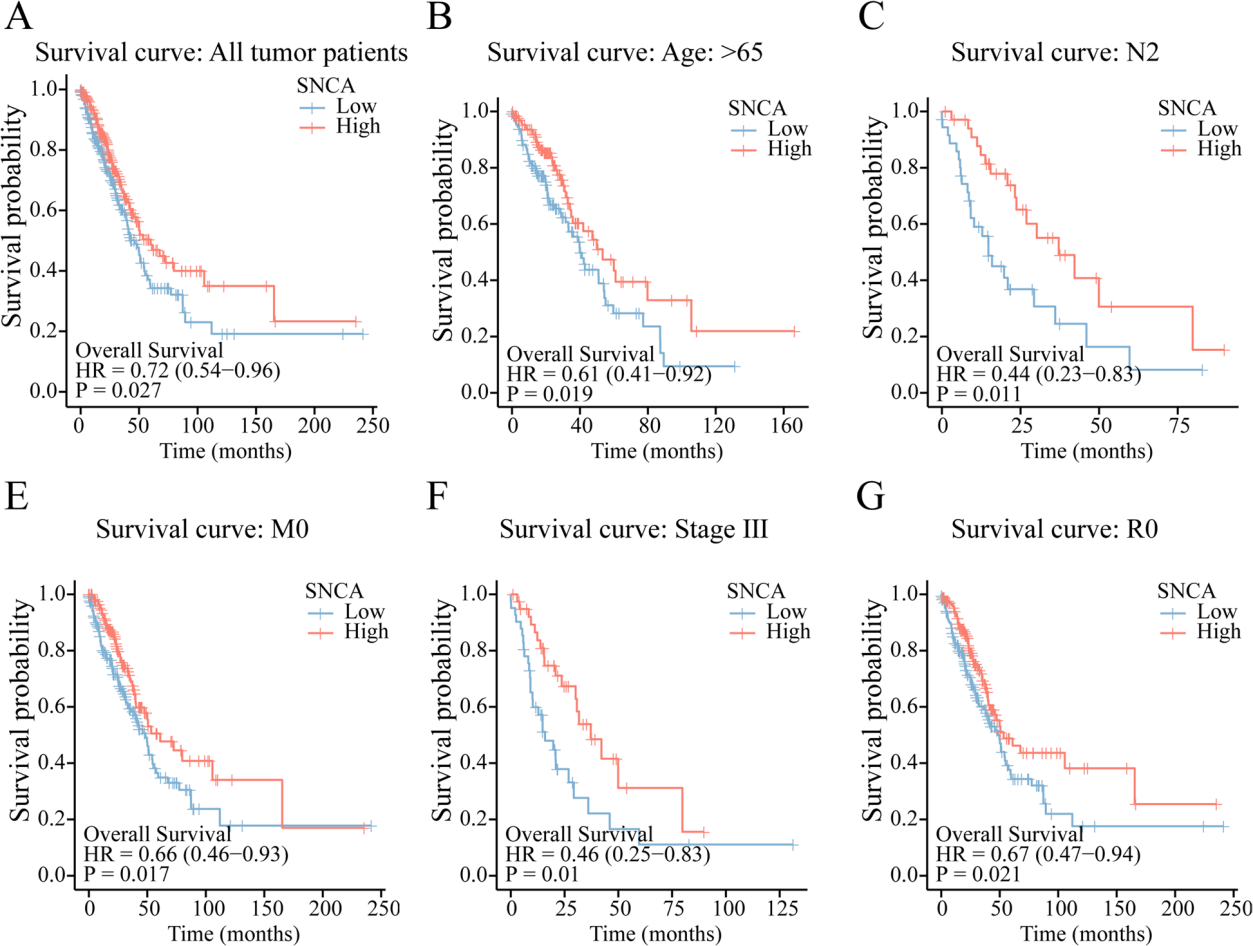
The correlation analysis between *SNCA* expression and immune infiltration, and the Kaplan–Meier survival curves of *SNCA* expression based on different immune cell subsets in LUAD. **A** The correlation between *SNCA* expression and the level of immune infiltration of B cells, CD8+ T cells, CD4+ T cells, macrophages, neutrophils, and dendritic cells in LUAD. **B**–**I** Correlation analysis of *SNCA* expression and the markers of dendritic cells, CD8+ T cells, macrophages, NK cells, regulatory T cells, neutrophils, B cells, monocytes, and neutrophils in LUAD. **J**–**Q** Relationship between *SNCA* expression and prognosis of LUAD in different immune cell subgroups (enriched / decreased CD4 + T cells, enriched / decreased macrophages, enriched / decreased NK cells, enriched /decreased CD8 + T cells). *P* < 0.05 was considered statistically significant

Immune infiltration as a component of the tumor microenvironment is usually associated with better prognosis and effect of immunotherapy [23]. Therefore, we explored the relationship between the transcription level of *SNCA* and immune infiltration in LUAD through the TIMER database. We found that there was a significant negative correlation between *SNCA* expression and tumor purity in LUAD (R = − 0.286, *P* = 9.68e-11) (Fig. 3A). Moreover, the expression of *SNCA* highly correlated with the infiltration levels of CD8 + T cells (R = 0.283, *P* = 1.92e-10), macrophages (R = 0.572, *P* = 2.10e-43), neutrophils (R = 0.463, *P* = 4.84e-27), and dendritic cells (R = 0.475, *P* = 6.99e-29). Furthermore, *SNCA* expression was also correlated with the B cell and CD4+ T cell (Fig. 3A). Next, we further explored the relationship between the expression of *SNCA* and markers of different immune cell types in LUAD. The cell type markers of dendritic cells, CD8+ T cells, macrophages, NK cells, Treg, B cells, monocytes, and neutrophils were analyzed. The results showed that *SNCA* in LUAD positively correlated with CD209 in dendritic cells; with CD8A and CD8B in CD8+ T cells; with CD68, CD84, and MS4A4A in macrophages; with NCR1 in NK cells; with CCR8 and FOXP in Treg; with CD19, MS4A1, and FCRL2 in B cells; with C3AR1, CD86, and CSF1R in monocytes; and with FCGR3B, FPR1, and SIGLEC5 in neutrophils (Fig. 3B–I). The data sets GSE68571, GSE104797 and GSE10072 were further used to verify the correction between the expression of *SNCA* and tumor microenvironment cells. As shown in Supplementary Table S3, the *SNCA* is most positively correlated with FPR1, which is reported the plague receptor on host immune cells [27], as well as CD84 and CD8B. Meanwhile the CD209, MS4A1 and FOXP3 were negatively correlated with expression of *SNCA*.

### Prognostic analysis of *SNCA* expression in LUAD based on immune cells

Previously, we confirmed that the expression of *SNCA* was related to the good prognosis of LUAD and also confirmed that the expression of *SNCA* was related to the immune infiltration of LUAD. Therefore, we speculated that the immune infiltration is one of the factors influencing the prognostic value of *SNCA* in LUAD. We further conducted prognostic analysis for *SNCA* expression levels in tumors with different immune cell subsets in LUAD by the Kaplan-Meier Plotter. The results showed that high expression of *SNCA* in LUAD resulted in a better prognosis in enriched CD4+ T cells (*P* = 0.0051) and enriched macrophages (*P* = 0.0057) respectively (Fig. 3J, L). Similarly, those with decreased numbers of NK cells (*P* = 0.016) and CD8+ T cells (*P* = 0.0014) also had a better prognosis (Fig. 3O, Q). However, decreased CD4+ T cells, decreased macrophages, enriched NK cells, and enriched CD8+ T cells have showed no significant correlation with the prognosis of LUAD (Fig. 3K, M, N, P). These analyses suggest that the influence of high expression of *SNCA* in LUAD on the prognosis of patients may be due to partial involvement of immune infiltration.

### *SNCA* is associated with immune checkpoints in LUAD

**Fig. 4:**
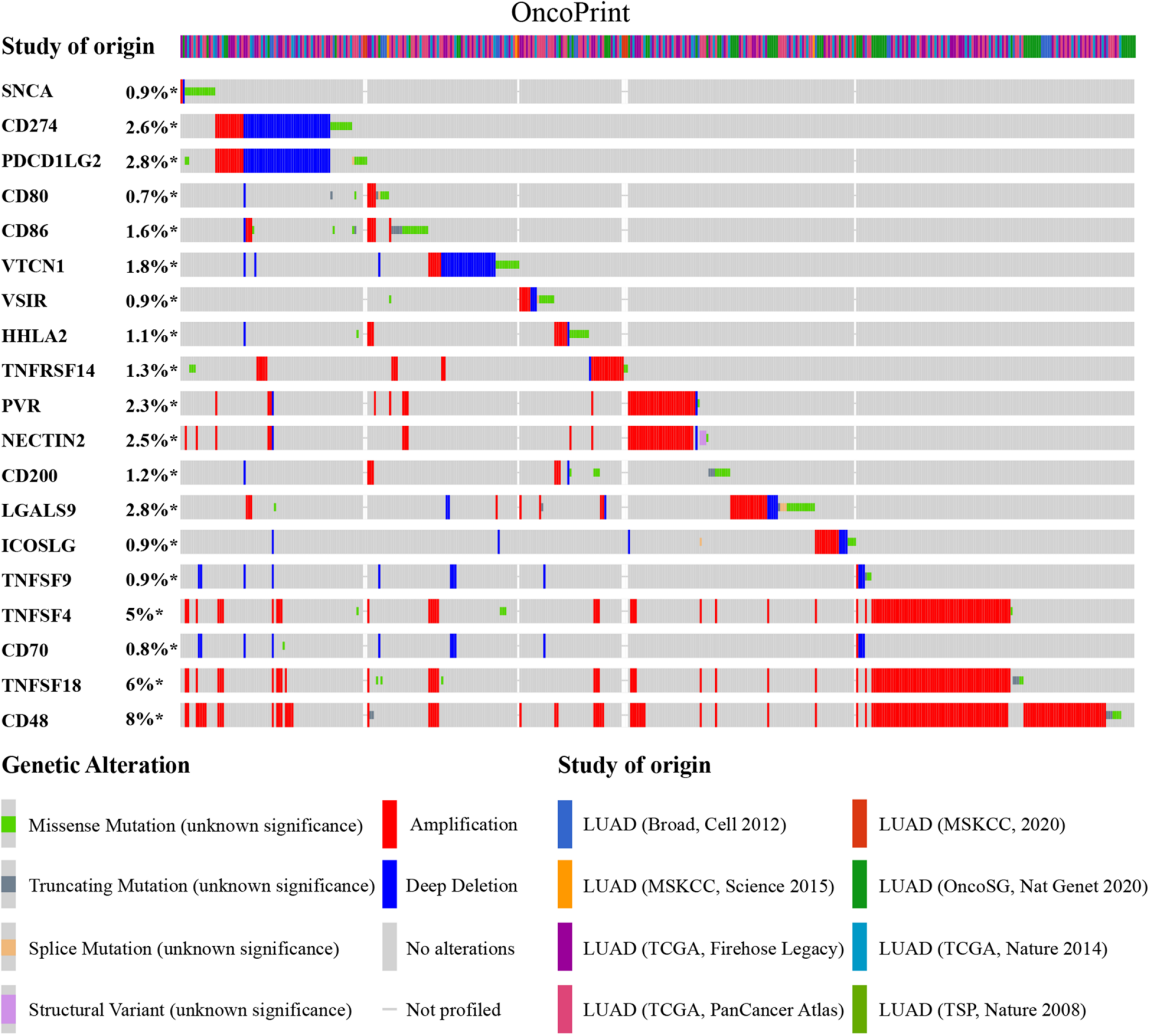
Landscape of *SNCA* and immune checkpoint alteration in LUAD. Compact visualization of cases with multiple genetic alterations of *SNCA* and immune checkpoints is individually shown by cBioPortal, including fusion, amplification, deep deletion, truncating mutation, and missense mutation

Genomic studies have revealed that *SNCA* is actually involved in the alteration of immune checkpoints in LUAD. The general landscapes of *SNCA* and immune checkpoint alteration in LUAD were analyzed using the cBioPortal database, including amplification, deep deletion, missense mutations, truncating mutations, splice mutations, and structural variant (Fig. 4). The detailed relationship between *SNCA* and representative immune checkpoints in LUAD is shown in Table 1. Of concern, *SNCA* alteration was associated with a wide range of immune checkpoints, such as *CD48*, *CD70*, *TNFRSF14*, *TNFRSF9*, *TNFRSF4*, and *TNFRSF18* with statistically significant co-occurrence. However, there was no statistically significant mutual exclusivity (Table 1). These results suggest that *SNCA* is a potential co-regulator of immune checkpoints in LUAD. A part of the beneficial effects of *SNCA* in LUAD may be derived from antitumor immunity.

**Table 1:** Mutual-exclusivity analysis between *SNCA* and multiple-immune checkpoints in LUAD

A	B	Neither	Log2 Odds Ratio	*p*-Value	Tendency	Significant
SNCA	CD48	1354	> 3	< 0.001	Co-occurrence	Yes
SNCA	TNFRSF14	1454	> 3	0.002	Co-occurrence	Yes
SNCA	CD70	1466	> 3	0.006	Co-occurrence	Yes
SNCA	TNFSF9	1464	> 3	0.008	Co-occurrence	Yes
SNCA	TNFSF4	1399	2.183	0.042	Co-occurrence	Yes
SNCA	TNFSF18	1394	2.087	0.049	Co-occurrence	Yes
SNCA	CD274	1424	<−3	0.586	Mutual exclusivity	No
SNCA	LGALS9	1433	<−3	0.643	Mutual exclusivity	No
SNCA	VTCN1	1438	<−3	0.678	Mutual exclusivity	No
SNCA	PVR	1440	<−3	0.692	Mutual exclusivity	No
SNCA	CD86	1450	<−3	0.767	Mutual exclusivity	No
SNCA	CD200	1456	<−3	0.816	Mutual exclusivity	No
SNCA	ICOSLG	1456	<−3	0.816	Mutual exclusivity	No
SNCA	HHLA2	1459	<−3	0.841	Mutual exclusivity	No

The relationship between *SNCA* and each immune checkpoint, expressed in terms of co-occurrence and mutual exclusivity. *p*- value < 0.05 is defined as statistically significant

### Identification of the immune-related DEGs regulated by *SNCA* in LUAD

**Fig. 5:**
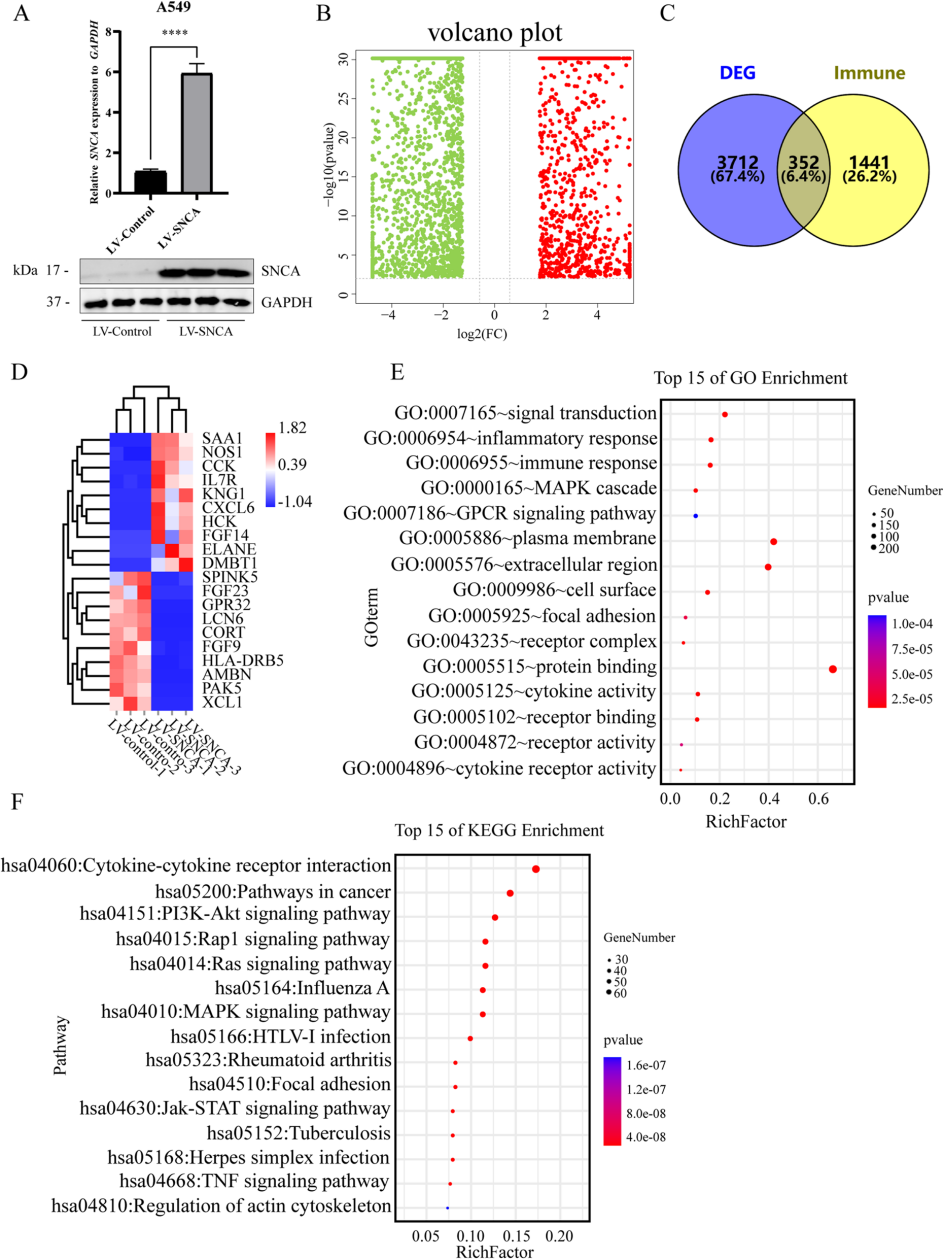
Genes related to immune regulation by *SNCA* in LUAD. **A** mRNA and protein levels of *SNCA* in the A549 cell line overexpressing *SNCA* were detected. **B** Volcanic map distribution of DEGs. Red dots are upregulated genes, and green dots are downregulated genes. **C** SNCA-regulated immune-related DEGs were obtained by Wayne analysis between the obtained DEGs and immune-related genes from Immport Resource. **D** Heat map showing the top 20 upregulated and top 20 downregulated immune-related DEGs. **E** GO analysis of immune-related DEGs, including five BP items, CC items, and MF items from top to bottom. **F** KEGG analysis of the top 15 immune-related DEGs (**** *P* < 0.0001)

To explore the function of *SNCA* in LUAD, we constructed A549 cells overexpressing *SNCA* by lentiviral vector (Fig. 5A). Through transcriptome sequencing analysis and using the screening criteria of *P* < 0.01 and | log FC | > 1.5, we identified 4064 DEGs, including 1708 upregulated genes and 2356 downregulated genes (Fig. 5B) (Supplementary Table S3). In order to explore the possible role of *SNCA* in LUAD immunization, we analyzed the intersection of immune-related genes from Immport Resource (https://www.immport.org/resources) with the identified DEGs and obtained 352 immune-related DEGs affected by *SNCA* (Fig. 5C) (Supplementary Table S4). The heat map shows the top 20 upregulated and top 20 downregulated immune-related DEGs. The clusters of DEGs are shown on the left side of the heat map, and the clusters of samples are shown on the top of the heat map (Fig. 5D). We used the DAVID tool to perform GO and KEGG enrichment analysis on these 351 immune-related DEGs (Supplementary Table S4). GO analysis is divided into three functional groups: biological processes (BP), cell components (CC), and molecular functions (MF). The GO analysis results showed that the changes of BP were significantly enriched in signal transduction, inflammatory response, immune response, MAPK cascade, and G-protein coupled receptor signaling pathway. The changes of CC were enriched in plasma membrane, extracellular region, cell surface, focal adhesion, receptor complex, and other aspects. MF changes mainly involved protein binding, cytokine activity, receptor binding, receptor activity, and cytokine receptor activity (Fig. 5E and Supplementary Table S5). KEGG pathway analysis showed that immune-related DEGs regulated by *SNCA* were mainly enriched in cytokine–cytokine receptor interaction, PI3K-Akt signaling pathway, Rap1 signaling pathway, Ras signaling pathway, MAPK signaling pathway, focal adhesion, and Jak-STAT signaling pathway (Fig. 5F and Supplementary Table S5).

**Fig. 6:**
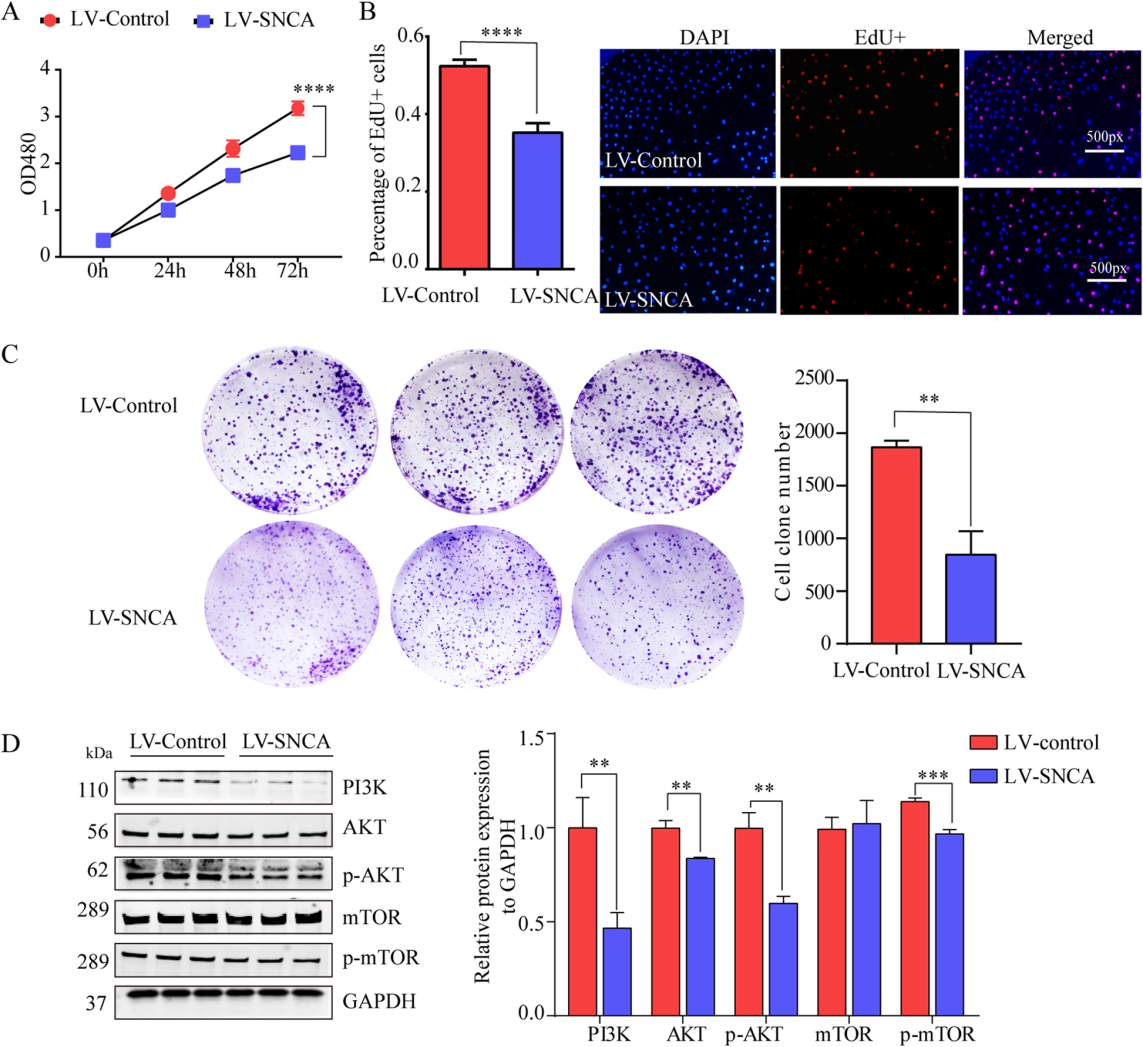
*SNCA* affects proliferation of LUAD cells in vitro via inhibiting PI3K/AKT/mTOR signaling pathway. **A** Proliferation of A549 cells overexpressing *SNCA* was assessed by CCK8; *** *P* < 0.001. **B** Effect of *SNCA* on A549 cell growth were evaluated by EdU assay (*n* = 5). Results of quantitative analysis were measured by Image J; **** *P* < 0.0001. **C** Effects of *SNCA* on A549 cell growth were further evaluated using a colony formation assay. Quantitative analysis of colony numbers is measured by Image J; ** *P* < 0.01. **D** Levels of PI3K, AKT, p-AKT, mTOR and p-mTOR proteins were assessed in stably-transfected A549 cells by Western blotting; ** *P* < 0.01, *** *P* < 0.001

To further study whether *SNCA* takes part in tumorigenesis of LUAD, we used CCK-8, EdU assays and colony formation to assess the effects of *SNCA* on the proliferation of A549 cells. The results of CCK-8 assay showed that the OD480 value on SNCA-overexpressing cells was significantly lower than that in empty vector controls after cultured for 72 h (Fig. 6A), indicating that the growth of A549 cells overexpressing *SNCA* were inhibited. Similar results were obtained using an EdU assay (Fig. 6B). In the colony formation assay showed that the proliferation of SNCA-overexpressing A549 cells was significantly decreased (Fig. 6C). There results suggest that *SNCA* exerted an inhibitory effect on the proliferation of LUAD cells. PI3K/AKT pathway was reported to regulating tumor cell proliferation and we have identified *SNCA* may participate in PI3K/AKT pathway (Fig. 5F). We measures PI3K, AKT, p-AKT (Ser at 473 aa), mTOR and p-mTOR (Ser at 2448 aa) levels in A549 cells stably-transfected with *SNCA*. Our results showed that *SNCA* significantly decreased the levels of PI3K, AKT, p-AKT and p-mTOR, but had no effect on mTOR levels (Fig. 6D). There results indicated that *SNCA* inhibited growth in LUAD depend on the PI3K/AKT/mTOR signaling pathway.

### The identification of key genes

**Fig. 7:**
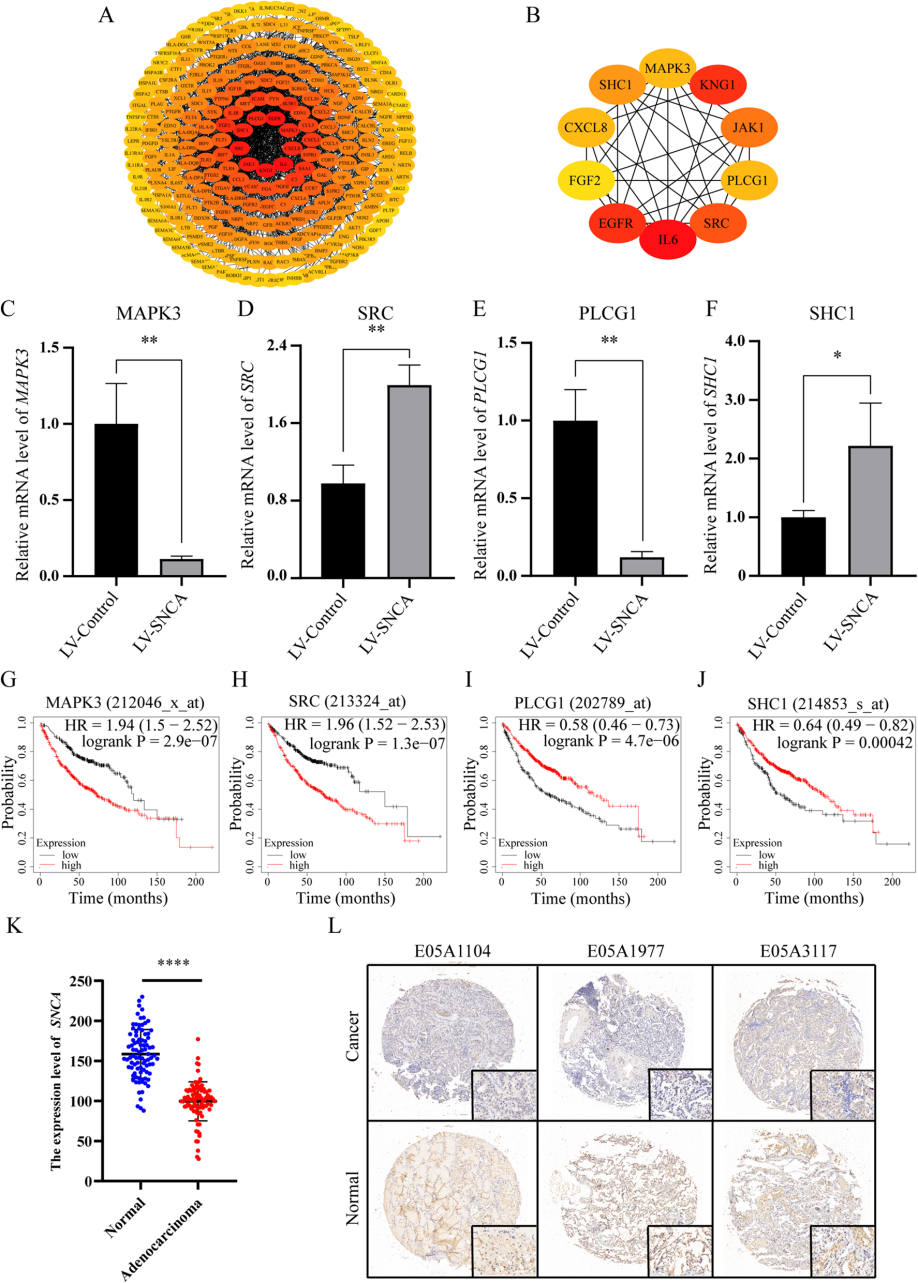
The screening of the key genes and analysis of *SNCA* in LUAD clinical samples. **A** Protein interaction network constructed by immune-related DEGs through Cytoscape. The orange node is the node with high degree, and the yellow node is the node with low degree. **B** The interaction network diagram of 10 node genes with the highest degree screened by Cytohubba application in Cytoscape. **C**–**F** qPCR method was used to verify the expression of four central genes in SNCA-overexpressing A549 cell line. **G**–**J** Kaplan–Meier curve was used to analyze the prognosis of four central genes in LUAD. **K** Statistical analysis of *SNCA* immunostaining score in clinical samples, ****P < 0.0001. **L** Immunohistochemical images of *SNCA* in clinical samples

In order to screen the key genes regulated by *SNCA*, we constructed a PPI network with 255 nodes and 1814 edges by STRING using 352 DEGs (Fig. 7A and Supplementary Table S6). Then, the Cytohubba application from Cytoscape was used to identify 10 hub genes with the highest degree in DEGs, namely, *IL6*, *EGFR*, *KNG1*, *SRC*, *JAK1*, *SHC1*, *PLCG1*, *MAPK3*, *CXCL8*, and *FGF2* (Fig. 7B). Next, we verified the expression of these 10 hub genes in the A549 cells overexpressing *SNCA*, by RT-qPCR. The results showed that the expression levels of *MAPK3*, *SRC*, *PLCG1*, and *SHC1* were consistent with the transcriptome sequencing results (Fig. 7C–F). We also showed that in the A549 cells, *SNCA* significantly inhibited the expression of *MAPK3* and *PLCG1* and significantly upregulated the expression of *SRC* and *SHC1* (Fig. 7C–F). We further analyzed the expression levels of these four genes and their effects on survival prognosis by the Kaplan–Meier Plotter; the results showed that these four genes significantly correlated with the prognosis of LUAD (Fig. 7G–J).

### *SNCA* expression is downregulated in LUAD clinical samples

We purchased a commercial tissue microarray containing 93 pairs of cancer tissues and adjacent tissues of LUAD (OUTDO BIOTECH, China) and verified the expression of *SNCA* by immunohistochemical method (Table 2). Immunohistochemistry score (H-Score) was determined on the immunostaining results using Image-Pro Plus 6.0 software. Statistical results showed that compared with the paracancerous tissues, *SNCA* expression was significantly downregulated in cancer tissues (*P* < 0.0001) (Fig. 7K). Figure 7L shows the immunohistochemical graph. The results were consistent with the results of LUAD data sets in TCGA, emphasizing that *SNCA* expression was significantly downregulated in LUAD.

**Table 2:** Patient clinical characteristics at presentation

Clinical characteristics		Total (78)	%	High	Low	*P*-Value
Age	> = 65	33	42.3	19	14	0.252
	< 65	45	57.7	20	25	
Gender	Male	42	53.8	17	25	0.069
	Female	36	46.2	22	14	
Stage	I	23	37.1	14	9	0.752
	II	17	27.4	9	8	
	III/IV	22	35.5	11	11	
T stage	T1	21	28.4	13	8	0.091
	T2	35	47.3	13	22	
	T3	12	16.2	7	5	
	T4	6	8.1	5	1	
N stage	N0	30	62.5	18	12	0.462
	N1	12	25	6	6	
	N2/N3	6	12.5	2	4	
Pleural invasion	Negative	67	85.9	33	34	0.745
	Positive	11	14.1	6	5	
ALK	Negative	65	87.8	34	31	0.658
	Positive	9	12.2	4	5	
EGFR	Negative	59	75.6	26	33	0.065
	Positive	19	24.4	13	6	

Seventy-eight patients with lung adenocarcinoma with complete follow-up data were selected as the research objects. The staging was in accordance with the 7th edition of the AJCC Cancer Staging Manual. The follow-up ended in July 2016. The Pearson correlation coefficient by SPSS was used to analyze the relationships among *SNCA* expression and clinical characteristics of LUAD patients

### The lower expression of *SNCA* in LUAD is related to the increased methylation of its promoter

**Fig. 8:**
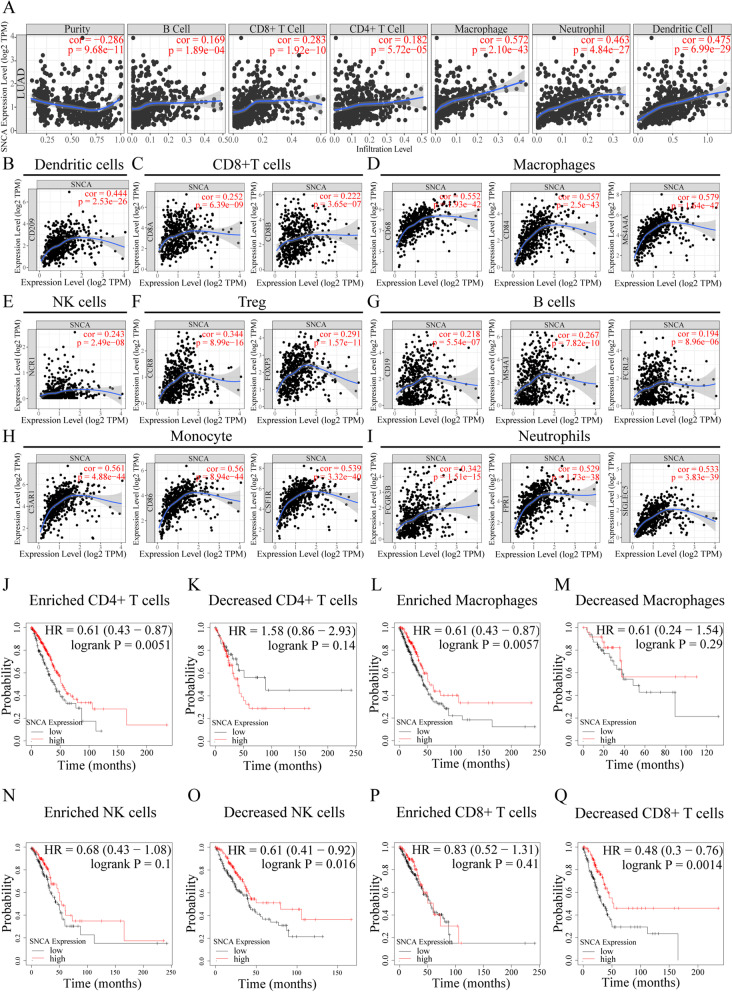
The analysis of *SNCA* promoter methylation level in LUAD. **A** The correlation analysis between the expression level of *SNCA* in LUAD samples and the methylation level of cg06632027 methylation site. **B** The analysis of methylation levels of four genomic fragments of *SNCA* promoter in tumor samples and normal samples in LUAD patient samples of the DiseaseMeth database. The expression level of *SNCA* methylation in LUAD patient samples downloaded from UALCAN database was analyzed with (**C**) sample type, (**D**) TNM stage, and (**E**) lymph node status. **F** After A549 and H1299 cells were treated with 5-aza-dC, the mRNA expression level of *SNCA* was detected by qPCR (* *P* < 0.05, ** *P* < 0.01, *** *P* < 0.001, **** *P* < 0.0001)

DNA methylation is one of the most important epigenetic mechanisms, which tightly regulates gene expression [1, 2]. In the development of tumor, DNA hypermethylation occurs in the tumor suppressor gene promoter region, while hypomethylation occurs in the oncogene promoter region. Therefore, abnormal DNA methylation is often used as an important molecular marker for tumor diagnosis, classification, and treatment. Our previous study confirmed that the transcriptional level and protein expression level of *SNCA* were significantly inhibited in LUAD, and we speculated whether it was related to the methylation level of its DNA promoter region. We selected the most statistically significant methylation site CG06632027 from Wanderer database for analysis; the results showed that the expression level of *SNCA* significantly negatively correlated with the methylation level of its promoter, and the expression of *SNCA* decreased with the increase of methylation level in LUAD tissues (*P* = 8.25E-15, Spearman = − 0.274) (Fig. 8A). Four genome fragments of the *SNCA* promoter of LUAD were selected and analyzed. As shown in Fig. 8B, the methylation levels of the four genomic fragments of the *SNCA* promoter in the normal samples were generally low, but the average methylation levels of the tumor samples were higher. In addition, data from the UALCAN database showed that the total methylation levels of *SNCA* in LUAD patients were significantly higher than those in the normal population (Fig. 8C). Subsequently, we further explored the correlation between *SNCA* methylation level and clinicopathological parameters of LUAD patients through UALCAN database. Higher methylation values of *SNCA* in LUAD patients significantly correlated with advanced TNM staging (*P* < 0.05) and lymph node metastasis *(P* < 0.0001) (Fig. 8D, E). Therefore, these results suggest that DNA hypermethylation may cause a decrease in *SNCA* gene expression at the transcriptional level. 5-aza-dC (Sigma, USA) — a DNA demethylation drug that could inhibit the methylation level of the genome to activate gene expression, was used to treat A549 and H1299 cells, at a final concentration of 5 μM for 72 h. The changes in *SNCA* transcription level were detected by RT-qPCR. The results showed that the mRNA levels of *SNCA* in the 5-aza-dC-treated group were significantly higher than those in the solvent-treated group (Fig. 8F), suggesting that the high methylation of *SNCA* promoter in LUAD was one of the reasons for the downregulation of *SNCA* mRNA level. The high methylation level of *SNCA* can also be used as a biomarker of LUAD.

## Discussion

LUAD is the most common type of lung cancer, accounting for about 40% of lung cancer cases. Although some progress has been made in exploring the mechanism and treatment of this disease in recent years, the total survival time of patients is still less than 5 years due to the tumor’s high invasiveness and lethality [28]. Therefore, the screening of molecular markers for the invasion and prognosis of LUAD is particularly important. Previous studies have reported that *SNCA* is downregulated in LUAD tissues and cell lines [14]. We also confirmed that *SNCA* was downregulated in LUAD, through multiplatform data analysis and clinical sample detection (Figs. 1 and 7), suggesting that *SNCA* may be used as a molecular diagnostic marker or a molecular therapeutic target for LUAD.

According to our results, there are differences in *SNCA* expression level among different tumors (Fig. 1), indicating that *SNCA* may play different roles, and it seems to be a “double-edged sword”. Meta-analyses of transcriptome data have shown significant overlap between genes upregulated in neurodegenerative diseases and those downregulated in tumors, and vice versa [29] . This is also true for *SNCA* in LUAD [14]. However, there are some exceptions. According to Fig. 1B, *SNCA* is upregulated in skin melanoma (SKCM), pheochromocytoma, and paraganglioma (PCPG). The incidence of SKCM in PD patients is very high, and the incidence of PD in SKCM patients is also higher [30–32]. The link between PD and SKCM involves the alteration of melanin and melanin biosynthase [33]. Surprisingly, upregulation of *SNCA* inhibits melanin synthesis in melanocytes and increases the risk of SKCM [31]. It has been proposed that neurodegenerative diseases and tumors share specific genetic and cellular changes that involve common molecular pathways, such as cell cycle and autophagy regulation [34]. Therefore, the study of *SNCA* in LUAD can also refer to the association between neurodegenerative diseases and tumors. According to data sets of LUAD in PrognoScan database (http://dna00.bio.kyutech.ac.jp/PrognoScan/index.html), *SNCA* has significant prognostic significance in bladder cancer, acute myeloid leukemia, astrocytoma, breast cancer, and other diseases [35–38]. The biological function of *SNCA* in human tumors has not been repeatedly mentioned [39–41], and clear reports on the relationship between *SNCA* expression and clinical outcomes in these diseases are still rare. In this study, we explored the effect of *SNCA* expression on the overall survival rate and prognosis of subgroups of clinical characteristics in LUAD patients, confirming that high expression of *SNCA* is a marker of good prognosis of LUAD (Fig. 2) and suggesting that *SNCA* may play a beneficial protective role against LUAD.

Lung tissue contains abundant immune cells, which form a strong first line of defense against foreign particles and microorganisms. The malignant phenotype of LUAD is not only defined by intrinsic tumor cells but also has a relationship with the degree of immune infiltration in LUAD [42]. Therefore, more detailed understanding of the immune microenvironment is of great significance for the diagnosis and treatment of LUAD. A key finding in this study is that the expression of *SNCA* highly correlated with the immune infiltration in LUAD. *SNCA* expression correlated strongly positively with macrophages, neutrophils, and dendritic cells and weakly positively with CD8+, CD4+, and B cells (Fig. 3). The correlation between *SNCA* expression and immune cell marker genes suggests that *SNCA* plays a role in regulating LUAD tumor immunology. Firstly, the gene marker *CD209* of dendritic cells showed a strong positive correlation with *SNCA* expression. Secondly, gene markers *CD68*, *CD84*, and *MS4A4A* of macrophages showed a strong positive correlation with *SNCA* expression. The gene markers *C3AR1*, *CD86*, and *CSF1R* in monocytes and *FPR1* and *SIGLEC5* in neutrophils also showed a strong positive correlation with *SNCA* expression (Fig. 3). Tumor associated macrophages (TAMs) are the most abundant infiltrating inflammatory cells in the tumor microenvironment (TME), which are closely related to the occurrence and development of various tumors [43, 44]. Dendritic cells constitute a rare immune cell group in tumor and lymphatic organs, but they are essential for the initiation of antigen-specific immunity and tolerance [45]. Dendritic cells in tumors promote immunity or tolerance by sampling and presenting antigens to T cells and providing immuneregulatory signals through cell–cell contact and cytokines [46]. The role of neutrophils in tumors is controversial as they may act as inert bystanders or promote/inhibit tumor development. Tumors manipulate neutrophils, sometimes in the early stages of their differentiation, to produce multiple phenotypes and functional polarizations that alter tumor behavior. However, intravenous injection of neutrophils inhibited tumor metastasis [47]. The effects of *SNCA* expression on LUAD prognosis were analyzed based on immune cells. The high expression of *SNCA* showed better prognosis in LUAD rich in CD4+ T cells and macrophages. These findings jointly indicate that *SNCA* plays an important role in the recruitment and regulation of immune infiltrating cells in LUAD.

In our study, *SNCA* may be involved in the alteration of LUAD immune checkpoints. Moreover, *SNCA* and extensive immune checkpoint changes showed statistically significant synergy rather than mutual exclusion (Table 1). *SNCA* had the highest correlation with *CD48*. *CD48* encodes members of the CD2 subfamily of immunoglobulin-like receptors, which include signaling lymphocyte activation molecule (SLAM) proteins. The encoded proteins are present on the surfaces of lymphocytes and other immune cells, dendritic cells, and endothelial cells and are involved in the activation and differentiation pathways of these cells [48]. This seems to explain the strong positive correlation between *SNCA* and immune-infiltrating cells. CD48 has been reported to be involved in antigen processing and presentation, T cell co-stimulation, and T cell receptor signaling pathways in glioma (GBM). GBM with high CD48 is associated with antigen processing and MHC I presentation, which interacts with CD8+ T cells and is involved in immunosuppression [49]. Treatment of advanced (metastatic) NSCLC is currently based on immunotherapy using anti-PD-1 or PD-L1 antibodies, alone or in combination with chemotherapy, but it is accompanied with low response rates and very short median progression-free survival [50]. This suggests that in addition to PD-1 / PD-L1, other checkpoints that may play an important role in tumor immunity in LUAD remain to be identified. Perhaps, *SNCA* and *CD48* as targets can make up for the lack of immunosuppressive therapy in LUAD. Of course, the specific mechanism of *SNCA* and its interaction with *CD48* should be clarified, and further biological experiments are needed.

In order to further study the possible mechanism of *SNCA* in LUAD immunity, we constructed an LUAD cell line overexpressing *SNCA* for transcriptome analysis and screened 352 immune-related DEGs regulated by *SNCA*. BP, CC, and MF enrichment analysis showed that DEGs were mainly enriched in the plasma membrane, extracellular, cell surface, protein binding, receptor binding, receptor activity, and adhesive plaque. These results showed that these DEGs were involved in intercellular signal transduction and migration in LUAD. The KEGG pathway analysis showed that these integrated DEGs were mainly enriched in the following five pathways: phosphatidylinositol 3-kinase/protein kinase B (PI3K-Akt) signaling pathway, Rap1 signaling pathway, Ras signaling pathway, MAPK signaling pathway, and focal adhesion (Fig. 5). These signaling pathways have been shown to play important roles in tumorigenesis. Overactivation of the PI3K/Akt signaling pathway can promote malignant transformation of cells by regulating tumor cell proliferation, apoptosis, migration, invasion, angiogenesis, immune evasion, and drug resistance [51]. Rap1 plays an important role in cell adhesion and integrin function in a variety of cell types. Ras gene is considered a carcinogenic gene. Rap1 and Ras have high sequence similarity and overlapping binding objects. Ras and Rap1 cooperate to start and maintain ERK signals to promote the invasion, cell migration, and metastasis of various types of cancer [52]. MAPK pathway is one of the most important pathways for cell proliferation. Inhibition of MAPK pathway can usually hinder the development of cancer [53]. Among them, the Ras/raf/mapk (mek)/erk pathway is the most important cascade signal in all MAPK pathways, which plays a vital role in the survival and development of tumor cells [54]. Focal adhesion can connect actin cytoskeleton and integrin to extracellular matrix. In recent years, a large number of studies have shown that focal adhesion-related structural molecules are involved in regulating the epithelial–mesenchymal transition process of tumor cells and promoting tumor invasion and metastasis. We studied the role of *SNCA* in the progression of LUAD and found that *SNCA* could inhibit proliferation of A549 cells via decreased the activity of PI3K, AKT and p-mTOR, indicating that *SNCA* could affect the growth of LUAD via inhibition PI3K/AKT/mTOR pathway (Fig. 6).

In addition, we further established the PPI network and screened four key genes closely related to *SNCA* that is *MAPK3 (ERK1)*, *SRC*, *PLCG1* and *SHCI*. RT-qPCR was used to verify that *SNCA* can significantly inhibit the levels of *MAPK3* and *PLCG1* and significantly promote the expression of *SRC* and *SHCI* (Fig. 7). The underlying molecular mechanism needs to be further studied.

It has been reported that the expression of *SNCA* is affected by epigenetic mechanisms in a variety of tumors, such as cholangiocarcinoma (CHOL) [55], colonic adenocarcinoma (COAD) [13], breast cancer [56], and non-Hodgkin lymphoma (NHL) [13] . In our study, we verified that the methylation level of *SNCA* in LUAD was significantly upregulated, which negatively correlated with the mRNA level. When LUAD cells were treated with the DNA demethylation drug 5-aza-dC, the mRNA level of *SNCA* significantly increased, suggesting that *SNCA* promoter methylation may be one of the reasons for *SNCA* downregulation at the mRNA level (Fig. 8). Moreover, DNA methylation level of *SNCA* is expected to be a biological diagnostic marker in LUAD.

In summary, by comprehensive bioinformatics analysis, this study confirmed that the expression level of *SNCA* is significantly reduced in LUAD, and the high expression of *SNCA* is related to immune infiltration and good prognosis, suggesting that immune infiltration may be one of the reasons for the influence of *SNCA* on prognosis in LUAD. Furthermore, by screening immune-related DEGs and analyzing the related pathways that may be involved in LUAD and the central genes that may be regulated, we confirmed that *SNCA* is involved in the key pathways for the occurrence and development of multiple tumors. The proliferation of A549 cells could be inhibited by *SNCA* via decreased the activity of PI3K/AKT/mTOR signaling pathway. These findings suggest that *SNCA* is a prognostic marker with potential research value in the diagnosis and treatment of LUAD. However, its specific regulatory mechanism in LUAD requires verification in additional biological experiments.

## Conclusion

*SNCA* is downregulated in LUAD and is negatively regulated by DNA methylation in LUAD. The high expression of *SNCA* is related to immune infiltration and good prognosis. Moreover, it is confirmed that *SNCA* is involved in the key pathways for the occurrence and development of LUAD. The proliferation of LUAD cells were inhibited by *SNCA* via decreased the activity of PI3K/AKT/mTOR signaling pathway. Therefore, *SNC*A could act as a promising prognostic biomarker in LUAD patients.

## Supplementary Information

**Additional file 1.**

**Additional file 2.**

**Additional file 3.**

**Additional file 4.**

**Additional file 5.**

**Additional file 6.**

**Additional file 7.**

**Additional file 8.**

## Data Availability

Most of the datasets analyzed during the current study are available in the Oncomine database (https://www.oncomine.org/resource/main.html), UALCAN database (http://ualcan.path.uab.edu/index.html), TIMER database (https://cistrome.shinyapps.io/timer/), cBioPortal database (www.cbioportal.org), TCGA database (https://tcga-data.nci.nih.gov/tcga/), The Kaplan–Meier plotter (http://kmplot.com/), STRING database (https://string-db.org/), DAVID database (https://david.ncifcrf.gov/), Immport Resource (https://www.immport.org/resources). KEGG database (www.kegg.jp/kegg/kegg1.html). The transcriptome datasets generated and analysed during the current study are not publicly available due to privacy but are available from the corresponding author on reasonable request.

## Abbreviations

PD: Parkinson’s disease
LUAD: Lung adenocarcinoma
NSCLC: Non-small-cell lung cancer
HR: Hazard ratio
DEGs: Differentially expressed genes
GO: Gene Ontology
KEGG: Kyoto Encyclopedia of Gene and Genomes
PPI: Protein protein interaction
RT-qPCR: Quantitative real-time PCR
IHC: Immunohistochemical
BLCA: Bladder urothelial carcinoma
COAD: Colon adenocarcinoma
GBM: Glioblastoma multiforme
KIRC: Kidney renal clear cell carcinoma
KIRP: Kidney renal papillary cell carcinoma
UCEC: Uterine corpus endometrial carcinoma
OS: Overall survival
BP: Biological processes
CC: Cell components
MF: Molecular functions
SKCM: Skin melanoma
PCPG: Pheochromocytoma, and paraganglioma
TAMs: Tumor associated macrophages
TME: Tumor microenvironment
SLAM: Signaling lymphocyte activation molecule
GBM: Glioma
CHOL: Cholangiocarcinoma
