# Correction to: *SNCA* correlates with immune infiltration and serves as a prognostic biomarker in lung adenocarcinoma

**DOI:** 10.1186/s12885-022-09544-x

**Published:** 2022-05-02

**Authors:** Xiuao Zhang, Zhengcun Wu, Kaili Ma

**Affiliations:** 1 Institute of Medical Biology, Chinese Academy of Medical Sciences and Peking Union Medical College, Kunming, 650118 China; 2 Neuroscience Center, Chinese Academy of Medical Sciences and Peking Union Medical College, Beijing, 100005 China; 3 Yunnan Key Laboratory of Vaccine Research Development on Severe Infectious Diseases, Kunming, 650118 China

**Correction: BMC Cancer 22, 406 (2022)**

**https://doi.org/10.1186/s12885-022-09289-7**

Following publication of the original article [1], the authors identified an error in the order of the figures. The correct order is given below. The original article [1] has been corrected.

Figs. 1, 2, 3, 4, 5, 6, 7 and 8

**Fig. 1:**
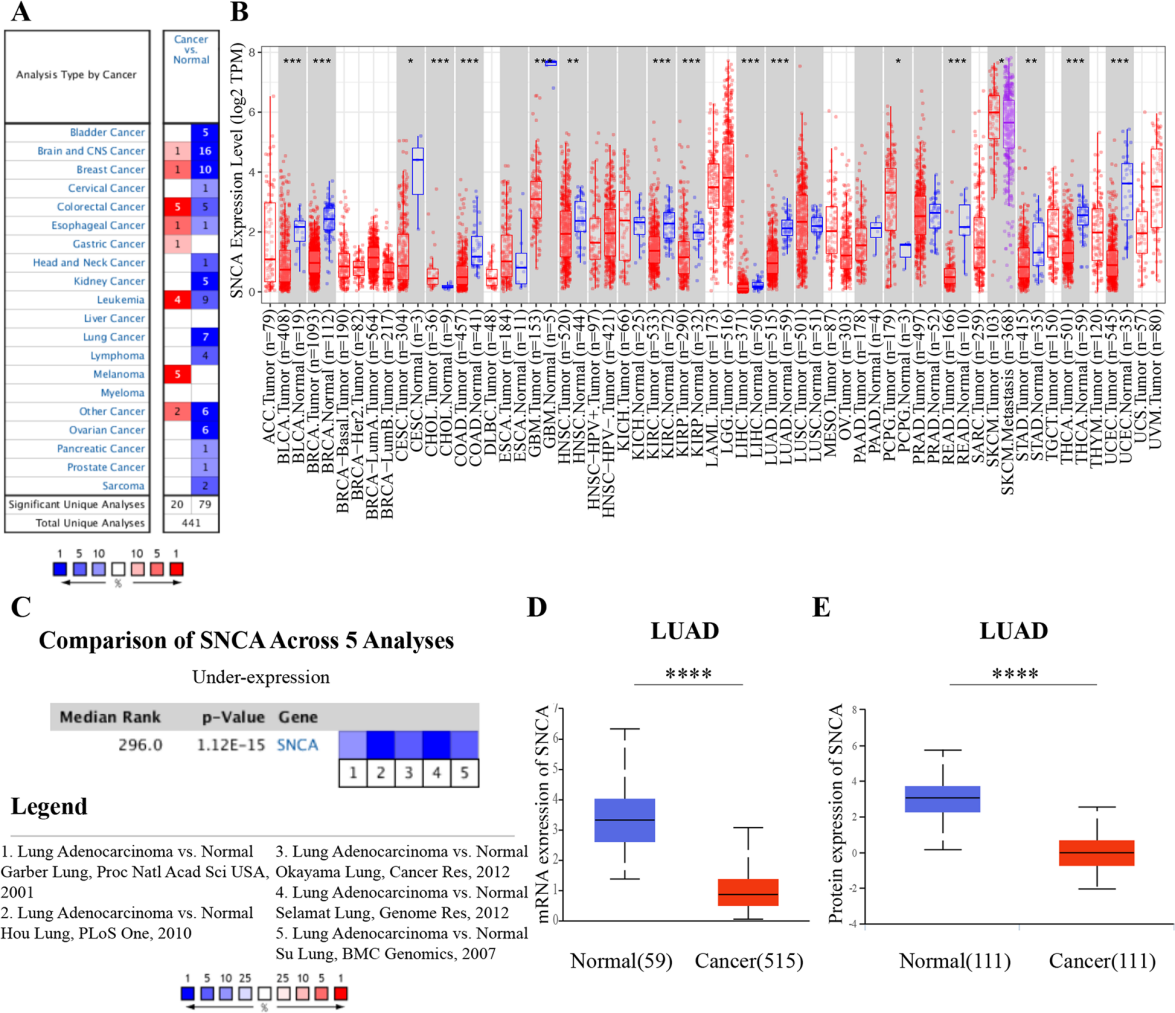
The expression of *SNCA* in different tumors was analyzed in Oncomine and TIMER databases. **A** Oncomine database was used to analyze the expression of *SNCA* gene in Pan-cancer species. Note: red represents upregulation of the target gene, while blue represents downregulation of the target gene. Threshold parameter: *p*-value is 0.01, fold change is 1.5. **B** The analysis of the expression level of *SNCA* in different tumors in TCGA through TIMER database. **C** Meta-analysis of *SNCA* expression was performed using five LUAD sequencing data sets through Oncomine database. **D** UALCAN database analysis of the mRNA expression level of *SNCA* in LUAD (**** *P* < 0.0001). **E** According to the analysis of UALCAN database, the protein expression level of *SNCA* in LUAD (**** *P* < 0.0001)

**Fig. 2:**
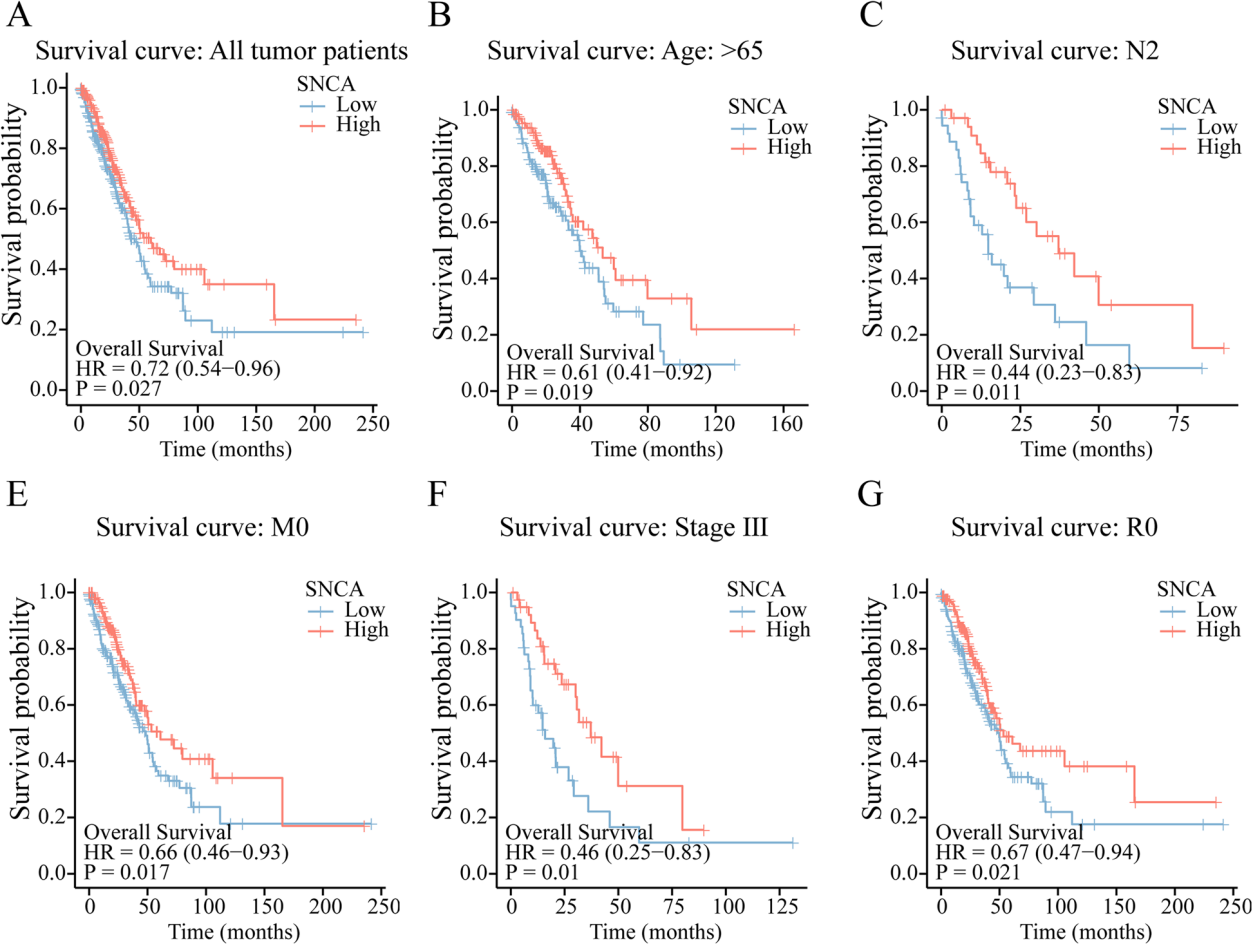
Kaplan–Meier curve analysis of the influence of *SNCA* expression level in LUAD on the overall survival rate. **A** Kaplan–Meier curves of *SNCA* in all tumor patients. **B**–**F** Subgroup analysis based on age (> 65), N2, M0, stage III, and R0

**Fig. 3:**
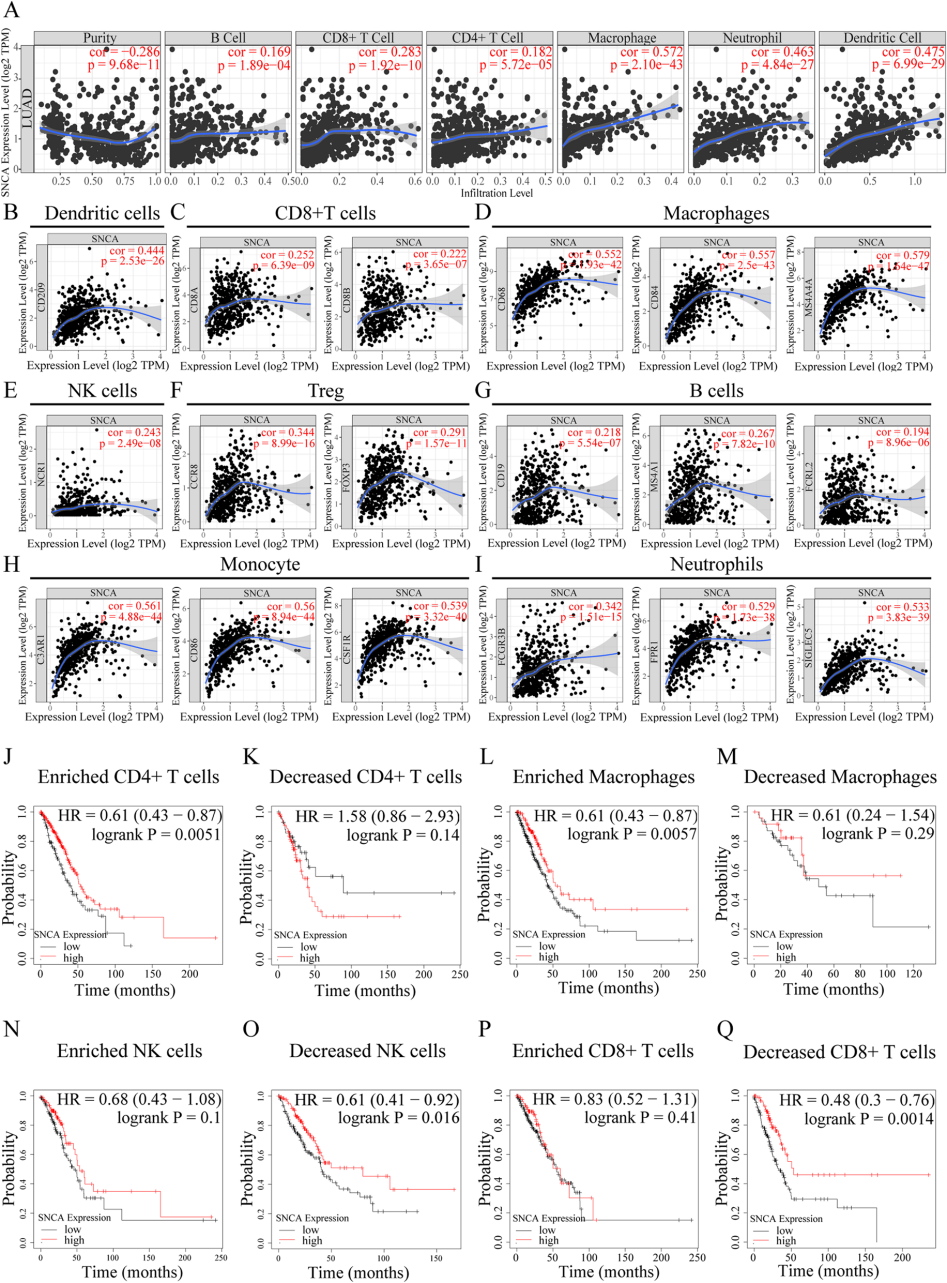
The correlation analysis between *SNCA* expression and immune infiltration, and the Kaplan–Meier survival curves of *SNCA* expression based on different immune cell subsets in LUAD. **A** The correlation between *SNCA* expression and the level of immune infiltration of B cells, CD8+ T cells, CD4+ T cells, macrophages, neutrophils, and dendritic cells in LUAD. **B**–**I** Correlation analysis of *SNCA* expression and the markers of dendritic cells, CD8+ T cells, macrophages, NK cells, regulatory T cells, neutrophils, B cells, monocytes, and neutrophils in LUAD. **J**–**Q** Relationship between *SNCA* expression and prognosis of LUAD in different immune cell subgroups (enriched / decreased CD4 + T cells, enriched / decreased macrophages, enriched / decreased NK cells, enriched /decreased CD8 + T cells). *P* < 0.05 was considered statistically significant

**Fig. 4:**
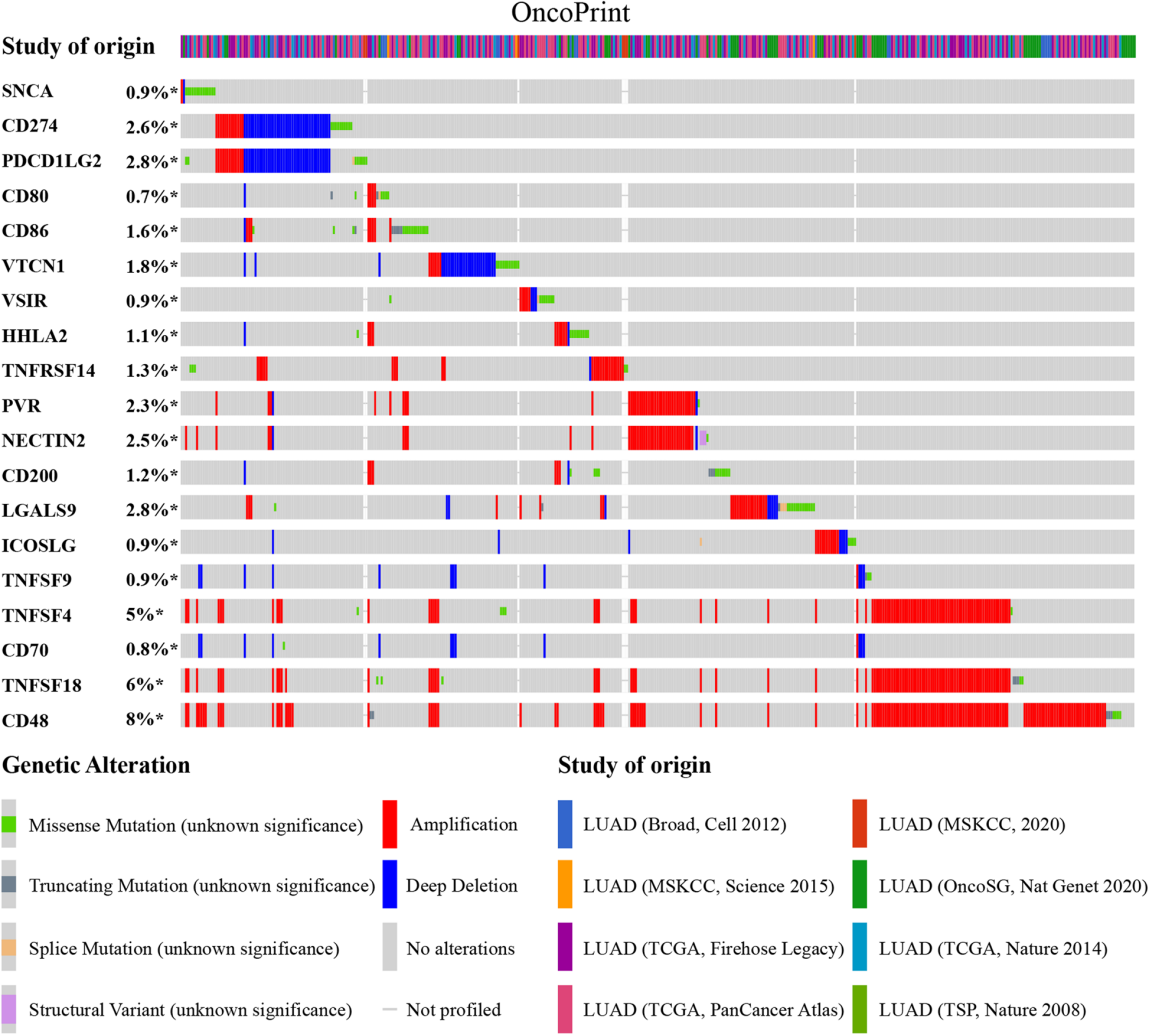
Landscape of *SNCA* and immune checkpoint alteration in LUAD. Compact visualization of cases with multiple genetic alterations of *SNCA* and immune checkpoints is individually shown by cBioPortal, including fusion, amplification, deep deletion, truncating mutation, and missense mutation

**Fig. 5:**
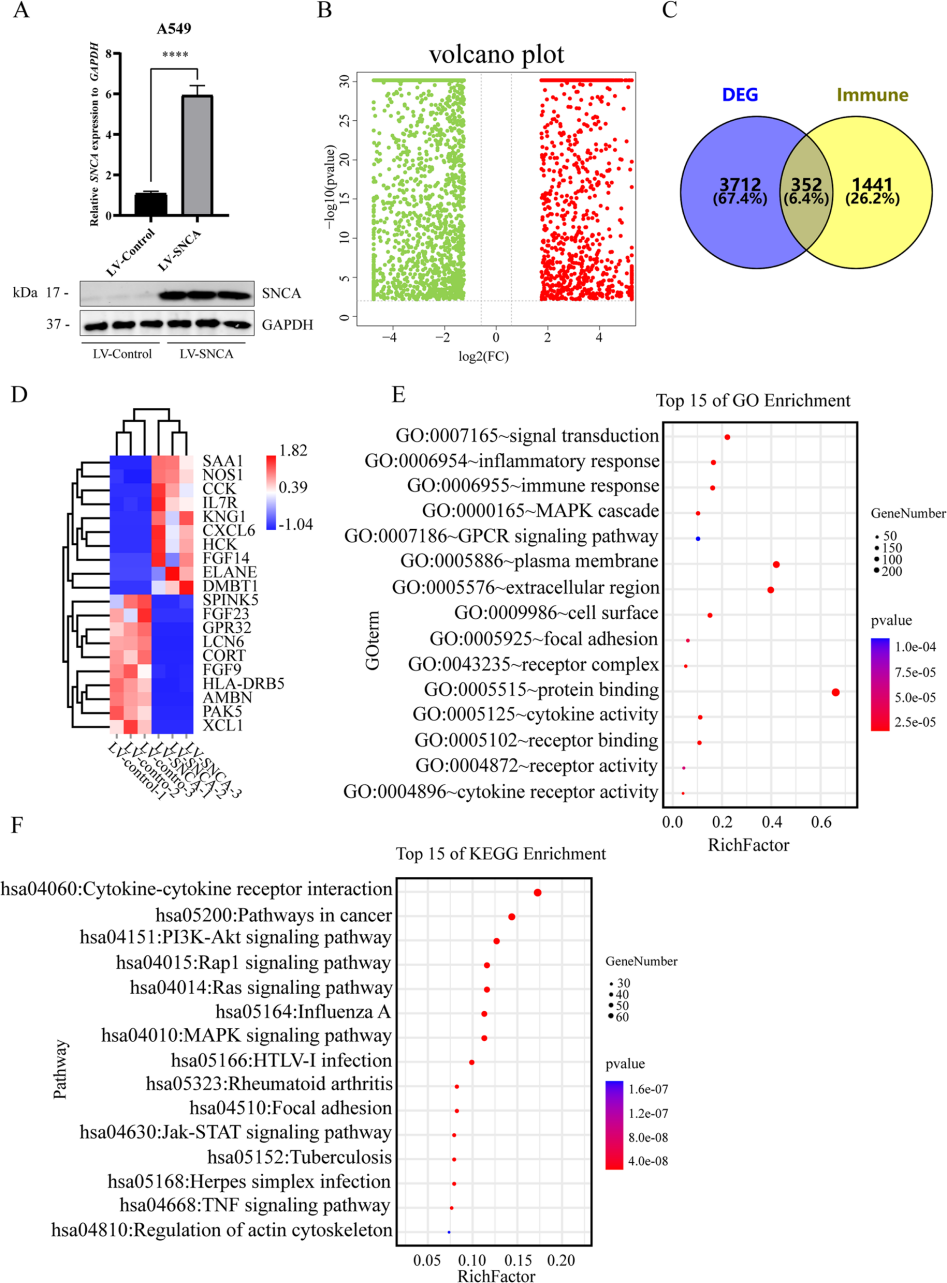
Genes related to immune regulation by *SNCA* in LUAD. **A** mRNA and protein levels of *SNCA* in the A549 cell line overexpressing *SNCA* were detected. **B** Volcanic map distribution of DEGs. Red dots are upregulated genes, and green dots are downregulated genes. **C** SNCA-regulated immune-related DEGs were obtained by Wayne analysis between the obtained DEGs and immune-related genes from Immport Resource. **D** Heat map showing the top 20 upregulated and top 20 downregulated immune-related DEGs. **E** GO analysis of immune-related DEGs, including five BP items, CC items, and MF items from top to bottom. **F** KEGG analysis of the top 15 immune-related DEGs (**** *P* < 0.0001)

**Fig. 6:**
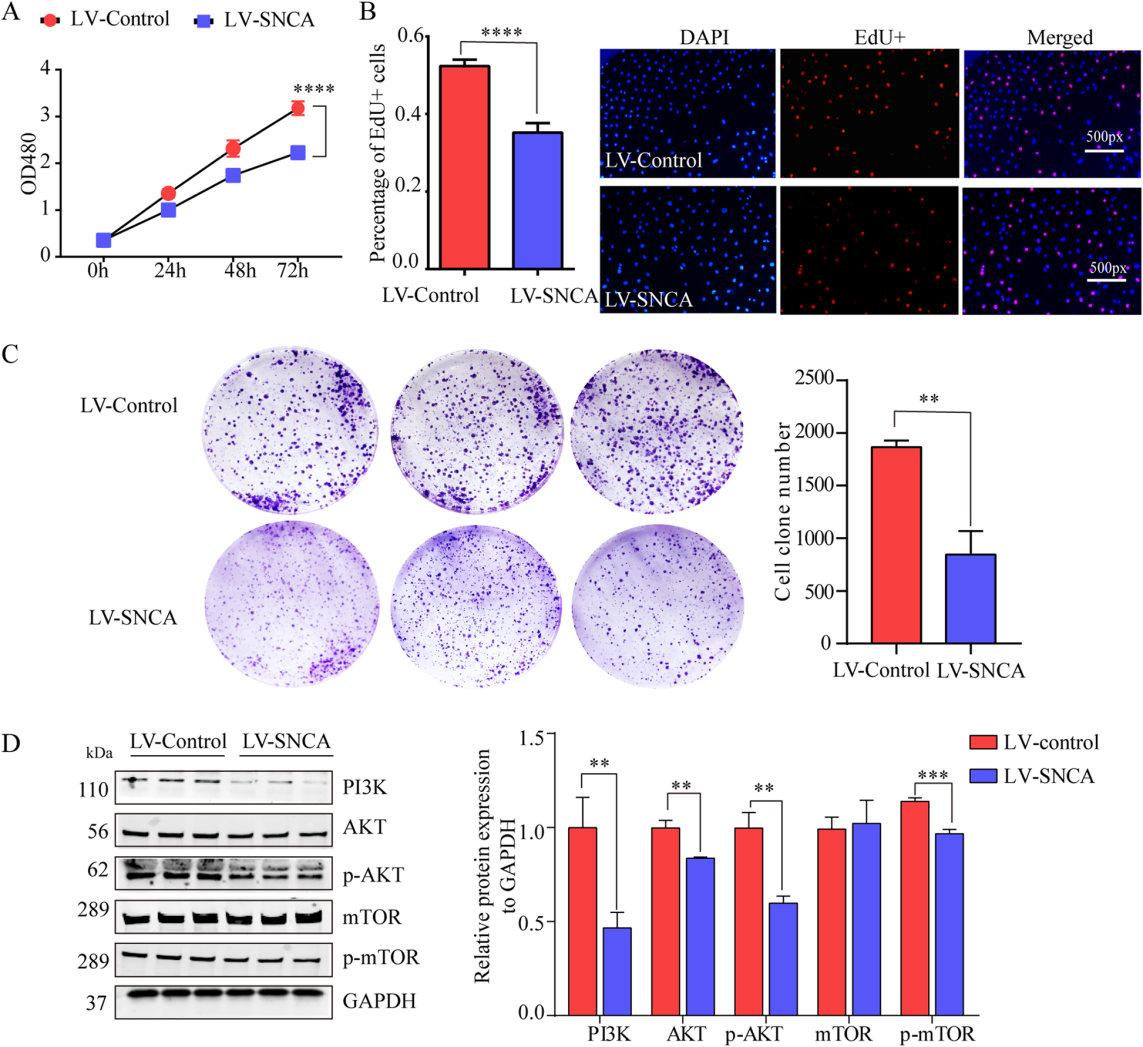
*SNCA* affects proliferation of LUAD cells in vitro via inhibiting PI3K/AKT/mTOR signaling pathway. **A** Proliferation of A549 cells overexpressing *SNCA* was assessed by CCK8; *** *P* < 0.001. **B** Effect of *SNCA* on A549 cell growth were evaluated by EdU assay (*n* = 5). Results of quantitative analysis were measured by Image J; **** *P* < 0.0001. **C** Effects of *SNCA* on A549 cell growth were further evaluated using a colony formation assay. Quantitative analysis of colony numbers is measured by Image J; ** *P* < 0.01. **D** Levels of PI3K, AKT, p-AKT, mTOR and p-mTOR proteins were assessed in stably-transfected A549 cells by Western blotting; ** *P* < 0.01, *** *P* < 0.001

**Fig. 7:**
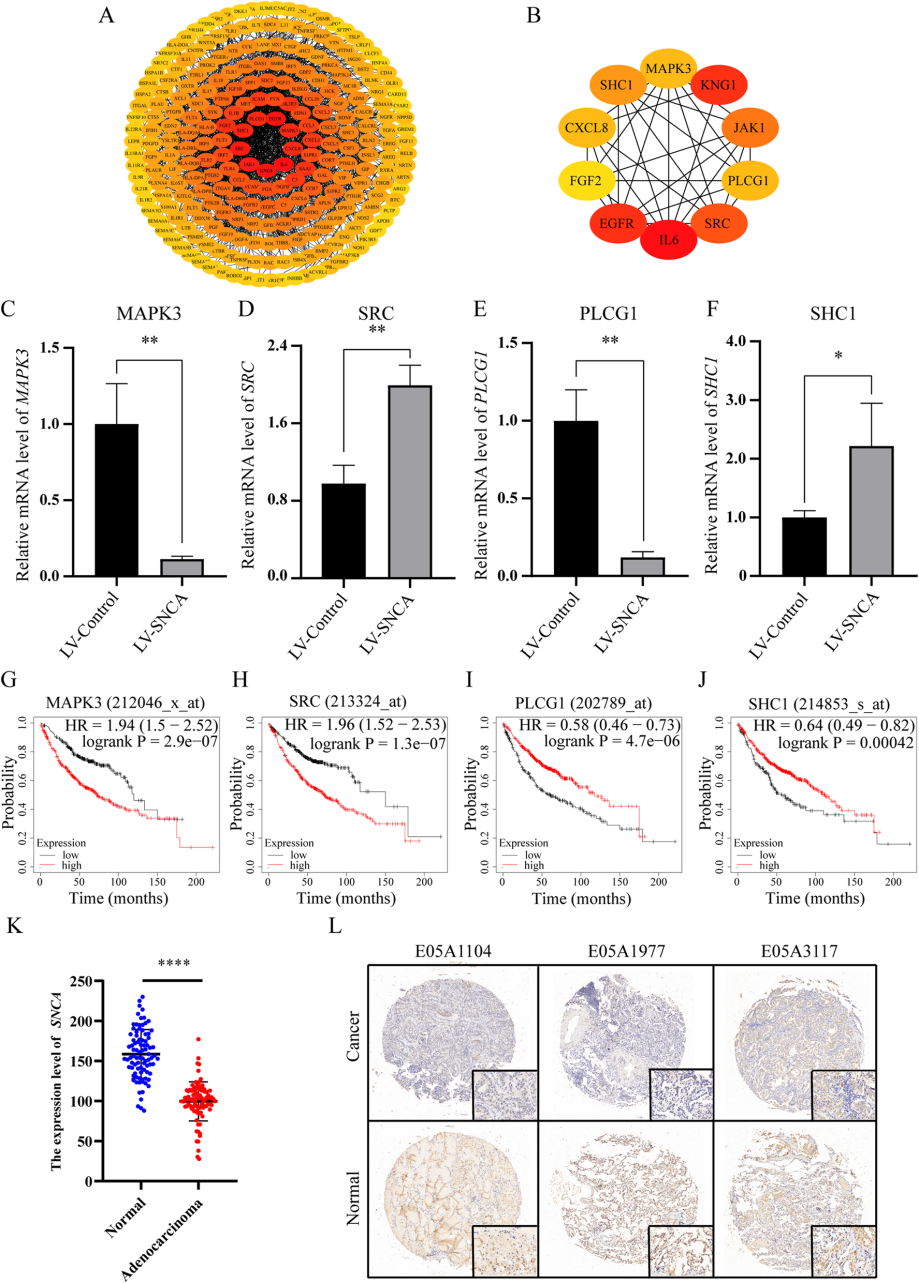
The screening of the key genes and analysis of *SNCA* in LUAD clinical samples. **A** Protein interaction network constructed by immune-related DEGs through Cytoscape. The orange node is the node with high degree, and the yellow node is the node with low degree. **B** The interaction network diagram of 10 node genes with the highest degree screened by Cytohubba application in Cytoscape. **C**–**F** qPCR method was used to verify the expression of four central genes in SNCA-overexpressing A549 cell line. **G**–**J** Kaplan–Meier curve was used to analyze the prognosis of four central genes in LUAD. **K** Statistical analysis of *SNCA* immunostaining score in clinical samples, ****P < 0.0001. **L** Immunohistochemical images of *SNCA* in clinical samples

**Fig. 8:**
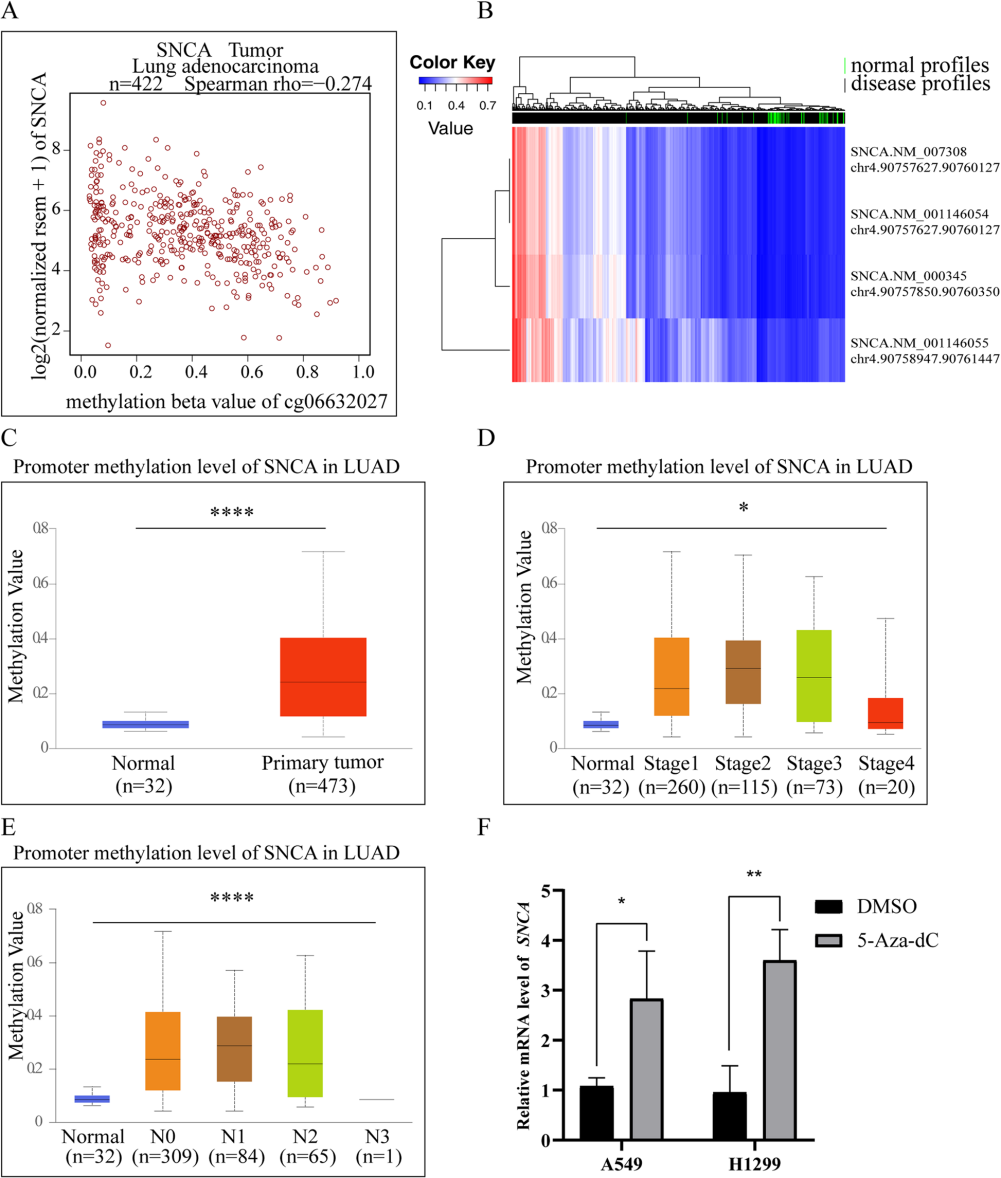
The analysis of *SNCA* promoter methylation level in LUAD. **A** The correlation analysis between the expression level of *SNCA* in LUAD samples and the methylation level of cg06632027 methylation site. **B** The analysis of methylation levels of four genomic fragments of *SNCA* promoter in tumor samples and normal samples in LUAD patient samples of the DiseaseMeth database. The expression level of *SNCA* methylation in LUAD patient samples downloaded from UALCAN database was analyzed with **C** sample type, **D** TNM stage, and **E** lymph node status. **F** After A549 and H1299 cells were treated with 5-aza-dC, the mRNA expression level of *SNCA* was detected by qPCR (* *P* < 0.05, ** *P* < 0.01, *** *P* < 0.001, **** *P* < 0.0001)
