# Supplementary material for: SNCA correlates with immune infiltration and serves as a prognostic biomarker in lung adenocarcinoma
Source: BMC Cancer. 2022 Apr 14;22:406. doi: 10.1186/s12885-022-09289-7 (PMC9009002; doi:10.1186/s12885-022-09289-7)
Supplement: Supplementary file 1 — Additional file 1. [file 12885_2022_9289_MOESM1_ESM.pdf]

	A	C	D	O	R	S	U	W
1		Target	Sample	Expression	Expression SEM	Corrected Expression SEM	Mean Cq	Cq SEM
2		MAPK3	LV-Control-1	3.19395	0.11508	0.11508	29.22	0.04402
3		MAPK3	LV-Control-2	2.04010	0.55920	0.55920	29.38	0.36010
4		MAPK3	LV-Control-3	2.09926	0.33007	0.33007	29.96	0.22397
5		MAPK3	LV-SNCA-1	0.26661	0.01540	0.01540	31.45	0.07657
6		MAPK3	LV-SNCA-2	0.23586	0.01364	0.01364	31.59	0.07686
7		MAPK3	LV-SNCA-3	0.32745	0.03344	0.03344	30.83	0.13992
8		SRC	LV-Control-1	0.50594	0.03292	0.03292	23.79	0.08971
9		SRC	LV-Control-2	0.46309	0.05425	0.05425	23.42	0.04307
10		SRC	LV-Control-3	0.45759	0.01645	0.01645	24.06	0.03734
11		SRC	LV-SNCA-1	0.83969	0.04854	0.04854	21.70	0.07665
12		SRC	LV-SNCA-2	0.83609	0.03508	0.03508	21.67	0.05111
13		SRC	LV-SNCA-3	1.00000	0.04858	0.04858	21.13	0.05272
14		PLCG1	LV-Control-1	1.80328	0.11406	0.11406	26.90	0.08992
15		PLCG1	LV-Control-2	1.84694	0.25021	0.25021	26.97	0.16627
16		PLCG1	LV-Control-3	1.26188	0.10627	0.10627	27.38	0.11395
17		PLCG1	LV-SNCA-1	0.16644	0.01315	0.01315	29.49	0.11327
18		PLCG1	LV-SNCA-2	0.15969	0.02068	0.02068	29.68	0.16607
19		PLCG1	LV-SNCA-3	0.26549	0.01057	0.01057	29.49	0.05549
20		SHC1	LV-Control-1	0.53635	0.02577	0.02577	26.91	0.06754
21		SHC1	LV-Control-2	0.57712	0.06509	0.06509	26.91	0.12620
22		SHC1	LV-Control-3	0.46017	0.02000	0.02000	27.09	0.04642
23		SHC1	LV-SNCA-1	0.86672	0.05851	0.05851	25.36	0.09659
24		SHC1	LV-SNCA-2	1.02513	0.06792	0.06792	25.25	0.04262
25		SHC1	LV-SNCA-3	1.59586	0.08607	0.08607	25.16	0.07639
26		SNCA	LV-Control-1	1.20478	0.02047	0.02047	21.78	0.00746
27		SNCA	LV-Control-2	1.00000	0.02633	0.02633	21.85	0.03483
28		SNCA	LV-Control-3	1.02396	0.05659	0.05659	21.86	0.03332
29		SNCA	LV-SNCA-1	6.45686	0.29204	0.29204	20.63	0.04407
30		SNCA	LV-SNCA-2	5.51194	0.18626	0.18626	21.12	0.01614
31		SNCA	LV-SNCA-3	5.81408	0.21293	0.21293	20.94	0.04173
32		SNCA	A549+5-aza-1	5.41359	0.24451	0.05746	22.33	0.05170
33		SNCA	A549+5-aza-2	8.30556	0.41767	0.41767	20.96	0.06349
34		SNCA	A549+5-aza-3	5.91100	0.26698	0.26698	22.94	0.05746
35		SNCA	A549+DMSO-1	0.89458	0.11649	0.11649	23.51	0.04166
36		SNCA	A549+DMSO-2	1.09188	0.00997	0.00997	22.80	0.00616
37		SNCA	A549+DMSO-3	1.00000	0.01664	0.01664	23.85	0.01319
38		SNCA	H1299+5-aza-1	3.53271	0.04809	0.04809	24.71	0.03883
39		SNCA	H1299+5-aza-2	2.79196	0.08348	0.08348	24.59	0.05992
40		SNCA	H1299+5-aza-3	3.78751	0.02430	0.02430	25.09	0.03140
41		SNCA	H1299+DMSO-1	0.41057	0.00682	0.00682	26.95	0.09981
42		SNCA	H1299+DMSO-2	1.11831	0.03904	0.03904	26.18	0.19669
43		SNCA	H1299+DMSO-3	1.47111	0.03680	0.03680	25.78	0.12552
44								
45								
46		SHC1-F	CCCGCTCAGCTCTATCCTG					
47		SHC1-R	GGCAACATAGGCGACATACTC					
48		PLCG1-F	GGAAGACCTCACGGGACTTTG					
49		PLCG1-R	GCGTTTTCAGGCGAAATTCCA					
50		MAPK3-F	CTACACGCAGTTGCAGTACAT					
51		MAPK3-R	CAGCAGGATCTGGATCTCCC					
52		SRC-F	GAGCGGCTCCAGATTGTCAA					
53		SRC-R	CTGGGGATGTAGCCTGTCTGT					
54		GAPDH-F	GGAGCGAGATCCCTCCAAAAT					

	A	C	D	O	R	S	U	W
55		GAPDH-R	GGCTGTTGTCATACTTCTCATGG					
56		SNCA-F	AAGAGGGTGTTCTCTATGTAGGC					
57		SNCA-R	GCTCCTCCAACATTTGTCACTT					
