# Supplementary material for: SNCA correlates with immune infiltration and serves as a prognostic biomarker in lung adenocarcinoma
Source: BMC Cancer. 2022 Apr 14;22:406. doi: 10.1186/s12885-022-09289-7 (PMC9009002; doi:10.1186/s12885-022-09289-7)
Supplement: Supplementary file 2 — Additional file 2. [file 12885_2022_9289_MOESM2_ESM.docx]

| **Primary antibodies** | **Type** | **Souse** |
| --- | --- | --- |
| Anti-SNCA | Rabbit monoclonal (ab13850) | abcam |
| Anti-GAPDH | Mouse monoclonal (60004-1-Ig) | Proteintech |
| Anti-PI3K | Rabbit polyclonal (20548-1-AP) | Proteintech |
| Anti-AKT | Rabbit polyclonal (10176-2-AP) | Proteintech |
| Anti-p-AKT | Mouse monoclonal (66444-1-Ig) | Proteintech |
| Anti-mTOR | Mouse monoclonal (66888-1-Ig) | Proteintech |
| Anti-p-mTOR | Mouse monoclonal (67778-1-Ig) | Proteintech |
| IRDye-conjugated secondary antibodies | goat anti-mouse | Odyssey |
| IRDye-conjugated secondary antibodies | goat anti-rabbit | Odyssey |
| secondary antibodies | goat anti-rabbit | Abcam |

**Supplementary Table 1 The antibodies associated with this research.**
