# Supplementary figures and images for: SNCA correlates with immune infiltration and serves as a prognostic biomarker in lung adenocarcinoma
Source: BMC Cancer. 2022 Apr 14;22:406. doi: 10.1186/s12885-022-09289-7 (PMC9009002; doi:10.1186/s12885-022-09289-7)

# LUAD (GSE31210)

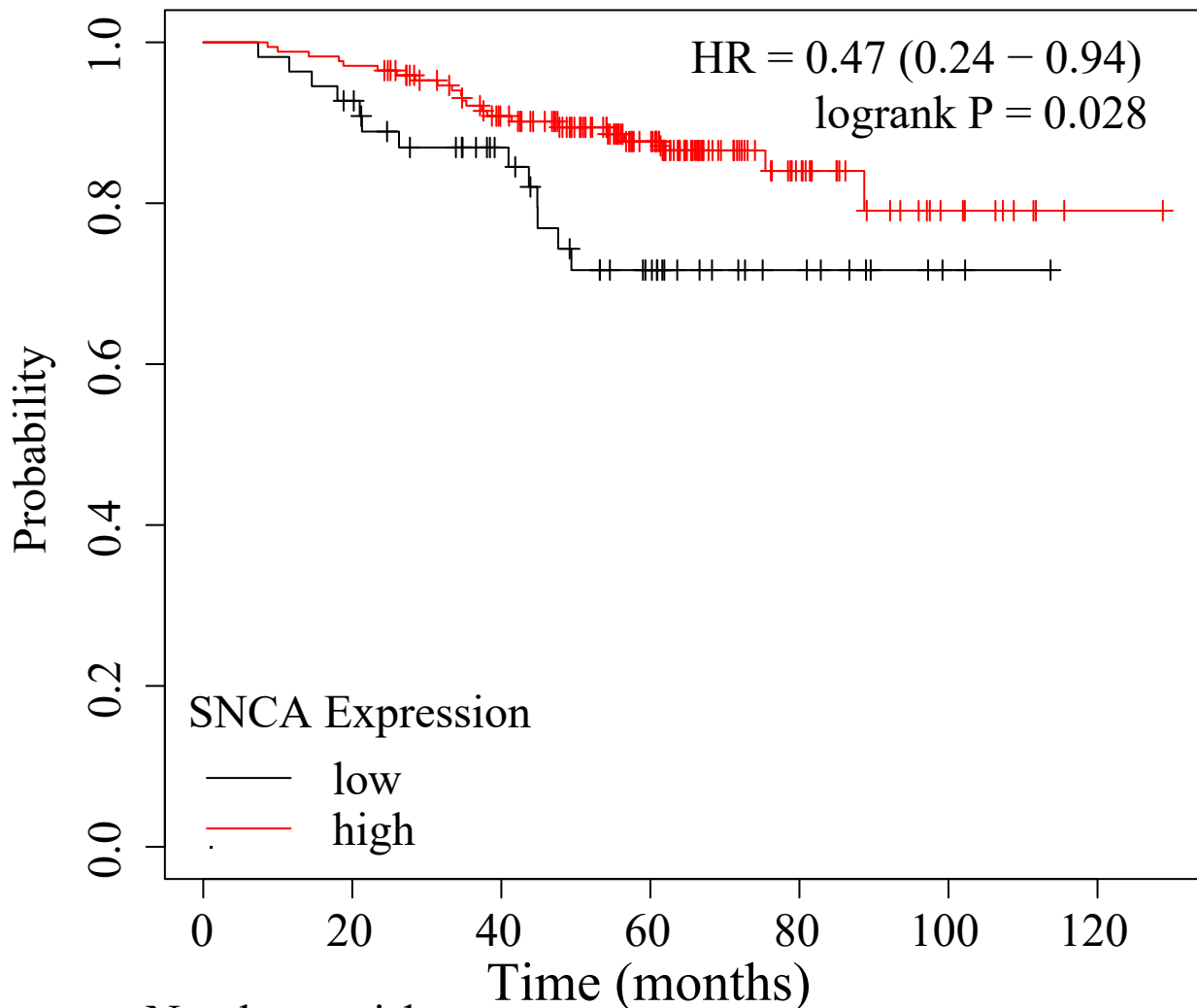

Number at risk

|      |     |     |     |    |    |   |   |
|------|-----|-----|-----|----|----|---|---|
| low  | 55  | 50  | 36  | 22 | 9  | 2 | 0 |
| high | 171 | 166 | 137 | 88 | 27 | 9 | 1 |

Supplement: Supplementary file 3 — Additional file 3. [file 12885_2022_9289_MOESM3_ESM.pdf]

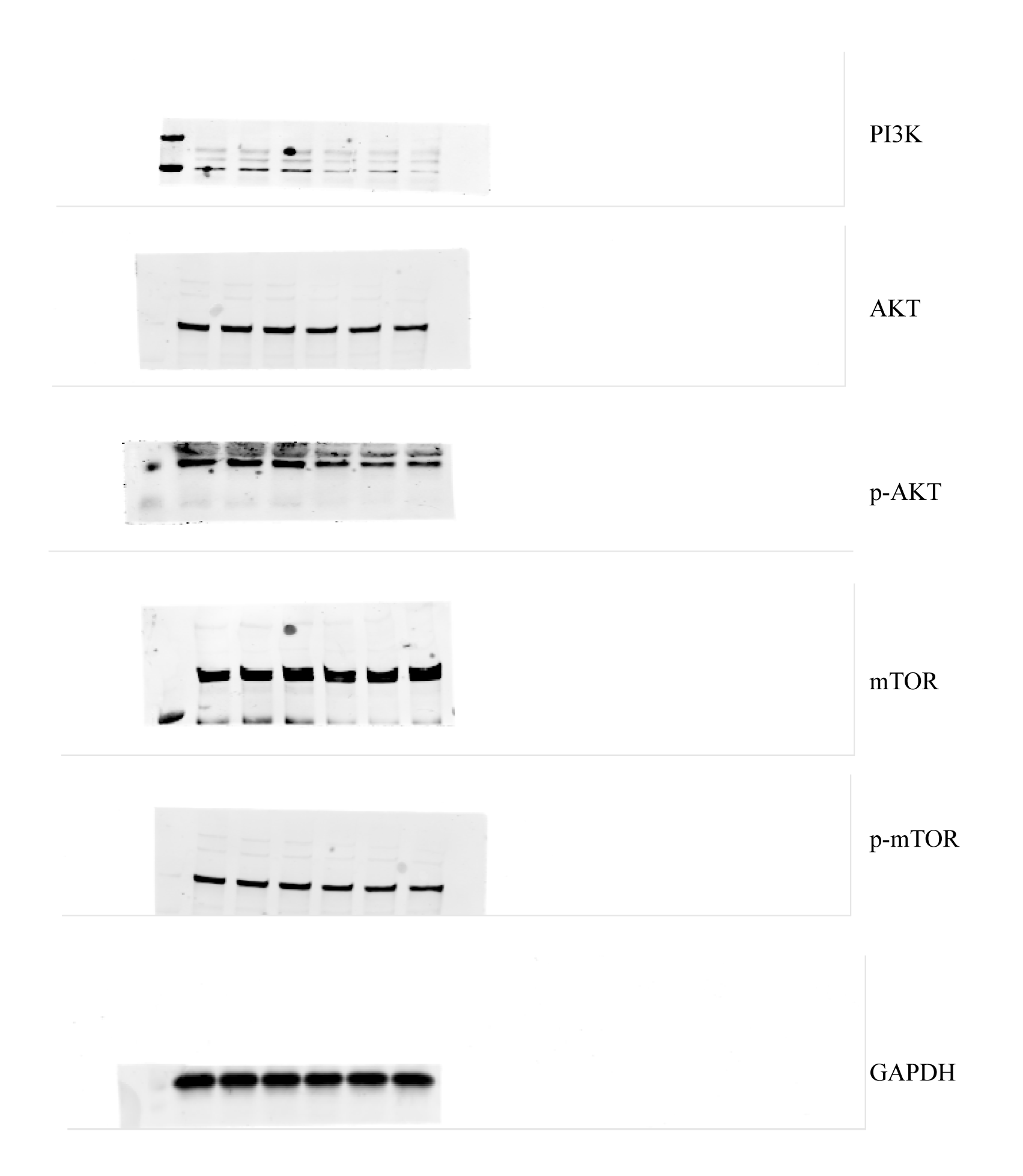

Supplement: Supplementary file 8 — Additional file 8. [file 12885_2022_9289_MOESM8_ESM.tif]
