# Supplementary material for: SNCA correlates with immune infiltration and serves as a prognostic biomarker in lung adenocarcinoma
Source: BMC Cancer. 2022 Apr 14;22:406. doi: 10.1186/s12885-022-09289-7 (PMC9009002; doi:10.1186/s12885-022-09289-7)
Supplement: Supplementary file 4 — Additional file 4. [file 12885_2022_9289_MOESM4_ESM.pdf]

GSE68571			GSE104797			GSE10072		
Gene markers	Cor	*P*	Gene markers	Cor	*P*	Gene markers	Cor	*P*
FPR1	0.525095	2.10E-07	MS4A4A	0.98159	8.75E-05	CD84	0.644238	4.86E-08
CD8B	0.4219	5.21E-05	MS4A1	-0.92204	0.003124	CCR8	0.383993	0.002923
CD68	0.419207	5.89E-05	FOXP3	-0.85388	0.014467	NCR1	0.366549	0.004655
CCR8	0.356205	0.000762	CD84	0.846228	0.016364	FOXP3	0.363084	0.00509
C3AR1	-0.25825	0.016362	CD209	-0.7821	0.037705	CD8A	0.265505	0.043976
			CD8B	0.769801	0.042948			

Supplementary Table3. Correlation analysis between *SNCA* expression and immune cell markers in LUAD data sets of GSE68571, GSE104797, and GSE10072. P values less than 0.05 were considered statistically significant.
