# Supplementary material for: SNCA correlates with immune infiltration and serves as a prognostic biomarker in lung adenocarcinoma
Source: BMC Cancer. 2022 Apr 14;22:406. doi: 10.1186/s12885-022-09289-7 (PMC9009002; doi:10.1186/s12885-022-09289-7)
Supplement: Supplementary file 5 — Additional file 5. [file 12885_2022_9289_MOESM5_ESM.pdf]

DEGs			Immune-Related DEGs		
Gene symbol	Log FC	P value	Gene symbol	Log FC	P value
TFF1	11.010761	5.52E-07	SAA1	10.437405	7.69E-06
UTP14C	10.842874	4.3E-17	CCK	9.137845	0.0002079
SAA1	10.437405	7.69E-06	CXCL6	8.6677029	0.0000242
SNURF	10.34799	0.0000155	ELANE	8.0945176	0.0047151
C4BPB	9.6073303	0.0000039	HCK	8.0223678	0.0000713
C2orf70	9.5761694	0.0000639	KNG1	6.7813597	0.0087942
GPRC5D	9.4851584	0.000031	FGF14	6.3808218	0.0078883
CCK	9.137845	0.0002079	IL7R	6.0660892	0.0016881
AC073111.3	9.0223678	0.0017649	NOS1	5.1898246	2.34E-09
TSPAN8	8.9267902	0.0000726	DMBT1	5.0588937	0.0094059
CST1	8.8454901	0.0011437	NOX1	5.0074945	5.68E-06
LCE3D	8.7256503	0.0056032	IL1B	4.8454901	0.0012354
CXCL6	8.6677029	0.0000242	SERPINA3	4.7470157	9.13E-105
TM4SF5	8.6438562	0.0034791	R3HDML	4.5698556	7.38E-11
ZNF726	8.5824556	0.0000284	C5AR2	4.523562	0.0040493
ADIRF	8.5443205	0.0013504	TGFB2	4.3929437	0
AC011604.2	8.464886	4.68E-06	PTGS2	4.3735924	0
ARL14EPL	8.4093909	0.0016509	EDN1	4.3120474	1.29E-150
TMEM30B	8.3219281	2.26E-08	CXCL3	4.2927009	1.07E-46
LRRC31	8.1963972	9.77E-06	OSMR	4.2866687	0
ELANE	8.0945176	0.0047151	SFTPD	4.2479275	0.0054567
KRT34	8.0223678	0.0002698	EREG	4.2443405	0
HCK	8.0223678	0.0000713	CXCL8	4.1868034	3.2E-199
WDR86	8.0037521	0.0010283	IL31RA	4.169925	8.52E-11
CRISP3	7.8454901	0.0017774	AREG	4.1406009	1.24E-240
SLC6A14	7.5443205	2.19E-06	BTC	4.0859989	1.29E-54
ADAM29	7.3219281	0.0016	CXCL1	4.0836515	1.41E-186
CHL1	7.2605276	0.0059048	DKK1	4.0691029	1.39E-298
CTNNA2	7.1632303	0.0084261	ANGPTL4	4.065623	2.56E-73
HIST1H3G	7.0223678	0.0026187	FGF12	4.0511043	3.66E-19
FRG2	6.8662486	0.0079696	CRABP1	4.0492435	0.0056523
KNG1	6.7813597	0.0087942	TFR2	4.0395284	1.31E-06
HRASLS5	6.6911619	0.0059779	CCL2	4.0348127	1.96E-74
IYD	6.6911619	0.0058595	VCAM1	4.0297473	0.0024127
KCNE1B	6.5443205	0.0039647	IL6	3.994483	1.7E-22
FGF14	6.3808218	0.0078883	OAS1	3.8816971	1.16E-53
PCDHA6	6.3219281	0.004413	S100A3	3.8458769	1.37E-28
SH3TC2	6.2605276	0.0053089	CHGB	3.8344232	2.21E-58
MMP7	6.219689	1.63E-65	OLR1	3.8189429	9.81E-22
KCNV1	6.1963972	0.0006626	INSL4	3.8107113	1.88E-90
IL7R	6.0660892	0.0016881	MARCO	3.8073549	0.0017129
SRD5A2	6.0588937	0.0027528	PSMB8	3.798773	3.39E-58
RP1	6.0588937	0.0016231	SLPI	3.7738024	6.39E-36
PCDHB4	5.9848931	0.0059048	HFE	3.7264413	3.12E-112
LY75	5.9848931	0.0003184	PCSK1	3.725825	4.39E-11
ADAMTS12	5.9541963	5.57E-06	BDNF	3.7161414	4.96E-147
C2CD6	5.9068906	0.009808	TLR2	3.7142455	0.0001775
TCN1	5.9008668	5.14E-06	CSRP1	3.6808938	0
VNN1	5.7279205	1.73E-06	ICAM1	3.6568998	8.4E-86
ANKS1B	5.6438562	0.000008	IL1A	3.6466016	1.82E-08
PTPRN	5.523562	0.0066662	RBP4	3.6295459	1.63E-13
RARRES1	5.4594316	1.63E-53	SPP1	3.6272624	5.97E-301
FAM153B	5.3309169	0.0000609	IL18	3.6109239	0
BCL2A1	5.2687235	2.49E-20	NRG1	3.597769	0
NOS1	5.1898246	2.34E-09	IFIH1	3.5849625	4.39E-26
TP53TG3F	5.1223966	7.08E-06	GBP2	3.5745351	1.8E-49
ARMC3	5.1085245	0.0017666	IL15	3.5739914	1.92E-75
AL355102.2	5.1085245	2.05E-06	IL11	3.5479208	9.85E-141
PQLC2L	5.0611115	9.28E-07	GLP2R	3.5347997	3.39E-93
DMBT1	5.0588937	0.0094059	CCL20	3.4924359	4.54E-07
NOX1	5.0074945	5.68E-06	C5	3.4798177	0
ADTRP	5	0.004929	RAET1E	3.4749613	5.49E-11
IRF4	5	0.0006477	CXCL2	3.4617801	2.41E-42
HTR1F	4.9395792	2.51E-19	PDGFB	3.4364772	2.12E-123
PCDHA2	4.9307373	0.0008252	VIP	3.4329594	0.0022533
C5orf38	4.9205655	0.0005861	SHC3	3.4299878	1.43E-06

SNCA	4.9188113	0
FLG2	4.9068906	0.0028124
RELN	4.9068906	0.0018014
DIO2	4.8899175	3.75E-83
RARRES3	4.8853574	0.000288
HRH2	4.8662486	1.16E-07
EIF1AY	4.8644553	7.6E-30
IL1B	4.8454901	0.0012354
KRTAP5-2	4.8356995	4.47E-08
CSMD3	4.8073549	4.54E-06
CD38	4.8008088	1.33E-103
PCDHB6	4.7999754	6.26E-23
CEACAM5	4.7900769	0.0002286
SERPINA3	4.7470157	9.13E-105
RIN1	4.6853644	4.27E-27
EID3	4.6147098	1.53E-12
EVA1C	4.6084874	5.16E-70
OLAH	4.6073303	0.000109
SERPINB7	4.606508	1.36E-20
C6orf58	4.5969351	0.0015076
ZFP82	4.5944228	2.28E-14
PRR18	4.5849625	0.0036205
R3HDML	4.5698556	7.38E-11
NPHS1	4.5599956	3.87E-42
SLCO2B1	4.5554182	1.19E-20
PRODH2	4.5545889	0.0012996
CYP7B1	4.5443205	1.15E-07
XDH	4.5413732	7.18E-13
CES1	4.5338716	9.16E-51
C5AR2	4.523562	0.0040493
CT62	4.5145732	0.0000578
MYOCD	4.5029937	4.26E-82
C15orf48	4.4988298	6.21E-94
LRMDA	4.4956952	1.88E-19
PCSK9	4.4918531	3.17E-07
CEACAM6	4.4649627	7.15E-51
CLDN11	4.4594316	0.0016372
PPP1R14D	4.4521268	0.0012289
GPRC5A	4.4253364	0
SOHLH2	4.4150375	0.0031518
AOX1	4.4115653	4.01E-43
ST8SIA1	4.3985494	2.49E-06
TGFB2	4.3929437	0
SFRP1	4.3923174	1.01E-186
RNF212	4.3846639	7.71E-07
SLC35F3	4.3822476	3.02E-66
PTGS2	4.3735924	0
MUC12	4.3504972	7.84E-22
SDR42E1	4.3479233	1.02E-28
RHBDL2	4.3462478	3.79E-23
ZG16B	4.3219281	0.0000118
EDN1	4.3120474	1.29E-150
BIRC3	4.2976805	5.06E-230
CXCL3	4.2927009	1.07E-46
CYP24A1	4.2906167	0
OSMR	4.2866687	0
CDCP1	4.2859524	0
CYP4F11	4.2809113	9.36E-47
GGT5	4.2779847	0.0008541
44259	4.2735476	1.91E-55
ZNF558	4.2697419	2.46E-93
NPIPA8	4.2587343	0.0001893
SFTPD	4.2479275	0.0054567
SDR16C5	4.2479275	0.0001668
EREG	4.2443405	0
TPRG1	4.2302976	8.23E-09
RHCG	4.2263272	8.94E-76
COL21A1	4.2254201	1.15E-17
C3	3.4287112	0
TNFSF15	3.4201516	1.72E-36
VTN	3.4147856	1.7E-38
PTPN6	3.3906863	2.77E-79
PSMD5	3.3817607	2.31E-273
S100A6	3.3815937	0
PLAU	3.375287	0
TNFRSF10A	3.3343592	1.34E-126
NPR3	3.3085599	1.65E-133
LTBR	3.2840628	0
CTSS	3.2707938	6.7E-170
ADM	3.255979	1.87E-225
EDN2	3.239705	1.83E-31
IL33	3.2394659	0.0008242
INSL3	3.2326608	0.000705
TAPBPL	3.216414	2.27E-19
CXCL5	3.2093928	0
S100A16	3.208815	1.08E-228
GREM1	3.2034343	2.68E-147
CD14	3.2021238	5.88E-12
FGA	3.1965392	0
ISG20	3.1900747	1.02E-33
TNFRSF12A	3.1633998	1.83E-218
THBS1	3.1591986	0
IRF5	3.1361367	1.3E-38
CCN2	3.1169717	1.24E-252
EGFR	3.0943557	0
NR0B1	3.0914298	5.77E-36
NR2E3	3.0874628	0.0010014
NR1H4	3.071661	2.72E-07
HNF4A	3.0654054	5.83E-172
S100P	3.0649028	7.24E-17
TLR3	3.0641303	1.95E-06
CCL5	3.0622843	6.04E-07
TMSB4X	3.0502601	0
IL20RB	3.0432278	9.87E-51
TNFSF10	3.0381351	9.14E-09
PLXNA4	3.0356239	0.0000795
TNFRSF9	3.0232074	9.82E-54
UCN2	2.9945952	3.23E-30
PDGFD	2.984078	7.16E-72
STC1	2.9789301	2.96E-145
NRP2	2.9612729	0
TLR4	2.959358	0.000038
SCG2	2.9581159	5.81E-19
TLR1	2.9574023	3.85E-27
EBI3	2.9562674	1.21E-25
VEGFD	2.9385995	0.0071338
NFATC2	2.9293454	2.15E-19
LCN2	2.9091876	9.24E-145
SEMA3C	2.8899232	0
BCL3	2.8638147	4.34E-165
SEMA4B	2.849904	0
NRP1	2.8456458	0
TPM2	2.8315745	0
NTS	2.821388	1.3E-51
IL22RA1	2.8124982	3.44E-10
IL1R1	2.8017463	1.28E-122
OSGIN1	2.7792764	2.8E-85
LTB	2.751665	8.3E-07
LIF	2.7484564	3.04E-301
JAG1	2.6973577	0
CLCF1	2.6815898	1.53E-35
RELB	2.6814377	6.34E-109
PTHLH	2.639359	2.39E-33
IL32	2.6345945	8.32E-103
CCR7	2.6182387	2.78E-08
SOCS3	2.6176511	4.24E-52

UNC5D	4.223563	3.06E-38	CDH1	2.6015664	0
MYEOV	4.2228878	9.48E-106	TGFBR2	2.5868877	0
THEG	4.2223924	0.0000347	APOH	2.5795793	1.6E-23
GLIPR1	4.220397	1.79E-234	INHBB	2.5696382	8.54E-78
KMO	4.2191685	1.71E-07	OXTR	2.5360529	6.19E-40
ZMAT4	4.2146429	1.17E-31	MX1	2.5092736	7.6E-14
APOL1	4.2029401	7.72E-44	PLAUR	2.4636403	6.85E-100
DAW1	4.1996723	0.000202	NGF	2.4594316	0.0003142
PRDM1	4.1982696	4.38E-39	RAET1G	2.4109331	1.7E-13
RBFOX3	4.1963274	2.58E-152	CRIM1	2.4104593	0
CXCL8	4.1868034	3.2E-199	NFKBIZ	2.3776326	4E-79
SNORC	4.1722463	8.7E-09	SEMA3A	2.3453158	2.24E-132
IL31RA	4.169925	8.52E-11	IL6ST	2.3099555	0
SERPINB8	4.1494245	8.56E-59	MAP3K8	2.3080392	2.52E-81
DDX60L	4.1469263	7.44E-120	CSF2RA	2.2825794	6.68E-32
NBPF4	4.1456775	0.0000011	AHNAK	2.2770275	0
THY1	4.1416455	1.44E-29	MC1R	2.269401	1.12E-22
SUSD2	4.1413073	0	TGFA	2.2509873	2.65E-70
AREG	4.1406009	1.24E-240	GIP	2.2451125	0.0062896
RNF103-CHMP3	4.1404812	0.0005654	TYMP	2.2091963	5.53E-24
ADAMTS16	4.138414	2.64E-19	ACKR3	2.2083991	5.5E-19
PDZK1IP1	4.1361233	3.83E-91	HSPA1A	2.2032444	8.38E-83
FAM24B	4.1348094	2.64E-22	LTBP2	2.1946474	4.97E-216
CFTR	4.129283	0.0013323	CHP1	2.158001	0
UGT1A1	4.1219905	1.07E-11	IFNE	2.1497471	0.0006806
NPY2R	4.1210154	0.000035	RXRA	2.1323171	5.89E-126
PSMB9	4.1172102	4.09E-20	NR5A2	2.1312909	5.05E-83
CYP4F12	4.1162071	3.06E-17	PROC	2.1189411	0.0024748
ABCA8	4.1043367	3.62E-19	HSPA2	2.1055919	2.34E-183
TDO2	4.101538	0.000241	MUC5AC	2.0960018	6.95E-49
MEIOB	4.0951572	6.14E-47	IGF1R	2.0933031	0
KRT81	4.0937644	0	BMP1	2.0900984	6.56E-245
LHFPL4	4.0935119	2.33E-16	IL1R2	2.0759489	0.0064802
HTR1B	4.0874628	0.0069606	MET	2.0704462	0
CADM3	4.0874628	0.0015488	ERAP2	1.974909	1.11E-06
MB	4.0874628	1.11E-27	ITGAL	1.9696264	0.0041602
BTC	4.0859989	1.29E-54	GDF15	1.9686895	2E-173
CXCL1	4.0836515	1.41E-186	MAP3K14	1.9534024	3.86E-81
STRA6	4.0772325	0	S100A2	1.9350825	0.0001368
DNER	4.0759933	1.25E-36	IKBKE	1.9311175	5.13E-32
DKK1	4.0691029	1.39E-298	DDX58	1.9211446	5.64E-40
CTAGE4	4.0660892	0.002016	SRC	1.9113771	4.98E-277
ANGPTL4	4.065623	2.56E-73	JAK1	1.8784557	0
GDA	4.0633951	9.84E-30	NDRG1	1.860179	1.81E-73
SBK3	4.0600474	9.18E-08	ITGAV	1.8575169	0
SLFN13	4.0541751	1.35E-37	NR1H2	1.854213	4.72E-116
FGF12	4.0511043	3.66E-19	CTSB	1.8497839	0
UPK1B	4.0503822	2.25E-14	S100A11	1.8437651	0
ATP10A	4.0495283	3.11E-111	BMP2	1.8398428	1.17E-13
CRABP1	4.0492435	0.0056523	PRKCA	1.8278736	0
FAM218A	4.0443941	0.0005644	IRF9	1.8143108	1.14E-42
CDH4	4.0427567	1.01E-201	TNFRSF1A	1.806068	4.41E-249
C4BPA	4.0408451	1.33E-17	F2RL1	1.7871416	4.73E-148
TFR2	4.0395284	1.31E-06	TMSB10	1.7711015	7.74E-47
CCL2	4.0348127	1.96E-74	NEDD4	1.7669355	2.83E-245
NALCN	4.0300572	2.14E-64	IKBKG	1.7317723	3.55E-47
VCAM1	4.0297473	0.0024127	BMPR1B	1.731456	1.81E-79
ASB4	4.0297473	5.68E-06	TNFSF12	1.7252124	1.57E-15
CP	4.0285402	1.08E-249	CARD11	1.7243656	0.0001171
DRD1	4.0129659	1.28E-73	HSPA1L	1.7232907	4.02E-07
ZFP3	4	5.9E-22	ERAP1	1.7092396	4.83E-123
PHLDA1	3.9976309	0	TNFAIP3	1.6884604	1.1E-95
IL6	3.994483	1.7E-22	FGF2	1.6842828	8.21E-191
CAVIN2	3.9931136	4.71E-64	PDGFA	1.6444601	1.05E-62
SNRPN	3.9928954	0	VEGFC	1.6435373	8.47E-32
IRX2	3.9899463	7.1E-07	PTK2B	1.6346344	1.13E-55
SLITRK6	3.987244	3.57E-25	RASGRP3	1.6297504	5.54E-07
SRGN	3.9832105	1.89E-264	SDC1	1.6122668	5.65E-263

CRIP1	3.9780384	1.47E-172	PDGFRL	1.6073355	9.88E-50
TCIM	3.9354808	0	IRF7	1.5849625	2.96E-10
NPBWR1	3.9315255	7E-60	SHC1	1.5619062	9.19E-249
CPNE4	3.9271494	1.08E-59	CSF1	1.5436788	5.74E-62
KIRREL3	3.9234192	1.59E-50	PSME2	1.52896	1.99E-69
TNFAIP2	3.9157114	0	ITGB2	1.525091	0.0026924
XKR9	3.9142701	0.0000383	AKT3	1.5206383	4.79E-88
TP53TG3D	3.9132884	3.87E-06	IL13RA1	1.5193927	1.56E-232
C6orf141	3.9099407	1.85E-18	SDC4	1.51139	4.17E-129
MPV17L	3.909636	3.17E-39	PLXNC1	-1.527247	2.46E-06
FGG	3.9015314	5.01E-266	SEMA3E	-1.53981	0.000101
PCDHAC1	3.8883851	4.21E-49	IFNLR1	-1.540444	3.1E-15
OAS1	3.8816971	1.16E-53	KITLG	-1.568432	7.91E-28
CAVIN3	3.8797058	6.62E-06	MAPK3	-1.616593	7.84E-66
HORMAD1	3.8790308	4.03E-100	ULBP2	-1.618498	2.65E-10
IFI44	3.8786937	1.6E-10	RBP1	-1.639885	3.66E-06
PARP12	3.8617449	1.6E-81	PLCG1	-1.649905	3.09E-98
PCDHA7	3.857981	0.0041016	CALCRL	-1.720846	5.16E-10
SLC4A10	3.857981	0.0022702	JAG2	-1.741964	2.88E-71
GPR20	3.857981	0.0001231	PIK3R3	-1.748098	2.18E-06
UBD	3.857981	0.0000543	LEPR	-1.811822	3.95E-31
S100A3	3.8458769	1.37E-28	WNT5A	-1.818926	9.1E-34
DECR1	3.8379755	2.77E-171	ENG	-1.853779	0.0004933
CHGB	3.8344232	2.21E-58	IL11RA	-1.85947	1.8E-10
STAT5A	3.8340948	5.34E-28	HLA-B	-1.982471	2.05E-94
ANXA10	3.83289	1.37E-07	IFI30	-1.990734	1.35E-36
NNMT	3.825647	8.62E-286	FGF19	-2.005538	0.0044979
CAPN12	3.8212425	1.28E-18	FYN	-2.012744	7.8E-80
OLR1	3.8189429	9.81E-22	TMSB15B	-2.019744	3.48E-07
TGFBI	3.8124935	0	APLN	-2.020272	0.0004355
INSL4	3.8107113	1.88E-90	HDAC1	-2.070942	6.31E-211
P4HTM	3.810226	4.44E-67	SEMA6C	-2.08254	2.53E-21
MARCO	3.8073549	0.0017129	GDNF	-2.106199	0.0015036
PSMB8	3.798773	3.39E-58	BIRC5	-2.107185	7.42E-97
PON1	3.7973477	1.06E-14	MAPT	-2.115289	2.85E-28
KRT7	3.7948605	0	S1PR2	-2.13774	1.15E-18
PON3	3.7905812	1.57E-169	SHC2	-2.139024	1.7E-17
ZBED2	3.7842713	0.000098	SLIT1	-2.193681	1.62E-12
SEC14L4	3.7813597	1.33E-24	KCNH2	-2.195148	6.45E-10
SLC22A18AS	3.7789731	0.0000648	FGF11	-2.199203	1.57E-22
IGFBP3	3.7781218	0	ARG2	-2.329812	1.65E-48
CGREF1	3.7767801	2.57E-28	TOR2A	-2.394628	1.87E-67
SLPI	3.7738024	6.39E-36	CALCB	-2.39837	1.97E-24
SERPINE1	3.7724514	0	FGF5	-2.429988	0.0012644
SQOR	3.7710876	2.02E-209	PGF	-2.434072	2.63E-13
SCNN1B	3.7655347	0.0028363	NR3C2	-2.44859	6.49E-16
NKAIN4	3.7641988	3.29E-33	ARTN	-2.471306	0.00089
GRIN2B	3.7625007	5.48E-58	ACKR4	-2.473931	0.0090272
XYLT1	3.7592134	4.99E-62	ACVR2B	-2.491088	1.8E-158
NDN	3.7548875	0.0016995	CLEC11A	-2.513095	1.24E-17
LINC00672	3.7548875	8.97E-10	BACH2	-2.527932	8.44E-22
BDKRB2	3.7548875	1.6E-223	GDF11	-2.532334	3.45E-43
SERPINA5	3.7489382	0.0005287	ROBO3	-2.545342	2E-24
IGSF11	3.7452451	2.76E-71	HSPA1B	-2.548589	9.93E-173
FILIP1	3.7440366	4.29E-120	RLN2	-2.596676	4.33E-06
SLC35D2	3.730834	3.49E-85	PLTP	-2.60395	1.25E-68
GALNT14	3.7280069	4.05E-118	NRTN	-2.66446	2.88E-09
HFE	3.7264413	3.12E-112	S1PR1	-2.693487	0.0006455
PCSK1	3.725825	4.39E-11	OPRD1	-2.70044	4.56E-07
PRRT1B	3.722466	5.86E-09	PRKCB	-2.706034	1.31E-26
MILR1	3.7207216	2.11E-34	IL9R	-2.721425	1.11E-07
AKR1E2	3.7188182	1.65E-17	SEMA4A	-2.757697	2.21E-10
CFH	3.7167654	0	PTGER2	-2.778644	9.08E-08
PCDHA4	3.7166682	1.34E-49	CYSLTR1	-2.79518	0.0027587
BDNF	3.7161414	4.96E-147	TGFB3	-2.804449	3.72E-17
TOX3	3.7159319	9.85E-18	HLA-DQA1	-2.821499	0.003206
TLR2	3.7142455	0.0001775	CRLF1	-2.867949	7.66E-29
EHF	3.7123876	2.69E-41	PTGFR	-2.882643	0.0055827

B4GALNT2	3.7099208	1.89E-25	S100A1	-2.893085	0.0019815
PCDHA11	3.708073	6.4E-55	RAC3	-2.895513	2.42E-117
ELF3	3.7077331	0	GAL	-2.919296	1.13E-15
KYNU	3.7037261	0	ESRRB	-2.921997	1.1E-15
MALL	3.7031817	0	ESR2	-2.927149	6.29E-06
PEAR1	3.7030486	2.13E-65	RFXAP	-2.946875	1.07E-35
SOX17	3.7004397	0.0000822	NPPC	-2.997902	1.05E-06
CYP4F3	3.6994949	6.9E-125	GDF6	-3.038135	3.07E-07
DDX3Y	3.6930575	8.82E-115	SECTM1	-3.048759	2.58E-08
ADGRE2	3.6920397	5.26E-39	RAC2	-3.085488	0.0000763
CSRP1	3.6808938	0	APOBEC3H	-3.108836	0.0000173
PRRX2	3.6780719	1.07E-08	CSPG5	-3.123259	3.25E-28
KCNK3	3.6775595	1.33E-28	OSTN	-3.137504	1.87E-25
GPR39	3.6764609	1.7E-46	MDK	-3.154812	0
NT5E	3.6755651	1.78E-226	FGF13	-3.16694	9.57E-29
MMP10	3.675309	8.37E-13	FGFR2	-3.167727	8.5E-48
SLCO3A1	3.6745997	3.03E-128	IL17RB	-3.203736	1.24E-41
CCDC170	3.6683785	1.35E-06	PTGER1	-3.203872	0.000015
WNT7B	3.6665349	0	NGFR	-3.205904	3.8E-12
NCMAP	3.6644828	2.54E-17	MASP2	-3.234342	5.28E-13
PLA2R1	3.6614146	2.54E-49	FGFR3	-3.267357	2.19E-94
MAP10	3.6605135	8.68E-19	TNFRSF14	-3.273018	0.0021832
FYB1	3.659891	2.24E-99	FLT1	-3.289879	4.9E-77
PKP3	3.6595833	7.57E-107	SEMA5B	-3.295456	1.31E-07
MYD88	3.6592444	2.94E-97	PTH1R	-3.340307	3.19E-07
AKR1B15	3.658704	5.29E-26	PIK3R5	-3.350497	2.8E-07
TRIM31	3.6569579	1.75E-17	HTR3A	-3.369234	0.0094444
ICAM1	3.6568998	8.4E-86	ROBO2	-3.375867	2.69E-09
FAM110C	3.6553518	1.38E-07	CRABP2	-3.401828	7.67E-160
XAGE1B	3.65469	3.41E-74	FLT4	-3.424649	6.12E-110
AC245033.1	3.6520767	0.001686	SEMA3D	-3.43221	1.29E-43
XAGE1A	3.6497887	8.85E-74	ACVR1C	-3.491853	0.0014311
DENND2D	3.6496155	5.64E-08	SEMA6A	-3.522461	1.29E-73
DACT2	3.6496155	2.65E-10	ACVRL1	-3.542821	2.04E-09
ECHDC3	3.6470586	5.34E-19	TNFRSF13C	-3.549162	8.15E-25
IL1A	3.6466016	1.82E-08	VIPR1	-3.583683	3.85E-24
ARPP21	3.6438562	0.0070415	SEMA6D	-3.673772	9.57E-13
CACNG7	3.6415025	3.77E-107	FLT3	-3.708345	1.95E-07
OCIAD2	3.6376985	2.51E-124	CNTFR	-3.771059	1.5E-21
ANKRD22	3.6374299	1.01E-06	LGR5	-3.798975	6.78E-20
EVC2	3.6307662	7.47E-22	IFITM1	-3.809791	3.66E-63
RBP4	3.6295459	1.63E-13	COLEC12	-3.813883	3.73E-43
SPP1	3.6272624	5.97E-301	IL21R	-3.894009	1.09E-06
HIST1H4H	3.6151245	4.35E-25	GHR	-3.989139	1.75E-17
CDH6	3.6139139	1.5E-22	PI15	-4	1E-16
IL18	3.6109239	0	SSTR2	-4.015825	9.24E-20
TMEM26	3.6088092	2.1E-07	SDC2	-4.031544	1.7E-121
CLDN1	3.6083465	0	CD1D	-4.050626	5.87E-06
FGL1	3.6082248	3.59E-143	SYK	-4.06301	7.51E-51
NIPAL4	3.6064352	4.47E-147	HLA-DQB1	-4.076316	5.52E-52
UTY	3.6027581	2.41E-35	CTF1	-4.079097	6.73E-55
NRG1	3.597769	0	HLA-DPA1	-4.08145	7.39E-21
P4HA3	3.592404	3.68E-155	INPP5D	-4.124071	7.89E-61
PDE4B	3.5922774	4.04E-263	NOS2	-4.142958	1.96E-12
ZNF544	3.5909414	1.06E-85	GDF7	-4.16355	2.49E-87
GBP3	3.5892499	1.42E-34	BMP7	-4.180956	5.51E-135
IFIH1	3.5849625	4.39E-26	SLIT2	-4.194737	1.13E-247
GABRA5	3.5838332	1.5E-195	BST2	-4.261378	4.07E-40
ETS1	3.5816359	1.98E-215	PRDX2	-4.361863	3.08E-245
NPHP3-ACAD11	3.5774288	0.0031917	ANGPTL2	-4.375039	4.08E-16
PLK2	3.5760569	0	HLA-DPB1	-4.378512	6.62E-40
GBP2	3.5745351	1.8E-49	NFATC4	-4.38202	1.09E-143
IL15	3.5739914	1.92E-75	DES	-4.446256	0.0016917
LGSN	3.5722274	2.41E-194	SEMA3G	-4.469749	4.92E-17
P2RX5	3.5707486	5.67E-16	TMSB15A	-4.472266	4.48E-15
DOCK10	3.5698556	8.58E-139	ADCYAP1R1	-4.481127	3.98E-35
VSIR	3.5679803	1.31E-269	TSLP	-4.507795	6.54E-17
TFPI2	3.5649192	3.73E-18	BLNK	-4.592457	0.0002356

FA2H	3.5638711	5.52E-94
HENMT1	3.5635889	3.68E-25
NEDD9	3.562846	0
CCDC85A	3.5555187	7.1E-21
FFAR4	3.5545889	0.0074677
MLKL	3.5491173	8.15E-51
ETV5	3.5486871	2.24E-225
IL11	3.5479208	9.85E-141
SCN9A	3.5472204	0
TM4SF18	3.5418344	4.88E-140
AGR2	3.5404424	0
GNG11	3.5379101	3.66E-143
ANXA8L1	3.5364375	2.74E-138
CSGALNACT1	3.5360779	0
A1CF	3.5360529	7.6E-08
GSAP	3.5356288	2.47E-76
GLP2R	3.5347997	3.39E-93
FGB	3.525614	2.84E-161
MGAM	3.523562	8.49E-14
TMC5	3.523562	3.52E-116
SNAP25	3.5190746	9.55E-148
SNX19	3.5177268	5.15E-252
INSYN2B	3.5136237	8E-17
DUSP6	3.5112228	5.87E-31
SLC16A4	3.5100542	0
APOL6	3.5094115	3.73E-69
STK32B	3.5084838	6.21E-45
MAFK	3.5076946	0
SLC28A1	3.5025003	0.001557
C11orf86	3.5019631	6.52E-26
LYL1	3.4964258	0.0067433
KRT4	3.4939888	0.000016
PCDHGB1	3.4937377	6.13E-11
CCL20	3.4924359	4.54E-07
ZNF671	3.4893848	8.4E-10
MEST	3.4837057	6.8E-204
KRT80	3.4817805	0
MUC13	3.4814864	5.74E-155
PARP14	3.4806685	4.23E-250
CSTF2T	3.4805042	4.31E-94
C5	3.4798177	0
RAET1E	3.4749613	5.49E-11
PCDHGA2	3.4731717	3.89E-13
NPY4R	3.469712	3.84E-206
UGT1A7	3.4656636	1.85E-28
ST6GAL2	3.4627068	6.66E-91
CXCL2	3.4617801	2.41E-42
NTN4	3.4607906	0
HIPK4	3.4594316	0.0024512
TUBAL3	3.4594316	7.15E-07
ABCA10	3.4594316	7.96E-11
OPLAH	3.4575701	1.44E-68
SLCO1B3	3.4547663	6.65E-170
NUAK2	3.4524402	0
F2RL2	3.4521845	2.22E-56
KCNF1	3.4398694	2.84E-17
CPLX2	3.439664	0
PAPPA	3.4384015	7.69E-122
PDGFB	3.4364772	2.12E-123
SLIT3	3.4342946	1.61E-294
VIP	3.4329594	0.0022533
SULT2B1	3.4302534	1.88E-161
SHC3	3.4299878	1.43E-06
C3	3.4287112	0
MAATS1	3.4262648	0.0004532
CD163L1	3.4262648	0.0000411
CFB	3.4248185	2.11E-216
KIAA1217	3.4237588	6.62E-88

PROK2	-4.714246	0.0048811
HLA-DRB1	-4.870878	8.44E-31
HLA-DOA	-4.877909	1.36E-91
CGB2	-4.925179	1.26E-15
SPINK5	-5.209453	0.001004
FGF9	-5.807355	9.71E-07
HLA-DRB5	-6.847997	3.9E-07
PAK5	-6.946419	0.0003532
FGF23	-8.003752	0.0000611
GPR32	-8.260528	0.0034823
CORT	-8.946419	0.0004421
XCL1	-9.040746	0.0001838
LCN6	-9.058894	0.0005502
AMBN	-11.92308	6.58E-14

AKR1C2	3.4226986	0
PCDHAC2	3.4202703	8.88E-97
TNFSF15	3.4201516	1.72E-36
SPOCK1	3.418727	0
ZNF354C	3.4176114	2.02E-24
GALC	3.4174593	1.15E-247
VTN	3.4147856	1.7E-38
GCNT3	3.4147435	0
EVPL	3.4140779	1.71E-73
SGCD	3.4135439	2.13E-182
EMP1	3.4111954	3.11E-62
DPYSL3	3.4052509	0
ALDH1A1	3.4039671	0
COL16A1	3.4038969	4.73E-17
RIOX1	3.4020984	6.82E-41
PID1	3.4016553	3.57E-30
HAS2	3.4009997	8.61E-40
F5	3.3989505	1.11E-27
CGAS	3.3980998	2.3E-32
SLCO1B1	3.3980311	7.75E-08
AGMO	3.3978769	2.27E-21
ARSI	3.3960598	8.03E-21
NAP1L5	3.3948596	4.58E-08
ASIC2	3.3923174	0.0062907
PTPN6	3.3906863	2.77E-79
TM4SF1	3.3892596	0
FAM111A	3.3885528	3.61E-99
DNAH5	3.3870231	3.08E-52
SLFN11	3.3837934	3.77E-75
TNS4	3.3827063	0
PSMD5	3.3817607	2.31E-273
S100A6	3.3815937	0
KCNH1	3.379172	2.49E-119
ANXA3	3.3782523	0
B3GALNT1	3.3763759	0.0018769
PLAU	3.375287	0
EPM2AIP1	3.3752439	2.03E-77
BDKRB1	3.371817	9.33E-18
LOXL2	3.3697105	0
JPH2	3.3692338	9.53E-16
FOXL2	3.3671169	3.64E-09
GFRA1	3.3658452	5.2E-96
IGFBP1	3.3650969	0
C2CD2	3.3623424	2.69E-67
DTX3	3.3600547	7.92E-69
BICC1	3.3567924	0
NEURL3	3.353637	0.0048825
TRPC6	3.353637	2.55E-06
DCBLD2	3.3488728	0
SYNPO	3.3486399	0
CNKSR2	3.3485223	7.17E-105
UPK3B	3.3444842	2.98E-38
MUC3A	3.33985	2.15E-33
CTSZ	3.3397892	1.79E-244
ME3	3.3387333	1.48E-59
PLEKHG6	3.3378696	0.0009514
COL7A1	3.3362475	0
DHRS3	3.3361052	0
TNFRSF10A	3.3343592	1.34E-126
SLC22A4	3.3334237	1.99E-35
KRT86	3.3329833	1.66E-39
BMPER	3.324299	4.34E-118
AMTN	3.3219281	0.000597
SMOX	3.3180847	0
DUSP5	3.3176983	1.51E-181
COPZ2	3.3168571	4.23E-13
MLH1	3.3164426	5.4E-269
KRT83	3.3146965	0.000017

PCDHA12	3.3125294	2.45E-09
HRH1	3.3106128	3.48E-33
NLRP2	3.3104553	4.78E-140
NPR3	3.3085599	1.65E-133
PCDHA3	3.3070548	9.07E-22
FAM111B	3.3025628	7.61E-07
MYOF	3.301328	0
COL5A2	3.3000032	0
KDM5D	3.2998053	1.39E-49
ZNF229	3.2996929	2.03E-44
CD44	3.2994354	0
CES4A	3.2957984	2.09E-10
TRPA1	3.2901026	1.62E-31
SP100	3.2901026	1.06E-123
ADPRH	3.2892909	6.99E-25
RSPO3	3.2885275	1.36E-80
HGD	3.2864863	1.12E-95
MAP3K7CL	3.2851014	1.61E-21
LTBR	3.2840628	0
TNK1	3.283793	1.19E-09
TMEM156	3.2836547	2.71E-170
CLDN3	3.2831873	4.45E-19
KIFC3	3.282178	0
ABCG2	3.2820956	2.45E-158
MYL9	3.2806939	8.4E-36
LAMB3	3.2796999	0
PTPRD	3.2794234	4.63E-18
GLIS3	3.2759264	4.71E-257
BOK	3.2752278	5.27E-37
TM4SF4	3.2736998	4.65E-14
PCDHGA7	3.2718046	1.49E-20
CTSS	3.2707938	6.7E-170
CASP4	3.2691267	1.33E-46
WWC3	3.2686761	0
ESYT3	3.2684888	1.56E-06
ENTPD8	3.2647032	0.000043
SLC45A1	3.2636796	1.94E-93
ARSE	3.2624296	3.46E-196
THSD4	3.2618722	0
GASK1B	3.259691	1.43E-124
TMCC1	3.259407	3.63E-62
KCNMA1	3.258981	8.54E-193
MT1A	3.2573878	0.0016787
ADM	3.255979	1.87E-225
AADAC	3.2504286	7.33E-45
BANK1	3.2479275	2.05E-26
DCDC2	3.2476494	2.14E-132
PRG4	3.2462392	5.46E-25
PMEPA1	3.2457313	0
S100A4	3.2425915	1.61E-231
ARFRP1	3.2409697	1.98E-57
EDN2	3.239705	1.83E-31
IL33	3.2394659	0.0008242
SLCO1B7	3.2391877	1.38E-13
ITIH2	3.2387869	3.77E-10
VSX1	3.2377676	2.69E-08
ANXA1	3.2351739	0
ACOT4	3.2340553	5.74E-12
INSL3	3.2326608	0.000705
THBD	3.2325642	6.83E-124
GBP1	3.2254201	1.3E-16
NPY4R2	3.2240439	1.7E-141
FOSL2	3.2225453	0
C1S	3.2221545	0
ZNF469	3.2209992	1.44E-52
PDE1C	3.2184235	8.14E-76
PCED1B	3.2183827	0
TAPBPL	3.216414	2.27E-19

TESC	3.2162309	3.16E-212
LIPH	3.2139209	1.25E-163
DUSP23	3.2135907	4.82E-53
CXCL5	3.2093928	0
S100A16	3.208815	1.08E-228
RPH3A	3.2051144	8.82E-06
GSDMD	3.2040392	1.5E-65
GREM1	3.2034343	2.68E-147
CD14	3.2021238	5.88E-12
AXL	3.1989226	0
FOXA2	3.1975637	2.15E-122
ALDH3A1	3.1965758	0
FGA	3.1965392	0
LAMC2	3.1948697	0
SCEL	3.1926451	6.28E-06
MMP24	3.1919255	0
ABCC3	3.1904256	0
ISG20	3.1900747	1.02E-33
DAB2	3.1893797	3.19E-118
MBP	3.1872866	0
PTPRH	3.1848209	2.54E-75
RPS4Y1	3.176831	3.07E-220
AIFM2	3.1753589	3.23E-159
CCN5	3.173528	1.01E-143
WDR72	3.1731597	1.29E-193
BCL2L15	3.169925	0.0004473
ADHFE1	3.169925	0.0000206
TNFRSF12A	3.1633998	1.83E-218
PPARGC1A	3.1600265	1.76E-55
GPX2	3.1599059	5.92E-260
SYNPO2	3.1598713	1.04E-64
THBS1	3.1591986	0
STK31	3.1559181	1.94E-09
PNPLA4	3.1553213	1.63E-80
ANXA8	3.1550213	6.6E-117
NCKAP5	3.154577	2.28E-42
SIK1B	3.1537648	0
FLG	3.1529519	4E-25
NTRK3	3.1529103	0
DUSP4	3.1516304	0
KCNK6	3.1512188	1.82E-38
KLHL35	3.1505597	0.0000325
MYO1E	3.1483892	0
CCDC68	3.1475324	5.65E-56
SHANK2	3.1457059	6.5E-169
MGST1	3.1454489	0
B3GNT7	3.1450503	0.0000023
IGFBP4	3.1446632	0
KPNA7	3.1443899	0.0000822
SFRP4	3.1409021	4.07E-32
SMIM6	3.1406604	2.2E-09
PLPP2	3.1388522	1.75E-145
HIST1H2AC	3.1367853	1.95E-45
IRF5	3.1361367	1.3E-38
CNTNAP3B	3.1352001	1.4E-207
NQO1	3.1341527	0
AP1M2	3.1313222	1.88E-13
ALPK2	3.1302503	1.32E-140
AL590132.1	3.129283	0.0025607
C10orf55	3.129283	0.0000167
C19orf33	3.1261069	0
PTPRJ	3.1260897	0
PRAG1	3.1251887	5.94E-272
NAV3	3.1242474	1.71E-41
TNS3	3.1237315	0
PLP2	3.1231224	0
ITGB4	3.1229061	0
OAS3	3.1214036	3.11E-159

NUTM2E	3.1210154	0.000118
KLB	3.1210154	5.11E-13
DOCK5	3.118666	0
FRMD3	3.11862	3.32E-83
C16orf45	3.1185912	9.64E-131
CCN2	3.1169717	1.24E-252
PYCARD	3.1092114	4.52E-17
PPP1R3C	3.1073737	3.26E-73
PHYHIPL	3.1069152	1.4E-10
IFIT2	3.1069152	1.27E-19
BST1	3.1068352	1.49E-52
LBH	3.1053132	0
MMP1	3.1043367	0.0033749
ZNF575	3.1026151	1.16E-07
ATP8B1	3.1022863	1.08E-265
CACNG4	3.0997412	1.2E-37
GOLT1A	3.0993443	3.9E-30
ADRA1D	3.0976108	3.77E-20
FANCF	3.0966474	2.14E-99
NOSTRIN	3.0954196	3.99E-17
EGFR	3.0943557	0
TGM2	3.0925758	0
NR0B1	3.0914298	5.77E-36
G6PD	3.088316	0
NR2E3	3.0874628	0.0010014
SLC8A1	3.0874628	8.26E-18
STAC	3.086509	1.08E-47
CLEC2B	3.0837684	8.13E-09
CLDN23	3.0817941	2.9E-20
MAGEH1	3.0771152	2.55E-31
POF1B	3.0718618	3.81E-136
NR1H4	3.071661	2.72E-07
DGKG	3.069162	4.35E-33
SH3TC1	3.0680454	1.44E-16
HNF4A	3.0654054	5.83E-172
S100P	3.0649028	7.24E-17
IGFBP6	3.0645503	2.33E-87
IER3	3.0643415	1.31E-302
TLR3	3.0641303	1.95E-06
CCL5	3.0622843	6.04E-07
CLU	3.0619243	0
TRIM67	3.0588937	0.0021672
KCNJ6	3.0588937	6.57E-08
EPAS1	3.0582523	0
HNF1B	3.0575963	4.32E-115
PTGES	3.0571803	6.72E-105
MLPH	3.0571353	0
OPN3	3.0553014	1.19E-256
MNS1	3.0511043	3.19E-11
TMSB4X	3.0502601	0
P3H2	3.0488961	5.74E-228
TP63	3.0483013	1.6E-107
TNFAIP6	3.0443941	3.95E-06
IL20RB	3.0432278	9.87E-51
MAP1LC3A	3.0410273	3.21E-28
ALOX5AP	3.0387783	4.04E-08
TNFSF10	3.0381351	9.14E-09
PLXNA4	3.0356239	0.0000795
LMCD1	3.0336287	1.93E-193
PDCD1LG2	3.0324215	4.84E-06
MGMT	3.0319429	3.78E-24
RNLS	3.0319266	5.14E-41
LGALS8	3.0277666	1.84E-181
EVA1A	3.0248689	1.94E-35
TNFRSF9	3.0232074	9.82E-54
AC073611.1	3.0223678	0.0023634
LPAR6	3.0216951	6.27E-08
CALB2	3.0213105	2.92E-07

FKBP11	3.0192025	2.8E-99
P2RY6	3.0173472	1.18E-16
C19orf66	3.0162474	1.82E-48
CD68	3.0157329	8.53E-96
ITGA3	3.0140626	0
HAVCR1	3.0131065	4.14E-224
TRIM16L	3.0130913	0
GOLGA7B	3.012824	3.15E-10
KRT18	3.0109079	0
CD274	3.0079052	2.58E-31
ARSD	3.0044083	1.21E-229
FAM19A5	3.0013157	2.43E-24
TSPYL5	3.0005548	1.32E-121
VXN	3	0.0002577
DTX4	3	2.51E-39
ABLIM3	2.999499	1.44E-121
PON2	2.9992361	0
UCN2	2.9945952	3.23E-30
KRT19	2.9935737	8.26E-79
ADGRF4	2.9933434	1.21E-99
BTG4	2.9904772	0.0096285
LXN	2.9900866	2.41E-108
DPYD	2.9845227	1.56E-87
PDGFD	2.984078	7.16E-72
HR	2.9834646	1.13E-153
C1orf116	2.9820396	2.64E-83
MSC	2.9817827	4.76E-194
AMIGO2	2.9810059	0
C1QTNF1	2.9807157	1.94E-213
TSPAN1	2.9805476	0.0000144
GPRIN2	2.9796514	3.34E-58
FERMT1	2.9794031	6.27E-116
STC1	2.9789301	2.96E-145
SLC16A3	2.9696704	3.23E-188
LGALS4	2.9696264	0.0058143
ANXA2	2.9677393	0
TBX4	2.9655665	1.84E-11
C6orf132	2.9652346	2.22E-09
SPRY4	2.9652346	1.24E-20
NMI	2.9620186	7.85E-45
NRP2	2.9612729	0
EFHC2	2.9608294	0.0000163
COL5A1	2.9599359	0
TLR4	2.959358	0.000038
NSUN7	2.959358	6.25E-12
SCG2	2.9581159	5.81E-19
TLR1	2.9574023	3.85E-27
EBI3	2.9562674	1.21E-25
PCDHA1	2.9541963	0.0000925
LRRK1	2.9523546	3.28E-187
MISP	2.9515499	0
ABCC2	2.950395	0
RGL3	2.9482175	2.54E-07
MUC5B	2.9481881	1.87E-95
DSCAML1	2.9475326	0.0000788
FAM129A	2.9448139	0
RNF180	2.9434165	0.0002627
FBXO6	2.9402278	1.92E-11
CCDC198	2.9395354	2.49E-19
VEGFD	2.9385995	0.0071338
PDE7B	2.9385995	2.95E-98
SPX	2.9351189	1.19E-70
SLC23A1	2.9316682	3.93E-24
NFATC2	2.9293454	2.15E-19
HRNR	2.9289169	5.96E-21
HAS3	2.9287883	2.18E-100
PARP9	2.9268113	4.34E-57
LIMCH1	2.9254166	0

AMPD3	2.9243478	4.32E-71
CCDC33	2.9196579	1.54E-16
RASSF6	2.9146679	2.29E-57
ENKUR	2.9142701	0.0001556
ANKRD2	2.9140861	3.13E-11
LCN2	2.9091876	9.24E-145
CLVS2	2.9068906	0.0000227
ITGB1	2.9063401	0
PTPN20	2.9046779	2.97E-29
DTX3L	2.9023033	8.08E-103
ALDH3B1	2.900966	0
FN1	2.8973882	0
SAMD9L	2.8954341	4.32E-33
ETV4	2.8952781	1.31E-103
NPEPL1	2.8952771	2.54E-107
MCTP1	2.8910887	1.17E-19
SOWAHB	2.8907709	0.000374
SEMA3C	2.8899232	0
PLCXD3	2.8798826	8.73E-66
BTG1	2.8759472	0
MYZAP	2.8756091	8.19E-23
SLN	2.8744691	0.0086717
TRANK1	2.8666677	7.34E-129
PLEK2	2.8655666	6.27E-49
CFAP57	2.8643449	5.79E-11
PTPRE	2.8640775	3.68E-27
BCL3	2.8638147	4.34E-165
CNR1	2.8601423	1.1E-15
PTGR1	2.8596963	3.67E-287
SLC22A3	2.859256	0
NLGN4Y	2.8549069	3.07E-29
MYPN	2.8538294	3.87E-20
LMF1	2.8505801	1.55E-63
SEMA4B	2.849904	0
TIMP4	2.8498479	1.98E-103
HKDC1	2.8493246	0
COL12A1	2.8481799	0
MLH3	2.8468972	1.07E-99
NRP1	2.8456458	0
ATP8B4	2.8454901	2.17E-07
SCNN1A	2.8410609	1.64E-37
CDH19	2.8410609	1.4E-39
CUZD1	2.8399596	1.87E-08
EFEMP1	2.8372076	4.12E-248
EPS8L3	2.8372049	2.77E-08
TPM2	2.8315745	0
FGD6	2.8296286	0
SLC2A5	2.827819	0.0094629
SSPN	2.8251662	2.55E-16
GBGT1	2.8214991	8.4E-14
NTS	2.821388	1.3E-51
PAQR5	2.8209826	0
MMP28	2.8206763	1.07E-10
ITIH5	2.819792	3.66E-23
AKR1B1	2.8165548	0
IL22RA1	2.8124982	3.44E-10
SHISA4	2.8122025	6.38E-40
FAM20C	2.8120773	3.67E-73
AGBL2	2.8118775	2.19E-10
RIC3	2.8107296	3.56E-10
TMEM238	2.8037971	8.34E-15
ODF3B	2.8031937	1.63E-14
TM4SF20	2.8028838	9.82E-126
TGIF1	2.8024937	0
IL1R1	2.8017463	1.28E-122
APBB1IP	2.7953823	1.05E-23
TPM1	2.7947425	0
CPA4	2.7939344	2.91E-13

GRAMD2B	2.793641	3.25E-21
SLC16A5	2.7926324	9.97E-131
ERVMER34-1	2.7914134	0.0002806
SIDT1	2.7894774	1.51E-22
PDE4D	2.7863586	0
COL22A1	2.7858752	4.98E-06
RAB27B	2.7844636	4.38E-170
PDE3A	2.7825515	1.56E-86
LAMA5	2.7812489	0
OSGIN1	2.7792764	2.8E-85
MGLL	2.7747007	0
PTK6	2.7695917	6.24E-20
ASB9	2.7671351	2.47E-31
C1R	2.763008	0
SFN	2.7625632	2.69E-68
DOC2B	2.7625007	2.14E-07
HNF1A	2.7598974	1.3E-34
RHOD	2.7574297	7.32E-30
HECW1	2.7554164	2E-41
LTB	2.751665	8.3E-07
HHIPL2	2.7508132	1.16E-36
LIF	2.7484564	3.04E-301
AP003419.1	2.7434895	4.45E-15
CEBPD	2.7368187	8.91E-73
AKR1C3	2.73504	0
CYB5R2	2.7343401	5.3E-12
TAGLN	2.7316586	5.69E-112
FSCN2	2.7305867	6.42E-13
ACY3	2.722466	0.0088263
NAV2	2.7216988	8.18E-247
SPINT2	2.709645	0
LURAP1L	2.7058992	1.45E-133
ADAM28	2.7042614	2.68E-08
PEG10	2.7021566	1.18E-281
HS3ST1	2.7004397	0.0005142
GPR37L1	2.7004397	1.84E-07
JAG1	2.6973577	0
LGALS1	2.6956615	0
ARHGEF28	2.6923714	9.65E-248
CLCF1	2.6815898	1.53E-35
RELB	2.6814377	6.34E-109
ACSS3	2.6780719	7.3E-07
FAAH2	2.6774087	2.24E-10
MYOM3	2.6772593	6.53E-42
ROS1	2.6770231	9.42E-22
USP9Y	2.6752514	2.65E-51
ZC3H12A	2.6737498	6.76E-84
DRAM1	2.673249	2.7E-219
UPK1A	2.6724253	6.82E-08
B3GALT5	2.6713773	3.57E-15
PRSS3	2.6690831	1.2E-10
NCF2	2.6651328	2.65E-45
PTPRQ	2.662965	0.0069443
TOX	2.662965	7.11E-06
CCDC144A	2.6625384	2.15E-80
SLC52A3	2.66073	5.29E-08
CLPTM1L	2.6571266	0
F13B	2.6571123	0.0017823
ATP1B1	2.6557723	0
CA12	2.6549846	0
PCDHA5	2.6545034	1.02E-15
TMEM184A	2.654048	1.76E-113
MVP	2.6514326	4.09E-282
CAPN15	2.6481741	2.23E-75
PTPRB	2.6443579	2.11E-41
DDC	2.6438562	5.89E-07
ISLR	2.6421064	0.0013504
PTHLH	2.639359	2.39E-33

TRIML2	2.6381005	2.06E-66
RRAD	2.6380738	0.0000385
SLC22A18	2.6376696	3.2E-134
IL32	2.6345945	8.32E-103
CAV2	2.6333219	0
UBASH3B	2.6304113	0
SORCS2	2.6301513	2.58E-27
PCDHB5	2.6291623	1.04E-17
CDH17	2.6289697	1.98E-49
HTR1D	2.622778	2.21E-105
KSR2	2.6224372	1.69E-22
SCARA5	2.6206542	1.69E-67
DOCK2	2.6185151	2.75E-18
CCR7	2.6182387	2.78E-08
SOCS3	2.6176511	4.24E-52
BCL2L1	2.6172546	0
ESRP1	2.6156593	7.22E-08
RIN2	2.613564	2.38E-192
CLDN16	2.6114347	0.0000914
SYTL2	2.6094979	1.82E-72
B3GNT9	2.6088092	7.6E-51
DUSP1	2.6058148	0
HACD4	2.6029964	2.14E-28
CDH1	2.6015664	0
CAVIN1	2.6001956	0
SAT1	2.5995186	0
CRYZ	2.598908	2.3E-167
TIMD4	2.5977298	6.68E-07
LRRC27	2.5941458	6.22E-36
BTBD11	2.5929087	1.81E-249
PFKP	2.5902355	0
NCOA7	2.5890848	0
COLCA2	2.5875737	9.17E-08
PLA2G4A	2.5868961	1.43E-211
TGFBR2	2.5868877	0
CAV1	2.5852486	0
UGT1A6	2.5849625	0.0001525
TM4SF19	2.5849625	4.56E-07
KCNN4	2.583731	5.51E-70
SMAD3	2.5809913	0
NHLRC1	2.580047	7.38E-15
APOH	2.5795793	1.6E-23
LGALS3	2.5795122	8.07E-130
ARHGEF5	2.5787839	1.55E-08
PTPRM	2.5779597	0
CPPED1	2.5768429	4.01E-181
CCNJL	2.5751003	4.27E-106
INHBB	2.5696382	8.54E-78
SLC27A2	2.5682714	7.54E-257
BASP1	2.5664527	0
AC005324.3	2.5661035	5.42E-23
TUBA4A	2.5642375	1.13E-151
ANKFN1	2.5618789	7.33E-07
CLEC4E	2.560715	0.0005434
ANKRD30A	2.5594274	9.62E-07
FOXQ1	2.5592322	5.67E-22
SPSB2	2.5578128	5.79E-64
FOXS1	2.5537116	7.02E-07
AIFM3	2.5517956	0.0019208
NRCAM	2.5514966	0
MAML3	2.549734	9.83E-156
COL18A1	2.5487591	0
PCDHGB5	2.547141	2.23E-47
PLD5	2.5443205	2.43E-27
PHLDB2	2.5403877	0
OXTR	2.5360529	6.19E-40
GCNT2	2.5336914	5.93E-63
TCIRG1	2.5330837	9.84E-203

IGFBP7	2.5255194	1.15E-283
RASSF10	2.5252563	5.1E-23
STEAP1	2.5197753	4.54E-171
MAFF	2.5121505	2.16E-35
SUSD4	2.5113396	1.43E-10
CCN4	2.5109619	2.35E-06
KLHDC9	2.5104843	0.0000153
MX1	2.5092736	7.6E-14
LYSMD2	2.5082177	2.59E-42
CCDC149	2.5073416	1.8E-20
FAM107B	2.5047202	0
IRAK2	2.503242	7.64E-190
TMEM45B	2.5025003	0.0002566
AK5	2.5009841	1.36E-10
SAMD9	2.5004589	1.98E-74
GALNT9	2.4982509	0.0005972
TRIM34	2.494961	2.2E-30
AVPI1	2.4946448	1.16E-148
RBM24	2.4930677	7.21E-86
GALNT10	2.4920712	0
LHFPL2	2.4917814	0
ATOH8	2.4905701	5.45E-16
NUDT18	2.4846808	3.15E-66
ZNF418	2.4835172	0.0000117
LRRK2	2.4765669	2.6E-124
FAM114A1	2.4763198	2.07E-203
TSKU	2.4756902	0
HPGD	2.4732725	1.79E-266
GALNS	2.4727123	4.62E-135
SVIL	2.4726667	4.38E-208
SHH	2.4673109	1.45E-19
EXOC3L4	2.466318	1.82E-10
PLAUR	2.4636403	6.85E-100
SRGAP1	2.4628676	3.26E-229
NGF	2.4594316	0.0003142
PAX7	2.4594316	2.31E-15
H2AFJ	2.4521905	1.87E-74
TSPAN4	2.4501297	4.37E-190
MICAL2	2.4495012	4.8E-160
MACROD2	2.4493074	0.0083623
CD24	2.4486184	0
EGLN3	2.4475933	1.32E-161
SYNDIG1	2.4462562	7.02E-06
BAMBI	2.4451337	8.17E-91
ITGA11	2.4407959	3.07E-66
CITED4	2.4399994	2.06E-68
ARPC1B	2.4390799	0
INPP4B	2.4386914	6.59E-140
GPRIN3	2.437296	7.75E-283
AKR1B10	2.4362231	0
ADGRG6	2.4341672	3.43E-266
GATM	2.4336846	1.16E-30
FAM174B	2.4327055	1.76E-40
ITGB5	2.4317117	0
GAS2L1	2.4308915	3.09E-44
MKRN3	2.4306344	0.0003273
BIRC7	2.4306344	3.19E-06
PADI1	2.4284047	2.17E-10
CA9	2.4274212	5.12E-44
ITGA2	2.4240263	0
ERRFI1	2.4238855	0
TACC2	2.4227502	1.88E-299
HSPB1	2.4225917	0
FADS2	2.4221606	3.76E-280
SLC38A10	2.4212454	5.07E-177
GABRE	2.4186193	6.71E-83
SOCS6	2.4155541	0
SYT13	2.4144631	3.4E-149

DHRSX	2.4135375	5.31E-92
RAET1G	2.4109331	1.7E-13
CRIM1	2.4104593	0
AKR1C1	2.4091849	0
CYP1B1	2.4087368	3.18E-101
AC073111.5	2.4080847	0.0068781
ZNF141	2.4037222	3.31E-15
TXNRD1	2.4028291	0
PCDHGB4	2.4020984	4.03E-11
COL4A3	2.4008236	4.08E-51
CLIC3	2.3971473	0.0000452
ZBTB20	2.396659	1.94E-59
SLC7A5	2.3963987	0
ANXA4	2.3927061	0
C7orf50	2.3925177	4.64E-77
AC022414.1	2.3912601	2.2E-12
TRIM16	2.3845846	0
ADGRE1	2.3833286	5.76E-29
RNASE4	2.3825496	6.99E-111
GGT1	2.382505	1.09E-80
TRABD2A	2.378765	1.12E-19
NFKBIZ	2.3776326	4E-79
FMNL1	2.3768718	3.63E-174
LASP1	2.3762467	0
ZFP92	2.3750394	5.64E-06
BCL9L	2.3733526	0
F7	2.3717185	6.05E-18
EHD2	2.3693512	0
FBXO15	2.3681511	0.0000677
HSPG2	2.3673648	0
AC090517.4	2.364723	6.67E-24
TRPM2	2.3636726	8.01E-10
ZNF860	2.3630973	5.58E-13
KCNJ16	2.3625701	0.0000322
IDH1	2.361109	0
RGS20	2.3580239	1.64E-42
MYADM	2.3577398	0
HS3ST6	2.3563915	7.61E-07
MCMDC2	2.3530666	6.27E-07
COL11A1	2.3521283	4.71E-24
ZNF718	2.3504972	0.0008007
RRAS	2.350256	4.57E-174
SH2D4A	2.349043	1.09E-130
PCDH1	2.3460658	1.77E-83
LAMA2	2.3457748	1.88E-06
SEMA3A	2.3453158	2.24E-132
GJB3	2.3439544	0.0003041
SLC17A3	2.342106	0.0000144
IER5L	2.3401791	1.54E-56
PIGQ	2.3400486	1.2E-73
MFSD3	2.3361675	1.23E-76
CNTN1	2.3360128	0
GTF2IRD2	2.3358866	3.54E-14
ITGB8	2.335603	4.58E-09
FBXW10	2.3300713	6.23E-18
GLI1	2.3264178	3.89E-114
OSBPL10	2.3244944	5.3E-117
UGDH	2.3231752	0
ANPEP	2.3219281	2.44E-13
SLC6A6	2.3197526	0
CYP2S1	2.3191286	6.2E-187
B4GALT4	2.3158945	3.74E-275
B4GALT1	2.3154393	0
VLDLR	2.3135403	3.56E-227
COL4A1	2.3127303	0
IL6ST	2.3099555	0
AMBP	2.3096845	0.0000202
EFNA1	2.3083158	3.59E-129

MAP3K8	2.3080392	2.52E-81
LSAMP	2.3053452	1.33E-08
THSD7A	2.3035135	8.98E-80
SCHIP1	2.3011695	0.0041134
SLC2A1	2.3010102	0
EXOC3L2	2.2999018	1.24E-10
FLNB	2.299863	0
SLC22A1	2.2976805	0.0067778
PIWIL2	2.2976805	0.0004123
SRXN1	2.297581	0
FAM102A	2.2928479	1.95E-253
GPAT3	2.2927479	4.97E-96
ZNF365	2.2913564	3.04E-21
FN3K	2.290632	6.68E-16
PTPRN2	2.2880803	2.05E-13
MICALL2	2.2880803	4.2E-30
ZNF25	2.2846767	1.98E-35
CSF2RA	2.2825794	6.68E-32
PRICKLE2	2.2773795	2.81E-150
RBMS3	2.2773379	1.04E-23
AHNAK	2.2770275	0
RBM47	2.2741605	3.73E-231
LRRC6	2.2730185	0.0000215
MC1R	2.269401	1.12E-22
MTUS1	2.2686374	0
MDH1B	2.2671041	2.08E-06
TRIM29	2.2658941	0.0001398
TTLL13P	2.2653446	0.000753
TDRP	2.2579163	8.7E-68
TGFA	2.2509873	2.65E-70
SLC7A11	2.2497281	5.99E-64
ZNF280A	2.2494156	5.21E-10
GPR132	2.2479275	4.72E-10
TIMP2	2.24708	0
GIP	2.2451125	0.0062896
NKX3-1	2.2446789	4.88E-40
JAKMIP3	2.2441883	1.29E-18
RNF112	2.2439256	0.0014804
CCT6B	2.2428565	3.4E-09
SLFN5	2.2428454	0
KIAA0319	2.2410081	7.44E-25
TBC1D8	2.2397042	1.13E-204
DOK4	2.2396272	2.75E-268
CDRT1	2.236574	3.06E-19
INA	2.2351002	6.63E-187
TOR4A	2.2325166	5.92E-69
TJP3	2.2314234	3.83E-25
AQP7	2.2293789	1.78E-07
PLEC	2.2264887	0
SQSTM1	2.2230029	0
CD151	2.2219927	0
MYO10	2.2205874	0
TNS1	2.2196673	7.12E-136
ARHGEF35	2.2184235	0.0012296
DSTN	2.2179868	0
SLAMF8	2.2172307	0.0006926
ARMCX1	2.2163179	9.06E-06
NPAS2	2.2152541	1.7E-274
IRS1	2.2103599	3.59E-140
NPTXR	2.210275	1.91E-90
TYMP	2.2091963	5.53E-24
ACKR3	2.2083991	5.5E-19
RAB32	2.2078397	1.7E-69
FTL	2.2062578	0
SLC12A7	2.2051986	4.25E-142
HSPA1A	2.2032444	8.38E-83
F2	2.200533	3.48E-06
ITGAM	2.1987799	0.0001142

ZFY	2.1984136	1.44E-17
JUNB	2.1983909	2.12E-178
LTBP2	2.1946474	4.97E-216
RND1	2.1926451	1.32E-55
INAVA	2.1866712	1.68E-194
GJA1	2.1860468	0
ATP6V0D2	2.1832218	0.0077279
VEPH1	2.1810656	0.0001832
KRT8	2.1720248	0
SPDEF	2.1712944	7.46E-10
PLG	2.169925	0.0000796
ADGRG1	2.1680309	8.32E-222
NEBL	2.167799	1.3E-279
SCN1B	2.1668973	3.37E-17
MGAT5B	2.1654404	1.25E-52
AJUBA	2.1649594	0
ITGB6	2.1643868	6.2E-11
C1QL3	2.1604647	0.0081208
CHP1	2.158001	0
SPARC	2.1552404	2.29E-68
FRK	2.1545383	1.9E-88
EPDR1	2.1521523	2.8E-235
KCNB1	2.1509419	8.18E-15
IFNE	2.1497471	0.0006806
ADGRG2	2.1474135	1.14E-39
CAPN8	2.1468414	0.0012483
STX1A	2.1445799	4.46E-84
TCN2	2.1414133	3.7E-26
RASSF2	2.1384356	1.84E-151
AFAP1L2	2.1331711	8.55E-84
RXRA	2.1323171	5.89E-126
NR5A2	2.1312909	5.05E-83
DMRTA1	2.1284746	6.39E-12
PLBD1	2.1243281	6.31E-15
F8A1	2.1192989	3.38E-120
PROC	2.1189411	0.0024748
NCOA3	2.1189205	0
NHS	2.1186749	1.58E-82
GADD45B	2.1182471	1.87E-54
MYL12A	2.117564	0
GLRX	2.116568	5.07E-125
PRKY	2.1154772	0.0090781
SYNM	2.1101512	5.13E-131
BCAR3	2.1100333	3.1E-102
ITGB3	2.1069152	1.4E-13
MT2A	2.1063429	3.28E-139
CDK5R2	2.1057947	0.0021392
TBX19	2.1057947	1.74E-07
HSPA2	2.1055919	2.34E-183
DKK3	2.1048564	9.98E-76
UGT1A9	2.1045988	8.24E-21
TMEM151A	2.101538	0.0000105
PAPLN	2.1004924	2.43E-36
CIB1	2.100254	3.62E-207
C4orf19	2.0995357	0.0034774
CNTNAP3C	2.0994241	4.08E-20
PRR15	2.0982561	3.94E-20
HABP2	2.0968615	0.0044362
AHNAK2	2.0967748	0
MUC5AC	2.0960018	6.95E-49
PLSCR4	2.0941723	1.86E-40
EFHB	2.0939761	0.0046335
IGF1R	2.0933031	0
TBXAS1	2.0910242	4.49E-09
BMP1	2.0900984	6.56E-245
FOSL1	2.0895962	5.3E-31
RAB37	2.0886249	1.73E-25
ZBTB38	2.088223	0

PARD3	2.0869222	0
UNC13D	2.0838316	2.93E-16
RHPN2	2.0811131	0
SH3RF2	2.0802423	1.79E-47
IL1R2	2.0759489	0.0064802
DPP7	2.0735036	2.05E-86
WDR66	2.0730635	1.31E-10
POLD4	2.0713074	2.71E-177
MET	2.0704462	0
SLC19A1	2.0702549	2.36E-51
CLDN2	2.0690416	2.49E-28
POU5F1	2.0676487	0.0001555
GCLM	2.066286	2.87E-226
HILPDA	2.064417	4.06E-147
CCDC80	2.0619401	2.62E-224
ZFP36	2.0616002	1.1E-57
TMOD3	2.0615729	0
RPH3AL	2.0606751	1.55E-65
SLAMF7	2.0551416	1.29E-15
NT5C2	2.0504569	5.32E-289
MMD	2.0468792	2.14E-165
TLR6	2.0448104	1.81E-41
SYNGR3	2.0426564	9.88E-76
RFK	2.0405146	5.31E-279
FIBCD1	2.0400157	2.37E-09
ADAM9	2.0333608	0
CACNA1G	2.0331669	2.52E-42
SOX9	2.0325298	1.19E-61
PRNP	2.0325085	0
TMEM106A	2.0308374	3.77E-11
ULK1	2.0290686	0
NCEH1	2.0288388	1.33E-179
CMIP	2.0272456	1.15E-259
KIRREL2	2.0166787	0.0000194
PIR	2.0160443	1.49E-93
PDE8B	2.0157329	1.53E-43
RAB4B	2.0139298	4.43E-38
MAP1B	2.013881	0
PPEF1	2.0086131	8.44E-21
KLHL5	2.0070233	1.08E-302
MFSD6	2.0069659	1.52E-119
AL034430.2	2.0064454	0.0043237
PLOD2	2.0047078	0
DPY19L2	2.0040928	3.3E-11
TPBG	2.0036512	4.17E-236
FAM173A	2.0022906	8.82E-26
S1PR3	1.9989197	2.71E-138
KCTD9	1.9985829	1.35E-238
MAP7	1.9972554	0
NACC2	1.9965718	4.8E-119
ST3GAL5	1.9952673	1.01E-86
RYR1	1.9933053	0.0009138
SYT1	1.9926015	1.37E-218
SPAG4	1.9922703	2.54E-22
CACNG6	1.991005	7E-91
VASN	1.9868646	1E-26
GAS6	1.9860007	4.15E-19
NAGLU	1.9838195	5.1E-142
PACS2	1.9815302	1.27E-75
PEPD	1.9764035	1.98E-115
TRPM6	1.9763487	9.44E-31
ERAP2	1.974909	1.11E-06
NPR2	1.9743134	2.83E-10
LACTB	1.9717299	1.3E-217
C12orf75	1.9709328	3.06E-300
ITGAL	1.9696264	0.0041602
BCO1	1.9689731	9.56E-11
GDF15	1.9686895	2E-173

PGM2L1	1.9681083	3.25E-119
SIPA1L3	1.9667736	3.61E-268
GRB7	1.9631184	5.06E-18
CAMK2N1	1.9630289	1.29E-99
LEKR1	1.961932	0.0081613
ZP3	1.9614892	1.96E-46
F8	1.959358	3.12E-32
C9orf3	1.9588948	2.17E-57
PCDHGB3	1.9581798	5.56E-08
CCPG1	1.9581782	4.09E-268
ABTB2	1.9576746	5.87E-136
HSD3B7	1.9553173	5.89E-46
MAP3K14	1.9534024	3.86E-81
FAM167A	1.9522825	2.82E-74
RUSC2	1.9518457	1.56E-249
SEL1L3	1.9475514	1.2E-225
GALNT5	1.9464947	1.48E-28
ABCA12	1.944736	2.62E-40
SPTBN1	1.9445426	0
GPC1	1.9437162	9.73E-130
FTH1	1.9411711	0
SLC3A1	1.9409645	2.87E-07
FAM214B	1.9370784	1.61E-78
APH1B	1.9369978	4.85E-109
CCBE1	1.9357079	3.34E-41
DNAAF4	1.9350955	9.67E-26
S100A2	1.9350825	0.0001368
ACTN4	1.9334751	0
TMEM139	1.9323132	0.0001846
IKBKE	1.9311175	5.13E-32
EPS8L2	1.9308853	4.42E-148
AL139011.2	1.9284467	1.35E-07
LITAF	1.9282615	0
RBKS	1.9267811	8.12E-33
TPM4	1.9247955	0
PCDHB2	1.924684	2.41E-21
METRN	1.9246398	2.24E-71
DDX58	1.9211446	5.64E-40
TLR5	1.9179133	5.66E-11
TCEAL3	1.9160153	8.82E-23
RBCK1	1.9139015	0
DPYSL2	1.9136881	0
TRIB3	1.9135286	0
CCDC81	1.9132884	0.0029273
AGRN	1.9122349	0
RASD2	1.9114633	4.2E-17
SRC	1.9113771	4.98E-277
GLUD2	1.9084665	0.000014
ST8SIA4	1.9083471	7.01E-37
SERPINE2	1.9076966	2.02E-226
KIF12	1.9075925	2.68E-09
TMIE	1.9068906	0.0006987
GPR37	1.9035237	1.24E-13
RGL1	1.9032468	2.68E-66
CDC42EP3	1.9029054	1.12E-288
PDE8A	1.8961463	2.06E-234
AC073610.3	1.8938716	0.0001952
TSPAN14	1.8937161	0
MYLK4	1.8845228	0.0023568
SLC7A7	1.88253	2.61E-34
TRAM1	1.8811152	0
GLIS2	1.8794374	8.97E-114
ASPH	1.8789079	0
WWTR1	1.8785882	1.14E-291
JAK1	1.8784557	0
ECM2	1.8773175	9.35E-06
ARL14	1.8768518	0.0060903
QPCT	1.8768071	7.44E-195

ZFAND5	1.8766298	0
AGMAT	1.8749092	2.89E-25
SERPINB1	1.8747966	1.54E-287
MST1R	1.8721459	5.27E-42
CEMIP2	1.8703604	3.65E-292
CTHRC1	1.8655498	0.0034942
HYDIN	1.8650704	0.0036338
CAMK1D	1.8628182	4.54E-196
MMP14	1.8605969	1.05E-06
NDRG1	1.860179	1.81E-73
KLHDC7A	1.8596954	1.31E-16
DDIT4	1.8582383	8.49E-240
KCTD14	1.857981	0.0036338
ITGAV	1.8575169	0
BLVRB	1.8554664	2.3E-242
INCA1	1.8552146	0.0000332
TMBIM1	1.8551507	1.15E-140
ME1	1.8545824	7.58E-256
NR1H2	1.854213	4.72E-116
ABI3BP	1.8530306	1.19E-12
COL20A1	1.8528957	0.0005546
SLC5A10	1.8524428	0.0063969
ANKAR	1.8514775	0.0005818
CTSB	1.8497839	0
CHMP6	1.8484421	2.64E-40
GCLC	1.8477599	0
PPL	1.8466075	2.03E-64
S100A11	1.8437651	0
NAAA	1.8421731	6.22E-37
SLC4A7	1.842012	1.7E-207
SERTAD3	1.8416123	9.14E-89
ZNF285	1.8415991	8.03E-21
NKX2-8	1.8405727	2.05E-07
BMP2	1.8398428	1.17E-13
LMO7	1.839744	1.67E-160
ATP8B3	1.8365013	3.54E-16
SLC35F6	1.8348588	2.58E-277
EPHA2	1.8347245	1.1E-186
SLC45A4	1.8327437	4.93E-96
PRKCA	1.8278736	0
SPATS2	1.8271225	9.3E-253
MAP6	1.8259706	1.19E-07
COL4A4	1.8237494	1.73E-63
ABCA1	1.8215178	8.22E-213
HOGA1	1.8210299	0.0001949
BTBD19	1.8208067	0.0000232
ARL4C	1.820143	8.82E-163
ADAM12	1.8194278	2.06E-14
DNAJB9	1.8190869	3.6E-192
ANKEF1	1.8176352	2.02E-105
IRF9	1.8143108	1.14E-42
FAT1	1.8138209	0
SGCE	1.8133982	1.68E-208
EGLN1	1.8129189	2.86E-289
FSTL3	1.8108619	3.74E-209
UBE2H	1.8101537	8.55E-308
EMP3	1.8083298	1.36E-110
FAM83H	1.8075133	6.39E-143
TNFRSF1A	1.806068	4.41E-249
CASTOR1	1.8056073	2.91E-19
ZFP36L1	1.8049689	0
BTN3A3	1.8042524	2.64E-16
GPRC5B	1.8042517	1.88E-120
BHLHE40	1.7995602	5.16E-214
PYGB	1.7978517	2.43E-299
L1CAM	1.796593	2.34E-51
SPSB1	1.7962973	2.34E-71
LPP	1.7961417	3.28E-208

CCND1	1.7931406	2.55E-273
SP110	1.7925579	2.57E-15
EDEM2	1.7919511	1.11E-124
KRCC1	1.7891184	4.2E-83
HMCN1	1.7875917	5.84E-07
F2RL1	1.7871416	4.73E-148
SH3KBP1	1.7866912	0
CD55	1.7865361	2.15E-303
ABHD4	1.7854352	7.9E-147
ABCB6	1.785154	6.23E-115
SLC25A43	1.7844381	1.26E-129
LMNA	1.7813994	5.78E-268
GPCPD1	1.7809769	1.82E-288
CRISPLD2	1.7783683	1.29E-09
CTSK	1.7770937	6.74E-06
TMSB10	1.7711015	7.74E-47
NEDD4	1.7669355	2.83E-245
AKAP12	1.7664722	1.54E-261
AFAP1	1.7642033	4.97E-272
DIRAS3	1.7640708	0.0033289
GABRB3	1.763747	7.73E-243
SLC9A2	1.7626713	3.31E-10
TMEM265	1.7608486	1.49E-14
RAB38	1.7599196	2.85E-08
KIF13B	1.7595489	6.75E-110
SLC41A2	1.7584556	8.41E-84
HCN4	1.7548875	3.85E-06
PSENEN	1.7536272	1.66E-134
HIF1A	1.7515128	0
LPAR1	1.7506056	6.39E-133
DCDC1	1.7491697	5.8E-46
CACNA1H	1.7469927	7.33E-141
PFKFB3	1.7448323	0
STK32A	1.7441611	1.95E-13
TREX1	1.743952	0.0011561
RGS11	1.7438193	1.63E-07
TGFB1I1	1.7431632	1.21E-88
IFIT3	1.7428422	8.51E-09
TOB1	1.7413348	1.04E-109
IQCN	1.7410817	3.78E-06
CASTOR3	1.7409253	7.76E-53
RTN4	1.7387797	0
ANTXR1	1.7384356	0
TANGO2	1.7379585	5.21E-25
RERG	1.7378872	2.96E-16
PRTG	1.7354867	1.39E-56
PIEZO2	1.7352051	1.31E-48
PLEKHA6	1.7343071	1.74E-89
TMEM51	1.7333723	7.1E-67
EFHD2	1.7330849	1.02E-239
CNTNAP3	1.7329161	1.13E-109
PCDHA10	1.7318039	1.26E-09
IKBKG	1.7317723	3.55E-47
BMPR1B	1.731456	1.81E-79
SLC52A1	1.7299108	0.0000196
TNFSF12	1.7252124	1.57E-15
CACNA1D	1.7248202	1.6E-33
SGK1	1.7247145	3.94E-46
CARD11	1.7243656	0.0001171
GBE1	1.7241429	4.88E-140
ETV1	1.7239877	1.34E-23
HSPA1L	1.7232907	4.02E-07
FRMPD4	1.722466	0.0024578
PAPPA2	1.7198921	3.15E-07
VMP1	1.7172419	0
RIN3	1.716663	6.77E-89
FZD8	1.7164486	6.37E-63
HCFC1R1	1.714938	3.56E-88

FLNA	1.7133763	0
CFAP300	1.7129235	2.78E-28
PIPOX	1.7115959	1.63E-06
EPCAM	1.7103626	3.74E-24
ELMO1	1.7099625	6.22E-36
MYH9	1.7097265	0
ERAP1	1.7092396	4.83E-123
ARHGAP12	1.7091949	1.06E-170
ABHD2	1.7091661	0
RHOF	1.7080409	3.73E-225
MAP2	1.7070233	2.49E-43
MTCL1	1.7065003	5.04E-174
PLPP5	1.7063965	5.9E-73
PGD	1.7048929	0
KDM4B	1.7046693	1.91E-52
SNPH	1.7039191	2.7E-41
RAI14	1.7027332	1.84E-276
HIST1H1C	1.7026966	9.25E-12
FMN1	1.7022002	4.93E-22
PACS1	1.7004689	4.52E-196
SLC6A17	1.7004397	5.52E-07
RINL	1.700039	0.0017924
OSGIN2	1.6990837	9.8E-172
C1RL	1.6975609	2.88E-123
LMO1	1.6964708	0.001955
CEBPB	1.6954293	9.46E-203
KCNS3	1.6911886	2.14E-15
ZFAND2A	1.6911512	8.73E-69
IRF2	1.6896249	2.25E-41
TNFAIP8	1.6895654	2E-111
TNFAIP3	1.6884604	1.1E-95
PIGB	1.686032	4.32E-59
PTP4A1	1.6854766	2.8E-242
ESAM	1.6851267	3.21E-15
QSOX1	1.684491	2.28E-227
FGF2	1.6842828	8.21E-191
PQLC3	1.6838014	3.67E-60
NFKB2	1.6837013	1.73E-111
CBLN3	1.6836965	0.0014587
INPP5A	1.6804986	4.88E-36
PRSS23	1.6779273	0
ELK3	1.6766347	5.28E-189
FAM83G	1.6763665	1.09E-100
ZNF487	1.6750798	0.0000206
KCNMB3	1.6745015	2.63E-06
ITGA5	1.674385	3.09E-231
ANG	1.6721075	1.25E-32
ATP2A3	1.6708403	4.08E-56
FZD2	1.6702375	2.53E-81
ITPRIP	1.6697448	1.54E-149
ZMYND12	1.6690831	4.13E-06
KLF5	1.6638162	3.03E-150
STEAP2	1.6627364	9.6E-136
LDAH	1.6615596	2.83E-54
FBXL17	1.6609114	2.9E-21
MYO5B	1.659159	2.57E-16
WWC1	1.6586628	6.48E-276
SERPINA1	1.6567532	3.06E-07
CDC42SE2	1.6539598	6.1E-191
ID1	1.6522433	1.4E-76
PHYHIP	1.6520767	0.0085279
FSTL4	1.6514712	5.46E-34
FANK1	1.6514142	0.0000275
HTATIP2	1.6510407	4.21E-260
ETHE1	1.6507392	1.6E-62
AL669918.1	1.6479265	1.4E-11
PDGFA	1.6444601	1.05E-62
FTCDNL1	1.6440756	6.18E-09

CHST3	1.6436384	5.61E-140
VEGFC	1.6435373	8.47E-32
C9orf43	1.6418649	0.0018099
GSDME	1.6415528	3.1E-109
SLC2A6	1.6408608	1.01E-24
SLC29A4	1.6398858	2.22E-130
DCLK2	1.6379325	1.77E-29
MTMR11	1.6378372	1.54E-36
LUM	1.6366775	0.0062692
BAIAP2L2	1.6359376	5.3E-16
PTK2B	1.6346344	1.13E-55
IMMP2L	1.6336061	2.79E-14
ECHDC2	1.6320111	3.86E-12
RASGRP3	1.6297504	5.54E-07
UGP2	1.6289757	7.69E-246
FNDC3B	1.6283825	4.97E-298
TMEM187	1.6265698	1.32E-15
NCOR2	1.6265111	0
TCF21	1.6244909	0.0082746
ERP44	1.6221826	2.58E-192
AC007950.1	1.6211796	1.18E-196
AHR	1.6200373	5.97E-128
ARX	1.6189098	0.0014118
ERMP1	1.6188966	2.26E-164
OSR1	1.6185933	6.91E-08
ARHGEF10	1.617354	3.42E-121
GRAMD1A	1.6156314	0
KLF13	1.6150922	4.08E-281
HHIPL1	1.6147098	0.0085361
C6orf223	1.6129769	0.0006714
SDC1	1.6122668	5.65E-263
UPP1	1.6115431	3.45E-47
PLPP1	1.6113683	2.95E-79
B3GALT6	1.6092452	3.59E-47
ZC3H3	1.6080461	3.93E-42
LAT2	1.6074891	3.21E-08
PDGFRL	1.6073355	9.88E-50
COL4A6	1.6070376	8.65E-240
MSRA	1.6061515	1.08E-11
SPATA6L	1.6055434	0.0003278
KCNJ2	1.603341	0.0006193
ABCC1	1.6004948	0
CLMP	1.5998772	1.39E-38
DMGDH	1.599684	0.0001432
TFPI	1.5988808	2.51E-151
ARHGAP26	1.5959385	2.05E-144
SLC2A3	1.5957399	5.57E-21
LRRC32	1.5949122	1.44E-18
SPPL2A	1.5945228	3.68E-142
KCNK1	1.5934276	8.91E-31
WIPI1	1.5933715	3.27E-74
TTLL6	1.5906762	4.37E-20
IDUA	1.5884016	5.86E-15
TMEM87B	1.5880441	4.77E-94
CPQ	1.5849625	1.42E-07
PRX	1.5849625	4.94E-10
IRF7	1.5849625	2.96E-10
CACNB1	1.5842026	1.58E-40
UVSSA	1.5819252	2.83E-67
FO681492.1	1.5815719	8.8E-08
CLIP2	1.5803131	1.09E-131
ADPRHL1	1.580286	1.48E-20
IDNK	1.5765712	3.28E-19
FHL2	1.5746354	1.83E-107
PPP2R2C	1.5744767	8.55E-120
FLRT3	1.5733878	1.72E-46
VASP	1.5716919	5.6E-167
ANXA13	1.569736	0.0000665

HCN2	1.5692489	2.76E-81
RPL22L1	1.5682659	7.14E-51
COMMD10	1.5681815	1.14E-50
MARVELD2	1.5674669	3.05E-45
ACTN1	1.5650262	0
TMEM129	1.5648052	8.02E-34
TRNP1	1.5622765	2.32E-114
SHC1	1.5619062	9.19E-249
KLF4	1.5593227	1.75E-50
RAB40C	1.5558162	9.73E-27
CDH15	1.5547479	3.68E-10
RBM43	1.5546549	3.78E-34
OLFML2A	1.5537536	2.16E-74
SREBF1	1.5533944	4.14E-164
PLEKHG4B	1.5532536	1.89E-07
BFSP1	1.5507555	4.42E-16
ABHD15	1.5507514	4.76E-47
ZNF532	1.5503484	1.87E-142
ECE1	1.5501385	0
MIA2	1.5461079	6.22E-195
MYO15B	1.5460708	1.97E-83
VIL1	1.5459684	7.89E-06
A4GALT	1.545583	3.09E-13
CELSR1	1.5446328	1.59E-204
CACNA1I	1.5443205	0.0012538
CSF1	1.5436788	5.74E-62
JOSD2	1.5422656	1.67E-27
TRIO	1.5422523	6.59E-273
NIM1K	1.5405684	0.0050059
FLRT2	1.5403395	3.25E-22
ARL4A	1.5400665	3.31E-67
SMKR1	1.5379277	6.2E-13
PCBP3	1.537028	0.0034613
PPFIBP2	1.5349542	8.41E-58
LRP10	1.5348862	8.83E-299
ALOX12B	1.5329679	0.0000118
SEL1L	1.5328061	2E-234
PDXK	1.5323804	0
ZNFX1	1.5308648	2.71E-137
SNX33	1.5305717	3.27E-82
TUFT1	1.5297993	4.55E-102
PSME2	1.52896	1.99E-69
TNIP1	1.5280614	2.93E-218
ACSF3	1.5275265	6.62E-21
ZNF160	1.5274115	2.16E-56
ITGB2	1.525091	0.0026924
SMIM14	1.5236457	5.42E-126
CYBC1	1.5227786	4.7E-38
CRB3	1.5221464	0.0000135
TICAM1	1.5220059	3.53E-21
ARHGAP23	1.5217687	1.15E-31
AKT3	1.5206383	4.79E-88
LFNG	1.5206279	3.27E-09
DGKD	1.5205219	6.66E-239
RBPMS	1.520369	8.71E-77
RAB34	1.5198419	1.15E-127
IL13RA1	1.5193927	1.56E-232
TAX1BP3	1.518615	7.73E-94
AMOTL2	1.5179015	6.37E-176
TRIB1	1.5164686	7.68E-108
CPNE7	1.5149734	2.22E-23
MAMDC2	1.5145732	0.009535
C8orf37	1.5145732	4.32E-32
KLF2	1.5143622	2.79E-26
TIGD5	1.5141585	1.02E-15
ABR	1.5129111	1.36E-136
ABHD16B	1.5122934	0.0048901
SDC4	1.51139	4.17E-129

PDLIM5	1.5113367	0
BNIP3L	1.5108301	1.94E-233
KLF6	1.5095227	2.56E-128
PVR	1.5056037	1.66E-180
MYCBPAP	1.504344	3.02E-06
LRRC23	1.503938	6.29E-24
PRKG2	1.5032015	2.09E-19
DLGAP4	1.5030071	1.63E-136
ZMIZ1	1.5005186	8.35E-196
GRAMD1B	1.5000055	1.51E-145
NUP205	-1.501222	1.67E-115
AGPAT4	-1.5025	1.62E-17
CCP110	-1.502554	1.22E-35
FXN	-1.502557	2.48E-28
NEIL1	-1.503433	0.0000306
YLPM1	-1.50369	4.05E-108
PRPF4B	-1.503752	3.28E-74
TLE2	-1.504459	1.98E-18
ZYG11A	-1.504883	8.47E-17
GNL2	-1.50557	1.4E-51
AGTPBP1	-1.505818	8.13E-69
GPLD1	-1.507402	7.03E-06
DZIP1	-1.507962	6.66E-63
SLC44A2	-1.508298	1.11E-47
TSPAN18	-1.508697	2.74E-23
MIGA2	-1.509069	1.38E-14
RFC4	-1.509665	1.1E-27
CEP131	-1.510195	4.87E-20
VARS2	-1.510713	3.54E-28
BTBD2	-1.512077	1.09E-72
KLHL11	-1.512154	3.68E-41
C9orf40	-1.512167	6.73E-18
ZBTB40	-1.51315	1.82E-45
RNF123	-1.513444	9.04E-36
FMO4	-1.514573	0.0014613
ZNF397	-1.514679	8.94E-41
EPPK1	-1.515838	2.78E-07
RIOK1	-1.516726	2.86E-39
ILF3	-1.516889	1.25E-145
ZSWIM5	-1.517653	5.67E-10
STK25	-1.518772	1.07E-65
USP28	-1.519778	1.77E-44
NDRG4	-1.519967	4.96E-25
CDCA3	-1.520394	3.82E-12
EPS15L1	-1.520607	1.13E-34
CHDH	-1.520781	1.28E-15
KHDRBS1	-1.520802	2.71E-118
UCK1	-1.521237	1.07E-38
DEPDC1B	-1.521263	1.73E-25
NUSAP1	-1.521443	1.79E-86
NOL9	-1.523695	2.68E-39
STAG1	-1.524077	1.41E-35
POLDIP3	-1.524444	2.13E-80
H2AFX	-1.525281	8.56E-30
PDIK1L	-1.52544	7.35E-19
LIMD2	-1.525803	8.93E-18
HDHD2	-1.526044	6.79E-23
HNRNPH2	-1.526458	1.27E-83
RPS4X	-1.526574	0
FAXC	-1.526657	1.55E-08
RAP2A	-1.526691	5.28E-56
ZC3H13	-1.526926	1.07E-39
ZFC3H1	-1.527107	4.75E-65
PLXNC1	-1.527247	2.46E-06
UBE2G2	-1.527362	1.78E-87
TMEM237	-1.528188	1.16E-48
TPH1	-1.52878	1.06E-08
LCAT	-1.528792	0.0032924

CHST2	-1.528928	0.0000417
LIMK2	-1.53064	7E-17
UBE2C	-1.530687	2.86E-49
NUDT3	-1.530987	3.92E-116
TRIM36	-1.531613	6.96E-11
ANP32E	-1.531937	1.31E-72
TROAP	-1.533413	2E-41
ATPAF1	-1.5355	2.23E-92
TRIM33	-1.536387	1E-94
KIAA1328	-1.536917	2.92E-22
GPX3	-1.537151	2.58E-18
ATG4D	-1.537406	1.38E-23
MTR	-1.537442	8.17E-107
PAXIP1	-1.538258	6.35E-48
PRPS1	-1.538367	1.9E-81
CCDC77	-1.539529	2.5E-18
KCNK5	-1.539733	2.25E-07
SEMA3E	-1.53981	0.000101
LSM7	-1.540231	1.74E-15
IFNLR1	-1.540444	3.1E-15
RBMX	-1.540836	1.31E-100
CLPB	-1.541574	3.85E-58
CXADR	-1.542127	3.59E-48
SLC30A3	-1.542149	2.39E-07
SLC43A1	-1.542361	1.19E-25
RRM1	-1.542644	2.31E-113
CPEB3	-1.543235	3.37E-09
SYNJ1	-1.543522	1.21E-37
AC244197.3	-1.543942	4.68E-41
AMIGO1	-1.545792	2.26E-10
POLR2D	-1.545934	1.16E-47
SGK3	-1.545984	2.12E-25
RNF8	-1.546044	1.22E-28
IQGAP3	-1.546228	4.09E-38
LSS	-1.547699	3.1E-35
TRIP13	-1.549134	2.21E-40
HAUS1	-1.549984	2.58E-38
FRY	-1.550197	4.2E-07
GNG4	-1.550641	2.02E-38
ISY1	-1.551172	9.61E-31
SLC25A11	-1.552152	2.99E-60
UBAP2L	-1.555155	9.92E-108
HSF4	-1.555471	0.007755
H2AFZ	-1.556637	2.26E-116
CYP2U1	-1.556993	2.56E-16
LRRC4B	-1.557109	1.61E-16
POLR3A	-1.557838	1.19E-85
USP18	-1.557995	4.26E-18
C2CD2L	-1.558873	1.45E-16
BRMS1L	-1.560604	1.9E-34
FAM216A	-1.561988	1.37E-36
ZUP1	-1.562106	1.45E-18
ZNF764	-1.562263	5.26E-17
PROSER1	-1.562659	2.77E-64
RGPD3	-1.562936	0.0061288
ZNF680	-1.563195	2.81E-12
KLF12	-1.564217	1.65E-32
ZFHX4	-1.565005	1.52E-61
ZNF891	-1.565999	5.86E-50
ZNF445	-1.566347	7.61E-70
FUZ	-1.566719	7.17E-35
DOCK11	-1.567773	6.12E-30
KITLG	-1.568432	7.91E-28
NUTM2A	-1.570749	0.0031896
RBM12	-1.571116	3.23E-99
PEF1	-1.571373	4.56E-66
SLC2A12	-1.571867	3.47E-12
LRRC3	-1.572829	1.24E-09

STK26	-1.573589	2.4E-50
ZRANB3	-1.573603	1.19E-26
POMK	-1.574869	1.11E-11
GCDH	-1.574919	6.56E-11
THAP9	-1.575039	1.9E-12
LBR	-1.575817	3.97E-119
GSTM3	-1.576488	6.55E-25
LARS2	-1.576552	1.09E-36
PXDN	-1.57656	4.61E-72
INTU	-1.576857	8.73E-10
GK	-1.576918	2.83E-34
CSTF2	-1.57719	1.47E-29
FAM221A	-1.57782	0.0001782
SKA2	-1.577897	2.32E-62
ATG4C	-1.578704	4.11E-29
44447	-1.579616	5.13E-90
SMPDL3B	-1.579632	1.77E-09
BSDC1	-1.579934	1.59E-74
ASB6	-1.580185	3.75E-24
PLAGL2	-1.58105	6.72E-50
RFC5	-1.581165	6.24E-80
PPFIA4	-1.581201	3.88E-10
KLHL22	-1.581807	4.17E-29
ZNF850	-1.582258	9.13E-12
FOXRED1	-1.582652	3.33E-29
RNF219	-1.583319	1.91E-33
PRKAA2	-1.58404	7.7E-61
CENPT	-1.584208	3.25E-29
RRP1	-1.584382	1.49E-30
RFTN2	-1.584963	0.0055268
CHRNA7	-1.584963	1.69E-08
ATG4A	-1.584963	8.93E-34
PCNA	-1.586766	5.38E-120
MSH2	-1.586952	2.19E-76
TMEM171	-1.587505	0.0052684
TMED8	-1.588221	2.14E-99
PSMG1	-1.5888	2.1E-51
SNRNP25	-1.589265	2.74E-16
RRP1B	-1.589356	5.19E-126
CHD4	-1.58966	1.27E-185
RAVER1	-1.590282	1.08E-67
KDM8	-1.590693	6.23E-07
SIGMAR1	-1.590729	1.9E-148
KRBA1	-1.591369	1.18E-17
LLGL1	-1.591825	4.98E-56
THAP7	-1.591835	8.73E-20
LBX2	-1.592267	0.0000584
SPINDOC	-1.592423	1.75E-39
GUCA1B	-1.592457	0.0013031
SLC5A5	-1.594549	0.0000233
VANGL2	-1.594958	1.11E-30
CLDN7	-1.594999	0.000228
TBKBP1	-1.5954	1.5E-25
CAMSAP3	-1.596052	7.1E-17
ZNF232	-1.596146	1.25E-08
VAX2	-1.596234	0.0009826
ACIN1	-1.59638	9.26E-143
TIMM29	-1.597958	2.38E-13
ARID5A	-1.598637	9.29E-10
PKNOX1	-1.599804	1.43E-20
UROD	-1.600479	4.43E-61
CFDP1	-1.601197	2.2E-62
PPP2R5D	-1.601554	8.09E-45
FRAT2	-1.601772	2.12E-22
CENPP	-1.602214	2.1E-20
TMPO	-1.602845	2.28E-162
SPOUT1	-1.603817	3.31E-21
HNRNPM	-1.60391	7.84E-153

ATP2A1	-1.605543	0.0067869
IGFBP2	-1.606063	6.38E-34
POLR3K	-1.606266	3.13E-19
STRIP2	-1.606989	1.17E-16
GLO1	-1.607193	8.39E-187
TIMM13	-1.608237	2.57E-42
ZNF747	-1.609449	7.5E-30
SECISBP2	-1.609501	1.79E-58
UBAC1	-1.610116	1.03E-46
ZNF616	-1.610155	2.7E-14
DFFB	-1.610225	1.71E-11
GSTA4	-1.610719	1.54E-34
CEP85L	-1.61094	3.11E-28
USP46	-1.611332	1.22E-52
RPS15	-1.61157	2.36E-133
SH3BGR	-1.611999	0.0002087
PDS5B	-1.612031	1.37E-67
BTBD6	-1.612032	1.4E-50
MCM7	-1.612236	4.33E-201
ASS1	-1.613062	1.49E-103
C11orf71	-1.614753	3.57E-11
TXNIP	-1.615769	0.0000602
ACADM	-1.616086	2.04E-78
CEP57	-1.616587	2.25E-68
MAPK3	-1.616593	7.84E-66
MAN1C1	-1.616821	1.48E-08
DEPDC4	-1.617075	0.0005272
KIAA0586	-1.617526	2.87E-42
ULBP2	-1.618498	2.65E-10
C11orf95	-1.61851	5.34E-30
NEK4	-1.618578	1.93E-41
MTFR2	-1.619959	1.74E-14
CENPC	-1.620152	4.15E-21
GPRIN1	-1.620524	4.32E-27
NPL	-1.620566	1.29E-09
PDE3B	-1.622437	1.16E-12
PITHD1	-1.622717	2.02E-38
AMER1	-1.622809	9.3E-32
RGS14	-1.623309	0.0000101
LBHD1	-1.623775	1.17E-15
DLGAP5	-1.623812	2.11E-46
RPRD1A	-1.623928	2.8E-149
FAM76A	-1.624664	1.48E-20
ZNF571	-1.624901	7.73E-08
SRSF12	-1.625073	1.08E-12
CCDC14	-1.625197	1.21E-68
FBF1	-1.625456	6.23E-35
PYGL	-1.62584	1.19E-138
ZNF362	-1.626011	1.37E-34
ADAMTS13	-1.626741	6.77E-12
AGAP3	-1.627137	1.77E-44
ZMYND19	-1.628913	8.51E-30
PRDM16	-1.629162	0.0000131
INCENP	-1.62945	3.76E-75
SLBP	-1.629769	2.56E-95
GNB3	-1.630286	0.0035758
PRRC2A	-1.631541	5.41E-188
MINDY1	-1.631625	3.89E-19
ACSS1	-1.632087	1.03E-33
KCNAB3	-1.632561	0.0000971
TUBA1B	-1.632813	1.44E-137
PPM1D	-1.633218	4.48E-69
DCXR	-1.634014	2.51E-22
PKIB	-1.634023	3.92E-08
MTHFD1	-1.63509	2.64E-190
SLITRK5	-1.635447	7.59E-37
BHLHB9	-1.635589	4.49E-25
SC5D	-1.636033	9.08E-104

SMC2	-1.636107	2.64E-68
CCDC180	-1.636564	1.75E-10
TUBB4A	-1.637308	2.29E-31
HIST3H2BB	-1.638901	0.0019572
RBP1	-1.639885	3.66E-06
NUPL2	-1.640884	3.19E-22
FBXO48	-1.641231	2.51E-09
USP51	-1.641993	2.84E-07
SLC25A1	-1.642087	4.25E-46
BCL11B	-1.643144	2.62E-07
ZNF77	-1.645107	4.36E-08
ZSCAN30	-1.647413	1.97E-48
DHX30	-1.648174	5.93E-101
POP1	-1.64831	4.65E-19
ARHGDIG	-1.649418	0.0000757
NEK10	-1.649451	0.0001822
HMGN3	-1.649748	2.62E-28
PLCG1	-1.649905	3.09E-98
FARSA	-1.650314	1.19E-108
SPDYE2	-1.650339	0.0044789
SHOX2	-1.650523	1.83E-06
CREB5	-1.650551	4.69E-37
NAA80	-1.651984	3.5E-09
EXOSC10	-1.652167	2.45E-92
USP44	-1.652251	1.73E-07
MOXD1	-1.654262	5.34E-29
AC234031.1	-1.654681	0.0013656
ING3	-1.655773	2.96E-24
UAP1L1	-1.656442	7.69E-28
SCML2	-1.658824	6.02E-49
GAB1	-1.659586	4.89E-23
CTXN1	-1.659891	2.06E-12
SMARCB1	-1.66051	1.11E-115
TCTA	-1.660712	8.64E-31
RPL36A-HNRNPH2	-1.66095	0.0002679
TLE3	-1.661292	6.12E-50
PRIM2	-1.661776	2.89E-35
SOX21	-1.662071	7.41E-08
TTC12	-1.662098	1.04E-14
OXCT1	-1.663527	1.17E-29
HDAC5	-1.663577	8.46E-47
SGO2	-1.663921	1.67E-25
FAM229B	-1.66427	9.09E-18
BRCA1	-1.664424	1.47E-98
ETFDH	-1.664756	9.53E-34
HAPLN3	-1.665047	6.57E-16
BCHE	-1.665511	3.52E-15
RBM14	-1.666454	4.83E-54
DOK1	-1.666976	1.82E-13
THEM6	-1.667029	4.73E-26
HMGN1	-1.667575	2.03E-137
TAF11	-1.66796	1.87E-38
TIPIN	-1.670275	2.09E-46
TBC1D4	-1.670825	6.92E-87
ALG10B	-1.672087	6.77E-85
EPHA4	-1.672462	5.79E-24
SETD1A	-1.673883	7.56E-93
CHEK1	-1.675126	2.88E-57
FAM72B	-1.675415	7.45E-20
ARHGAP33	-1.675475	0.0000198
IPO5	-1.676093	1.51E-253
GINS3	-1.676307	1.97E-39
BCL7A	-1.676811	1.41E-48
HK2	-1.678015	2E-48
GPR180	-1.678159	3.12E-66
MON1A	-1.678226	6.7E-12
RPAP1	-1.678424	1.93E-45
UBE2T	-1.678706	2.07E-40

ZNF689	-1.679414	4.74E-39
RALGDS	-1.679995	2.27E-59
LIN9	-1.680425	3.83E-39
KIF11	-1.681818	2.34E-97
CDCA8	-1.683479	4.94E-30
CSPG4	-1.684843	5.27E-08
KCNMB4	-1.685707	8.44E-11
ABCC5	-1.685779	7.94E-60
ADGRL1	-1.686144	2.89E-115
HECA	-1.687095	1.5E-46
SORBS1	-1.687304	4.43E-17
FAM102B	-1.688282	4.88E-44
DDAH1	-1.688381	4.79E-70
SWSAP1	-1.689001	0.0000409
CCDC22	-1.689515	3.9E-26
SPDYE6	-1.689551	0.0028495
C1GALT1C1L	-1.689932	0.0048853
SSX2IP	-1.690099	2.94E-56
ZIC5	-1.691436	2.33E-24
TNPO2	-1.692662	2.76E-66
MRPL37	-1.692773	7.43E-144
ANGEL1	-1.693252	1.21E-51
SMAP2	-1.693908	1.31E-57
SLC25A19	-1.6942	7.46E-18
CHAF1A	-1.695062	3.57E-39
ZNF169	-1.69555	3.04E-15
PLEKHJ1	-1.695669	2.57E-27
TMEM63C	-1.695847	6.6E-09
ZBTB48	-1.696128	5.85E-16
PDE5A	-1.697464	4.92E-10
PGAP1	-1.698315	6.83E-60
LHFPL5	-1.698344	1.81E-06
HAT1	-1.698928	6.5E-73
RPAP2	-1.699544	2.89E-51
TSPYL2	-1.699642	1.82E-52
DVL2	-1.700123	1.75E-75
EBLN2	-1.70044	0.0000802
PNCK	-1.701238	0.0005647
CCDC66	-1.701393	5.75E-21
LHX2	-1.702614	1.31E-09
HES6	-1.70275	2.09E-09
ZNF684	-1.702799	3.42E-08
SYNE2	-1.703948	3.82E-48
TBXA2R	-1.70648	0.0005986
PPM1J	-1.706661	0.0001108
NUDT12	-1.706739	1.06E-22
TNRC6C	-1.706821	5.04E-17
TUBGCP4	-1.707046	2.27E-69
ANKRD18B	-1.708446	2.55E-18
TTLL11	-1.712168	1.88E-11
SEC31B	-1.712606	1.84E-20
TMEM173	-1.712931	0.0001992
ALMS1	-1.713845	3.48E-37
NOS1AP	-1.71473	0.0011193
TACC3	-1.716462	3.07E-63
SYDE2	-1.716467	1.97E-13
TTLL12	-1.716965	6.65E-57
SMIM8	-1.717027	8.58E-06
MCOLN2	-1.717284	4.54E-15
ENPP1	-1.718753	6.97E-54
CCDC142	-1.718926	6.05E-16
SMARCD3	-1.719187	1.3E-37
MUTYH	-1.719779	1.02E-21
CALCRL	-1.720846	5.16E-10
HSPA4L	-1.72187	1.26E-88
VAX1	-1.722057	0.0005609
POLR3B	-1.722364	3.36E-46
SYNGAP1	-1.723259	1.08E-34

OMA1	-1.723526	2.8E-24
POLQ	-1.724726	8.57E-44
ZNF346	-1.724784	1.65E-57
ST6GALNAC6	-1.7266	9.09E-50
ARHGAP19	-1.72661	1.11E-63
C8orf44	-1.726715	0.0003153
PBRM1	-1.728617	2.87E-75
RAD54L2	-1.73003	1.4E-66
TTK	-1.730256	7.37E-80
SCAI	-1.730302	9.66E-43
GCH1	-1.730377	1.34E-30
PCSK6	-1.730494	5.63E-32
PCGF6	-1.731183	9.66E-42
FNIP2	-1.732232	1.48E-25
ALKAL1	-1.732304	0.0032664
MAP4K1	-1.732896	0.0014792
MYSM1	-1.734842	5.18E-67
PDZD4	-1.736388	1.09E-10
PPP1R8	-1.73671	5.63E-90
PLD4	-1.736966	0.004118
STARD9	-1.736966	6.65E-27
KATNA1	-1.737327	4.67E-33
FRMD4B	-1.738018	6.17E-16
LYAR	-1.738072	1.44E-49
HDX	-1.738177	2.39E-18
GATD1	-1.738636	3.04E-41
RMI2	-1.740964	5.68E-34
ATAD2	-1.741354	1.24E-81
PLCH2	-1.741467	0.000481
JAG2	-1.741964	2.88E-71
NKX2-5	-1.744343	9.93E-32
ADORA2A	-1.744482	3.26E-07
CDC5L	-1.745533	6.53E-113
SESN2	-1.746565	1.85E-90
PIK3R3	-1.748098	2.18E-06
ZNF74	-1.748105	3.16E-29
PRADC1	-1.748117	3.76E-09
TIMELESS	-1.748198	9.69E-155
SRD5A3	-1.752341	1.63E-63
SNRNP40	-1.753319	1.92E-58
SIX1	-1.755234	1.8E-33
PAGR1	-1.755574	6.13E-76
ALDH6A1	-1.755622	2.1E-67
TDP1	-1.756576	1.38E-28
SLC25A33	-1.75741	4.04E-40
KCNC3	-1.757557	1.25E-09
HOMEZ	-1.757691	4.2E-30
DOP1A	-1.757847	6.39E-40
PPIF	-1.758282	6.57E-80
PPIG	-1.758587	1.11E-79
SURF2	-1.758787	4.82E-25
NUF2	-1.759368	2.61E-28
SAMD14	-1.761061	0.0000187
SRBD1	-1.76116	2.21E-63
S1PR5	-1.763513	4.59E-06
ZNF721	-1.764116	3.56E-54
ALX3	-1.764398	0.0000104
CRTC1	-1.76513	6.71E-33
KDELC1	-1.765217	2.45E-28
BUB1	-1.765504	4.97E-71
MRE11	-1.766438	8.84E-124
GPATCH11	-1.766752	6.18E-42
SRSF4	-1.766777	3.82E-103
SIX4	-1.767237	1.21E-47
INAFM2	-1.772984	2.54E-18
SMARCC1	-1.773278	1.33E-198
KIF22	-1.773623	3.1E-60
COLEC11	-1.775616	0.0005877

MMS22L	-1.776316	9.82E-28
TMEM131L	-1.776895	3.03E-49
CAPN3	-1.777061	0.0002223
SH3D21	-1.777608	1.74E-06
PPIL4	-1.777882	1.93E-63
EFCAB7	-1.7784	2.18E-20
44443	-1.780434	0.0007211
INPP5J	-1.78136	1.37E-11
RNF157	-1.781389	5.62E-43
CHAC2	-1.783581	8.23E-15
ATRIP	-1.785592	1.78E-20
FKBP5	-1.786455	3.93E-92
SH3BGRL2	-1.787085	2.26E-32
ZC4H2	-1.787585	4.89E-40
VRK1	-1.789094	2.99E-79
PDSS1	-1.790139	2.41E-25
AKAP5	-1.790393	1.18E-15
DNM3	-1.790711	0.0000462
MAGED4B	-1.791973	3.09E-46
ISYNA1	-1.792926	1.14E-69
CDCA7	-1.795141	8.38E-99
TYMS	-1.795244	3.77E-111
RYR2	-1.795529	4.29E-13
SDE2	-1.79697	3.35E-109
44445	-1.79801	3.05E-84
VASH2	-1.798366	4.56E-09
SCN2A	-1.79862	1.4E-09
CCDC120	-1.79886	2.56E-20
GPR3	-1.799886	0.0054063
ARMC6	-1.799994	4.48E-51
UPF1	-1.800515	5.51E-132
ZNF620	-1.801619	1.15E-20
PRPF39	-1.801864	5.08E-46
MCM5	-1.80188	9.65E-87
KIF20B	-1.802101	5.19E-44
ZDBF2	-1.802159	1.23E-72
EZH2	-1.803996	1.82E-80
SLC18B1	-1.804031	4.57E-60
BRPF3	-1.804731	1.35E-50
KLHL15	-1.80496	9.44E-76
NUP188	-1.80517	7.18E-155
SETD6	-1.805871	5.96E-36
ADAMTSL1	-1.807355	0.0021608
PAN2	-1.808093	2.43E-84
MYCL	-1.810428	1.87E-10
TSNAXIP1	-1.81139	0.0000809
LEPR	-1.811822	3.95E-31
CHD7	-1.815661	7.34E-83
SAMHD1	-1.816921	1.08E-79
UNG	-1.818073	9.17E-93
WNT5A	-1.818926	9.1E-34
NFRKB	-1.819576	3.51E-61
MANEA	-1.820021	2.54E-37
SNX10	-1.820461	4.41E-23
PRDM13	-1.823122	0.0078502
HOXD3	-1.823367	0.0000137
GPR155	-1.824041	1.22E-18
GTPBP1	-1.825181	1.12E-64
TUBG1	-1.8258	1.38E-92
C1orf35	-1.826018	6.45E-40
UBR7	-1.826769	8.86E-108
ZNF823	-1.82697	1.32E-06
GMPR	-1.827066	0.0010223
WSCD1	-1.827298	2.19E-21
HOXB9	-1.828714	1.44E-80
EIF4EBP2	-1.829338	1.96E-168
SPDYE16	-1.8296	4.62E-11
DUSP15	-1.830286	0.0001241

IRX3	-1.830489	1.08E-11
WASF1	-1.831154	1.16E-89
ATP6V0E2	-1.831455	1.29E-52
NXPH4	-1.831733	2.86E-18
ZNF675	-1.832368	1.28E-16
MYOM2	-1.832412	3.68E-17
RANBP1	-1.832643	7.27E-136
TBX20	-1.833642	4.42E-06
FOXM1	-1.834816	1.18E-118
TIMM8A	-1.835029	7.68E-36
DFFA	-1.83582	2.14E-173
MTBP	-1.838098	2.38E-22
MYCBP2	-1.838279	2.02E-170
RUFY3	-1.838456	9.64E-62
ZNF92	-1.839597	4.32E-33
PCOLCE	-1.840582	1.79E-09
TIMP3	-1.84081	0.0021938
PIBF1	-1.841663	7.41E-28
MARS2	-1.842038	6.89E-33
GMNN	-1.845158	6.87E-56
AKR7A2	-1.846131	1.41E-52
SPATA33	-1.846401	3.65E-14
CCNI2	-1.846455	0.006736
AP1S2	-1.846629	1.4E-87
CEP295	-1.848315	4.47E-43
DCAF15	-1.850084	1.11E-37
CD302	-1.850634	5.85E-26
ENG	-1.853779	0.0004933
RAD51C	-1.853973	1.05E-46
SARM1	-1.854052	2.11E-29
DIP2A	-1.854605	2.74E-51
SPOCK3	-1.855052	0.0000596
SSC4D	-1.855816	0.0001872
ZNF114	-1.856443	4.51E-16
ZNF691	-1.856511	2.59E-21
TUB	-1.858276	1.01E-79
SCAF4	-1.859186	2.4E-97
IL11RA	-1.85947	1.8E-10
PITX3	-1.859822	0.0071839
ABHD14A	-1.860725	4.03E-17
BBS7	-1.863151	4.1E-59
OSGEP	-1.863284	1.11E-25
KCNC4	-1.86384	9.75E-67
ITGA7	-1.864405	1.37E-24
PPARGC1B	-1.865699	3.19E-47
ATP5IF1	-1.866614	2.3E-89
PSRC1	-1.867272	8.12E-20
CROCC	-1.86755	5.49E-21
PLEKHG5	-1.867943	3.89E-20
ZMYM1	-1.868092	7.51E-53
SESN3	-1.868175	6.71E-38
DDI2	-1.869895	3.64E-97
ATG16L2	-1.870871	1.14E-14
TMUB1	-1.871146	4.13E-77
PI4KA	-1.872062	3.47E-166
ZNF768	-1.872897	2.1E-120
TELO2	-1.872913	3.51E-47
GGT7	-1.87457	2.04E-36
EXOSC9	-1.87457	2.32E-67
CSNK1E	-1.874775	1.79E-199
ZNF431	-1.874916	1.3E-39
MAP6D1	-1.874987	1.69E-07
EMILIN2	-1.875024	1.39E-36
DDX11	-1.875026	1.47E-111
KANSL1L	-1.875151	4.8E-16
RIMKLA	-1.876729	0.0000131
NPAS1	-1.877618	1.84E-26
WDR77	-1.880472	1.16E-103

GLMN	-1.88148	2.4E-23
RSRP1	-1.881658	7.72E-26
ACE	-1.883104	4.31E-12
EN1	-1.883437	3.94E-10
CFAP298	-1.883562	6.9E-29
MSX2	-1.886688	1.93E-35
ESS2	-1.886779	5.01E-39
HSF2	-1.886855	5.42E-79
KIAA1586	-1.88705	8.09E-30
FUS	-1.887268	1.1E-120
TOPBP1	-1.888858	1.19E-134
C17orf53	-1.890522	1.05E-17
AC008736.1	-1.892017	0.0000292
SMC1A	-1.892155	0
LRIG2	-1.893594	1.02E-55
PIDD1	-1.893609	5.78E-44
BIVM	-1.894103	2.85E-42
POLH	-1.894419	5E-68
EMB	-1.895871	6.41E-76
FRRS1	-1.896041	2.58E-17
GPRASP2	-1.89683	8.06E-81
MEIS1	-1.898808	3.48E-22
SYNGR1	-1.899846	8.09E-41
FAM120C	-1.900028	4.52E-07
MED26	-1.900464	7.67E-20
GREB1	-1.900803	1.15E-40
RBM26	-1.901611	1.26E-156
PRPF38B	-1.902039	2.71E-107
SHISA7	-1.902703	0.0003556
HHLA3	-1.90277	1.87E-11
DNAJC6	-1.903182	2.8E-34
RBM20	-1.903686	1.75E-58
CCNA2	-1.905264	3.46E-94
TGIF2	-1.90534	2.79E-52
CGN	-1.906372	8.74E-39
TMEM39B	-1.907418	8.5E-38
PRRT3	-1.907855	3.58E-07
GP1BB	-1.90837	0.0000153
CHEK2	-1.90886	1.44E-28
NRXN3	-1.910203	0.0003885
ANKS6	-1.910258	1.21E-83
PRPF38A	-1.910777	1.6E-100
ZNF490	-1.912257	6.58E-15
KREMEN2	-1.913447	2.56E-09
MEX3A	-1.913792	3.86E-37
MZT1	-1.914162	1.03E-62
CT45A10	-1.914472	0.0002119
SAMD1	-1.914531	1.03E-38
RNF208	-1.918793	6.88E-06
FBXO5	-1.918962	1.8E-61
NCBP3	-1.919631	1.26E-27
TTYH2	-1.920666	3.1E-25
DIS3	-1.920772	8.2E-150
ALG6	-1.920916	1.66E-65
FMC1	-1.921446	3.4E-10
SPEN	-1.921744	7.25E-199
SMPD3	-1.921997	0.0029001
PPFIA3	-1.923655	5.97E-36
CLK2	-1.923773	8.16E-90
SHLD3	-1.924179	4.31E-12
B3GNT4	-1.924273	0.0001139
ZEB2	-1.925804	2.48E-33
CENPM	-1.926491	7.67E-27
ARHGEF26	-1.926729	1.6E-34
NPNT	-1.928643	1.45E-21
ANKRD36C	-1.931717	5.77E-33
MARK1	-1.931919	1.52E-20
ZNF829	-1.934905	2.84E-10

STIL	-1.934963	3.52E-46
ZFP69B	-1.93587	1.4E-10
HPSE	-1.936748	2.19E-18
ZNF10	-1.937117	1.79E-19
KLHL17	-1.937309	4.76E-18
CDS1	-1.937745	2.16E-20
LRRC40	-1.937762	1.99E-85
C4orf46	-1.937911	6.64E-69
GAS7	-1.937939	4.91E-12
MCM9	-1.93823	2.06E-31
DCP2	-1.939575	6.05E-156
LIG1	-1.940641	3.38E-112
FOXO6	-1.940761	0.0034728
ANO7	-1.941583	0.0003248
AARS2	-1.942527	3.69E-49
CCDC74A	-1.943889	4.68E-22
LIPE	-1.943966	9.13E-06
KAZN	-1.944413	1.9E-16
HOXA4	-1.944727	3.91E-06
KNL1	-1.944732	2.58E-64
BMF	-1.947653	2.64E-26
ATAT1	-1.948677	1.87E-32
REEP2	-1.949379	2.02E-56
LMNB2	-1.949557	5.91E-138
PPAT	-1.950998	3.89E-133
SLC17A7	-1.952047	1.83E-07
PLA2G6	-1.952732	2.04E-21
CBX2	-1.953729	7.45E-79
SAPCD1	-1.957772	0.0000736
CPE	-1.958904	9.93E-81
POLD1	-1.959824	4.25E-128
BORA	-1.960472	1.22E-31
DYNC2H1	-1.960745	2.11E-145
NOTCH4	-1.961526	0.005559
RPS6KA5	-1.96197	2.08E-42
CCNF	-1.963202	2.15E-53
C1orf52	-1.963719	1.01E-18
NAALAD2	-1.964896	0.0009612
KHSRP	-1.96637	7.42E-238
MDN1	-1.970094	2.7E-220
PHGDH	-1.970951	2.82E-232
CENPJ	-1.972714	5.48E-37
SNCG	-1.972765	0.0004876
RCC1	-1.972833	2.39E-172
GMEB1	-1.972862	1.38E-30
USP37	-1.973478	1.53E-76
KYAT1	-1.976688	2.68E-24
FBLN5	-1.979686	1.21E-12
DHCR24	-1.980699	2.48E-108
GPR162	-1.981668	0.0000378
HLA-B	-1.982471	2.05E-94
MIS18A	-1.985224	3.18E-47
KIF7	-1.985798	4.96E-36
CCDC117	-1.987205	9.42E-113
SPTBN2	-1.988763	6.38E-79
CKAP2L	-1.989209	4.41E-40
GATA4	-1.989324	5.85E-40
IFI30	-1.990734	1.35E-36
WHRN	-1.991233	9.81E-30
XKR8	-1.991416	1.44E-24
TRPV1	-1.992797	5.42E-46
RADIL	-1.992826	2.15E-16
MERTK	-1.994402	5.89E-34
CACNA1C	-2	0.0000169
FEN1	-2.001091	1.89E-86
FAM210B	-2.002733	1.08E-81
ZNF711	-2.002763	7.57E-59
ACAP3	-2.004432	2.34E-36

FGF19	-2.005538	0.0044979
SH3PXD2A	-2.005688	1.42E-62
MAOB	-2.007342	0.002256
WNT11	-2.010608	4.69E-08
PCGF3	-2.011924	1.86E-125
FYN	-2.012744	7.8E-80
RRAGD	-2.013087	3.38E-44
CD83	-2.013632	2.32E-13
HAUS8	-2.01495	1.32E-25
EXOG	-2.015212	1.32E-19
HASPIN	-2.016629	3.85E-40
LUC7L3	-2.017882	4.48E-207
TUBB2B	-2.017897	2.79E-77
MTF2	-2.018176	2.28E-86
MAMSTR	-2.019176	2.21E-08
TMSB15B	-2.019744	3.48E-07
APLN	-2.020272	0.0004355
CEP128	-2.02084	5.87E-36
PRR14	-2.024634	8.94E-96
PHF8	-2.0252	5.39E-100
FAM171A2	-2.025588	1.13E-24
LRP1B	-2.0268	2.78E-11
TRAM1L1	-2.027136	3.9E-11
EFNB3	-2.027273	4.13E-21
CDH12	-2.028569	0.0053098
C5orf34	-2.028672	2.48E-21
DDX39A	-2.02909	4.44E-80
CDC45	-2.029805	4.28E-50
ZNF107	-2.032941	3.46E-51
CHST6	-2.033167	0.0006626
TMEM201	-2.033211	2E-56
PCLO	-2.034126	1.34E-37
RFC3	-2.035118	4.74E-103
INSYN1	-2.035908	2.1E-11
NDRG2	-2.040549	2.65E-29
RAD51	-2.040908	2.2E-31
OAF	-2.042945	4.14E-40
ADAMTS4	-2.044394	0.0021598
KIF2C	-2.044808	6.24E-70
TBX6	-2.044921	2.51E-06
NKD1	-2.046294	9.89E-21
FKBP1B	-2.04684	3.65E-12
ZSCAN18	-2.047418	9.39E-21
RBBP4	-2.047909	0
BACE1	-2.048154	9.03E-96
CSRNP3	-2.048759	3.81E-28
ZBTB34	-2.049244	6.76E-64
LAMA4	-2.04968	1.07E-48
DNAJC8	-2.05033	1.78E-250
TECTA	-2.050626	1.3E-07
CDC6	-2.050917	3.23E-160
ANK1	-2.051003	1.85E-25
ZNF430	-2.051592	7.17E-32
CENPU	-2.051839	4.18E-55
TMEM178B	-2.052151	5.22E-26
ZBTB16	-2.053111	0.0003071
FGD3	-2.053638	2.06E-06
SFPQ	-2.053692	5.52E-180
KDM4D	-2.053771	1.21E-09
GALNT16	-2.054522	1.09E-61
ITGA2B	-2.054792	8.62E-08
PKN3	-2.054998	3.22E-60
TRIM45	-2.055481	3.35E-22
KHDC4	-2.055887	2.4E-130
AUTS2	-2.056168	8.05E-38
STAG2	-2.056374	6.78E-189
SYT16	-2.056831	4.62E-11
PPM1L	-2.056875	1.97E-37

HOXB6	-2.056911	1.37E-13
USP45	-2.057472	2.36E-27
DBF4B	-2.059454	2.48E-35
THOC3	-2.060348	6.77E-161
MARCKSL1	-2.060418	6.35E-142
PNISR	-2.060967	2.05E-147
ZNF713	-2.061816	4.28E-09
CASP2	-2.062002	2.73E-106
C19orf44	-2.062311	1.18E-37
PNN	-2.062698	1.23E-239
KIAA0895L	-2.06281	9.04E-18
ENTPD1	-2.063128	6.26E-64
CEP85	-2.066122	4.31E-78
CCDC110	-2.066495	0.0092045
SMC4	-2.067453	1.43E-130
GON7	-2.067645	3.15E-30
TMEM80	-2.068123	9.33E-40
HDAC1	-2.070942	6.31E-211
SLC25A21	-2.072408	1.75E-06
SHISAL1	-2.072756	5.57E-15
KCNJ11	-2.074768	6.64E-18
CDK2	-2.075052	6.17E-105
SYTL4	-2.077804	2.57E-36
TIGD1	-2.079135	1.04E-25
PMAIP1	-2.079177	1.19E-123
MTSS1	-2.0799	3.44E-18
PARD6G	-2.080835	2.17E-20
HADH	-2.081876	4.28E-87
SEMA6C	-2.08254	2.53E-21
SHPRH	-2.082865	2.48E-75
PPM1K	-2.08379	1.26E-28
ZNF774	-2.084224	1E-30
TBX1	-2.084662	6.91E-10
FBLL1	-2.085973	0.0066396
MGAT4A	-2.086208	1.68E-36
FOXC2	-2.086509	3.75E-06
LSM11	-2.086889	2.29E-64
MCM6	-2.087961	1.79E-129
MAP2K7	-2.088365	1.91E-77
ANKRD20A1	-2.088729	0.0000167
TPTEP2-CSNK1E	-2.089171	0.000056
TTC7B	-2.090506	4.25E-117
CHERP	-2.091722	2.52E-168
CKAP2	-2.092621	3.49E-152
DUSP7	-2.093411	8.57E-32
ZRANB2	-2.096114	1.77E-244
KDELC2	-2.096401	1.23E-144
PRIM1	-2.098422	2.67E-122
SRSF11	-2.09987	3.29E-175
GRID1	-2.100088	2.98E-10
HOXA10	-2.100976	1.18E-50
ABHD1	-2.102098	1.06E-06
MRNIP	-2.103986	6.49E-66
GDNF	-2.106199	0.0015036
KNTC1	-2.106408	2.05E-132
SUSD5	-2.106994	1.84E-27
BIRC5	-2.107185	7.42E-97
PPAN-P2RY11	-2.108158	8.71E-09
PPP4R4	-2.109419	5.74E-15
SLC16A9	-2.109562	4.58E-21
AP001267.5	-2.111773	0.0000248
RBM4B	-2.113924	1.42E-33
FEM1A	-2.114986	2.32E-99
MAPT	-2.115289	2.85E-28
HOXC6	-2.116841	1.9E-17
CCHCR1	-2.117652	4.1E-59
TMEM98	-2.11934	1.54E-41
TRMT2A	-2.11934	1.33E-77

RASSF5	-2.119847	7.86E-21
ZNF852	-2.121687	4.54E-10
NASP	-2.121989	1.47E-157
TMEM38B	-2.124116	9.86E-64
ELOVL4	-2.124206	2.42E-47
CENPE	-2.125891	1.41E-56
ZNF782	-2.126204	4.53E-33
PAG1	-2.126377	1.57E-27
FAM160A1	-2.126532	4.89E-23
RNASEH2C	-2.127879	8.08E-114
KIF18B	-2.130917	4.73E-53
TTLL4	-2.132273	2.65E-104
TTF2	-2.132821	5.07E-116
SOX8	-2.132951	6.69E-07
SHF	-2.133319	4.84E-07
ZIC2	-2.133962	1.39E-58
EPB41	-2.134586	2.15E-219
STMN1	-2.13614	4.37E-276
DCAKD	-2.136154	2.79E-38
ZBED8	-2.137374	7.57E-26
AL121768.1	-2.137682	9.51E-54
S1PR2	-2.13774	1.15E-18
SHC2	-2.139024	1.7E-17
DNA2	-2.139469	3.11E-74
BARD1	-2.139504	3.47E-59
SKA3	-2.14006	2.91E-54
NPAT	-2.141822	1.13E-64
DSN1	-2.142585	6.92E-78
SERBP1	-2.142934	0
RBM15	-2.143071	5.95E-69
RRM2	-2.143211	1.21E-102
DISP2	-2.14543	2.28E-09
AC093668.3	-2.145733	1.51E-31
INKA2	-2.145886	2.79E-24
HIRIP3	-2.147458	1.1E-36
SLC16A14	-2.147952	2.14E-28
KIF24	-2.148046	2.1E-56
RFC2	-2.148562	1.88E-137
NPTX1	-2.148945	1.2E-70
FAM234B	-2.149012	1.35E-81
SH3RF3	-2.14903	3.49E-11
HAP1	-2.151631	1.38E-22
UPF3B	-2.152147	3.16E-79
SLC24A4	-2.15277	4.31E-08
KCTD12	-2.154498	9.49E-48
LYG1	-2.154681	0.0003602
POLA1	-2.155583	2.29E-146
WDHD1	-2.156566	3.39E-96
USP2	-2.158481	7.43E-28
TRIM73	-2.158527	1.39E-10
RASAL1	-2.160465	3.29E-07
HAUS3	-2.168316	3.85E-58
FAM155B	-2.168489	2.12E-26
RIPPLY3	-2.168662	8.23E-07
RIMBP3C	-2.169925	0.0044708
TBX2	-2.170601	2.96E-16
FOXD3	-2.172269	3.48E-10
RTKN2	-2.172807	1.82E-36
IGSF9	-2.174251	0.000042
GLDC	-2.175258	1.76E-56
ZNF608	-2.176878	2.87E-47
MTMR7	-2.177538	3.64E-14
ADAM23	-2.177538	1.1E-34
SCN3B	-2.178642	2.66E-13
STK40	-2.178939	3.41E-94
SSBP3	-2.181785	4.32E-90
TULP2	-2.182203	0.0018285
GLCCI1	-2.186655	5.31E-18

MED28	-2.190684	2.97E-144
CTSV	-2.19186	3.85E-56
C8orf34	-2.192645	0.0061661
SLIT1	-2.193681	1.62E-12
TMPRSS9	-2.194378	0.0030395
CACNA2D3	-2.194647	4.32E-14
KCNH2	-2.195148	6.45E-10
ST3GAL6	-2.196143	1.24E-12
CDK18	-2.196252	5.26E-71
EXPH5	-2.197192	1.97E-17
FGF11	-2.199203	1.57E-22
KBTBD6	-2.201169	4.89E-70
PKMYT1	-2.203963	1.41E-70
TICRR	-2.204235	2.89E-88
OTUD3	-2.204678	2.82E-78
RELT	-2.205886	9E-28
RAPGEF3	-2.207595	1.03E-11
AC093323.1	-2.208291	1.84E-35
GTF2H2	-2.210074	1.52E-108
VASH1	-2.212577	1.31E-57
RGL4	-2.215133	6.87E-12
ASIC1	-2.215483	8.64E-83
NECAB1	-2.216318	7.9E-12
DERL3	-2.216687	3.08E-17
Z83844.3	-2.217987	0.001046
RCBTB2	-2.218092	1.52E-06
ANKRD36B	-2.218284	1.33E-16
OXCT2	-2.218571	0.000141
RET	-2.219313	3.3E-12
CDCA5	-2.220772	6.32E-122
ADAT2	-2.221606	1.31E-41
SATB2	-2.223154	3.13E-53
LIN52	-2.225881	6.62E-56
MRI1	-2.22635	1.81E-81
MLXIPL	-2.228414	4.95E-27
HELLS	-2.231329	9.88E-186
EME1	-2.231647	3.29E-50
NEMP1	-2.231842	1.34E-195
CENPI	-2.233149	1.25E-50
PCNT	-2.233944	2.73E-128
ZNF483	-2.234275	5.05E-09
CACNB2	-2.236634	6.37E-13
AC011448.1	-2.237039	0.003444
PAXBP1	-2.237655	1.33E-124
FOXRED2	-2.240977	2.35E-154
C2	-2.243736	6.6E-10
MTURN	-2.246085	7.14E-122
OTUD7A	-2.246214	6.29E-06
C6orf163	-2.246311	0.0000217
CHRM3	-2.246438	0.0000017
MESP2	-2.24878	0.000163
OCA2	-2.248967	0.0000014
ZC3H12B	-2.250375	1.69E-11
FOXO4	-2.250378	2.36E-85
CHN2	-2.250429	2.11E-08
BLM	-2.253869	4.3E-92
FAM57B	-2.254024	0.0025869
DCLRE1B	-2.254997	1.78E-61
ANKRD6	-2.256113	1.14E-46
SERPING1	-2.257271	4.44E-14
MAZ	-2.258141	0
SALL2	-2.258788	5.07E-81
KRI1	-2.259659	3.72E-98
C8orf88	-2.260215	1.05E-23
USH1G	-2.261668	0.000057
HS3ST3B1	-2.262656	2.83E-20
SRRT	-2.263732	2.04E-162
GTSE1	-2.269977	4.21E-71

CCDC138	-2.270295	2.09E-48
ID2	-2.273919	4.31E-112
DDTL	-2.274385	3.07E-14
ARHGAP20	-2.275634	0.0049257
SLC27A6	-2.275634	0.0008017
GABRA3	-2.27684	4.99E-07
BARX1	-2.276891	2.28E-44
CEP135	-2.277152	2.61E-71
TRIM52	-2.277694	2.16E-77
RSPH4A	-2.277985	0.0005382
KLHDC3	-2.278293	3.21E-163
SLC27A3	-2.279128	1.78E-12
TIGD4	-2.283172	3.15E-06
ITGB3BP	-2.283823	1.7E-51
SPRYD4	-2.284113	7.26E-44
GBX2	-2.2862	1.81E-09
ARHGAP8	-2.287237	1.89E-20
NRIP3	-2.289425	8.29E-31
PLXDC1	-2.289507	0.0008566
FAM229A	-2.290758	0.0077191
FANCD2	-2.291241	1.69E-154
PIM3	-2.292403	8.78E-133
PMEL	-2.292431	2.79E-11
NCR3LG1	-2.292644	1.45E-101
RAVER2	-2.293803	5.67E-143
OBSL1	-2.297565	4.55E-142
DPYSL5	-2.298883	9.5E-135
WLS	-2.301839	1.15E-194
ZNF678	-2.30317	1.44E-109
SESN1	-2.30356	6.58E-99
PXMP2	-2.304478	1.28E-50
PARP1	-2.304758	0
DEPDC1	-2.30533	1.9E-122
FIGNL1	-2.305496	7.67E-134
MFSD13A	-2.307035	6.9E-42
BRIP1	-2.309003	2.2E-87
NT5DC2	-2.310455	5.2E-133
EPHX4	-2.311333	6.63E-07
SASS6	-2.311902	2.43E-72
CSRP2	-2.312163	1.38E-55
EYA3	-2.313294	8.2E-99
TUBB8	-2.315776	0.0021198
KIF17	-2.316237	7.44E-24
C11orf65	-2.318405	0.0001061
SIKE1	-2.319807	1.02E-152
WDR76	-2.320629	4.11E-67
MSX1	-2.32119	1.08E-33
EPHB3	-2.321928	0.0000749
PLA2G12A	-2.323966	1.5E-88
GTPBP3	-2.325059	8.36E-87
SYCE2	-2.326044	0.0005848
EBF4	-2.326271	2.76E-27
MYO1D	-2.327677	1.29E-67
ARG2	-2.329812	1.65E-48
ANKRD20A4	-2.330917	0.000587
ASAH2	-2.335103	0.0000228
CASP8AP2	-2.339641	6.97E-79
GOLGA8A	-2.340676	2.61E-121
RAD51AP1	-2.343784	2.47E-107
ABCC8	-2.344067	7.83E-27
PPIH	-2.34458	1.12E-37
CCNG1	-2.344629	0
MPC1	-2.345374	1.26E-36
LMNB1	-2.346905	7.94E-198
PLAGL1	-2.347409	2.45E-164
ST6GALNAC3	-2.348026	5.37E-11
ZNF443	-2.3484	2.31E-08
TRAIP	-2.348904	3.63E-28

DONSON	-2.350696	1.79E-110
NDC80	-2.353669	7.43E-53
MND1	-2.353782	9.89E-51
DIAPH3	-2.354821	1.07E-81
PHOSPHO2	-2.3561	9.5E-16
KIFC1	-2.356814	2.02E-83
CALY	-2.357552	0.0030648
TRIM74	-2.358454	0.0012439
SHISA2	-2.359227	6.2E-08
ORC6	-2.360483	1.67E-75
POLE	-2.362189	9.31E-244
AC135050.2	-2.364103	0.0000893
NID1	-2.365099	1.9E-238
AC009779.3	-2.365476	4.93E-14
ARC	-2.365906	2.13E-07
LMX1B	-2.367341	4.74E-47
ADAT3	-2.367732	1.22E-10
HOXB2	-2.369878	7.98E-17
STRC	-2.370838	1.16E-07
ZNF771	-2.371203	1E-30
DTL	-2.371876	1.34E-160
DZIP1L	-2.372533	4.47E-21
TTC34	-2.373458	0.0005013
SLC25A42	-2.378704	3.23E-23
ZNF121	-2.379301	8.02E-100
ZWINT	-2.38299	3.04E-121
STOX1	-2.383843	8.65E-15
ASB16	-2.385822	0.0008949
C1orf226	-2.389042	4.39E-12
FAM117A	-2.390197	1.12E-43
NES	-2.393914	1.71E-13
TOR2A	-2.394628	1.87E-67
EFNA2	-2.394684	2.16E-09
LHX6	-2.397189	5.86E-15
CALCB	-2.39837	1.97E-24
RAPGEF4	-2.40077	1.58E-26
PHF21B	-2.407552	2.21E-12
KCNQ4	-2.411694	3.44E-11
ADGRB1	-2.412647	3.19E-24
ZNF28	-2.415037	5.74E-23
GPR27	-2.416816	4.39E-31
ZNF273	-2.418194	1.35E-22
SPC25	-2.419637	1.25E-27
NEIL3	-2.42053	3.68E-89
ZNF468	-2.423542	1.71E-43
PRSS12	-2.424907	1.06E-23
SYNE3	-2.425022	1.42E-34
B3GAT1	-2.426265	0.0027901
PUSL1	-2.426371	4.54E-50
NCAN	-2.428843	0.0013478
ITGA4	-2.429138	6.63E-33
GREB1L	-2.429865	1.51E-66
FGF5	-2.429988	0.0012644
FIBIN	-2.432959	2.09E-14
BSPRY	-2.43351	8.53E-08
PGF	-2.434072	2.63E-13
USP1	-2.434221	0
CDIP1	-2.436073	2.14E-80
GCHFR	-2.438252	1.1E-12
STAB1	-2.440573	0.0044743
TYRO3	-2.441019	2.04E-78
PAX2	-2.4411	2.07E-08
C16orf86	-2.441916	3.13E-06
SUGCT	-2.442602	8.47E-12
GCAT	-2.447972	4.33E-24
NCAPG	-2.448257	1.69E-239
NR3C2	-2.44859	6.49E-16
HIC2	-2.450252	1.87E-59

TMEM86A	-2.450826	2.85E-13
TCEANC2	-2.451422	2.05E-166
HAUS5	-2.45298	1.14E-113
ESPL1	-2.456944	3.64E-123
CCDC62	-2.459432	0.0020438
CTH	-2.462436	2.8E-71
MMACHC	-2.462869	9.51E-45
E2F7	-2.463361	4.43E-135
UHRF1	-2.463409	1.26E-157
E2F8	-2.465949	1.09E-32
IKZF3	-2.467606	1.77E-13
TJP2	-2.468042	5.65E-129
LRRC39	-2.468996	9.07E-06
B3GAT2	-2.469114	9.53E-11
ARTN	-2.471306	0.00089
NCAPH2	-2.47161	3.96E-111
COQ8A	-2.471914	1.65E-149
EFNB2	-2.472676	4.97E-27
RNF138	-2.47271	6.68E-118
DSCC1	-2.473658	4.59E-80
ACKR4	-2.473931	0.0090272
ARL10	-2.475002	5.99E-297
DCHS1	-2.475733	0.0000034
TCF24	-2.477047	0.0005179
SAGE1	-2.479231	4.32E-13
SPEG	-2.482465	9.26E-33
PLEKHO1	-2.484805	2E-31
MMP16	-2.485004	5.61E-56
APOE	-2.487061	1.07E-72
FANCE	-2.489329	1.21E-45
GALNTL6	-2.489385	0.000032
GSC	-2.490986	0.0006008
ACVR2B	-2.491088	1.8E-158
SMIM10L2B	-2.493989	9.98E-07
SNAI2	-2.495968	1.04E-37
PLAT	-2.496612	8.99E-104
ATAD5	-2.496697	2.41E-66
SLC9A9	-2.49834	0.0000038
CEL	-2.498923	1.31E-08
IQCC	-2.501666	3.74E-27
PODXL2	-2.503129	4.64E-72
SULT1A1	-2.504116	5.02E-25
POLA2	-2.505347	2.78E-139
RPA2	-2.50656	3.33E-161
AP002495.2	-2.507447	9.78E-07
XYLB	-2.507874	1.45E-68
TTN	-2.51245	2.56E-41
CLEC11A	-2.513095	1.24E-17
MAD2L1	-2.513628	1.17E-196
PTPRU	-2.514065	8.91E-221
VAMP1	-2.515668	4.05E-25
SOX7	-2.516917	6.25E-14
CHAF1B	-2.517171	5.78E-131
TBC1D32	-2.517297	1.46E-32
TMPRSS13	-2.519374	0.0089052
CHRNA3	-2.520353	6.44E-14
HOXC4	-2.520422	4E-36
IRX5	-2.521683	1.01E-14
DIRAS1	-2.52275	1.33E-70
HCN3	-2.522997	1.46E-49
EFHD1	-2.523682	3.52E-54
ARMCX4	-2.525628	4.6E-22
PPP1R3E	-2.526421	9.96E-69
BACH2	-2.527932	8.44E-22
PHF7	-2.528704	1.69E-22
CBFA2T3	-2.530515	0.0035358
NCAPD3	-2.531018	1.35E-246
CFAP43	-2.532111	8.48E-12

GDF11	-2.532334	3.45E-43
STON1	-2.532414	1.07E-38
ATP1A3	-2.532668	1.51E-54
DSC3	-2.532728	2.91E-123
ANKRD34A	-2.534232	6.66E-12
ONECUT2	-2.534689	1.03E-99
ATXN7L2	-2.535332	9.59E-23
COL6A6	-2.536053	0.0087037
RNPC3	-2.536556	6.59E-111
IGSF9B	-2.538593	1.02E-23
LRRC66	-2.541894	0.0032846
RECQL4	-2.54255	1.35E-108
WNT2B	-2.544321	6.49E-23
SCN5A	-2.545279	8.55E-52
ROBO3	-2.545342	2E-24
HMGN2	-2.547431	0
HSPA1B	-2.548589	9.93E-173
ZNF44	-2.549967	1.8E-13
GPSM3	-2.550197	0.0064439
KCP	-2.550197	0.0004912
MAK	-2.550197	2.11E-06
ARID3B	-2.552465	1.3E-80
PSMC3IP	-2.553863	2.13E-37
FOXC1	-2.554676	4.43E-109
WNK2	-2.555775	2.21E-125
NHSL2	-2.558268	7.38E-34
AP3B2	-2.558368	9.73E-11
CENPW	-2.559546	1.79E-26
CHRNA4	-2.560715	0.0066122
HBQ1	-2.562059	1.38E-10
RNASEH2A	-2.562635	1.18E-139
NCAPH	-2.563644	9.47E-129
MEGF10	-2.564785	0.0001363
GRIK5	-2.565199	1.98E-06
SBSPON	-2.567041	0.0000947
HOXD9	-2.567571	8.14E-21
SCUBE3	-2.567685	0.0000417
PLK4	-2.570749	5.85E-128
TAF5	-2.573109	1.04E-77
UNC5B	-2.575967	1.73E-113
ZNF594	-2.577088	7.71E-52
RDH12	-2.578076	0.0026444
NIPAL1	-2.582375	2.31E-17
TMEM74	-2.584963	5.2E-07
SFXN2	-2.58918	2.15E-91
NEURL1	-2.59238	1.2E-09
MPP2	-2.59316	3.3E-83
RLN2	-2.596676	4.33E-06
GPR153	-2.597902	1.44E-27
CUX2	-2.599525	1.41E-24
PPP1R16B	-2.599652	1.49E-24
SELENOP	-2.600111	3.02E-43
PLEKHH1	-2.600375	6.34E-95
C10orf95	-2.600393	0.0000991
SLC44A5	-2.600411	9.82E-55
NKX6-1	-2.60138	4.51E-23
ADCY1	-2.603549	4.97E-94
ZNF138	-2.603706	1.14E-51
PLTP	-2.60395	1.25E-68
SMIM4	-2.604442	2.86E-11
FANCM	-2.610123	7.19E-65
FZD4	-2.611297	2.01E-49
DNMT1	-2.612242	0
TRABD2B	-2.612443	8.06E-08
CDKN2C	-2.615492	4.87E-54
TEX14	-2.616671	0.0010843
C1orf21	-2.617171	9.45E-89
RUBCNL	-2.618239	1.26E-06

TSSK3	-2.618767	3.28E-17
HVCN1	-2.620009	0.0002141
PASK	-2.62179	7.55E-48
GLB1L2	-2.622549	3.59E-48
COL6A1	-2.623079	1.96E-217
TMEM52	-2.624184	5.17E-12
ZNF367	-2.625261	1.46E-89
L3MBTL1	-2.626705	9.26E-26
TNIK	-2.631375	1.1E-79
FANCB	-2.633074	7.9E-25
LRRCC1	-2.633604	4.16E-30
RNF128	-2.633636	4.97E-49
PTGIS	-2.63743	0.0036907
TESK2	-2.640504	9E-33
CCDC189	-2.645255	1.71E-14
CHRDL1	-2.645992	0.0012542
ZIC1	-2.645992	0.0003105
POLE2	-2.647039	1.29E-73
SPAG8	-2.649814	0.0034486
GJA9	-2.653442	0.0000963
ZNF433	-2.653675	0.0002096
ORC1	-2.654354	5.67E-153
SLC16A10	-2.654367	2.36E-58
NEXMIF	-2.655352	2.94E-06
SLC15A1	-2.656347	0.0003802
F8A3	-2.657299	3.49E-07
MCM3	-2.661629	0
NRTN	-2.66446	2.88E-09
LARGE2	-2.667195	1.29E-40
CYB5RL	-2.66905	6.23E-121
ADAMTS1	-2.67223	0
ANK3	-2.673808	3.88E-64
TRIM17	-2.676461	0.0004177
BHMG1	-2.678072	0.0001744
SERPINA10	-2.678072	0.0000423
HOOK1	-2.679229	1.26E-122
AC092329.3	-2.67948	0.0024049
NTRK2	-2.680721	4.45E-06
U2AF1	-2.680879	1.98E-09
SYT3	-2.681908	1.3E-23
TMEM145	-2.684687	2.28E-13
HOXD11	-2.690017	2.54E-32
PRR22	-2.690862	2.14E-12
GLUL	-2.693022	1.69E-269
S1PR1	-2.693487	0.0006455
CACNB4	-2.696324	6.46E-57
OPRD1	-2.70044	4.56E-07
RUNDC3A	-2.703799	0.0014428
CNTNAP2	-2.703799	3.15E-09
PRKCB	-2.706034	1.31E-26
FRMPD1	-2.706953	0.0000754
ADARB1	-2.707184	1.44E-61
PPT2	-2.710928	1.15E-87
SOX18	-2.711279	6.97E-09
ABHD12B	-2.715073	2.76E-06
ANKRD34B	-2.717413	2.51E-09
GPR137C	-2.718088	3.95E-16
RASIP1	-2.718142	1.78E-11
LAMC3	-2.718818	5.3E-48
IL9R	-2.721425	1.11E-07
PRSS35	-2.724597	1.53E-09
PDZD7	-2.725243	3.28E-24
ENPP4	-2.732249	2.71E-68
HOXD10	-2.733251	1.46E-57
MAFA	-2.733826	3.93E-08
FOXD2	-2.733826	1.96E-19
HOXB4	-2.734189	0.000014
CACHD1	-2.735365	7.46E-107

TTYH1	-2.736966	0.0006464
CCDC136	-2.737709	2.64E-42
PROX1	-2.739291	2.39E-19
MGAT3	-2.742019	4.19E-47
UNC5C	-2.742109	1.33E-19
CCDC15	-2.74311	7.61E-38
MOCS1	-2.743841	1.18E-62
ZNF789	-2.74599	2.8E-46
ZNF649	-2.747854	1.65E-24
KIF26A	-2.749755	2.26E-19
MCM4	-2.751611	0
CERS1	-2.755241	5.65E-43
E2F1	-2.756335	7.43E-151
MPP7	-2.757655	2.02E-22
SEMA4A	-2.757697	2.21E-10
HES1	-2.760452	7.58E-29
CCDC74B	-2.764264	1.36E-27
PRRX1	-2.765535	0.0029537
ZFPM2	-2.765535	5.79E-10
WDR63	-2.767248	3.21E-14
MAP1A	-2.769387	6.34E-36
FAM78A	-2.773003	1.99E-33
RIMS2	-2.775294	0.0000938
DCAF16	-2.775608	1.16E-218
AGAP2	-2.777206	8.4E-52
PTGER2	-2.778644	9.08E-08
LOXL1	-2.779341	3.08E-12
FANCA	-2.780034	1.9E-189
SPON2	-2.780513	0.0008971
EML6	-2.78136	2.37E-26
MGARP	-2.783179	1.41E-26
KRT17	-2.785495	0.0046761
TEX45	-2.785875	0.0029089
TMEFF2	-2.786596	0.0007435
FMNL3	-2.793732	1.11E-60
CRISPLD1	-2.794022	5.36E-38
CYSLTR1	-2.79518	0.0027587
KIAA1257	-2.796467	0.004396
KBTBD8	-2.798041	1.19E-20
ZNF701	-2.800147	3.86E-14
SLC2A4	-2.802478	7.9E-16
MDC1	-2.80293	1.27E-266
CCDC153	-2.803735	0.0004331
TGFB3	-2.804449	3.72E-17
KLHL13	-2.809851	3.85E-19
COL3A1	-2.813741	1.53E-06
HPCA	-2.814697	0.0051057
HMGB2	-2.816851	8.31E-290
CABP1	-2.817623	0.000323
HLA-DQA1	-2.821499	0.003206
PROCA1	-2.824596	4.89E-10
KCNG3	-2.825502	1.22E-13
CCDC28B	-2.826744	7.4E-24
PLPPR4	-2.830075	0.0017037
SH3BP1	-2.830886	3.45E-17
RBMXL1	-2.83262	1.85E-89
NDNF	-2.832629	3.42E-14
ALDH8A1	-2.833479	1.38E-13
KCNK13	-2.83399	0.00069
CARMIL2	-2.835442	3.41E-23
C2CD4C	-2.835995	1.06E-09
PLAC1	-2.839399	1.16E-19
THEMIS2	-2.840365	4.39E-19
FAM83B	-2.842016	1.02E-14
CCDC18	-2.84343	6.32E-49
BEX4	-2.846784	7.95E-174
GRHL1	-2.848858	6.06E-16
P2RY1	-2.850857	5.17E-34

CFAP77	-2.851749	0.0010539
SBK1	-2.854932	2.35E-47
CLCNKB	-2.856841	1.43E-14
DDN	-2.85726	8.53E-14
PITX2	-2.860936	3.96E-37
ADGRL2	-2.862473	5.96E-172
RAB3A	-2.863229	1.17E-10
SLC25A27	-2.863407	2.58E-29
PDXP	-2.863574	3.74E-113
MCM2	-2.864471	0
ARHGAP28	-2.865499	1.33E-15
CRLF1	-2.867949	7.66E-29
TET1	-2.86818	5.62E-85
CAND2	-2.874469	2.63E-47
TSPYL4	-2.87489	2.53E-101
TMEM38A	-2.875206	4.71E-45
CYP4X1	-2.877744	0.0016779
STX1B	-2.881217	5.44E-13
CNIH2	-2.882164	0.0001483
PTGFR	-2.882643	0.0055827
TMOD1	-2.882803	2.32E-41
LGI2	-2.886132	3.37E-06
MYBL2	-2.886776	0
AUNIP	-2.886906	1.51E-79
S100A1	-2.893085	0.0019815
RAC3	-2.895513	2.42E-117
STON2	-2.895857	1.18E-32
CDK14	-2.896474	3.67E-37
CCDC181	-2.896979	7.64E-32
CDKN1C	-2.897509	9.53E-13
EMID1	-2.898985	1.42E-11
DOCK8	-2.900004	5.09E-12
NUDT11	-2.90079	1.89E-61
MTUS2	-2.906891	0.0050039
FAT2	-2.906891	0.0017087
ASRGL1	-2.908502	1.98E-44
LDHC	-2.910448	2.05E-09
PROX2	-2.915608	0.0040094
TOGARAM2	-2.915608	5.91E-15
KLC3	-2.916738	9E-25
GAL	-2.919296	1.13E-15
SCARF2	-2.919806	1.25E-35
C21orf58	-2.921183	1.02E-63
ESRRB	-2.921997	1.1E-15
ISM2	-2.924813	0.0024548
ERCC6L	-2.925087	7.19E-112
C1orf54	-2.926182	0.0000351
ESR2	-2.927149	6.29E-06
FP565260.3	-2.927649	0.0025216
TSPAN11	-2.92785	1.25E-07
TRIM2	-2.930568	6.57E-105
ELFN1	-2.931765	5.91E-10
PARP8	-2.932733	1.54E-58
ADAM22	-2.932971	7E-61
FRRS1L	-2.940921	3.63E-35
MAPK4	-2.941448	9.65E-14
HOXA11	-2.944147	2.94E-21
PLIN5	-2.944858	0.0091351
KPNA5	-2.946574	1.45E-87
RFXAP	-2.946875	1.07E-35
EXO1	-2.951668	1.32E-206
SHANK1	-2.959358	1.02E-07
RND2	-2.959358	1.67E-20
TCF19	-2.960542	4.04E-138
IGDCC3	-2.961526	1.25E-15
BTNL9	-2.963474	6.66E-23
PNPLA3	-2.964106	8.95E-17
KLRG2	-2.964936	1.28E-21

ARHGEF6	-2.965167	7.05E-62
ZNF347	-2.966833	0.005646
HOXC10	-2.967733	1.77E-56
GPR63	-2.96782	7.15E-33
SOX6	-2.977747	3.05E-17
NMUR1	-2.981853	0.0000792
CAVIN4	-2.984403	2.15E-28
ARL17A	-2.98738	1.67E-271
CRACR2A	-2.988372	9.46E-12
CCDC151	-2.991779	8.68E-16
RNF125	-2.991779	3.07E-44
PRKG1	-2.992911	1.27E-16
RIMS4	-2.9934	9.84E-87
NPPC	-2.997902	1.05E-06
IZUMO4	-3	0.0031991
CNBD2	-3	9.6E-09
GRAMD1C	-3	9.39E-20
FNDC5	-3.002815	8.1E-09
SAXO2	-3.003752	5.11E-08
MCM10	-3.006291	1.9E-199
CDT1	-3.007698	0
PLA2G7	-3.008052	1.37E-08
PLCL1	-3.018536	4.42E-08
LOXL3	-3.019089	2.68E-35
COL9A3	-3.021349	1.71E-18
MCOLN3	-3.023145	7.64E-95
SLAIN1	-3.023519	2.91E-134
ACPP	-3.031849	0.000266
LMOD1	-3.032576	5.11E-24
SPC24	-3.038093	3.7E-68
GDF6	-3.038135	3.07E-07
CHTF18	-3.040635	2.41E-128
SLC25A48	-3.041477	0.0004529
KCTD19	-3.041758	4.34E-13
CYP4F22	-3.046294	0.0000887
ARMH4	-3.046474	2.64E-19
SECTM1	-3.048759	2.58E-08
MRVI1	-3.052467	4.44E-11
MCC	-3.056228	2.18E-94
NPIPB15	-3.057551	1.44E-08
E2F2	-3.060301	3.61E-110
LINGO3	-3.062284	7.75E-06
RGS16	-3.065001	5.17E-29
RCAN2	-3.066089	0.0000914
RASL11A	-3.066467	4.79E-12
DPF1	-3.068022	1.35E-37
KLHL23	-3.069879	2.95E-139
ICAM5	-3.07397	8.16E-51
ATP8A2	-3.075288	0.000132
FAM71E1	-3.075503	6.25E-27
RAC2	-3.085488	0.0000763
P2RX1	-3.087463	0.0065549
CCSER1	-3.087463	1.67E-06
LRP2	-3.090308	1.66E-74
FAXDC2	-3.091044	8.73E-35
MYH14	-3.092249	5.25E-132
DHFR	-3.093566	0
KCNN3	-3.096862	0.0001369
STAP2	-3.101538	0.0000841
EDA	-3.103999	1.49E-55
C21orf91	-3.104202	4.42E-95
THBS4	-3.105972	7.38E-09
MCTS2P	-3.106851	8.77E-10
APOBEC3H	-3.108836	0.0000173
LIN7A	-3.10905	3.16E-77
CAMK2N2	-3.110644	2.15E-07
CLSPN	-3.111022	4.26E-189
TRIM54	-3.11221	0.0021991

ACADL	-3.1127	1.21E-07
SYP	-3.117863	1.28E-44
GRK4	-3.121388	9.68E-26
CSPG5	-3.123259	3.25E-28
CDC7	-3.125679	0
BRCA2	-3.127171	1.1E-176
OSTN	-3.137504	1.87E-25
TSPAN7	-3.138925	8.64E-89
ENOX1	-3.142444	1.49E-31
CDC14A	-3.143949	2.09E-53
TENT5C	-3.144046	1.78E-19
GVQW3	-3.145308	7.26E-34
HOXA6	-3.145956	1.66E-18
DMBX1	-3.150679	9.31E-21
HRASLS	-3.150942	7.97E-06
MDK	-3.154812	0
CRIP3	-3.157776	0.0000952
C19orf57	-3.159199	1.51E-15
COL13A1	-3.159498	3.43E-20
GPM6B	-3.160245	3.38E-50
LINGO1	-3.163061	5.01E-64
GNG7	-3.163434	3.05E-26
FGF13	-3.16694	9.57E-29
FGFR2	-3.167727	8.5E-48
NKX3-2	-3.168038	3.24E-15
RDM1	-3.168741	6.48E-28
GINS2	-3.169622	3E-170
UBE2QL1	-3.169925	0.0000414
ELL3	-3.173927	6.33E-16
CORO1A	-3.175796	1.81E-13
PDZRN3	-3.182018	1.62E-19
GATA2	-3.1837	5.2E-80
ZBBX	-3.191141	0.0021691
ACSL6	-3.191141	9.8E-18
SLC7A8	-3.201439	2.1E-47
IL17RB	-3.203736	1.24E-41
PTGER1	-3.203872	0.000015
ZNF391	-3.204882	1.33E-44
NGFR	-3.205904	3.8E-12
CD207	-3.206107	6.53E-06
PCDHGA11	-3.206107	3.64E-12
KCNIP3	-3.206666	1.18E-10
AC099489.1	-3.210218	1.77E-30
SOSTDC1	-3.211504	0.001081
MFAP2	-3.212898	7.3E-26
TMEM100	-3.216656	0.0000788
ZNF790	-3.218019	2.42E-21
PAIP2B	-3.220876	9.65E-85
ARL17B	-3.225812	3.41E-45
NAP1L2	-3.227069	5.44E-20
ACYP1	-3.231094	5.22E-22
ZNF708	-3.232322	1.34E-40
RAD54L	-3.234065	9.15E-122
MASP2	-3.234342	5.28E-13
TMEFF1	-3.236567	1.44E-08
PPP1R1A	-3.238231	3.58E-08
CSAG3	-3.244856	1.12E-12
ATP1B2	-3.246521	1.85E-25
CILP2	-3.246873	1.6E-41
FAM186B	-3.249692	1.81E-08
LIN28B	-3.250189	6.85E-236
PAX6	-3.250574	1.45E-52
HOXA5	-3.252298	2.67E-89
TONSL	-3.252321	5.21E-229
ZNF695	-3.254421	1.06E-20
CDH23	-3.262896	1.51E-40
FGFR3	-3.267357	2.19E-94
TEX15	-3.268035	3.88E-46

CHRD	-3.268867	3.43E-10
STAC2	-3.271302	4.59E-08
TNFRSF14	-3.273018	0.0021832
HTR6	-3.275634	4.39E-11
MTTP	-3.27684	0.004761
SARDH	-3.278859	4.72E-10
HES5	-3.280491	2.06E-17
ZNF503	-3.280901	1.78E-169
SALL1	-3.283196	1.26E-98
GJB7	-3.285402	1.92E-07
PGBD5	-3.289834	2.2E-12
FLT1	-3.289879	4.9E-77
RNF165	-3.290677	2.72E-08
ZNF215	-3.291713	1.22E-49
MAPK15	-3.292112	1.07E-12
GPR50	-3.292602	2.18E-25
SLCO4C1	-3.294034	4.89E-19
EYA1	-3.294834	2.74E-13
SEMA5B	-3.295456	1.31E-07
CAMK2B	-3.297681	0.0002374
NKX2-1	-3.299208	6.05E-07
QRFPR	-3.302299	7.14E-11
KCNJ4	-3.304334	1.83E-06
DACT3	-3.308285	2.21E-19
TMEM25	-3.308915	8.35E-30
PLPP7	-3.310246	1.18E-09
ZCCHC12	-3.310585	1.79E-28
XKR6	-3.313417	0.0004399
ZNF703	-3.315657	5.85E-259
CCDC150	-3.316783	4.27E-81
RAPGEF5	-3.318877	1.25E-51
MAGEB6	-3.321928	0.0099213
SOBP	-3.321928	4.85E-36
ZFP69	-3.323193	5.59E-25
ALPL	-3.33254	3.31E-31
MAGEA9B	-3.332864	5.87E-35
ZC3H11B	-3.337035	6.83E-15
FRMPD3	-3.338607	4.7E-24
RAI2	-3.339137	0.0000644
PTH1R	-3.340307	3.19E-07
INTS6L	-3.343856	4.36E-34
SCML1	-3.344033	3.9E-278
RIBC2	-3.347463	4.26E-18
IFFO1	-3.348549	2.03E-50
PIK3R5	-3.350497	2.8E-07
FAR2	-3.354413	7.92E-33
HMGN5	-3.354828	2.16E-95
CDX2	-3.356187	2.94E-41
GPR85	-3.357552	0.0041632
DMC1	-3.359497	1.17E-39
LTK	-3.359542	9.63E-12
UNC5A	-3.365649	0.0000592
HTR3A	-3.369234	0.0094444
KIF26B	-3.373458	1.62E-13
SKP2	-3.373771	4.87E-264
GBX1	-3.374124	0.0065706
JAM3	-3.374987	2.72E-180
ROBO2	-3.375867	2.69E-09
MED12L	-3.382278	3.92E-62
MTAP	-3.384122	2.75E-265
DLX3	-3.392317	2.66E-07
44442	-3.397647	1.06E-124
ALOX15B	-3.398031	4.7E-07
MAPK8IP2	-3.398031	3.22E-107
LYPD6B	-3.399171	5.81E-06
CRABP2	-3.401828	7.67E-160
BARX2	-3.402098	6.82E-18
SMARCA4	-3.406259	0

RPS6KL1	-3.406709	1.31E-23
ZGRF1	-3.419039	4.77E-131
NACAD	-3.420332	1.97E-15
PIANP	-3.420777	2.17E-42
PPM1E	-3.421811	7.84E-84
FLT4	-3.424649	6.12E-110
ZNF714	-3.425592	1.53E-107
CA2	-3.427442	5.04E-102
COL14A1	-3.429657	4.13E-202
SEMA3D	-3.43221	1.29E-43
MPPED1	-3.432959	0.0002412
SYT6	-3.435925	1.13E-14
RFPL4A	-3.438573	0.0058207
ABCB4	-3.438573	8.35E-14
PMFBP1	-3.446256	0.001143
SPON1	-3.449307	8.21E-24
WNT3A	-3.452127	8.22E-16
ZNF440	-3.45269	3.34E-26
GUCY1B1	-3.454634	6.98E-84
CCNE2	-3.460812	3.03E-57
HOXD4	-3.46219	1.65E-10
CPLX1	-3.462707	1.07E-16
MSANTD1	-3.465974	0.0008777
PRIMA1	-3.465974	5.57E-08
ZNF441	-3.466318	8.09E-09
SORCS1	-3.468149	6.52E-07
SLCO2A1	-3.47032	0.0000149
NMU	-3.473275	6.99E-20
HEY2	-3.473799	1.06E-26
DOC2A	-3.476959	1.72E-39
GFI1	-3.478851	6.21E-45
MAN1A1	-3.481074	1.38E-167
C1orf167	-3.481127	0.002431
ACSBG2	-3.483083	0.0008286
KIF5C	-3.488181	4.03E-142
ZNF793	-3.488533	1.74E-20
ACVR1C	-3.491853	0.0014311
TMEM132E	-3.493738	6.75E-16
KCNA7	-3.504994	1.33E-07
PKIA	-3.51058	2.28E-51
PLEKHA4	-3.5118	1.85E-43
EPHA6	-3.513409	4.41E-13
TMEM255A	-3.513491	9.93E-49
AFF3	-3.513624	1.53E-40
EN2	-3.515556	1.94E-84
HRK	-3.516576	4.06E-59
NRARP	-3.517585	1.15E-149
ZNF345	-3.520257	9.2E-24
MSI1	-3.52221	6.32E-75
SEMA6A	-3.522461	1.29E-73
DENND2C	-3.531657	1.91E-40
DUSP9	-3.537771	3.44E-54
KIF5A	-3.538445	6.54E-113
ACVRL1	-3.542821	2.04E-09
KCNK9	-3.548437	0.0030753
SERPINF1	-3.549105	2.06E-218
TNFRSF13C	-3.549162	8.15E-25
DLX1	-3.559153	6.34E-25
NRXN2	-3.561085	3.56E-18
KCNB2	-3.567685	1.47E-06
DDR2	-3.570527	1.28E-167
GJA3	-3.583655	1.01E-45
VIPR1	-3.583683	3.85E-24
PPFIA2	-3.584963	0.0001773
SPINK2	-3.584963	5.65E-06
ROR2	-3.587108	4.82E-44
PLEKHB1	-3.588918	2.47E-83
CARD9	-3.591168	8.5E-14

DEF6	-3.592275	1.57E-19
SLITRK3	-3.597787	1.23E-13
NKX2-3	-3.598259	0.0046932
COLGALT2	-3.599093	5.21E-61
MAGEA11	-3.600061	3.67E-57
UGT8	-3.601652	9.1E-72
ADGRL3	-3.602158	6.74E-57
NTN1	-3.607726	6.1E-80
CKMT1A	-3.610599	8.65E-52
MEGF11	-3.611025	6.83E-15
GAL3ST4	-3.613914	7.89E-08
DNAAF1	-3.61471	0.0077123
AC007040.2	-3.621488	2.68E-12
EPHA8	-3.626783	2.18E-09
GIPC3	-3.626783	1.22E-23
MAGEA3	-3.627607	6.03E-11
TRO	-3.628182	1.78E-54
CCDC3	-3.634249	7.58E-58
SYN2	-3.634518	1.23E-21
ZNF382	-3.635428	5.18E-29
PDX1	-3.636309	1.13E-07
EPHA7	-3.639557	4.59E-79
ADAMTS3	-3.639728	1.81E-19
HPCAL4	-3.643144	2.72E-39
ASF1B	-3.652248	6.33E-222
ZSCAN23	-3.655352	1.38E-09
OLIG2	-3.659925	6.98E-15
ADAM11	-3.662394	2.81E-53
KCND3	-3.662965	0.0023446
GSTM2	-3.664927	2.62E-91
CCDC89	-3.667856	7.45E-09
BAIAP3	-3.670823	1.03E-50
AIF1L	-3.671441	5.31E-157
SEMA6D	-3.673772	9.57E-13
FOXP2	-3.673945	5.17E-43
MEF2C	-3.677612	7.12E-44
COL9A2	-3.681824	2.65E-10
C16orf96	-3.688056	0.0000495
MYB	-3.690182	1.12E-21
ETNPPL	-3.691162	0.0005714
TMEM198	-3.692092	4.03E-27
KCNJ8	-3.692196	3.17E-21
BSN	-3.692676	1.12E-73
ATP6V1G2	-3.699876	1.29E-33
FOXB1	-3.703018	4.55E-15
RGMA	-3.703456	1.44E-46
XRCC2	-3.70397	0
FLT3	-3.708345	1.95E-07
COL23A1	-3.710314	8.67E-18
FLNC	-3.713112	0
ADAMTS2	-3.71394	4.51E-35
GABBR2	-3.716207	0.0000164
NKX2-2	-3.717857	7.18E-10
PABPC4L	-3.718412	2.02E-72
ZNF556	-3.719892	4.94E-07
SNCAIP	-3.720477	9.79E-17
REEP1	-3.723188	1.16E-36
STEAP1B	-3.723317	2.22E-11
AFF2	-3.723377	2.52E-20
COMP	-3.729103	2.06E-13
TXNDC16	-3.730359	1.21E-135
PRRT4	-3.736541	2.27E-24
ZNF208	-3.736966	0.0024883
DACH1	-3.738042	3.83E-37
ARMC4	-3.740641	1.77E-20
POU4F1	-3.741228	1.16E-151
MMP11	-3.743629	4.41E-22
SULF1	-3.743952	4.49E-99

WASF3	-3.744556	7.75E-109
PCDH10	-3.751473	1.82E-120
GRASP	-3.752749	0.0001593
CAMK4	-3.755793	3.92E-32
ATP12A	-3.759737	9.1E-07
CACNA2D2	-3.762724	9.95E-43
BEX5	-3.763332	6.96E-32
PDZD2	-3.765535	1.55E-15
MATK	-3.765894	1.9E-63
CNTFR	-3.771059	1.5E-21
GRID2IP	-3.77444	8.39E-09
HES4	-3.779452	5.93E-209
MECOM	-3.779556	3.59E-50
DPF3	-3.782534	5.64E-19
TMEM121B	-3.794416	0.0000269
ZNF589	-3.795859	6.33E-193
IRF8	-3.798062	1.72E-27
HAND1	-3.798287	1.39E-33
LGR5	-3.798975	6.78E-20
GPM6A	-3.799232	4.92E-74
TMEM35A	-3.799406	0.0002176
PCDH18	-3.800762	1.21E-27
SYTL5	-3.80223	1.77E-75
MYT1	-3.80449	0.0001206
KIF6	-3.807355	0.0002017
GRIK4	-3.807355	4.59E-06
IFITM1	-3.809791	3.66E-63
PRUNE2	-3.811877	8.66E-46
COLEC12	-3.813883	3.73E-43
SIM1	-3.81526	1.49E-13
TMTC1	-3.817253	1.17E-65
SYPL2	-3.819668	7.8E-24
TRIB2	-3.820982	1.56E-47
KIF1A	-3.821716	8.74E-278
CADPS2	-3.824428	5.81E-23
TMEM272	-3.827819	0.0006903
TXLNB	-3.827819	1.48E-15
C10orf82	-3.833574	3.27E-10
ESPNL	-3.83605	2.16E-16
RUNDC3B	-3.83735	6.41E-34
NLGN1	-3.843465	2.54E-13
ZNF816	-3.84549	1.99E-09
GCK	-3.847997	0.0084197
ELAVL2	-3.848564	1.26E-47
CBSL	-3.849233	2.22E-22
DLX6	-3.852443	5.95E-12
SCUBE1	-3.855816	9.04E-14
EPB41L3	-3.857856	1.19E-161
ABCD2	-3.857981	0.0001738
CCDC102B	-3.861294	0.0011504
ZNF521	-3.863498	6.3E-24
NCAM2	-3.86507	5.68E-06
GPC3	-3.865491	1.31E-108
FHAD1	-3.866935	1.04E-61
HFM1	-3.868237	1.6E-21
SALL3	-3.870365	5.44E-26
ALG1L	-3.871542	2.25E-17
MPPED2	-3.880039	1.3E-16
HOPX	-3.884081	1.45E-08
EMX2	-3.888969	4.86E-09
ZMYND10	-3.890771	0.0092276
ACTN2	-3.893085	6.65E-18
IL21R	-3.894009	1.09E-06
DACH2	-3.898503	2.91E-11
SPAG17	-3.906891	0.0035268
CHST9	-3.906891	0.0014146
DLL1	-3.910585	5.85E-26
LRCH2	-3.91514	2.13E-59

ID4	-3.916038	2.72E-134
ZNF506	-3.920249	3.19E-46
ESPN	-3.921841	2.09E-06
AC139530.1	-3.922972	8.6E-29
NKX1-2	-3.923689	1.36E-30
KHDRBS3	-3.924421	1.72E-59
TEX19	-3.924757	6.03E-26
PRDM6	-3.926387	2.11E-56
DCAF4L1	-3.928107	5.69E-07
STUM	-3.929709	6.78E-47
ADAMTS17	-3.93002	8.73E-18
SPATC1L	-3.931956	4.38E-11
B4GALNT3	-3.933921	1.54E-63
EBF3	-3.936059	3.73E-79
SLC6A11	-3.940167	7.2E-13
TIAM1	-3.94679	7.69E-191
AC009086.2	-3.947511	9.64E-48
KLF14	-3.947533	0.0003878
PACSIN1	-3.947533	3E-21
CDC25A	-3.950673	3.32E-267
QPRT	-3.953137	1.39E-243
CPVL	-3.95516	9.86E-177
HOXC11	-3.955764	2.97E-30
NCAM1	-3.956095	2.27E-61
PTK7	-3.958224	7.52E-276
TSPAN12	-3.958937	1.17E-37
AASS	-3.963066	6.12E-101
RENBP	-3.965634	3.66E-15
CTCFL	-3.965784	0.0000697
ZNF83	-3.967012	2.24E-38
P2RX7	-3.969626	0.0026419
GLT8D2	-3.970078	1.88E-48
PLAG1	-3.971192	2.07E-69
CNRIP1	-3.975971	2.28E-37
MME	-3.976693	1.22E-20
KCNQ2	-3.97712	1.37E-29
TMEM108	-3.980548	0.0002298
PSTPIP2	-3.980914	6.74E-78
CCND2	-3.981249	2.32E-106
HS6ST3	-3.984893	1.14E-07
LRRC17	-3.985692	1.61E-06
GOLGA8M	-3.987061	0.0039453
GHR	-3.989139	1.75E-17
RAMACL	-3.995595	1.26E-12
CKB	-3.998146	0
SLC5A12	-4	4.6E-09
PI15	-4	1E-16
DNAH6	-4	1.73E-25
SHISA8	-4.00265	1.13E-07
LZTS1	-4.006426	4.15E-12
ART5	-4.009715	6.53E-08
KLF15	-4.011912	1.73E-34
TRIM71	-4.013297	1.07E-31
CDH22	-4.01495	0.0001522
SSTR2	-4.015825	9.24E-20
NOL4	-4.019194	0.0000818
ZNF536	-4.020782	1.25E-30
PNMT	-4.022368	0.003449
EMILIN3	-4.023469	1.38E-74
ITGA8	-4.023624	1.23E-49
NYNRIN	-4.024527	3.83E-115
EMX1	-4.026398	8.88E-14
SCN4B	-4.027292	4.56E-23
CITED1	-4.027631	1.24E-25
SPSB4	-4.028569	4.3E-22
SMTNL2	-4.028881	1.5E-11
RTN1	-4.031219	6.09E-10
SDC2	-4.031544	1.7E-121

ADAMTS20	-4.031849	5.12E-12
SOWAHA	-4.03868	2.11E-22
KCNJ12	-4.039341	1.24E-36
HS6ST2	-4.045206	2.06E-157
TP73	-4.046215	1.47E-26
EPHA3	-4.048698	9.55E-56
CD1D	-4.050626	5.87E-06
MAGEL2	-4.053111	0.0001976
SH3GL3	-4.054797	1.78E-33
NRGN	-4.057519	2.98E-64
RASL11B	-4.058894	0.0000112
SYK	-4.06301	7.51E-51
CFAP70	-4.063461	4.96E-38
TMEM266	-4.066529	2.22E-18
STMN3	-4.069299	4.79E-86
SOX5	-4.070389	0.0000374
NSG1	-4.070959	6.5E-40
HEY1	-4.07323	0
PCDH7	-4.075656	1.42E-121
ESRP2	-4.075844	1.58E-35
HLA-DQB1	-4.076316	5.52E-52
CTF1	-4.079097	6.73E-55
DLX5	-4.08017	7.7E-16
HLA-DPA1	-4.08145	7.39E-21
RIPOR2	-4.087463	0.0017045
LAMA1	-4.087463	2.11E-173
TRPV4	-4.090723	1.42E-13
CAMKV	-4.092446	4.43E-29
CLEC4F	-4.096862	5.04E-11
SATL1	-4.104337	0.0011114
CA14	-4.108524	0.0025728
PKNOX2	-4.111228	8.34E-45
KIT	-4.113601	5.45E-54
GUCY1A1	-4.117326	3.92E-23
SMAD9	-4.117867	8.17E-94
ZNF519	-4.118941	3.02E-29
INPP5D	-4.124071	7.89E-61
ITGA9	-4.129283	1.17E-11
LRRC69	-4.131451	0.001382
MMP23B	-4.13505	8.21E-23
MYOD1	-4.137504	0.000405
SYT5	-4.14083	3.66E-10
NOS2	-4.142958	1.96E-12
ZNF540	-4.145323	3.22E-10
LRRC61	-4.145498	1.39E-49
CLDN6	-4.146841	0.0002316
HS3ST3A1	-4.146841	6.21E-39
OGDHL	-4.150363	2.03E-55
CD80	-4.153805	0.0078166
ELMOD1	-4.154158	1.01E-34
SYN3	-4.158429	0.0000347
DLX2	-4.161187	3.38E-52
GDF7	-4.16355	2.49E-87
AC008687.1	-4.164309	1.22E-12
PRAP1	-4.165338	0.000035
GAD1	-4.166163	1.41E-33
DMD	-4.168897	3.19E-55
GLS2	-4.171666	3.64E-18
ENHO	-4.175362	1.43E-32
TENM1	-4.179909	1.76E-78
BMP7	-4.180956	5.51E-135
PDPN	-4.181728	2.94E-71
GPC4	-4.186061	1.13E-121
SLC38A3	-4.187096	7.11E-43
RASGEF1C	-4.188027	2.06E-09
ZNF471	-4.188662	5.86E-11
IGFBP5	-4.188744	0
SATB1	-4.189671	6.76E-58

NPC1L1	-4.189825	0.0002429
PURG	-4.193772	9.58E-16
SLIT2	-4.194737	1.13E-247
CCDC8	-4.195551	5.91E-118
TRMT9B	-4.205114	0.0000206
ENPP5	-4.206852	3.47E-15
PDLIM3	-4.213675	2.44E-23
CKMT1B	-4.213717	1.61E-20
RAB39A	-4.214159	5.91E-29
ZNF724	-4.220999	2.97E-61
ACBD7	-4.221906	1.5E-57
SLC37A2	-4.222392	0.0001807
COL24A1	-4.222392	0.0001054
HOXC12	-4.224966	0.0000015
AL391987.2	-4.227141	1.05E-44
NPTX2	-4.233035	2.53E-17
AMOT	-4.241119	7.67E-185
KLHL14	-4.247928	4.31E-07
SV2A	-4.249551	1.09E-128
BRINP3	-4.251539	5.07E-08
BCL11A	-4.25326	6.48E-68
RADX	-4.255535	0
BST2	-4.261378	4.07E-40
SH3GL2	-4.266787	1.34E-06
ENPP2	-4.269461	3.98E-52
HAPLN2	-4.271104	0.0000703
ATP8A1	-4.271673	1.45E-147
PCYT1B	-4.27244	1.16E-23
TBX21	-4.273018	0.0061773
SPDYC	-4.278775	5.07E-36
HBA1	-4.285931	8.68E-15
DTNA	-4.291676	8.39E-123
FXYD6	-4.294655	1.65E-72
HOXA9	-4.297741	1.2E-108
HIC1	-4.307429	6.02E-28
PRRT2	-4.310021	8.94E-32
APCDD1	-4.317413	3.76E-08
PHACTR3	-4.321928	0.0010683
SLITRK2	-4.321928	1.57E-06
ADGRB3	-4.321928	6.3E-14
HMX1	-4.321928	6.08E-28
TRPC3	-4.329921	4.77E-15
ILDR2	-4.332983	3.51E-26
KIAA1324L	-4.34016	3.89E-162
SLC35D3	-4.35435	5.14E-06
FREM2	-4.354617	8.29E-71
BICDL2	-4.355204	0.0000001
ARHGAP40	-4.357552	0.0010585
JPH1	-4.361821	4.43E-89
PRDX2	-4.361863	3.08E-245
ASXL3	-4.364572	2.1E-14
HOXD13	-4.367533	3.44E-164
PWP2	-4.369724	1.12E-48
LYPD6	-4.372292	2.57E-28
FAM184A	-4.374787	4.05E-87
ANGPTL2	-4.375039	4.08E-16
HLA-DPB1	-4.378512	6.62E-40
POU3F3	-4.380644	3.2E-37
NFATC4	-4.38202	1.09E-143
ERC2	-4.384664	5.55E-16
NAP1L3	-4.390633	1.54E-96
ZDHHC11B	-4.400879	0.000719
ZNF492	-4.401251	2.22E-19
NRN1	-4.406559	2.05E-173
FOXN4	-4.407376	6.86E-37
SULT4A1	-4.40913	4.26E-31
ITGAD	-4.409391	0.0002625
CCDC116	-4.409391	1.49E-07

C2orf92	-4.413851	0.0007305
DMRTA2	-4.415037	4.36E-15
RBM11	-4.416223	3.93E-22
SP8	-4.420957	2.36E-10
SLC1A3	-4.421464	3.01E-41
SLC35F1	-4.425404	1.87E-71
UGT3A2	-4.428192	5.21E-29
SAMD10	-4.433987	5.23E-17
ECEL1	-4.435462	7.49E-38
TSHZ2	-4.444785	8.08E-09
DES	-4.446256	0.0016917
PCDH19	-4.446256	1.76E-27
MPZ	-4.453458	0.0000742
FOXG1	-4.457749	6.95E-72
NEUROG2	-4.459432	0.0061823
DAAM2	-4.463284	7.52E-57
PCDH20	-4.464467	1.37E-36
RHCE	-4.468448	3.31E-11
SEMA3G	-4.469749	4.92E-17
TMSB15A	-4.472266	4.48E-15
BCL6B	-4.47978	9.35E-29
ADCYAP1R1	-4.481127	3.98E-35
GABRD	-4.482848	5.27E-10
COL25A1	-4.483392	8.44E-72
TDRD6	-4.496426	4.57E-13
ALDH1A2	-4.499592	1.68E-189
TSLP	-4.507795	6.54E-17
LPAR3	-4.51002	3.89E-85
BEND4	-4.53318	2.58E-152
CDKN2A	-4.533604	0
GAS1	-4.533779	4.34E-66
AL035425.2	-4.533876	3.7E-87
GLI3	-4.549569	1.29E-126
MAGEA6	-4.5556	1.17E-64
KCNC1	-4.557595	1.69E-73
ZNF577	-4.563332	2.24E-14
WNT8B	-4.566347	3.77E-07
DQX1	-4.576349	1.33E-12
ZNF91	-4.578658	4.21E-68
GLB1L3	-4.579801	7.51E-27
CFAP52	-4.584963	0.0053071
BLNK	-4.592457	0.0002356
MKRN2OS	-4.594947	0.0078476
SMIM10	-4.596409	4.56E-44
LURAP1	-4.601697	1.96E-11
ZNF85	-4.60456	2.18E-53
CCDC175	-4.604862	0.0023661
CBLN1	-4.604862	0.0021819
EBF2	-4.604862	2.68E-14
ZBED6CL	-4.608809	2.51E-103
RAMP1	-4.609416	5.78E-15
LRRC34	-4.613273	1.98E-30
UTS2R	-4.61471	0.0000146
SLC25A53	-4.61471	2.67E-17
CECR2	-4.618239	1.48E-125
ZNF681	-4.621621	3.41E-43
DYNC1I1	-4.622052	0.0000274
ZNF479	-4.627273	0.0004022
MAGEC2	-4.634206	0.0005334
ENPP3	-4.634206	0.0000114
ATAD3B	-4.643511	9.84E-80
ISL1	-4.643856	3.04E-15
CFAP221	-4.649615	5.36E-06
ITPRIPL1	-4.650551	9E-46
POU3F2	-4.659163	1.52E-77
MAGEB2	-4.66562	1.2E-54
ZNF423	-4.667425	5.07E-33
SERP2	-4.67948	0.0043625

SLC17A9	-4.70044	0.0010037
RAMP2	-4.70044	0.0000453
PROK2	-4.714246	0.0048811
MPZL2	-4.718818	0.0042456
LEF1	-4.719224	3.31E-131
INSM1	-4.724893	8.15E-13
OTOG	-4.72792	3.29E-10
DNAH8	-4.736966	0.0034823
SNX32	-4.736966	0.000004
CCDC177	-4.741467	0.0000416
POU3F1	-4.742964	4.83E-06
ATP2B3	-4.748193	1.57E-20
FOXI3	-4.748709	1.21E-18
FBN3	-4.754888	1.22E-06
PHYHD1	-4.759333	4.06E-152
CLUL1	-4.759967	1.17E-10
ADAMTS19	-4.761551	2.47E-21
CNPY1	-4.761766	1.36E-11
NELL2	-4.762975	1.94E-60
WNK3	-4.769202	9.09E-53
ZNF257	-4.780455	5.72E-24
MAFB	-4.797013	1.12E-22
PRMT6	-4.801073	2.09E-195
CDHR1	-4.807355	2.76E-32
TCEAL7	-4.814301	2.18E-11
SNCB	-4.822002	2.38E-20
CCDC140	-4.83289	0.0019894
SHISA3	-4.863208	1.32E-12
PCDHGC4	-4.867279	9.74E-16
SLC13A3	-4.868356	1.71E-56
ZNF736	-4.870148	2.63E-96
CKM	-4.870365	0.0025201
PDE1B	-4.870365	0.0000433
HLA-DRB1	-4.870878	8.44E-31
IRS4	-4.871738	0
SLC2A10	-4.873606	1.35E-21
ATG9B	-4.874469	0.0001162
ZNF888	-4.877744	2.11E-22
HLA-DOA	-4.877909	1.36E-91
GLIS1	-4.879146	1.98E-06
NUDT10	-4.882419	5E-54
AMPH	-4.891624	6.8E-16
RASGEF1B	-4.891888	1.32E-20
CGB2	-4.925179	1.26E-15
IRX6	-4.92837	8.96E-18
KLHL41	-4.929398	4.11E-34
RAB9B	-4.93002	7.57E-34
ZDHHC22	-4.931333	2.63E-96
METTL7A	-4.93615	7.56E-127
PRAC2	-4.941537	2.68E-08
RASL10B	-4.943296	3.49E-26
C1QTNF9B	-4.949959	0.00006
ZNF563	-4.954196	0.0002028
AC010330.1	-4.954196	0.0000697
NKX2-4	-4.954196	7.4E-18
RNF152	-4.961932	8.59E-12
P2RX2	-4.979494	9.61E-15
DAND5	-4.980548	0.0000612
ZNF501	-4.98225	5.33E-20
JAM2	-4.983808	9.66E-23
PDLIM4	-4.985203	1.03E-15
SIX2	-4.99271	1.48E-74
MKX	-5.006871	1.91E-56
STXBP5L	-5.01495	1.12E-06
C3orf70	-5.017922	2.58E-11
SULT2A1	-5.022368	0.0005114
NDST4	-5.022368	0.0000441
FER1L5	-5.044394	0.001081

ZNF738	-5.065291	3.05E-43
ZNF781	-5.087463	0.0001107
ZCCHC18	-5.097222	1.37E-12
BEX1	-5.097737	1.98E-50
PAX3	-5.102316	3.79E-40
MAB21L1	-5.108524	2.01E-08
ZNF429	-5.109245	1.9E-36
ZIC3	-5.114847	4.27E-55
COL6A5	-5.129283	3.78E-06
CDKN2B	-5.130999	4.19E-27
ZNF493	-5.13149	1.82E-45
MBNL3	-5.14714	1.25E-116
SLC13A4	-5.148183	8.2E-13
PCDH17	-5.149747	2.93E-18
SPINK5	-5.209453	0.001004
ZBTB8B	-5.220551	2.7E-56
STPG3	-5.247928	0.0003686
AC010487.3	-5.281286	6.69E-07
CELF3	-5.297681	0.0001937
LBX1	-5.324931	9.49E-13
UNCX	-5.333901	0.0004897
AQP6	-5.333901	0.0004232
CA3	-5.348728	0.0002588
PWWP3B	-5.352955	1.64E-32
FOXF1	-5.357552	1.47E-10
GZMM	-5.392317	0.0002974
GPC5	-5.416647	3.66E-07
XKR7	-5.437405	0.0090261
KCNA2	-5.437405	0.0086527
JAKMIP1	-5.442943	1.69E-12
GYPC	-5.454615	6.49E-28
LRRTM4	-5.456697	5.6E-11
DNAJC5G	-5.491853	0.0001742
FSIP2	-5.507795	1.14E-31
MYOZ1	-5.532495	2.11E-13
NT5C1A	-5.548437	0.0003908
CRYGD	-5.617511	0.0000017
ZNF385B	-5.633326	1.44E-12
PLCB2	-5.686501	0.0004367
LRRC9	-5.686501	1.44E-07
RPRM	-5.689045	5.25E-14
KLK1	-5.692491	0.0001099
TUBB8P12	-5.694648	3.22E-14
ZNF709	-5.710493	0.0008011
ICAM4	-5.711495	1.2E-11
ZNF676	-5.714246	8.46E-10
FAM13C	-5.736966	3.63E-09
NHLH2	-5.78136	0.0000557
ZNF785	-5.799758	9.11E-18
BARHL2	-5.807355	1.22E-06
FGF9	-5.807355	9.71E-07
GFRA3	-5.849666	0.0000677
RPRML	-5.857981	4.67E-07
ZNF100	-5.859822	1.46E-70
ZNF816-ZNF321P	-5.870365	6.8E-07
C11orf52	-5.878562	3.79E-07
TRIM58	-5.914883	2.45E-15
ZNF66	-5.946419	1.76E-12
ZBTB7C	-6.076816	1.75E-06
TLX3	-6.089818	7.69E-08
NCKAP1L	-6.108524	0.0001874
LRRC63	-6.266787	7.17E-06
ZNF660	-6.437405	0.0009212
LRFN5	-6.622052	3.32E-07
LRRC4	-6.768184	0.0000869
RAB6C	-6.78136	0.0083964
COL19A1	-6.807355	3.86E-08
HLA-DRB5	-6.847997	3.9E-07

PAK5	-6.946419	0.0003532
MSH4	-6.984893	0.0026744
GPR83	-7.022368	0.0048055
SSTR3	-7.022368	0.002092
DCAF8L1	-7.040016	2.59E-24
KRTAP19-1	-7.050211	1.17E-14
MDGA2	-7.058894	0.0002432
GAL3ST3	-7.228819	0.0011456
TNMD	-7.231821	1.12E-15
RSPO4	-7.321928	0.0039681
OXGR1	-7.409391	0.0048482
SYNPO2L	-7.437405	0.0000543
ZNF442	-7.467606	0.0001453
SLCO6A1	-7.491853	0.0067151
VGLL2	-7.491853	0.0063356
MMEL1	-7.518325	0.0046546
ARHGAP36	-7.544321	0.0010536
AC004233.2	-7.569856	0.0035562
KRT75	-7.643856	0.0020388
PLK5	-7.759333	0.0021511
ACTR3C	-7.794416	0.0005876
FRG2B	-7.803055	0.0095154
MS4A15	-7.824428	0.006544
OLIG1	-7.824428	0.0050129
CXorf67	-7.906891	0.0016394
VSTM2B	-7.946419	0.0048322
FCER2	-7.946419	0.0048055
PRR35	-7.965784	0.0004407
FGF23	-8.003752	0.0000611
CDK15	-8.003752	0.0000183
CTNND2	-8.076816	0.0063233
TAS1R1	-8.129283	0.0002818
NPFFR2	-8.196397	0.0018313
CYP2F1	-8.212699	0.0036157
LGI1	-8.260528	0.0055128
GPR32	-8.260528	0.0034823
LUZP4	-8.336878	0.0004487
HSFX4	-8.380822	0.0040741
CHST8	-8.491853	0.0000749
NELL1	-8.544321	5.22E-06
FOLR1	-8.569856	0.0064672
STMND1	-8.667703	0.0006361
CLDN5	-8.813781	0.0000127
CORT	-8.946419	0.0004421
XCL1	-9.040746	0.0001838
LCN6	-9.058894	0.0005502
NMRK2	-9.094518	0.0002746
METTL24	-9.268347	0.0001252
TMEM271	-9.464886	1.33E-07
LPL	-9.498517	0.000063
RUNX1T1	-9.70275	8.02E-07
UTF1	-9.770389	5.19E-06
TEX46	-9.866249	0.0014068
AMBN	-11.92308	6.58E-14
