# Supplementary material for: SNCA correlates with immune infiltration and serves as a prognostic biomarker in lung adenocarcinoma
Source: BMC Cancer. 2022 Apr 14;22:406. doi: 10.1186/s12885-022-09289-7 (PMC9009002; doi:10.1186/s12885-022-09289-7)
Supplement: Supplementary file 6 — Additional file 6. [file 12885_2022_9289_MOESM6_ESM.pdf]

| Category | Term | Count | % | PValue | List Total | Fold Enricl | FDR |
|---|---|---|---|---|---|---|---|
| KEGG_PA | hsa04060:Cytokine-cytokine receptor interaction | 61 | 17.37892 | 1.52E-34 | 254 | 6.798532 | 1.48E-32 |
| KEGG_PA | hsa05200:Pathways in cancer | 51 | 14.52991 | 2.60E-15 | 254 | 3.514546 | 4.20E-14 |
| KEGG_PA | hsa04151:PI3K-Akt signaling pathway | 45 | 12.82051 | 1.48E-13 | 254 | 3.532523 | 1.59E-12 |
| KEGG_PA | hsa04015:Rap1 signaling pathway | 41 | 11.68091 | 1.40E-18 | 254 | 5.28757 | 3.39E-17 |
| KEGG_PA | hsa04014:Ras signaling pathway | 41 | 11.68091 | 2.11E-17 | 254 | 4.913229 | 4.10E-16 |
| KEGG_PA | hsa05164:Influenza A | 40 | 11.39601 | 8.90E-21 | 254 | 6.225903 | 4.31E-19 |
| KEGG_PA | hsa04010:MAPK signaling pathway | 40 | 11.39601 | 8.23E-15 | 254 | 4.281846 | 9.97E-14 |
| KEGG_PA | hsa05166:HTLV-I infection | 35 | 9.97151 | 3.11E-11 | 254 | 3.731865 | 1.88E-10 |
| KEGG_PA | hsa05323:Rheumatoid arthritis | 29 | 8.262108 | 1.50E-19 | 254 | 8.924973 | 4.86E-18 |
| KEGG_PA | hsa04510:Focal adhesion | 29 | 8.262108 | 1.39E-09 | 254 | 3.81261 | 6.11E-09 |
| KEGG_PA | hsa04630:Jak-STAT signaling pathway | 28 | 7.977208 | 1.59E-12 | 254 | 5.229758 | 1.54E-11 |
| KEGG_PA | hsa05152:Tuberculosis | 28 | 7.977208 | 1.99E-10 | 254 | 4.284265 | 1.07E-09 |
| KEGG_PA | hsa05168:Herpes simplex infection | 28 | 7.977208 | 4.32E-10 | 254 | 4.143798 | 2.09E-09 |
| KEGG_PA | hsa04668:TNF signaling pathway | 27 | 7.692308 | 5.30E-15 | 254 | 6.833947 | 7.34E-14 |
| KEGG_PA | hsa04810:Regulation of actin cytoskeleton | 26 | 7.407407 | 1.63E-07 | 254 | 3.353093 | 4.61E-07 |
| KEGG_PA | hsa04360:Axon guidance | 25 | 7.122507 | 2.13E-11 | 254 | 5.331236 | 1.38E-10 |
| KEGG_PA | hsa05205:Proteoglycans in cancer | 25 | 7.122507 | 2.51E-07 | 254 | 3.385335 | 6.59E-07 |
| KEGG_PA | hsa04650:Natural killer cell mediated cytotoxicity | 24 | 6.837607 | 6.01E-11 | 254 | 5.32774 | 3.43E-10 |
| KEGG_PA | hsa05162:Measles | 24 | 6.837607 | 3.70E-10 | 254 | 4.8871 | 1.89E-09 |
| KEGG_PA | hsa05161:Hepatitis B | 24 | 6.837607 | 2.17E-09 | 254 | 4.48265 | 8.91E-09 |
| KEGG_PA | hsa04062:Chemokine signaling pathway | 23 | 6.552707 | 1.05E-06 | 254 | 3.348933 | 2.42E-06 |
| KEGG_PA | hsa04064:NF-kappa B signaling pathway | 22 | 6.267806 | 2.86E-12 | 254 | 6.848493 | 2.52E-11 |
| KEGG_PA | hsa04380:Osteoclast differentiation | 22 | 6.267806 | 9.23E-09 | 254 | 4.548236 | 3.44E-08 |
| KEGG_PA | hsa05145:Toxoplasmosis | 21 | 5.982906 | 2.20E-09 | 254 | 5.170329 | 8.91E-09 |
| KEGG_PA | hsa05169:Epstein-Barr virus infection | 21 | 5.982906 | 1.42E-08 | 254 | 4.661772 | 4.76E-08 |
| KEGG_PA | hsa04145:Phagosome | 21 | 5.982906 | 4.85E-07 | 254 | 3.791575 | 1.21E-06 |
| KEGG_PA | hsa05218:Melanoma | 20 | 5.698006 | 4.46E-12 | 254 | 7.628923 | 3.33E-11 |
| KEGG_PA | hsa05140:Leishmaniasis | 20 | 5.698006 | 4.46E-12 | 254 | 7.628923 | 3.33E-11 |
| KEGG_PA | hsa04620:Toll-like receptor signaling pathway | 19 | 5.413105 | 4.40E-08 | 254 | 4.854442 | 1.42E-07 |
| KEGG_PA | hsa04012:ErbB signaling pathway | 18 | 5.128205 | 1.19E-08 | 254 | 5.603313 | 4.11E-08 |
| KEGG_PA | hsa05142:Chagas disease (American trypanosomiasis) | 18 | 5.128205 | 1.88E-07 | 254 | 4.687386 | 5.06E-07 |
| KEGG_PA | hsa04550:Signaling pathways regulating pluripotency of stem cells | 18 | 5.128205 | 1.30E-05 | 254 | 3.482058 | 2.68E-05 |
| KEGG_PA | hsa04080:Neuroactive ligand-receptor interaction | 18 | 5.128205 | 0.025972 | 254 | 1.759885 | 0.028629 |
| KEGG_PA | hsa05144:Malaria | 17 | 4.843305 | 7.91E-12 | 254 | 9.396031 | 5.48E-11 |
| KEGG_PA | hsa04612:Antigen processing and presentation | 17 | 4.843305 | 1.05E-08 | 254 | 6.057967 | 3.76E-08 |
| KEGG_PA | hsa04640:Hematopoietic cell lineage | 17 | 4.843305 | 7.96E-08 | 254 | 5.292017 | 2.41E-07 |
| KEGG_PA | hsa05146:Amoebiasis | 17 | 4.843305 | 1.32E-06 | 254 | 4.343448 | 2.99E-06 |
| KEGG_PA | hsa05160:Hepatitis C | 17 | 4.843305 | 2.63E-05 | 254 | 3.461696 | 5.11E-05 |
| KEGG_PA | hsa04514:Cell adhesion molecules (CAMs) | 17 | 4.843305 | 5.94E-05 | 254 | 3.242292 | 1.05E-04 |
| KEGG_PA | hsa05134:Legionellosis | 16 | 4.558405 | 4.83E-10 | 254 | 8.024497 | 2.23E-09 |
| KEGG_PA | hsa05321:Inflammatory bowel disease (IBD) | 16 | 4.558405 | 6.40E-09 | 254 | 6.770669 | 2.48E-08 |
| KEGG_PA | hsa05202:Transcriptional misregulation in cancer | 16 | 4.558405 | 0.001182 | 254 | 2.594748 | 0.001616 |

| | | | | | | | |
|---|---|---|---|---|---|---|---|
| KEGG_PA | hsa05214:Glioma | 15 | 4.273504 | 6.59E-08 | 254 | 6.249849 | 2.06E-07 |
| KEGG_PA | hsa04066:HIF-1 signaling pathway | 15 | 4.273504 | 9.31E-06 | 254 | 4.231668 | 1.96E-05 |
| KEGG_PA | hsa05416:Viral myocarditis | 14 | 3.988604 | 9.56E-08 | 254 | 6.651886 | 2.81E-07 |
| KEGG_PA | hsa04662:B cell receptor signaling pathway | 14 | 3.988604 | 1.02E-06 | 254 | 5.495036 | 2.41E-06 |
| KEGG_PA | hsa04915:Estrogen signaling pathway | 14 | 3.988604 | 6.08E-05 | 254 | 3.829874 | 1.05E-04 |
| KEGG_PA | hsa05203:Viral carcinogenesis | 14 | 3.988604 | 0.038421 | 254 | 1.849549 | 0.040954 |
| KEGG_PA | hsa05150:Staphylococcus aureus infection | 13 | 3.703704 | 4.09E-07 | 254 | 6.519904 | 1.04E-06 |
| KEGG_PA | hsa04370:VEGF signaling pathway | 13 | 3.703704 | 1.65E-06 | 254 | 5.771718 | 3.64E-06 |
| KEGG_PA | hsa05212:Pancreatic cancer | 13 | 3.703704 | 3.35E-06 | 254 | 5.416535 | 7.22E-06 |
| KEGG_PA | hsa05133:Pertussis | 13 | 3.703704 | 1.57E-05 | 254 | 4.694331 | 3.16E-05 |
| KEGG_PA | hsa04350:TGF-beta signaling pathway | 13 | 3.703704 | 5.04E-05 | 254 | 4.191367 | 9.23E-05 |
| KEGG_PA | hsa04660:T cell receptor signaling pathway | 13 | 3.703704 | 2.78E-04 | 254 | 3.520748 | 4.42E-04 |
| KEGG_PA | hsa05231:Choline metabolism in cancer | 13 | 3.703704 | 3.06E-04 | 254 | 3.485889 | 4.78E-04 |
| KEGG_PA | hsa04071:Sphingolipid signaling pathway | 13 | 3.703704 | 0.001456 | 254 | 2.933957 | 0.001935 |
| KEGG_PA | hsa04068:FoxO signaling pathway | 13 | 3.703704 | 0.003696 | 254 | 2.627424 | 0.004538 |
| KEGG_PA | hsa04390:Hippo signaling pathway | 13 | 3.703704 | 0.009486 | 254 | 2.331621 | 0.011251 |
| KEGG_PA | hsa04672:Intestinal immune network for IgA production | 12 | 3.418803 | 7.21E-07 | 254 | 6.914726 | 1.75E-06 |
| KEGG_PA | hsa04664:Fc epsilon RI signaling pathway | 12 | 3.418803 | 3.20E-05 | 254 | 4.779296 | 5.97E-05 |
| KEGG_PA | hsa05220:Chronic myeloid leukemia | 12 | 3.418803 | 5.53E-05 | 254 | 4.51378 | 9.94E-05 |
| KEGG_PA | hsa05132:Salmonella infection | 12 | 3.418803 | 2.07E-04 | 254 | 3.915568 | 3.40E-04 |
| KEGG_PA | hsa05215:Prostate cancer | 12 | 3.418803 | 3.48E-04 | 254 | 3.693092 | 5.28E-04 |
| KEGG_PA | hsa05332:Graft-versus-host disease | 11 | 3.133903 | 1.66E-07 | 254 | 9.027559 | 4.61E-07 |
| KEGG_PA | hsa04621:NOD-like receptor signaling pathway | 11 | 3.133903 | 3.03E-05 | 254 | 5.319812 | 5.76E-05 |
| KEGG_PA | hsa04520:Adherens junction | 11 | 3.133903 | 2.42E-04 | 254 | 4.195908 | 3.91E-04 |
| KEGG_PA | hsa04666:Fc gamma R-mediated phagocytosis | 11 | 3.133903 | 9.51E-04 | 254 | 3.546541 | 0.001337 |
| KEGG_PA | hsa04750:Inflammatory mediator regulation of TRP channels | 11 | 3.133903 | 0.003077 | 254 | 3.039892 | 0.003827 |
| KEGG_PA | hsa04670:Leukocyte transendothelial migration | 11 | 3.133903 | 0.009511 | 254 | 2.590517 | 0.011251 |
| KEGG_PA | hsa04919:Thyroid hormone signaling pathway | 11 | 3.133903 | 0.009511 | 254 | 2.590517 | 0.011251 |
| KEGG_PA | hsa04722:Neurotrophin signaling pathway | 11 | 3.133903 | 0.012631 | 254 | 2.482579 | 0.014585 |
| KEGG_PA | hsa04932:Non-alcoholic fatty liver disease (NAFLD) | 11 | 3.133903 | 0.051166 | 254 | 1.97291 | 0.052244 |
| KEGG_PA | hsa04940:Type I diabetes mellitus | 10 | 2.849003 | 1.64E-05 | 254 | 6.448256 | 3.25E-05 |
| KEGG_PA | hsa05223:Non-small cell lung cancer | 10 | 2.849003 | 1.78E-04 | 254 | 4.836192 | 2.97E-04 |
| KEGG_PA | hsa05210:Colorectal cancer | 10 | 2.849003 | 3.93E-04 | 254 | 4.368174 | 5.86E-04 |
| KEGG_PA | hsa05211:Renal cell carcinoma | 10 | 2.849003 | 6.31E-04 | 254 | 4.103436 | 9.27E-04 |
| KEGG_PA | hsa04917:Prolactin signaling pathway | 10 | 2.849003 | 0.001081 | 254 | 3.814462 | 0.001498 |
| KEGG_PA | hsa05322:Systemic lupus erythematosus | 10 | 2.849003 | 0.058085 | 254 | 2.021095 | 0.058085 |
| KEGG_PA | hsa04210:Apoptosis | 9 | 2.564103 | 0.001812 | 254 | 3.931356 | 0.002376 |
| KEGG_PA | hsa05230:Central carbon metabolism in cancer | 9 | 2.564103 | 0.002227 | 254 | 3.808501 | 0.002843 |
| KEGG_PA | hsa04623:Cytosolic DNA-sensing pathway | 9 | 2.564103 | 0.002227 | 254 | 3.808501 | 0.002843 |
| KEGG_PA | hsa05143:African trypanosomiasis | 8 | 2.279202 | 1.58E-04 | 254 | 6.565497 | 2.68E-04 |
| KEGG_PA | hsa05330:Allograft rejection | 8 | 2.279202 | 3.35E-04 | 254 | 5.855714 | 5.15E-04 |
| KEGG_PA | hsa05219:Bladder cancer | 8 | 2.279202 | 6.45E-04 | 254 | 5.284425 | 9.33E-04 |
| KEGG_PA | hsa05320:Autoimmune thyroid disease | 8 | 2.279202 | 0.00272 | 254 | 4.166566 | 0.003427 |

| | | | | | | | |
|---|---|---|---|---|---|---|---|
| KEGG_PA | hsa05120:Epithelial cell signaling in Helicobacter pylori infection | 8 | 2.279202 | 0.011103 | 254 | 3.233752 | 0.012976 |
| KEGG_PA | hsa04610:Complement and coagulation cascades | 8 | 2.279202 | 0.01295 | 254 | 3.140021 | 0.014778 |
| KEGG_PA | hsa05100:Bacterial invasion of epithelial cells | 8 | 2.279202 | 0.024044 | 254 | 2.77771 | 0.026807 |
| KEGG_PA | hsa05222:Small cell lung cancer | 8 | 2.279202 | 0.036264 | 254 | 2.548958 | 0.039084 |
| KEGG_PA | hsa04540:Gap junction | 8 | 2.279202 | 0.042567 | 254 | 2.462062 | 0.044397 |
| KEGG_PA | hsa05310:Asthma | 7 | 1.994302 | 6.58E-04 | 254 | 6.319291 | 9.39E-04 |
| KEGG_PA | hsa05020:Prion diseases | 7 | 1.994302 | 0.001318 | 254 | 5.575845 | 0.001775 |
| KEGG_PA | hsa04622:RIG-I-like receptor signaling pathway | 7 | 1.994302 | 0.043071 | 254 | 2.708268 | 0.044446 |
| KEGG_PA | hsa04960:Aldosterone-regulated sodium reabsorption | 6 | 1.709402 | 0.013399 | 254 | 4.166566 | 0.015113 |
| KEGG_PA | hsa05213:Endometrial cancer | 6 | 1.709402 | 0.041258 | 254 | 3.124924 | 0.043501 |
| KEGG_PA | hsa05221:Acute myeloid leukemia | 6 | 1.709402 | 0.053966 | 254 | 2.901715 | 0.054528 |
| KEGG_PA | hsa04150:mTOR signaling pathway | 6 | 1.709402 | 0.061082 | 254 | 2.801656 | 0.061082 |
| KEGG_PA | hsa05340:Primary immunodeficiency | 5 | 1.424501 | 0.034867 | 254 | 3.982747 | 0.038001 |
| GOTERM_ | GO:0005515~protein binding | 234 | 66.66667 | 5.93E-09 | 348 | 1.292091 | 2.35E-07 |
| GOTERM_ | GO:0008083~growth factor activity | 51 | 14.52991 | 3.23E-45 | 348 | 15.27123 | 1.66E-42 |
| GOTERM_ | GO:0005125~cytokine activity | 40 | 11.39601 | 2.78E-29 | 348 | 11.02469 | 7.14E-27 |
| GOTERM_ | GO:0005102~receptor binding | 39 | 11.11111 | 4.35E-17 | 348 | 5.359309 | 7.45E-15 |
| GOTERM_ | GO:0042803~protein homodimerization activity | 33 | 9.401709 | 4.79E-05 | 348 | 2.192855 | 7.94E-04 |
| GOTERM_ | GO:0005088~Ras guanyl-nucleotide exchange factor activity | 24 | 6.837607 | 1.15E-16 | 348 | 10.12354 | 1.48E-14 |
| GOTERM_ | GO:0008201~heparin binding | 21 | 5.982906 | 1.11E-10 | 348 | 6.366756 | 5.18E-09 |
| GOTERM_ | GO:0004713~protein tyrosine kinase activity | 20 | 5.698006 | 3.09E-11 | 348 | 7.294529 | 1.59E-09 |
| GOTERM_ | GO:0005179~hormone activity | 18 | 5.128205 | 7.08E-12 | 348 | 9.288885 | 4.55E-10 |
| GOTERM_ | GO:0046982~protein heterodimerization activity | 17 | 4.843305 | 0.031524 | 348 | 1.773433 | 0.213201 |
| GOTERM_ | GO:0046934~phosphatidylinositol-4,5-bisphosphate 3-kinase activity | 16 | 4.558405 | 1.42E-12 | 348 | 12.51835 | 1.22E-10 |
| GOTERM_ | GO:0004872~receptor activity | 16 | 4.558405 | 4.51E-05 | 348 | 3.576672 | 7.73E-04 |
| GOTERM_ | GO:0004896~cytokine receptor activity | 15 | 4.273504 | 4.65E-15 | 348 | 20.21193 | 4.78E-13 |
| GOTERM_ | GO:0019899~enzyme binding | 15 | 4.273504 | 0.009443 | 348 | 2.185073 | 0.083684 |
| GOTERM_ | GO:0004888~transmembrane signaling receptor activity | 14 | 3.988604 | 5.08E-04 | 348 | 3.173461 | 0.006525 |
| GOTERM_ | GO:0045499~chemorepellent activity | 12 | 3.418803 | 2.30E-12 | 348 | 21.55939 | 1.69E-10 |
| GOTERM_ | GO:0031625~ubiquitin protein ligase binding | 12 | 3.418803 | 0.036331 | 348 | 2.028235 | 0.23941 |
| GOTERM_ | GO:0030215~semaphorin receptor binding | 11 | 3.133903 | 1.07E-11 | 348 | 23.19978 | 6.10E-10 |
| GOTERM_ | GO:0008009~chemokine activity | 11 | 3.133903 | 4.77E-08 | 348 | 10.88969 | 1.75E-06 |
| GOTERM_ | GO:0004871~signal transducer activity | 11 | 3.133903 | 0.009889 | 348 | 2.615661 | 0.085015 |
| GOTERM_ | GO:0019838~growth factor binding | 10 | 2.849003 | 2.00E-09 | 348 | 17.96616 | 8.56E-08 |
| GOTERM_ | GO:0001664~G-protein coupled receptor binding | 10 | 2.849003 | 1.63E-06 | 348 | 8.819749 | 4.42E-05 |
| GOTERM_ | GO:0003707~steroid hormone receptor activity | 10 | 2.849003 | 1.91E-06 | 348 | 8.662254 | 4.68E-05 |
| GOTERM_ | GO:0001948~glycoprotein binding | 10 | 2.849003 | 6.85E-06 | 348 | 7.462865 | 1.53E-04 |
| GOTERM_ | GO:0016301~kinase activity | 10 | 2.849003 | 0.062894 | 348 | 2.012806 | 0.340289 |
| GOTERM_ | GO:0004879~RNA polymerase II transcription factor activity, ligand-activated sequence-spe | 9 | 2.564103 | 5.38E-07 | 348 | 12.12716 | 1.73E-05 |
| GOTERM_ | GO:0005160~transforming growth factor beta receptor binding | 9 | 2.564103 | 1.89E-06 | 348 | 10.3947 | 4.68E-05 |
| GOTERM_ | GO:0004702~receptor signaling protein serine/threonine kinase activity | 9 | 2.564103 | 1.16E-05 | 348 | 8.237313 | 2.39E-04 |
| GOTERM_ | GO:0005178~integrin binding | 9 | 2.564103 | 0.001453 | 348 | 4.157882 | 0.0166 |
| GOTERM_ | GO:0042605~peptide antigen binding | 8 | 2.279202 | 1.19E-06 | 348 | 13.85961 | 3.60E-05 |

| | | | | | | | |
|---|---|---|---|---|---|---|---|
| GOTERM_ | GO:0016303~1-phosphatidylinositol-3-kinase activity | 8 | 2.279202 | 2.49E-05 | 348 | 9.02486 | 4.41E-04 |
| GOTERM_ | GO:0048306~calcium-dependent protein binding | 8 | 2.279202 | 1.78E-04 | 348 | 6.690844 | 0.002619 |
| GOTERM_ | GO:0038191~neuropilin binding | 7 | 1.994302 | 3.09E-07 | 348 | 22.63736 | 1.06E-05 |
| GOTERM_ | GO:0019955~cytokine binding | 7 | 1.994302 | 1.56E-06 | 348 | 17.8716 | 4.42E-05 |
| GOTERM_ | GO:0042056~chemoattractant activity | 7 | 1.994302 | 1.48E-05 | 348 | 12.57631 | 2.83E-04 |
| GOTERM_ | GO:0004714~transmembrane receptor protein tyrosine kinase activity | 7 | 1.994302 | 1.14E-04 | 348 | 8.935799 | 0.00173 |
| GOTERM_ | GO:0050839~cell adhesion molecule binding | 7 | 1.994302 | 0.001686 | 348 | 5.47678 | 0.018839 |
| GOTERM_ | GO:0002020~protease binding | 7 | 1.994302 | 0.017922 | 348 | 3.361984 | 0.129742 |
| GOTERM_ | GO:0032395~MHC class II receptor activity | 6 | 1.709402 | 9.04E-06 | 348 | 19.40345 | 1.94E-04 |
| GOTERM_ | GO:0050431~transforming growth factor beta binding | 6 | 1.709402 | 1.29E-05 | 348 | 18.19073 | 2.56E-04 |
| GOTERM_ | GO:0005031~tumor necrosis factor-activated receptor activity | 6 | 1.709402 | 1.10E-04 | 348 | 12.12716 | 0.001711 |
| GOTERM_ | GO:0005184~neuropeptide hormone activity | 6 | 1.709402 | 3.33E-04 | 348 | 9.701724 | 0.004502 |
| GOTERM_ | GO:0004715~non-membrane spanning protein tyrosine kinase activity | 6 | 1.709402 | 0.002448 | 348 | 6.327211 | 0.025286 |
| GOTERM_ | GO:0005044~scavenger receptor activity | 6 | 1.709402 | 0.002958 | 348 | 6.063578 | 0.029812 |
| GOTERM_ | GO:0001618~virus receptor activity | 6 | 1.709402 | 0.014517 | 348 | 4.157882 | 0.114797 |
| GOTERM_ | GO:0003823~antigen binding | 6 | 1.709402 | 0.061299 | 348 | 2.825745 | 0.338791 |
| GOTERM_ | GO:0005021~vascular endothelial growth factor-activated receptor activity | 5 | 1.424501 | 5.85E-06 | 348 | 34.64901 | 1.37E-04 |
| GOTERM_ | GO:0045236~CXCR chemokine receptor binding | 5 | 1.424501 | 2.04E-05 | 348 | 26.94923 | 3.74E-04 |
| GOTERM_ | GO:0017154~semaphorin receptor activity | 5 | 1.424501 | 7.62E-05 | 348 | 20.21193 | 0.001224 |
| GOTERM_ | GO:0005161~platelet-derived growth factor receptor binding | 5 | 1.424501 | 2.00E-04 | 348 | 16.16954 | 0.002858 |
| GOTERM_ | GO:0005154~epidermal growth factor receptor binding | 5 | 1.424501 | 0.003561 | 348 | 7.823971 | 0.035203 |
| GOTERM_ | GO:0005518~collagen binding | 5 | 1.424501 | 0.034783 | 348 | 4.042385 | 0.232187 |
| GOTERM_ | GO:0000980~RNA polymerase II distal enhancer sequence-specific DNA binding | 5 | 1.424501 | 0.044646 | 348 | 3.731432 | 0.279853 |
| GOTERM_ | GO:0008329~signaling pattern recognition receptor activity | 4 | 1.139601 | 2.83E-04 | 348 | 27.71921 | 0.003937 |
| GOTERM_ | GO:0005501~retinoid binding | 4 | 1.139601 | 4.47E-04 | 348 | 24.25431 | 0.005886 |
| GOTERM_ | GO:0004675~transmembrane receptor protein serine/threonine kinase activity | 4 | 1.139601 | 6.60E-04 | 348 | 21.55939 | 0.00827 |
| GOTERM_ | GO:0016918~retinal binding | 4 | 1.139601 | 9.28E-04 | 348 | 19.40345 | 0.011094 |
| GOTERM_ | GO:0039706~co-receptor binding | 4 | 1.139601 | 9.28E-04 | 348 | 19.40345 | 0.011094 |
| GOTERM_ | GO:0043394~proteoglycan binding | 4 | 1.139601 | 0.001257 | 348 | 17.6395 | 0.014681 |
| GOTERM_ | GO:0044548~S100 protein binding | 4 | 1.139601 | 0.002113 | 348 | 14.92573 | 0.022626 |
| GOTERM_ | GO:0019841~retinol binding | 4 | 1.139601 | 0.002113 | 348 | 14.92573 | 0.022626 |
| GOTERM_ | GO:0005104~fibroblast growth factor receptor binding | 4 | 1.139601 | 0.009924 | 348 | 8.819749 | 0.085015 |
| GOTERM_ | GO:0017134~fibroblast growth factor binding | 4 | 1.139601 | 0.011241 | 348 | 8.436282 | 0.093195 |
| GOTERM_ | GO:0003785~actin monomer binding | 4 | 1.139601 | 0.015774 | 348 | 7.462865 | 0.119233 |
| GOTERM_ | GO:0001968~fibronectin binding | 4 | 1.139601 | 0.015774 | 348 | 7.462865 | 0.119233 |
| GOTERM_ | GO:0050840~extracellular matrix binding | 4 | 1.139601 | 0.015774 | 348 | 7.462865 | 0.119233 |
| GOTERM_ | GO:0017046~peptide hormone binding | 4 | 1.139601 | 0.017481 | 348 | 7.186462 | 0.12836 |
| GOTERM_ | GO:0005164~tumor necrosis factor receptor binding | 4 | 1.139601 | 0.021192 | 348 | 6.690844 | 0.151286 |
| GOTERM_ | GO:0005158~insulin receptor binding | 4 | 1.139601 | 0.0253 | 348 | 6.259177 | 0.173389 |
| GOTERM_ | GO:0030971~receptor tyrosine kinase binding | 4 | 1.139601 | 0.058117 | 348 | 4.51243 | 0.337517 |
| GOTERM_ | GO:0046332~SMAD binding | 4 | 1.139601 | 0.058117 | 348 | 4.51243 | 0.337517 |
| GOTERM_ | GO:0005080~protein kinase C binding | 4 | 1.139601 | 0.068397 | 348 | 4.218141 | 0.366209 |
| GOTERM_ | GO:0097110~scaffold protein binding | 4 | 1.139601 | 0.075676 | 348 | 4.042385 | 0.375969 |

| | | | | | | | |
|---|---|---|---|---|---|---|---|
| GOTERM_ | GO:0034714~type III transforming growth factor beta receptor binding | 3 | 0.854701 | 0.00246 | 348 | 36.38147 | 0.025286 |
| GOTERM_ | GO:0005127~ciliary neurotrophic factor receptor binding | 3 | 0.854701 | 0.004044 | 348 | 29.10517 | 0.039217 |
| GOTERM_ | GO:0055131~C3HC4-type RING finger domain binding | 3 | 0.854701 | 0.005983 | 348 | 24.25431 | 0.056954 |
| GOTERM_ | GO:0005024~transforming growth factor beta-activated receptor activity | 3 | 0.854701 | 0.008263 | 348 | 20.78941 | 0.074516 |
| GOTERM_ | GO:0005138~interleukin-6 receptor binding | 3 | 0.854701 | 0.008263 | 348 | 20.78941 | 0.074516 |
| GOTERM_ | GO:0005114~type II transforming growth factor beta receptor binding | 3 | 0.854701 | 0.008263 | 348 | 20.78941 | 0.074516 |
| GOTERM_ | GO:0071723~lipopeptide binding | 3 | 0.854701 | 0.010869 | 348 | 18.19073 | 0.091586 |
| GOTERM_ | GO:0046703~natural killer cell lectin-like receptor binding | 3 | 0.854701 | 0.013786 | 348 | 16.16954 | 0.110719 |
| GOTERM_ | GO:0034713~type I transforming growth factor beta receptor binding | 3 | 0.854701 | 0.013786 | 348 | 16.16954 | 0.110719 |
| GOTERM_ | GO:0051428~peptide hormone receptor binding | 3 | 0.854701 | 0.017001 | 348 | 14.55259 | 0.126642 |
| GOTERM_ | GO:0000975~regulatory region DNA binding | 3 | 0.854701 | 0.024269 | 348 | 12.12716 | 0.168571 |
| GOTERM_ | GO:0048185~activin binding | 3 | 0.854701 | 0.024269 | 348 | 12.12716 | 0.168571 |
| GOTERM_ | GO:0008528~G-protein coupled peptide receptor activity | 3 | 0.854701 | 0.051906 | 348 | 8.08477 | 0.321442 |
| GOTERM_ | GO:0070530~K63-linked polyubiquitin binding | 3 | 0.854701 | 0.057244 | 348 | 7.659256 | 0.337517 |
| GOTERM_ | GO:0042923~neuropeptide binding | 3 | 0.854701 | 0.062763 | 348 | 7.276293 | 0.340289 |
| GOTERM_ | GO:0001530~lipopolysaccharide binding | 3 | 0.854701 | 0.074307 | 348 | 6.614812 | 0.375969 |
| GOTERM_ | GO:0004709~MAP kinase kinase kinase activity | 3 | 0.854701 | 0.074307 | 348 | 6.614812 | 0.375969 |
| GOTERM_ | GO:0046875~ephrin receptor binding | 3 | 0.854701 | 0.099165 | 348 | 5.597149 | 0.447112 |
| GOTERM_ | GO:0005146~leukemia inhibitory factor receptor binding | 2 | 0.569801 | 0.04069 | 348 | 48.50862 | 0.258205 |
| GOTERM_ | GO:0035403~histone kinase activity (H3-T6 specific) | 2 | 0.569801 | 0.04069 | 348 | 48.50862 | 0.258205 |
| GOTERM_ | GO:0043185~vascular endothelial growth factor receptor 3 binding | 2 | 0.569801 | 0.04069 | 348 | 48.50862 | 0.258205 |
| GOTERM_ | GO:0031708~endothelin B receptor binding | 2 | 0.569801 | 0.060412 | 348 | 32.33908 | 0.337517 |
| GOTERM_ | GO:0004999~vasoactive intestinal polypeptide receptor activity | 2 | 0.569801 | 0.060412 | 348 | 32.33908 | 0.337517 |
| GOTERM_ | GO:0004517~nitric-oxide synthase activity | 2 | 0.569801 | 0.060412 | 348 | 32.33908 | 0.337517 |
| GOTERM_ | GO:0004924~oncostatin-M receptor activity | 2 | 0.569801 | 0.060412 | 348 | 32.33908 | 0.337517 |
| GOTERM_ | GO:0030369~ICAM-3 receptor activity | 2 | 0.569801 | 0.060412 | 348 | 32.33908 | 0.337517 |
| GOTERM_ | GO:0005010~insulin-like growth factor-activated receptor activity | 2 | 0.569801 | 0.060412 | 348 | 32.33908 | 0.337517 |
| GOTERM_ | GO:0035325~Toll-like receptor binding | 2 | 0.569801 | 0.079729 | 348 | 24.25431 | 0.375969 |
| GOTERM_ | GO:0034617~tetrahydrobiopterin binding | 2 | 0.569801 | 0.079729 | 348 | 24.25431 | 0.375969 |
| GOTERM_ | GO:0070851~growth factor receptor binding | 2 | 0.569801 | 0.079729 | 348 | 24.25431 | 0.375969 |
| GOTERM_ | GO:0004698~calcium-dependent protein kinase C activity | 2 | 0.569801 | 0.079729 | 348 | 24.25431 | 0.375969 |
| GOTERM_ | GO:0004897~ciliary neurotrophic factor receptor activity | 2 | 0.569801 | 0.079729 | 348 | 24.25431 | 0.375969 |
| GOTERM_ | GO:0005025~transforming growth factor beta receptor activity, type I | 2 | 0.569801 | 0.079729 | 348 | 24.25431 | 0.375969 |
| GOTERM_ | GO:0016361~activin receptor activity, type I | 2 | 0.569801 | 0.079729 | 348 | 24.25431 | 0.375969 |
| GOTERM_ | GO:0005172~vascular endothelial growth factor receptor binding | 2 | 0.569801 | 0.079729 | 348 | 24.25431 | 0.375969 |
| GOTERM_ | GO:0048408~epidermal growth factor binding | 2 | 0.569801 | 0.079729 | 348 | 24.25431 | 0.375969 |
| GOTERM_ | GO:0005534~galactose binding | 2 | 0.569801 | 0.079729 | 348 | 24.25431 | 0.375969 |
| GOTERM_ | GO:0001875~lipopolysaccharide receptor activity | 2 | 0.569801 | 0.09865 | 348 | 19.40345 | 0.447112 |
| GOTERM_ | GO:0005007~fibroblast growth factor-activated receptor activity | 2 | 0.569801 | 0.09865 | 348 | 19.40345 | 0.447112 |
| GOTERM_ | GO:0042015~interleukin-20 binding | 2 | 0.569801 | 0.09865 | 348 | 19.40345 | 0.447112 |
| GOTERM_ | GO:0004957~prostaglandin E receptor activity | 2 | 0.569801 | 0.09865 | 348 | 19.40345 | 0.447112 |
| GOTERM_ | GO:0005886~plasma membrane | 149 | 42.45014 | 3.56E-17 | 349 | 1.888 | 2.31E-15 |
| GOTERM_ | GO:0005576~extracellular region | 141 | 40.17094 | 6.07E-58 | 349 | 4.573109 | 1.58E-55 |

| | | | | | | | |
|---|---|---|---|---|---|---|---|
| GOTERM_ | GO:0005615~extracellular space | 130 | 37.03704 | 1.36E-57 | 349 | 5.039576 | 1.77E-55 |
| GOTERM_ | GO:0016021~integral component of membrane | 112 | 31.90883 | 0.077449 | 349 | 1.13275 | 0.327941 |
| GOTERM_ | GO:0070062~extracellular exosome | 82 | 23.36182 | 6.84E-05 | 349 | 1.52325 | 0.001111 |
| GOTERM_ | GO:0005887~integral component of plasma membrane | 76 | 21.65242 | 3.00E-16 | 349 | 2.804629 | 1.56E-14 |
| GOTERM_ | GO:0005829~cytosol | 75 | 21.36752 | 0.078201 | 349 | 1.181397 | 0.327941 |
| GOTERM_ | GO:0016020~membrane | 66 | 18.80342 | 2.12E-04 | 349 | 1.566533 | 0.002899 |
| GOTERM_ | GO:0009986~cell surface | 54 | 15.38462 | 5.18E-23 | 349 | 5.202508 | 4.49E-21 |
| GOTERM_ | GO:0005622~intracellular | 44 | 12.53561 | 4.97E-04 | 349 | 1.724911 | 0.006156 |
| GOTERM_ | GO:0048471~perinuclear region of cytoplasm | 32 | 9.116809 | 1.15E-06 | 349 | 2.690771 | 2.99E-05 |
| GOTERM_ | GO:0009897~external side of plasma membrane | 23 | 6.552707 | 1.38E-10 | 349 | 5.638538 | 5.14E-09 |
| GOTERM_ | GO:0005925~focal adhesion | 22 | 6.267806 | 2.21E-05 | 349 | 2.938084 | 4.10E-04 |
| GOTERM_ | GO:0043235~receptor complex | 19 | 5.413105 | 3.63E-11 | 349 | 7.812107 | 1.57E-09 |
| GOTERM_ | GO:0000139~Golgi membrane | 19 | 5.413105 | 0.03643 | 349 | 1.678744 | 0.209133 |
| GOTERM_ | GO:0045121~membrane raft | 18 | 5.128205 | 4.73E-07 | 349 | 4.562717 | 1.37E-05 |
| GOTERM_ | GO:0005788~endoplasmic reticulum lumen | 13 | 3.703704 | 3.30E-04 | 349 | 3.535578 | 0.004295 |
| GOTERM_ | GO:0030424~axon | 13 | 3.703704 | 0.001198 | 349 | 3.057797 | 0.011982 |
| GOTERM_ | GO:0010008~endosome membrane | 12 | 3.418803 | 8.79E-04 | 349 | 3.387098 | 0.009527 |
| GOTERM_ | GO:0005768~endosome | 12 | 3.418803 | 0.004099 | 349 | 2.784947 | 0.034382 |
| GOTERM_ | GO:0043025~neuronal cell body | 12 | 3.418803 | 0.039965 | 349 | 1.989248 | 0.212061 |
| GOTERM_ | GO:0031093~platelet alpha granule lumen | 11 | 3.133903 | 7.73E-08 | 349 | 10.44355 | 2.51E-06 |
| GOTERM_ | GO:0005796~Golgi lumen | 10 | 2.849003 | 9.17E-05 | 349 | 5.439351 | 0.001402 |
| GOTERM_ | GO:0072562~blood microparticle | 10 | 2.849003 | 0.002618 | 349 | 3.435379 | 0.023473 |
| GOTERM_ | GO:0043005~neuron projection | 10 | 2.849003 | 0.038609 | 349 | 2.203281 | 0.209133 |
| GOTERM_ | GO:0005765~lysosomal membrane | 10 | 2.849003 | 0.080243 | 349 | 1.905758 | 0.331163 |
| GOTERM_ | GO:0012507~ER to Golgi transport vesicle membrane | 9 | 2.564103 | 5.90E-06 | 349 | 9.03769 | 1.28E-04 |
| GOTERM_ | GO:0030666~endocytic vesicle membrane | 9 | 2.564103 | 3.57E-05 | 349 | 7.120604 | 6.19E-04 |
| GOTERM_ | GO:0005764~lysosome | 9 | 2.564103 | 0.068647 | 349 | 2.079469 | 0.318719 |
| GOTERM_ | GO:0005769~early endosome | 9 | 2.564103 | 0.072924 | 349 | 2.052227 | 0.321657 |
| GOTERM_ | GO:0030141~secretory granule | 8 | 2.279202 | 5.24E-04 | 349 | 5.645164 | 0.006189 |
| GOTERM_ | GO:0042613~MHC class II protein complex | 7 | 1.994302 | 2.68E-06 | 349 | 16.61474 | 6.33E-05 |
| GOTERM_ | GO:0071556~integral component of lumenal side of endoplasmic reticulum membrane | 7 | 1.994302 | 1.52E-05 | 349 | 12.60429 | 3.04E-04 |
| GOTERM_ | GO:0030669~clathrin-coated endocytic vesicle membrane | 7 | 1.994302 | 1.19E-04 | 349 | 8.915228 | 0.001716 |
| GOTERM_ | GO:0005901~caveola | 7 | 1.994302 | 0.001487 | 349 | 5.623452 | 0.014319 |
| GOTERM_ | GO:0031234~extrinsic component of cytoplasmic side of plasma membrane | 7 | 1.994302 | 0.001879 | 349 | 5.375358 | 0.017444 |
| GOTERM_ | GO:0032588~trans-Golgi network membrane | 7 | 1.994302 | 0.005108 | 349 | 4.403908 | 0.041501 |
| GOTERM_ | GO:0030658~transport vesicle membrane | 6 | 1.709402 | 7.38E-04 | 349 | 8.24491 | 0.008341 |
| GOTERM_ | GO:0044297~cell body | 6 | 1.709402 | 0.007014 | 349 | 4.97312 | 0.055262 |
| GOTERM_ | GO:0043202~lysosomal lumen | 6 | 1.709402 | 0.023374 | 349 | 3.68596 | 0.164251 |
| GOTERM_ | GO:0030133~transport vesicle | 6 | 1.709402 | 0.035477 | 349 | 3.297964 | 0.209133 |
| GOTERM_ | GO:0005623~cell | 6 | 1.709402 | 0.042746 | 349 | 3.133066 | 0.222279 |
| GOTERM_ | GO:0031941~filamentous actin | 5 | 1.424501 | 0.002736 | 349 | 8.42222 | 0.023715 |
| GOTERM_ | GO:0030670~phagocytic vesicle membrane | 5 | 1.424501 | 0.026136 | 349 | 4.425234 | 0.178824 |
| GOTERM_ | GO:0005884~actin filament | 5 | 1.424501 | 0.03561 | 349 | 4.016751 | 0.209133 |

| | | | | | | |
|---|---|---|---|---|---|---|
| GOTERM_ | GO:0042995~cell projection | 5 | 1.424501 | 0.055273 | 349 | 3.481184 | 0.265688 |
| GOTERM_ | GO:0002116~semaphorin receptor complex | 4 | 1.139601 | 0.001016 | 349 | 18.98828 | 0.010571 |
| GOTERM_ | GO:0031226~intrinsic component of plasma membrane | 4 | 1.139601 | 0.015884 | 349 | 7.459681 | 0.117993 |
| GOTERM_ | GO:1990712~HFE-transferrin receptor complex | 3 | 0.854701 | 0.009435 | 349 | 19.58166 | 0.072149 |
| GOTERM_ | GO:0042612~MHC class I protein complex | 3 | 0.854701 | 0.017845 | 349 | 14.24121 | 0.128884 |
| GOTERM_ | GO:0031988~membrane-bounded vesicle | 3 | 0.854701 | 0.028436 | 349 | 11.18952 | 0.189575 |
| GOTERM_ | GO:0008305~integrin complex | 3 | 0.854701 | 0.093389 | 349 | 5.801974 | 0.379394 |
| GOTERM_ | GO:0070435~Shc-EGFR complex | 2 | 0.569801 | 0.037828 | 349 | 52.21777 | 0.209133 |
| GOTERM_ | GO:0034687~integrin alphaL-beta2 complex | 2 | 0.569801 | 0.037828 | 349 | 52.21777 | 0.209133 |
| GOTERM_ | GO:0097059~CNTFR-CLCF1 complex | 2 | 0.569801 | 0.037828 | 349 | 52.21777 | 0.209133 |
| GOTERM_ | GO:0035354~Toll-like receptor 1-Toll-like receptor 2 protein complex | 2 | 0.569801 | 0.037828 | 349 | 52.21777 | 0.209133 |
| GOTERM_ | GO:0005900~oncostatin-M receptor complex | 2 | 0.569801 | 0.037828 | 349 | 52.21777 | 0.209133 |
| GOTERM_ | GO:0097058~CRLF-CLCF1 complex | 2 | 0.569801 | 0.056203 | 349 | 34.81184 | 0.265688 |
| GOTERM_ | GO:0070110~ciliary neurotrophic factor receptor complex | 2 | 0.569801 | 0.056203 | 349 | 34.81184 | 0.265688 |
| GOTERM_ | GO:0005896~interleukin-6 receptor complex | 2 | 0.569801 | 0.056203 | 349 | 34.81184 | 0.265688 |
| GOTERM_ | GO:0048237~rough endoplasmic reticulum lumen | 2 | 0.569801 | 0.056203 | 349 | 34.81184 | 0.265688 |
| GOTERM_ | GO:0036021~endolysosome lumen | 2 | 0.569801 | 0.074229 | 349 | 26.10888 | 0.321657 |
| GOTERM_ | GO:0035867~alphav-beta3 integrin-IGF-1-IGF1R complex | 2 | 0.569801 | 0.074229 | 349 | 26.10888 | 0.321657 |
| GOTERM_ | GO:0070022~transforming growth factor beta receptor homodimeric complex | 2 | 0.569801 | 0.074229 | 349 | 26.10888 | 0.321657 |
| GOTERM_ | GO:0007165~signal transduction | 79 | 22.50712 | 3.57E-21 | 348 | 3.283357 | 1.75E-18 |
| GOTERM_ | GO:0008284~positive regulation of cell proliferation | 63 | 17.94872 | 1.31E-32 | 348 | 6.523457 | 1.60E-29 |
| GOTERM_ | GO:0006954~inflammatory response | 59 | 16.80912 | 7.45E-34 | 348 | 7.511661 | 1.82E-30 |
| GOTERM_ | GO:0006955~immune response | 58 | 16.52422 | 2.24E-30 | 348 | 6.647664 | 1.83E-27 |
| GOTERM_ | GO:0045944~positive regulation of transcription from RNA polymerase II promoter | 50 | 14.24501 | 8.27E-09 | 348 | 2.459372 | 4.93E-07 |
| GOTERM_ | GO:0007267~cell-cell signaling | 42 | 11.96581 | 5.64E-25 | 348 | 7.978822 | 3.44E-22 |
| GOTERM_ | GO:0000165~MAPK cascade | 37 | 10.54131 | 1.23E-19 | 348 | 6.814337 | 4.29E-17 |
| GOTERM_ | GO:0007186~G-protein coupled receptor signaling pathway | 37 | 10.54131 | 1.14E-04 | 348 | 1.985936 | 0.002242 |
| GOTERM_ | GO:0001525~angiogenesis | 36 | 10.25641 | 4.87E-21 | 348 | 7.789702 | 1.98E-18 |
| GOTERM_ | GO:0045087~innate immune response | 33 | 9.401709 | 3.92E-10 | 348 | 3.703128 | 2.82E-08 |
| GOTERM_ | GO:0008285~negative regulation of cell proliferation | 32 | 9.116809 | 2.15E-10 | 348 | 3.899222 | 1.59E-08 |
| GOTERM_ | GO:0043547~positive regulation of GTPase activity | 30 | 8.547009 | 6.78E-06 | 348 | 2.562099 | 1.97E-04 |
| GOTERM_ | GO:0007166~cell surface receptor signaling pathway | 29 | 8.262108 | 3.27E-12 | 348 | 5.107056 | 3.81E-10 |
| GOTERM_ | GO:0070374~positive regulation of ERK1 and ERK2 cascade | 28 | 7.977208 | 2.70E-16 | 348 | 7.72046 | 5.50E-14 |
| GOTERM_ | GO:0043066~negative regulation of apoptotic process | 28 | 7.977208 | 9.61E-07 | 348 | 2.969408 | 3.61E-05 |
| GOTERM_ | GO:0006915~apoptotic process | 28 | 7.977208 | 5.22E-05 | 348 | 2.382858 | 0.00113 |
| GOTERM_ | GO:0045893~positive regulation of transcription, DNA-templated | 27 | 7.692308 | 2.71E-05 | 348 | 2.529762 | 6.36E-04 |
| GOTERM_ | GO:0045766~positive regulation of angiogenesis | 26 | 7.407407 | 7.04E-19 | 348 | 10.90935 | 2.15E-16 |
| GOTERM_ | GO:0030335~positive regulation of cell migration | 26 | 7.407407 | 8.00E-14 | 348 | 6.818341 | 1.22E-11 |
| GOTERM_ | GO:0019221~cytokine-mediated signaling pathway | 25 | 7.122507 | 2.29E-16 | 348 | 9.208564 | 5.08E-14 |
| GOTERM_ | GO:0032496~response to lipopolysaccharide | 24 | 6.837607 | 4.10E-13 | 348 | 7.061396 | 5.57E-11 |
| GOTERM_ | GO:0042127~regulation of cell proliferation | 24 | 6.837607 | 5.35E-12 | 348 | 6.259832 | 5.94E-10 |
| GOTERM_ | GO:0042493~response to drug | 23 | 6.552707 | 3.85E-07 | 348 | 3.650711 | 1.68E-05 |
| GOTERM_ | GO:0008283~cell proliferation | 23 | 6.552707 | 8.10E-06 | 348 | 3.032284 | 2.33E-04 |

| | | | | | | | |
|---|---|---|---|---|---|---|---|
| GOTERM_ | GO:0006935~chemotaxis | 21 | 5.982906 | 7.27E-13 | 348 | 8.305822 | 9.36E-11 |
| GOTERM_ | GO:0018108~peptidyl-tyrosine phosphorylation | 21 | 5.982906 | 5.34E-11 | 348 | 6.622943 | 4.83E-09 |
| GOTERM_ | GO:0001666~response to hypoxia | 21 | 5.982906 | 4.51E-10 | 348 | 5.891339 | 3.15E-08 |
| GOTERM_ | GO:0042981~regulation of apoptotic process | 21 | 5.982906 | 1.90E-08 | 348 | 4.757326 | 1.03E-06 |
| GOTERM_ | GO:0010628~positive regulation of gene expression | 21 | 5.982906 | 5.68E-07 | 348 | 3.867597 | 2.31E-05 |
| GOTERM_ | GO:0007399~nervous system development | 21 | 5.982906 | 2.35E-06 | 348 | 3.530698 | 8.15E-05 |
| GOTERM_ | GO:0035556~intracellular signal transduction | 21 | 5.982906 | 2.89E-04 | 348 | 2.514418 | 0.004775 |
| GOTERM_ | GO:0007155~cell adhesion | 21 | 5.982906 | 0.001452 | 348 | 2.207648 | 0.018292 |
| GOTERM_ | GO:0030154~cell differentiation | 21 | 5.982906 | 0.001571 | 348 | 2.193312 | 0.019489 |
| GOTERM_ | GO:0006468~protein phosphorylation | 20 | 5.698006 | 0.003113 | 348 | 2.116354 | 0.032538 |
| GOTERM_ | GO:0048015~phosphatidylinositol-mediated signaling | 19 | 5.413105 | 5.64E-12 | 348 | 8.6491 | 5.99E-10 |
| GOTERM_ | GO:0007411~axon guidance | 19 | 5.413105 | 5.24E-09 | 348 | 5.766067 | 3.37E-07 |
| GOTERM_ | GO:0051781~positive regulation of cell division | 18 | 5.128205 | 2.33E-17 | 348 | 18.47982 | 6.34E-15 |
| GOTERM_ | GO:0007169~transmembrane receptor protein tyrosine kinase signaling pathway | 18 | 5.128205 | 1.10E-11 | 348 | 9.047414 | 1.07E-09 |
| GOTERM_ | GO:0000187~activation of MAPK activity | 18 | 5.128205 | 6.64E-11 | 348 | 8.117306 | 5.61E-09 |
| GOTERM_ | GO:0043065~positive regulation of apoptotic process | 18 | 5.128205 | 1.78E-04 | 348 | 2.895172 | 0.003276 |
| GOTERM_ | GO:0007275~multicellular organism development | 18 | 5.128205 | 0.043977 | 348 | 1.667086 | 0.23467 |
| GOTERM_ | GO:0048661~positive regulation of smooth muscle cell proliferation | 17 | 4.843305 | 5.14E-14 | 348 | 13.67165 | 8.38E-12 |
| GOTERM_ | GO:0060333~interferon-gamma-mediated signaling pathway | 17 | 4.843305 | 8.95E-13 | 348 | 11.5535 | 1.09E-10 |
| GOTERM_ | GO:0007204~positive regulation of cytosolic calcium ion concentration | 17 | 4.843305 | 1.75E-08 | 348 | 6.121633 | 9.71E-07 |
| GOTERM_ | GO:0050919~negative chemotaxis | 16 | 4.558405 | 5.03E-17 | 348 | 22.70723 | 1.23E-14 |
| GOTERM_ | GO:0014066~regulation of phosphatidylinositol 3-kinase signaling | 16 | 4.558405 | 5.32E-11 | 348 | 9.898025 | 4.83E-09 |
| GOTERM_ | GO:0050731~positive regulation of peptidyl-tyrosine phosphorylation | 16 | 4.558405 | 1.13E-10 | 348 | 9.415195 | 8.88E-09 |
| GOTERM_ | GO:0046854~phosphatidylinositol phosphorylation | 16 | 4.558405 | 8.40E-10 | 348 | 8.213255 | 5.70E-08 |
| GOTERM_ | GO:0033209~tumor necrosis factor-mediated signaling pathway | 16 | 4.558405 | 2.09E-08 | 348 | 6.542763 | 1.11E-06 |
| GOTERM_ | GO:0016477~cell migration | 16 | 4.558405 | 2.93E-06 | 348 | 4.488639 | 9.69E-05 |
| GOTERM_ | GO:0030198~extracellular matrix organization | 16 | 4.558405 | 1.45E-05 | 348 | 3.93901 | 3.76E-04 |
| GOTERM_ | GO:0001755~neural crest cell migration | 15 | 4.273504 | 1.87E-13 | 348 | 16.08429 | 2.68E-11 |
| GOTERM_ | GO:0001938~positive regulation of endothelial cell proliferation | 15 | 4.273504 | 1.10E-10 | 348 | 10.48976 | 8.88E-09 |
| GOTERM_ | GO:0042060~wound healing | 15 | 4.273504 | 8.77E-10 | 348 | 9.047414 | 5.80E-08 |
| GOTERM_ | GO:0051607~defense response to virus | 15 | 4.273504 | 8.62E-06 | 348 | 4.386625 | 2.45E-04 |
| GOTERM_ | GO:0007507~heart development | 15 | 4.273504 | 2.80E-05 | 348 | 3.955154 | 6.52E-04 |
| GOTERM_ | GO:0016032~viral process | 15 | 4.273504 | 0.003887 | 348 | 2.420713 | 0.038774 |
| GOTERM_ | GO:0071526~semaphorin-plexin signaling pathway | 14 | 3.988604 | 3.95E-14 | 348 | 20.47092 | 6.90E-12 |
| GOTERM_ | GO:0019882~antigen processing and presentation | 14 | 3.988604 | 6.65E-11 | 348 | 12.28255 | 5.61E-09 |
| GOTERM_ | GO:0031295~T cell costimulation | 14 | 3.988604 | 6.59E-09 | 348 | 8.660772 | 4.13E-07 |
| GOTERM_ | GO:0043410~positive regulation of MAPK cascade | 14 | 3.988604 | 1.06E-08 | 348 | 8.340003 | 6.03E-07 |
| GOTERM_ | GO:0009615~response to virus | 14 | 3.988604 | 4.37E-07 | 348 | 6.141275 | 1.87E-05 |
| GOTERM_ | GO:0050900~leukocyte migration | 14 | 3.988604 | 1.45E-06 | 348 | 5.537215 | 5.37E-05 |
| GOTERM_ | GO:0043524~negative regulation of neuron apoptotic process | 14 | 3.988604 | 3.53E-06 | 348 | 5.117729 | 1.11E-04 |
| GOTERM_ | GO:0051092~positive regulation of NF-kappaB transcription factor activity | 14 | 3.988604 | 3.84E-06 | 348 | 5.07925 | 1.16E-04 |
| GOTERM_ | GO:0050852~T cell receptor signaling pathway | 14 | 3.988604 | 1.24E-05 | 348 | 4.564461 | 3.27E-04 |
| GOTERM_ | GO:0043123~positive regulation of I-kappaB kinase/NF-kappaB signaling | 14 | 3.988604 | 3.06E-05 | 348 | 4.195902 | 6.98E-04 |

| | | | | | | | |
|---|---|---|---|---|---|---|---|
| GOTERM_ | GO:0048843~negative regulation of axon extension involved in axon guidance | 13 | 3.703704 | 3.73E-14 | 348 | 24.12644 | 6.90E-12 |
| GOTERM_ | GO:0042517~positive regulation of tyrosine phosphorylation of Stat3 protein | 13 | 3.703704 | 8.39E-12 | 348 | 16.50756 | 8.54E-10 |
| GOTERM_ | GO:0010862~positive regulation of pathway-restricted SMAD protein phosphorylation | 13 | 3.703704 | 1.79E-10 | 348 | 13.06849 | 1.37E-08 |
| GOTERM_ | GO:0014068~positive regulation of phosphatidylinositol 3-kinase signaling | 13 | 3.703704 | 7.56E-09 | 348 | 9.650575 | 4.62E-07 |
| GOTERM_ | GO:0030593~neutrophil chemotaxis | 13 | 3.703704 | 9.07E-09 | 348 | 9.504354 | 5.28E-07 |
| GOTERM_ | GO:0070098~chemokine-mediated signaling pathway | 13 | 3.703704 | 2.15E-08 | 348 | 8.835033 | 1.12E-06 |
| GOTERM_ | GO:0048010~vascular endothelial growth factor receptor signaling pathway | 13 | 3.703704 | 2.53E-08 | 348 | 8.712324 | 1.29E-06 |
| GOTERM_ | GO:0050729~positive regulation of inflammatory response | 13 | 3.703704 | 2.98E-08 | 348 | 8.592977 | 1.45E-06 |
| GOTERM_ | GO:0071222~cellular response to lipopolysaccharide | 13 | 3.703704 | 3.82E-06 | 348 | 5.551216 | 1.16E-04 |
| GOTERM_ | GO:0042742~defense response to bacterium | 13 | 3.703704 | 4.84E-05 | 348 | 4.32612 | 0.001055 |
| GOTERM_ | GO:0043406~positive regulation of MAP kinase activity | 12 | 3.418803 | 2.89E-08 | 348 | 9.814144 | 1.44E-06 |
| GOTERM_ | GO:0060337~type I interferon signaling pathway | 12 | 3.418803 | 7.00E-08 | 348 | 9.047414 | 3.36E-06 |
| GOTERM_ | GO:0060326~cell chemotaxis | 12 | 3.418803 | 8.27E-08 | 348 | 8.908223 | 3.89E-06 |
| GOTERM_ | GO:0002576~platelet degranulation | 12 | 3.418803 | 9.16E-06 | 348 | 5.621694 | 2.54E-04 |
| GOTERM_ | GO:0071356~cellular response to tumor necrosis factor | 12 | 3.418803 | 1.72E-05 | 348 | 5.26395 | 4.34E-04 |
| GOTERM_ | GO:0030168~platelet activation | 12 | 3.418803 | 2.62E-05 | 348 | 5.035082 | 6.22E-04 |
| GOTERM_ | GO:0050776~regulation of immune response | 12 | 3.418803 | 0.001217 | 348 | 3.253003 | 0.016414 |
| GOTERM_ | GO:0006898~receptor-mediated endocytosis | 12 | 3.418803 | 0.001731 | 348 | 3.113089 | 0.020636 |
| GOTERM_ | GO:0042102~positive regulation of T cell proliferation | 11 | 3.133903 | 3.76E-07 | 348 | 8.84636 | 1.67E-05 |
| GOTERM_ | GO:0060395~SMAD protein signal transduction | 11 | 3.133903 | 5.17E-07 | 348 | 8.560994 | 2.14E-05 |
| GOTERM_ | GO:0046330~positive regulation of JNK cascade | 11 | 3.133903 | 8.14E-07 | 348 | 8.165871 | 3.21E-05 |
| GOTERM_ | GO:0071260~cellular response to mechanical stimulus | 11 | 3.133903 | 1.88E-06 | 348 | 7.475797 | 6.86E-05 |
| GOTERM_ | GO:0030509~BMP signaling pathway | 11 | 3.133903 | 3.55E-06 | 348 | 6.983969 | 1.11E-04 |
| GOTERM_ | GO:0051897~positive regulation of protein kinase B signaling | 11 | 3.133903 | 8.87E-06 | 348 | 6.318829 | 2.49E-04 |
| GOTERM_ | GO:0001501~skeletal system development | 11 | 3.133903 | 5.62E-04 | 348 | 3.874318 | 0.008635 |
| GOTERM_ | GO:0002250~adaptive immune response | 11 | 3.133903 | 0.001022 | 348 | 3.586362 | 0.014279 |
| GOTERM_ | GO:0046777~protein autophosphorylation | 11 | 3.133903 | 0.003115 | 348 | 3.08594 | 0.032538 |
| GOTERM_ | GO:0045740~positive regulation of DNA replication | 10 | 2.849003 | 1.51E-07 | 348 | 11.48878 | 6.98E-06 |
| GOTERM_ | GO:0007173~epidermal growth factor receptor signaling pathway | 10 | 2.849003 | 1.99E-06 | 348 | 8.616585 | 7.17E-05 |
| GOTERM_ | GO:0043401~steroid hormone mediated signaling pathway | 10 | 2.849003 | 2.33E-06 | 348 | 8.465416 | 8.15E-05 |
| GOTERM_ | GO:0045669~positive regulation of osteoblast differentiation | 10 | 2.849003 | 3.62E-06 | 348 | 8.042146 | 1.12E-04 |
| GOTERM_ | GO:0009887~organ morphogenesis | 10 | 2.849003 | 1.19E-04 | 348 | 5.244878 | 0.002293 |
| GOTERM_ | GO:0007179~transforming growth factor beta receptor signaling pathway | 10 | 2.849003 | 1.19E-04 | 348 | 5.244878 | 0.002293 |
| GOTERM_ | GO:0045471~response to ethanol | 10 | 2.849003 | 3.26E-04 | 348 | 4.595512 | 0.005348 |
| GOTERM_ | GO:0006367~transcription initiation from RNA polymerase II promoter | 10 | 2.849003 | 0.004383 | 348 | 3.174531 | 0.041517 |
| GOTERM_ | GO:0007568~aging | 10 | 2.849003 | 0.007421 | 348 | 2.924417 | 0.064087 |
| GOTERM_ | GO:0038095~Fc-epsilon receptor signaling pathway | 10 | 2.849003 | 0.01192 | 348 | 2.710836 | 0.091041 |
| GOTERM_ | GO:0001701~in utero embryonic development | 10 | 2.849003 | 0.016013 | 348 | 2.580368 | 0.114096 |
| GOTERM_ | GO:0031663~lipopolysaccharide-mediated signaling pathway | 9 | 2.564103 | 2.09E-07 | 348 | 13.57112 | 9.47E-06 |
| GOTERM_ | GO:0050918~positive chemotaxis | 9 | 2.564103 | 4.44E-07 | 348 | 12.40788 | 1.87E-05 |
| GOTERM_ | GO:0001657~ureteric bud development | 9 | 2.564103 | 8.73E-07 | 348 | 11.42831 | 3.39E-05 |
| GOTERM_ | GO:0045429~positive regulation of nitric oxide biosynthetic process | 9 | 2.564103 | 2.37E-06 | 348 | 10.09944 | 8.15E-05 |
| GOTERM_ | GO:0032755~positive regulation of interleukin-6 production | 9 | 2.564103 | 3.39E-06 | 348 | 9.650575 | 1.09E-04 |

| | | | | | | | |
|---|---|---|---|---|---|---|---|
| GOTERM_ | GO:0010595~positive regulation of endothelial cell migration | 9 | 2.564103 | 4.03E-06 | 348 | 9.44078 | 1.20E-04 |
| GOTERM_ | GO:0071346~cellular response to interferon-gamma | 9 | 2.564103 | 2.10E-05 | 348 | 7.618875 | 5.23E-04 |
| GOTERM_ | GO:0071347~cellular response to interleukin-1 | 9 | 2.564103 | 1.05E-04 | 348 | 6.116561 | 0.002122 |
| GOTERM_ | GO:0008543~fibroblast growth factor receptor signaling pathway | 9 | 2.564103 | 2.89E-04 | 348 | 5.296047 | 0.004775 |
| GOTERM_ | GO:0007565~female pregnancy | 9 | 2.564103 | 5.06E-04 | 348 | 4.879504 | 0.007873 |
| GOTERM_ | GO:0019886~antigen processing and presentation of exogenous peptide antigen via MHC cla | 9 | 2.564103 | 6.32E-04 | 348 | 4.72039 | 0.009652 |
| GOTERM_ | GO:0002223~stimulatory C-type lectin receptor signaling pathway | 9 | 2.564103 | 0.001502 | 348 | 4.135961 | 0.018733 |
| GOTERM_ | GO:0001934~positive regulation of protein phosphorylation | 9 | 2.564103 | 0.004894 | 348 | 3.419495 | 0.045136 |
| GOTERM_ | GO:0010629~negative regulation of gene expression | 9 | 2.564103 | 0.007653 | 348 | 3.169897 | 0.065421 |
| GOTERM_ | GO:0008360~regulation of cell shape | 9 | 2.564103 | 0.008672 | 348 | 3.10197 | 0.072094 |
| GOTERM_ | GO:0007596~blood coagulation | 9 | 2.564103 | 0.037901 | 348 | 2.360195 | 0.218466 |
| GOTERM_ | GO:0010575~positive regulation of vascular endothelial growth factor production | 8 | 2.279202 | 9.42E-07 | 348 | 14.29715 | 3.60E-05 |
| GOTERM_ | GO:0043552~positive regulation of phosphatidylinositol 3-kinase activity | 8 | 2.279202 | 2.60E-06 | 348 | 12.45235 | 8.70E-05 |
| GOTERM_ | GO:0038083~peptidyl-tyrosine autophosphorylation | 8 | 2.279202 | 1.57E-05 | 348 | 9.650575 | 4.00E-04 |
| GOTERM_ | GO:0007566~embryo implantation | 8 | 2.279202 | 2.19E-05 | 348 | 9.191024 | 5.41E-04 |
| GOTERM_ | GO:0031623~receptor internalization | 8 | 2.279202 | 2.57E-05 | 348 | 8.977279 | 6.17E-04 |
| GOTERM_ | GO:0023014~signal transduction by protein phosphorylation | 8 | 2.279202 | 3.01E-05 | 348 | 8.77325 | 6.93E-04 |
| GOTERM_ | GO:0032729~positive regulation of interferon-gamma production | 8 | 2.279202 | 4.05E-05 | 348 | 8.391804 | 9.00E-04 |
| GOTERM_ | GO:0036092~phosphatidylinositol-3-phosphate biosynthetic process | 8 | 2.279202 | 6.17E-05 | 348 | 7.87802 | 0.001311 |
| GOTERM_ | GO:0007189~adenylate cyclase-activating G-protein coupled receptor signaling pathway | 8 | 2.279202 | 7.05E-05 | 348 | 7.72046 | 0.001485 |
| GOTERM_ | GO:0048146~positive regulation of fibroblast proliferation | 8 | 2.279202 | 1.16E-04 | 348 | 7.148574 | 0.002276 |
| GOTERM_ | GO:0051592~response to calcium ion | 8 | 2.279202 | 1.84E-04 | 348 | 6.655569 | 0.003334 |
| GOTERM_ | GO:0007249~I-kappaB kinase/NF-kappaB signaling | 8 | 2.279202 | 2.28E-04 | 348 | 6.433716 | 0.004073 |
| GOTERM_ | GO:0006952~defense response | 8 | 2.279202 | 4.55E-04 | 348 | 5.761537 | 0.007213 |
| GOTERM_ | GO:0050728~negative regulation of inflammatory response | 8 | 2.279202 | 0.001227 | 348 | 4.886367 | 0.016414 |
| GOTERM_ | GO:0001503~ossification | 8 | 2.279202 | 0.001321 | 348 | 4.825287 | 0.016842 |
| GOTERM_ | GO:0071456~cellular response to hypoxia | 8 | 2.279202 | 0.003748 | 348 | 4.021073 | 0.037541 |
| GOTERM_ | GO:0031175~neuron projection development | 8 | 2.279202 | 0.004695 | 348 | 3.86023 | 0.043961 |
| GOTERM_ | GO:0090263~positive regulation of canonical Wnt signaling pathway | 8 | 2.279202 | 0.012331 | 348 | 3.216858 | 0.093887 |
| GOTERM_ | GO:0010951~negative regulation of endopeptidase activity | 8 | 2.279202 | 0.012864 | 348 | 3.190273 | 0.096443 |
| GOTERM_ | GO:0030308~negative regulation of cell growth | 8 | 2.279202 | 0.012864 | 348 | 3.190273 | 0.096443 |
| GOTERM_ | GO:0018105~peptidyl-serine phosphorylation | 8 | 2.279202 | 0.015162 | 348 | 3.088184 | 0.10996 |
| GOTERM_ | GO:0030036~actin cytoskeleton organization | 8 | 2.279202 | 0.018428 | 348 | 2.969408 | 0.125105 |
| GOTERM_ | GO:0090090~negative regulation of canonical Wnt signaling pathway | 8 | 2.279202 | 0.052812 | 348 | 2.368239 | 0.273456 |
| GOTERM_ | GO:0002504~antigen processing and presentation of peptide or polysaccharide antigen via M | 7 | 1.994302 | 7.62E-07 | 348 | 19.86883 | 3.05E-05 |
| GOTERM_ | GO:0042346~positive regulation of NF-kappaB import into nucleus | 7 | 1.994302 | 3.11E-06 | 348 | 16.08429 | 1.01E-04 |
| GOTERM_ | GO:0090023~positive regulation of neutrophil chemotaxis | 7 | 1.994302 | 4.21E-06 | 348 | 15.35319 | 1.24E-04 |
| GOTERM_ | GO:0002040~sprouting angiogenesis | 7 | 1.994302 | 9.47E-06 | 348 | 13.5108 | 2.57E-04 |
| GOTERM_ | GO:0045840~positive regulation of mitotic nuclear division | 7 | 1.994302 | 1.21E-05 | 348 | 12.99116 | 3.22E-04 |
| GOTERM_ | GO:0032757~positive regulation of interleukin-8 production | 7 | 1.994302 | 1.21E-05 | 348 | 12.99116 | 3.22E-04 |
| GOTERM_ | GO:0032728~positive regulation of interferon-beta production | 7 | 1.994302 | 1.53E-05 | 348 | 12.51 | 3.93E-04 |
| GOTERM_ | GO:0045909~positive regulation of vasodilation | 7 | 1.994302 | 2.37E-05 | 348 | 11.64725 | 5.79E-04 |
| GOTERM_ | GO:0048286~lung alveolus development | 7 | 1.994302 | 6.15E-05 | 348 | 9.934415 | 0.001311 |

| | | | | | | | |
|---|---|---|---|---|---|---|---|
| GOTERM_ | GO:2001240~negative regulation of extrinsic apoptotic signaling pathway in absence of ligar | 7 | 1.994302 | 1.01E-04 | 348 | 9.128922 | 0.002055 |
| GOTERM_ | GO:0032570~response to progesterone | 7 | 1.994302 | 1.37E-04 | 348 | 8.660772 | 0.002602 |
| GOTERM_ | GO:0045071~negative regulation of viral genome replication | 7 | 1.994302 | 1.58E-04 | 348 | 8.444253 | 0.002951 |
| GOTERM_ | GO:0043408~regulation of MAPK cascade | 7 | 1.994302 | 1.82E-04 | 348 | 8.238295 | 0.003321 |
| GOTERM_ | GO:0002548~monocyte chemotaxis | 7 | 1.994302 | 2.09E-04 | 348 | 8.042146 | 0.003752 |
| GOTERM_ | GO:0030155~regulation of cell adhesion | 7 | 1.994302 | 2.38E-04 | 348 | 7.855119 | 0.004158 |
| GOTERM_ | GO:0007187~G-protein coupled receptor signaling pathway, coupled to cyclic nucleotide sec | 7 | 1.994302 | 3.48E-04 | 348 | 7.342829 | 0.005667 |
| GOTERM_ | GO:0032760~positive regulation of tumor necrosis factor production | 7 | 1.994302 | 3.92E-04 | 348 | 7.186598 | 0.006342 |
| GOTERM_ | GO:0019233~sensory perception of pain | 7 | 1.994302 | 6.81E-04 | 348 | 6.495579 | 0.009972 |
| GOTERM_ | GO:0050829~defense response to Gram-negative bacterium | 7 | 1.994302 | 9.21E-04 | 348 | 6.141275 | 0.013167 |
| GOTERM_ | GO:0042475~odontogenesis of dentin-containing tooth | 7 | 1.994302 | 9.21E-04 | 348 | 6.141275 | 0.013167 |
| GOTERM_ | GO:0016049~cell growth | 7 | 1.994302 | 0.001014 | 348 | 6.031609 | 0.014245 |
| GOTERM_ | GO:0050679~positive regulation of epithelial cell proliferation | 7 | 1.994302 | 0.001459 | 348 | 5.629502 | 0.018292 |
| GOTERM_ | GO:0009611~response to wounding | 7 | 1.994302 | 0.001881 | 348 | 5.36143 | 0.021997 |
| GOTERM_ | GO:0051384~response to glucocorticoid | 7 | 1.994302 | 0.002209 | 348 | 5.196463 | 0.02477 |
| GOTERM_ | GO:0008217~regulation of blood pressure | 7 | 1.994302 | 0.002209 | 348 | 5.196463 | 0.02477 |
| GOTERM_ | GO:0009749~response to glucose | 7 | 1.994302 | 0.002781 | 348 | 4.967208 | 0.029806 |
| GOTERM_ | GO:0035690~cellular response to drug | 7 | 1.994302 | 0.002994 | 348 | 4.895219 | 0.031536 |
| GOTERM_ | GO:0030855~epithelial cell differentiation | 7 | 1.994302 | 0.003219 | 348 | 4.825287 | 0.033331 |
| GOTERM_ | GO:0097190~apoptotic signaling pathway | 7 | 1.994302 | 0.003456 | 348 | 4.757326 | 0.035045 |
| GOTERM_ | GO:0032355~response to estradiol | 7 | 1.994302 | 0.01143 | 348 | 3.71176 | 0.088403 |
| GOTERM_ | GO:0008584~male gonad development | 7 | 1.994302 | 0.013268 | 348 | 3.593299 | 0.099167 |
| GOTERM_ | GO:0007229~integrin-mediated signaling pathway | 7 | 1.994302 | 0.016775 | 348 | 3.411819 | 0.117884 |
| GOTERM_ | GO:0042593~glucose homeostasis | 7 | 1.994302 | 0.018342 | 348 | 3.344259 | 0.124866 |
| GOTERM_ | GO:0001764~neuron migration | 7 | 1.994302 | 0.021768 | 348 | 3.216858 | 0.141494 |
| GOTERM_ | GO:0042088~T-helper 1 type immune response | 6 | 1.709402 | 2.57E-06 | 348 | 24.12644 | 8.70E-05 |
| GOTERM_ | GO:0050930~induction of positive chemotaxis | 6 | 1.709402 | 9.27E-06 | 348 | 19.30115 | 2.55E-04 |
| GOTERM_ | GO:0002690~positive regulation of leukocyte chemotaxis | 6 | 1.709402 | 2.51E-05 | 348 | 16.08429 | 6.08E-04 |
| GOTERM_ | GO:0043536~positive regulation of blood vessel endothelial cell migration | 6 | 1.709402 | 3.35E-05 | 348 | 15.23775 | 7.59E-04 |
| GOTERM_ | GO:0014911~positive regulation of smooth muscle cell migration | 6 | 1.709402 | 4.40E-05 | 348 | 14.47586 | 9.68E-04 |
| GOTERM_ | GO:0042327~positive regulation of phosphorylation | 6 | 1.709402 | 1.38E-04 | 348 | 11.58069 | 0.002602 |
| GOTERM_ | GO:0007159~leukocyte cell-cell adhesion | 6 | 1.709402 | 1.38E-04 | 348 | 11.58069 | 0.002602 |
| GOTERM_ | GO:0032689~negative regulation of interferon-gamma production | 6 | 1.709402 | 2.43E-04 | 348 | 10.3399 | 0.004158 |
| GOTERM_ | GO:0035666~TRIF-dependent toll-like receptor signaling pathway | 6 | 1.709402 | 2.43E-04 | 348 | 10.3399 | 0.004158 |
| GOTERM_ | GO:0040007~growth | 6 | 1.709402 | 2.43E-04 | 348 | 10.3399 | 0.004158 |
| GOTERM_ | GO:2000352~negative regulation of endothelial cell apoptotic process | 6 | 1.709402 | 2.43E-04 | 348 | 10.3399 | 0.004158 |
| GOTERM_ | GO:0001569~patterning of blood vessels | 6 | 1.709402 | 2.43E-04 | 348 | 10.3399 | 0.004158 |
| GOTERM_ | GO:0001974~blood vessel remodeling | 6 | 1.709402 | 4.66E-04 | 348 | 9.047414 | 0.007298 |
| GOTERM_ | GO:0007259~JAK-STAT cascade | 6 | 1.709402 | 4.66E-04 | 348 | 9.047414 | 0.007298 |
| GOTERM_ | GO:0042130~negative regulation of T cell proliferation | 6 | 1.709402 | 9.27E-04 | 348 | 7.82479 | 0.013168 |
| GOTERM_ | GO:2001237~negative regulation of extrinsic apoptotic signaling pathway | 6 | 1.709402 | 0.001049 | 348 | 7.618875 | 0.014407 |
| GOTERM_ | GO:0030522~intracellular receptor signaling pathway | 6 | 1.709402 | 0.001049 | 348 | 7.618875 | 0.014407 |
| GOTERM_ | GO:0038128~ERBB2 signaling pathway | 6 | 1.709402 | 0.001049 | 348 | 7.618875 | 0.014407 |

| | | | | | | | |
|---|---|---|---|---|---|---|---|
| GOTERM_ | GO:0048468~cell development | 6 | 1.709402 | 0.00133 | 348 | 7.237931 | 0.016842 |
| GOTERM_ | GO:0030819~positive regulation of cAMP biosynthetic process | 6 | 1.709402 | 0.00133 | 348 | 7.237931 | 0.016842 |
| GOTERM_ | GO:0097191~extrinsic apoptotic signaling pathway | 6 | 1.709402 | 0.001662 | 348 | 6.893268 | 0.020073 |
| GOTERM_ | GO:0045785~positive regulation of cell adhesion | 6 | 1.709402 | 0.00185 | 348 | 6.732959 | 0.021947 |
| GOTERM_ | GO:0071363~cellular response to growth factor stimulus | 6 | 1.709402 | 0.00227 | 348 | 6.433716 | 0.025336 |
| GOTERM_ | GO:0000186~activation of MAPKK activity | 6 | 1.709402 | 0.002505 | 348 | 6.293853 | 0.027086 |
| GOTERM_ | GO:0071560~cellular response to transforming growth factor beta stimulus | 6 | 1.709402 | 0.003313 | 348 | 5.908515 | 0.033741 |
| GOTERM_ | GO:0006959~humoral immune response | 6 | 1.709402 | 0.006364 | 348 | 5.07925 | 0.055951 |
| GOTERM_ | GO:0009612~response to mechanical stimulus | 6 | 1.709402 | 0.00736 | 348 | 4.907072 | 0.063787 |
| GOTERM_ | GO:0016525~negative regulation of angiogenesis | 6 | 1.709402 | 0.00905 | 348 | 4.669633 | 0.074469 |
| GOTERM_ | GO:0038061~NIK/NF-kappaB signaling | 6 | 1.709402 | 0.011694 | 348 | 4.386625 | 0.089595 |
| GOTERM_ | GO:0030183~B cell differentiation | 6 | 1.709402 | 0.011694 | 348 | 4.386625 | 0.089595 |
| GOTERM_ | GO:0007200~phospholipase C-activating G-protein coupled receptor signaling pathway | 6 | 1.709402 | 0.011694 | 348 | 4.386625 | 0.089595 |
| GOTERM_ | GO:0050796~regulation of insulin secretion | 6 | 1.709402 | 0.012429 | 348 | 4.321153 | 0.094048 |
| GOTERM_ | GO:0032868~response to insulin | 6 | 1.709402 | 0.012429 | 348 | 4.321153 | 0.094048 |
| GOTERM_ | GO:0043154~negative regulation of cysteine-type endopeptidase activity involved in apoptot | 6 | 1.709402 | 0.013992 | 348 | 4.195902 | 0.101776 |
| GOTERM_ | GO:0007015~actin filament organization | 6 | 1.709402 | 0.016576 | 348 | 4.021073 | 0.117764 |
| GOTERM_ | GO:0060021~palate development | 6 | 1.709402 | 0.020487 | 348 | 3.809437 | 0.135293 |
| GOTERM_ | GO:0022617~extracellular matrix disassembly | 6 | 1.709402 | 0.020487 | 348 | 3.809437 | 0.135293 |
| GOTERM_ | GO:0030324~lung development | 6 | 1.709402 | 0.020487 | 348 | 3.809437 | 0.135293 |
| GOTERM_ | GO:0046718~viral entry into host cell | 6 | 1.709402 | 0.024958 | 348 | 3.618966 | 0.154422 |
| GOTERM_ | GO:0006919~activation of cysteine-type endopeptidase activity involved in apoptotic proces | 6 | 1.709402 | 0.028692 | 348 | 3.48816 | 0.172291 |
| GOTERM_ | GO:0007160~cell-matrix adhesion | 6 | 1.709402 | 0.038722 | 348 | 3.216858 | 0.222673 |
| GOTERM_ | GO:0006874~cellular calcium ion homeostasis | 6 | 1.709402 | 0.043599 | 348 | 3.113089 | 0.233164 |
| GOTERM_ | GO:0030336~negative regulation of cell migration | 6 | 1.709402 | 0.047046 | 348 | 3.04755 | 0.249181 |
| GOTERM_ | GO:0016337~single organismal cell-cell adhesion | 6 | 1.709402 | 0.058333 | 348 | 2.866507 | 0.293305 |
| GOTERM_ | GO:0043488~regulation of mRNA stability | 6 | 1.709402 | 0.062411 | 348 | 2.810847 | 0.300853 |
| GOTERM_ | GO:0007219~Notch signaling pathway | 6 | 1.709402 | 0.090143 | 348 | 2.517541 | 0.387186 |
| GOTERM_ | GO:0046888~negative regulation of hormone secretion | 5 | 1.424501 | 3.41E-05 | 348 | 24.12644 | 7.65E-04 |
| GOTERM_ | GO:0048846~axon extension involved in axon guidance | 5 | 1.424501 | 7.78E-05 | 348 | 20.10536 | 0.001598 |
| GOTERM_ | GO:0030195~negative regulation of blood coagulation | 5 | 1.424501 | 7.78E-05 | 348 | 20.10536 | 0.001598 |
| GOTERM_ | GO:0032727~positive regulation of interferon-alpha production | 5 | 1.424501 | 7.78E-05 | 348 | 20.10536 | 0.001598 |
| GOTERM_ | GO:0060389~pathway-restricted SMAD protein phosphorylation | 5 | 1.424501 | 1.11E-04 | 348 | 18.5588 | 0.002197 |
| GOTERM_ | GO:0048246~macrophage chemotaxis | 5 | 1.424501 | 1.11E-04 | 348 | 18.5588 | 0.002197 |
| GOTERM_ | GO:0032924~activin receptor signaling pathway | 5 | 1.424501 | 2.68E-04 | 348 | 15.07902 | 0.004516 |
| GOTERM_ | GO:0030949~positive regulation of vascular endothelial growth factor receptor signaling pat | 5 | 1.424501 | 2.68E-04 | 348 | 15.07902 | 0.004516 |
| GOTERM_ | GO:0010596~negative regulation of endothelial cell migration | 5 | 1.424501 | 4.36E-04 | 348 | 13.40358 | 0.00701 |
| GOTERM_ | GO:0031290~retinal ganglion cell axon guidance | 5 | 1.424501 | 5.43E-04 | 348 | 12.69812 | 0.008404 |
| GOTERM_ | GO:0046697~decidualization | 5 | 1.424501 | 6.68E-04 | 348 | 12.06322 | 0.009863 |
| GOTERM_ | GO:1900182~positive regulation of protein localization to nucleus | 5 | 1.424501 | 8.12E-04 | 348 | 11.48878 | 0.011743 |
| GOTERM_ | GO:0031954~positive regulation of protein autophosphorylation | 5 | 1.424501 | 8.12E-04 | 348 | 11.48878 | 0.011743 |
| GOTERM_ | GO:0032735~positive regulation of interleukin-12 production | 5 | 1.424501 | 0.001608 | 348 | 9.650575 | 0.01975 |
| GOTERM_ | GO:0050715~positive regulation of cytokine secretion | 5 | 1.424501 | 0.001608 | 348 | 9.650575 | 0.01975 |

| | | | | | | |
|---|---|---|---|---|---|---|
| GOTERM_ | GO:0032148~activation of protein kinase B activity | 5 | 1.424501 | 0.00187 | 348 | 9.279399 | 0.021971 |
| GOTERM_ | GO:0002053~positive regulation of mesenchymal cell proliferation | 5 | 1.424501 | 0.00187 | 348 | 9.279399 | 0.021971 |
| GOTERM_ | GO:0002224~toll-like receptor signaling pathway | 5 | 1.424501 | 0.00216 | 348 | 8.935717 | 0.024437 |
| GOTERM_ | GO:0001937~negative regulation of endothelial cell proliferation | 5 | 1.424501 | 0.00283 | 348 | 8.319461 | 0.029937 |
| GOTERM_ | GO:0043542~endothelial cell migration | 5 | 1.424501 | 0.00283 | 348 | 8.319461 | 0.029937 |
| GOTERM_ | GO:0048008~platelet-derived growth factor receptor signaling pathway | 5 | 1.424501 | 0.00283 | 348 | 8.319461 | 0.029937 |
| GOTERM_ | GO:0002474~antigen processing and presentation of peptide antigen via MHC class I | 5 | 1.424501 | 0.003212 | 348 | 8.042146 | 0.033331 |
| GOTERM_ | GO:0043200~response to amino acid | 5 | 1.424501 | 0.003629 | 348 | 7.782722 | 0.0365 |
| GOTERM_ | GO:0045765~regulation of angiogenesis | 5 | 1.424501 | 0.003629 | 348 | 7.782722 | 0.0365 |
| GOTERM_ | GO:0045907~positive regulation of vasoconstriction | 5 | 1.424501 | 0.004081 | 348 | 7.539511 | 0.038856 |
| GOTERM_ | GO:0010634~positive regulation of epithelial cell migration | 5 | 1.424501 | 0.004569 | 348 | 7.311041 | 0.042952 |
| GOTERM_ | GO:1902042~negative regulation of extrinsic apoptotic signaling pathway via death domain r | 5 | 1.424501 | 0.004569 | 348 | 7.311041 | 0.042952 |
| GOTERM_ | GO:0030282~bone mineralization | 5 | 1.424501 | 0.005096 | 348 | 7.096011 | 0.04647 |
| GOTERM_ | GO:0097192~extrinsic apoptotic signaling pathway in absence of ligand | 5 | 1.424501 | 0.005096 | 348 | 7.096011 | 0.04647 |
| GOTERM_ | GO:0001837~epithelial to mesenchymal transition | 5 | 1.424501 | 0.005096 | 348 | 7.096011 | 0.04647 |
| GOTERM_ | GO:0030501~positive regulation of bone mineralization | 5 | 1.424501 | 0.005661 | 348 | 6.893268 | 0.050752 |
| GOTERM_ | GO:0050714~positive regulation of protein secretion | 5 | 1.424501 | 0.006267 | 348 | 6.701788 | 0.055293 |
| GOTERM_ | GO:0045597~positive regulation of cell differentiation | 5 | 1.424501 | 0.006914 | 348 | 6.520659 | 0.060135 |
| GOTERM_ | GO:0001942~hair follicle development | 5 | 1.424501 | 0.007604 | 348 | 6.349062 | 0.065421 |
| GOTERM_ | GO:0030890~positive regulation of B cell proliferation | 5 | 1.424501 | 0.008338 | 348 | 6.186266 | 0.069635 |
| GOTERM_ | GO:0006953~acute-phase response | 5 | 1.424501 | 0.008338 | 348 | 6.186266 | 0.069635 |
| GOTERM_ | GO:0030326~embryonic limb morphogenesis | 5 | 1.424501 | 0.009117 | 348 | 6.031609 | 0.074768 |
| GOTERM_ | GO:0031397~negative regulation of protein ubiquitination | 5 | 1.424501 | 0.009941 | 348 | 5.884497 | 0.081257 |
| GOTERM_ | GO:0001658~branching involved in ureteric bud morphogenesis | 5 | 1.424501 | 0.010812 | 348 | 5.74439 | 0.086285 |
| GOTERM_ | GO:0043434~response to peptide hormone | 5 | 1.424501 | 0.0127 | 348 | 5.483281 | 0.095796 |
| GOTERM_ | GO:0032870~cellular response to hormone stimulus | 5 | 1.424501 | 0.013717 | 348 | 5.36143 | 0.101594 |
| GOTERM_ | GO:0032147~activation of protein kinase activity | 5 | 1.424501 | 0.013717 | 348 | 5.36143 | 0.101594 |
| GOTERM_ | GO:0007193~adenylate cyclase-inhibiting G-protein coupled receptor signaling pathway | 5 | 1.424501 | 0.015904 | 348 | 5.133284 | 0.114096 |
| GOTERM_ | GO:0045860~positive regulation of protein kinase activity | 5 | 1.424501 | 0.015904 | 348 | 5.133284 | 0.114096 |
| GOTERM_ | GO:0031100~organ regeneration | 5 | 1.424501 | 0.015904 | 348 | 5.133284 | 0.114096 |
| GOTERM_ | GO:0006909~phagocytosis | 5 | 1.424501 | 0.017075 | 348 | 5.026341 | 0.117884 |
| GOTERM_ | GO:0009408~response to heat | 5 | 1.424501 | 0.017075 | 348 | 5.026341 | 0.117884 |
| GOTERM_ | GO:0045727~positive regulation of translation | 5 | 1.424501 | 0.023727 | 348 | 4.552158 | 0.151408 |
| GOTERM_ | GO:0060548~negative regulation of cell death | 5 | 1.424501 | 0.02677 | 348 | 4.386625 | 0.165219 |
| GOTERM_ | GO:0071407~cellular response to organic cyclic compound | 5 | 1.424501 | 0.033528 | 348 | 4.089227 | 0.196507 |
| GOTERM_ | GO:0030097~hemopoiesis | 5 | 1.424501 | 0.033528 | 348 | 4.089227 | 0.196507 |
| GOTERM_ | GO:0045732~positive regulation of protein catabolic process | 5 | 1.424501 | 0.035359 | 348 | 4.021073 | 0.206249 |
| GOTERM_ | GO:0001523~retinoid metabolic process | 5 | 1.424501 | 0.037247 | 348 | 3.955154 | 0.216325 |
| GOTERM_ | GO:0007613~memory | 5 | 1.424501 | 0.039192 | 348 | 3.891361 | 0.224846 |
| GOTERM_ | GO:0002479~antigen processing and presentation of exogenous peptide antigen via MHC cla | 5 | 1.424501 | 0.041193 | 348 | 3.829593 | 0.225732 |
| GOTERM_ | GO:0050727~regulation of inflammatory response | 5 | 1.424501 | 0.041193 | 348 | 3.829593 | 0.225732 |
| GOTERM_ | GO:0007265~Ras protein signal transduction | 5 | 1.424501 | 0.056796 | 348 | 3.446634 | 0.292847 |
| GOTERM_ | GO:0033138~positive regulation of peptidyl-serine phosphorylation | 5 | 1.424501 | 0.056796 | 348 | 3.446634 | 0.292847 |

| | | | | | | | |
|---|---|---|---|---|---|---|---|
| GOTERM_ | GO:0045444~fat cell differentiation | 5 | 1.424501 | 0.064327 | 348 | 3.304991 | 0.307059 |
| GOTERM_ | GO:0030334~regulation of cell migration | 5 | 1.424501 | 0.066947 | 348 | 3.260329 | 0.318326 |
| GOTERM_ | GO:0006816~calcium ion transport | 5 | 1.424501 | 0.072353 | 348 | 3.174531 | 0.340712 |
| GOTERM_ | GO:0030307~positive regulation of cell growth | 5 | 1.424501 | 0.096089 | 348 | 2.872195 | 0.403503 |
| GOTERM_ | GO:0009636~response to toxic substance | 5 | 1.424501 | 0.099285 | 348 | 2.838404 | 0.403503 |
| GOTERM_ | GO:0050830~defense response to Gram-positive bacterium | 5 | 1.424501 | 0.099285 | 348 | 2.838404 | 0.403503 |
| GOTERM_ | GO:0038084~vascular endothelial growth factor signaling pathway | 4 | 1.139601 | 1.67E-04 | 348 | 32.16858 | 0.003093 |
| GOTERM_ | GO:0002455~humoral immune response mediated by circulating immunoglobulin | 4 | 1.139601 | 2.88E-04 | 348 | 27.57307 | 0.004775 |
| GOTERM_ | GO:0045580~regulation of T cell differentiation | 4 | 1.139601 | 4.54E-04 | 348 | 24.12644 | 0.007213 |
| GOTERM_ | GO:0071223~cellular response to lipoteichoic acid | 4 | 1.139601 | 6.70E-04 | 348 | 21.44572 | 0.009863 |
| GOTERM_ | GO:0002237~response to molecule of bacterial origin | 4 | 1.139601 | 6.70E-04 | 348 | 21.44572 | 0.009863 |
| GOTERM_ | GO:0021675~nerve development | 4 | 1.139601 | 6.70E-04 | 348 | 21.44572 | 0.009863 |
| GOTERM_ | GO:0021612~facial nerve structural organization | 4 | 1.139601 | 6.70E-04 | 348 | 21.44572 | 0.009863 |
| GOTERM_ | GO:0021785~branchiomotor neuron axon guidance | 4 | 1.139601 | 6.70E-04 | 348 | 21.44572 | 0.009863 |
| GOTERM_ | GO:0042989~sequestering of actin monomers | 4 | 1.139601 | 9.42E-04 | 348 | 19.30115 | 0.013314 |
| GOTERM_ | GO:0010759~positive regulation of macrophage chemotaxis | 4 | 1.139601 | 0.001276 | 348 | 17.5465 | 0.016414 |
| GOTERM_ | GO:0032703~negative regulation of interleukin-2 production | 4 | 1.139601 | 0.001276 | 348 | 17.5465 | 0.016414 |
| GOTERM_ | GO:0048711~positive regulation of astrocyte differentiation | 4 | 1.139601 | 0.001276 | 348 | 17.5465 | 0.016414 |
| GOTERM_ | GO:0042511~positive regulation of tyrosine phosphorylation of Stat1 protein | 4 | 1.139601 | 0.001276 | 348 | 17.5465 | 0.016414 |
| GOTERM_ | GO:0031669~cellular response to nutrient levels | 4 | 1.139601 | 0.001675 | 348 | 16.08429 | 0.020073 |
| GOTERM_ | GO:0007252~I-kappaB phosphorylation | 4 | 1.139601 | 0.001675 | 348 | 16.08429 | 0.020073 |
| GOTERM_ | GO:0035385~Roundabout signaling pathway | 4 | 1.139601 | 0.001675 | 348 | 16.08429 | 0.020073 |
| GOTERM_ | GO:1902287~semaphorin-plexin signaling pathway involved in axon guidance | 4 | 1.139601 | 0.001675 | 348 | 16.08429 | 0.020073 |
| GOTERM_ | GO:0051926~negative regulation of calcium ion transport | 4 | 1.139601 | 0.002145 | 348 | 14.84704 | 0.024382 |
| GOTERM_ | GO:0033630~positive regulation of cell adhesion mediated by integrin | 4 | 1.139601 | 0.002145 | 348 | 14.84704 | 0.024382 |
| GOTERM_ | GO:0051770~positive regulation of nitric-oxide synthase biosynthetic process | 4 | 1.139601 | 0.002145 | 348 | 14.84704 | 0.024382 |
| GOTERM_ | GO:0050730~regulation of peptidyl-tyrosine phosphorylation | 4 | 1.139601 | 0.002145 | 348 | 14.84704 | 0.024382 |
| GOTERM_ | GO:0016045~detection of bacterium | 4 | 1.139601 | 0.002145 | 348 | 14.84704 | 0.024382 |
| GOTERM_ | GO:0001878~response to yeast | 4 | 1.139601 | 0.002145 | 348 | 14.84704 | 0.024382 |
| GOTERM_ | GO:0060065~uterus development | 4 | 1.139601 | 0.002688 | 348 | 13.78654 | 0.028944 |
| GOTERM_ | GO:0002042~cell migration involved in sprouting angiogenesis | 4 | 1.139601 | 0.003309 | 348 | 12.86743 | 0.033741 |
| GOTERM_ | GO:0042026~protein refolding | 4 | 1.139601 | 0.003309 | 348 | 12.86743 | 0.033741 |
| GOTERM_ | GO:0002437~inflammatory response to antigenic stimulus | 4 | 1.139601 | 0.003309 | 348 | 12.86743 | 0.033741 |
| GOTERM_ | GO:0003203~endocardial cushion morphogenesis | 4 | 1.139601 | 0.004011 | 348 | 12.06322 | 0.038856 |
| GOTERM_ | GO:0061036~positive regulation of cartilage development | 4 | 1.139601 | 0.004011 | 348 | 12.06322 | 0.038856 |
| GOTERM_ | GO:0032722~positive regulation of chemokine production | 4 | 1.139601 | 0.004797 | 348 | 11.35362 | 0.044408 |
| GOTERM_ | GO:0006171~cAMP biosynthetic process | 4 | 1.139601 | 0.004797 | 348 | 11.35362 | 0.044408 |
| GOTERM_ | GO:0048514~blood vessel morphogenesis | 4 | 1.139601 | 0.004797 | 348 | 11.35362 | 0.044408 |
| GOTERM_ | GO:0033280~response to vitamin D | 4 | 1.139601 | 0.005669 | 348 | 10.72286 | 0.050752 |
| GOTERM_ | GO:0034142~toll-like receptor 4 signaling pathway | 4 | 1.139601 | 0.005669 | 348 | 10.72286 | 0.050752 |
| GOTERM_ | GO:0042267~natural killer cell mediated cytotoxicity | 4 | 1.139601 | 0.005669 | 348 | 10.72286 | 0.050752 |
| GOTERM_ | GO:0036120~cellular response to platelet-derived growth factor stimulus | 4 | 1.139601 | 0.005669 | 348 | 10.72286 | 0.050752 |
| GOTERM_ | GO:0046579~positive regulation of Ras protein signal transduction | 4 | 1.139601 | 0.00663 | 348 | 10.1585 | 0.057873 |

| | | | | | | | |
|---|---|---|---|---|---|---|---|
| GOTERM_ | GO:0051968~positive regulation of synaptic transmission, glutamatergic | 4 | 1.139601 | 0.00663 | 348 | 10.1585 | 0.057873 |
| GOTERM_ | GO:0071385~cellular response to glucocorticoid stimulus | 4 | 1.139601 | 0.007682 | 348 | 9.650575 | 0.065421 |
| GOTERM_ | GO:0030101~natural killer cell activation | 4 | 1.139601 | 0.007682 | 348 | 9.650575 | 0.065421 |
| GOTERM_ | GO:0050772~positive regulation of axonogenesis | 4 | 1.139601 | 0.008827 | 348 | 9.191024 | 0.072886 |
| GOTERM_ | GO:0045987~positive regulation of smooth muscle contraction | 4 | 1.139601 | 0.008827 | 348 | 9.191024 | 0.072886 |
| GOTERM_ | GO:0060045~positive regulation of cardiac muscle cell proliferation | 4 | 1.139601 | 0.010067 | 348 | 8.77325 | 0.08147 |
| GOTERM_ | GO:0001954~positive regulation of cell-matrix adhesion | 4 | 1.139601 | 0.010067 | 348 | 8.77325 | 0.08147 |
| GOTERM_ | GO:0045453~bone resorption | 4 | 1.139601 | 0.010067 | 348 | 8.77325 | 0.08147 |
| GOTERM_ | GO:0048754~branching morphogenesis of an epithelial tube | 4 | 1.139601 | 0.011403 | 348 | 8.391804 | 0.088403 |
| GOTERM_ | GO:0003148~outflow tract septum morphogenesis | 4 | 1.139601 | 0.011403 | 348 | 8.391804 | 0.088403 |
| GOTERM_ | GO:0035924~cellular response to vascular endothelial growth factor stimulus | 4 | 1.139601 | 0.011403 | 348 | 8.391804 | 0.088403 |
| GOTERM_ | GO:0032733~positive regulation of interleukin-10 production | 4 | 1.139601 | 0.011403 | 348 | 8.391804 | 0.088403 |
| GOTERM_ | GO:2001238~positive regulation of extrinsic apoptotic signaling pathway | 4 | 1.139601 | 0.015997 | 348 | 7.423519 | 0.114096 |
| GOTERM_ | GO:0070328~triglyceride homeostasis | 4 | 1.139601 | 0.015997 | 348 | 7.423519 | 0.114096 |
| GOTERM_ | GO:0001656~metanephros development | 4 | 1.139601 | 0.017726 | 348 | 7.148574 | 0.121013 |
| GOTERM_ | GO:0042476~odontogenesis | 4 | 1.139601 | 0.017726 | 348 | 7.148574 | 0.121013 |
| GOTERM_ | GO:2000145~regulation of cell motility | 4 | 1.139601 | 0.019556 | 348 | 6.893268 | 0.132394 |
| GOTERM_ | GO:0030203~glycosaminoglycan metabolic process | 4 | 1.139601 | 0.021486 | 348 | 6.655569 | 0.14003 |
| GOTERM_ | GO:0030449~regulation of complement activation | 4 | 1.139601 | 0.023516 | 348 | 6.433716 | 0.150454 |
| GOTERM_ | GO:0032480~negative regulation of type I interferon production | 4 | 1.139601 | 0.023516 | 348 | 6.433716 | 0.150454 |
| GOTERM_ | GO:0001654~eye development | 4 | 1.139601 | 0.023516 | 348 | 6.433716 | 0.150454 |
| GOTERM_ | GO:0045776~negative regulation of blood pressure | 4 | 1.139601 | 0.023516 | 348 | 6.433716 | 0.150454 |
| GOTERM_ | GO:0071773~cellular response to BMP stimulus | 4 | 1.139601 | 0.023516 | 348 | 6.433716 | 0.150454 |
| GOTERM_ | GO:0010803~regulation of tumor necrosis factor-mediated signaling pathway | 4 | 1.139601 | 0.023516 | 348 | 6.433716 | 0.150454 |
| GOTERM_ | GO:0042177~negative regulation of protein catabolic process | 4 | 1.139601 | 0.027878 | 348 | 6.031609 | 0.171189 |
| GOTERM_ | GO:0007498~mesoderm development | 4 | 1.139601 | 0.027878 | 348 | 6.031609 | 0.171189 |
| GOTERM_ | GO:0002755~MyD88-dependent toll-like receptor signaling pathway | 4 | 1.139601 | 0.030209 | 348 | 5.848833 | 0.180073 |
| GOTERM_ | GO:0010718~positive regulation of epithelial to mesenchymal transition | 4 | 1.139601 | 0.030209 | 348 | 5.848833 | 0.180073 |
| GOTERM_ | GO:0070555~response to interleukin-1 | 4 | 1.139601 | 0.030209 | 348 | 5.848833 | 0.180073 |
| GOTERM_ | GO:0043330~response to exogenous dsRNA | 4 | 1.139601 | 0.032639 | 348 | 5.676809 | 0.193694 |
| GOTERM_ | GO:0048565~digestive tract development | 4 | 1.139601 | 0.035168 | 348 | 5.514614 | 0.205624 |
| GOTERM_ | GO:0034605~cellular response to heat | 4 | 1.139601 | 0.04052 | 348 | 5.216527 | 0.225153 |
| GOTERM_ | GO:0019933~cAMP-mediated signaling | 4 | 1.139601 | 0.043342 | 348 | 5.07925 | 0.232296 |
| GOTERM_ | GO:0008625~extrinsic apoptotic signaling pathway via death domain receptors | 4 | 1.139601 | 0.043342 | 348 | 5.07925 | 0.232296 |
| GOTERM_ | GO:0009790~embryo development | 4 | 1.139601 | 0.043342 | 348 | 5.07925 | 0.232296 |
| GOTERM_ | GO:0032720~negative regulation of tumor necrosis factor production | 4 | 1.139601 | 0.043342 | 348 | 5.07925 | 0.232296 |
| GOTERM_ | GO:0002062~chondrocyte differentiation | 4 | 1.139601 | 0.046259 | 348 | 4.949013 | 0.24631 |
| GOTERM_ | GO:0051262~protein tetramerization | 4 | 1.139601 | 0.04927 | 348 | 4.825287 | 0.259518 |
| GOTERM_ | GO:0048839~inner ear development | 4 | 1.139601 | 0.052375 | 348 | 4.707597 | 0.27189 |
| GOTERM_ | GO:0030838~positive regulation of actin filament polymerization | 4 | 1.139601 | 0.065704 | 348 | 4.289144 | 0.313021 |
| GOTERM_ | GO:0045165~cell fate commitment | 4 | 1.139601 | 0.069256 | 348 | 4.195902 | 0.326761 |
| GOTERM_ | GO:0030900~forebrain development | 4 | 1.139601 | 0.069256 | 348 | 4.195902 | 0.326761 |
| GOTERM_ | GO:0019722~calcium-mediated signaling | 4 | 1.139601 | 0.088264 | 348 | 3.784539 | 0.379786 |

| | | | | | | | |
|---|---|---|---|---|---|---|---|
| GOTERM_ | GO:0042542~response to hydrogen peroxide | 4 | 1.139601 | 0.088264 | 348 | 3.784539 | 0.379786 |
| GOTERM_ | GO:0034097~response to cytokine | 4 | 1.139601 | 0.092304 | 348 | 3.71176 | 0.395079 |
| GOTERM_ | GO:0008344~adult locomotory behavior | 4 | 1.139601 | 0.092304 | 348 | 3.71176 | 0.395079 |
| GOTERM_ | GO:0040008~regulation of growth | 4 | 1.139601 | 0.096418 | 348 | 3.641726 | 0.403503 |
| GOTERM_ | GO:0070434~positive regulation of nucleotide-binding oligomerization domain containing 2 | 3 | 0.854701 | 0.00126 | 348 | 48.25287 | 0.016414 |
| GOTERM_ | GO:0021828~gonadotrophin-releasing hormone neuronal migration to the hypothalamus | 3 | 0.854701 | 0.00126 | 348 | 48.25287 | 0.016414 |
| GOTERM_ | GO:0060688~regulation of morphogenesis of a branching structure | 3 | 0.854701 | 0.00126 | 348 | 48.25287 | 0.016414 |
| GOTERM_ | GO:1903375~facioacoustic ganglion development | 3 | 0.854701 | 0.00126 | 348 | 48.25287 | 0.016414 |
| GOTERM_ | GO:0071727~cellular response to triacyl bacterial lipopeptide | 3 | 0.854701 | 0.00126 | 348 | 48.25287 | 0.016414 |
| GOTERM_ | GO:0038123~toll-like receptor TLR1:TLR2 signaling pathway | 3 | 0.854701 | 0.00126 | 348 | 48.25287 | 0.016414 |
| GOTERM_ | GO:0002741~positive regulation of cytokine secretion involved in immune response | 3 | 0.854701 | 0.002485 | 348 | 36.18966 | 0.026998 |
| GOTERM_ | GO:0097491~sympathetic neuron projection guidance | 3 | 0.854701 | 0.002485 | 348 | 36.18966 | 0.026998 |
| GOTERM_ | GO:0097490~sympathetic neuron projection extension | 3 | 0.854701 | 0.002485 | 348 | 36.18966 | 0.026998 |
| GOTERM_ | GO:0036486~ventral trunk neural crest cell migration | 3 | 0.854701 | 0.002485 | 348 | 36.18966 | 0.026998 |
| GOTERM_ | GO:1902285~semaphorin-plexin signaling pathway involved in neuron projection guidance | 3 | 0.854701 | 0.002485 | 348 | 36.18966 | 0.026998 |
| GOTERM_ | GO:0048861~leukemia inhibitory factor signaling pathway | 3 | 0.854701 | 0.002485 | 348 | 36.18966 | 0.026998 |
| GOTERM_ | GO:1905007~positive regulation of epithelial to mesenchymal transition involved in endocar | 3 | 0.854701 | 0.004086 | 348 | 28.95172 | 0.038856 |
| GOTERM_ | GO:0060666~dichotomous subdivision of terminal units involved in salivary gland branching | 3 | 0.854701 | 0.004086 | 348 | 28.95172 | 0.038856 |
| GOTERM_ | GO:0009597~detection of virus | 3 | 0.854701 | 0.004086 | 348 | 28.95172 | 0.038856 |
| GOTERM_ | GO:0021637~trigeminal nerve structural organization | 3 | 0.854701 | 0.004086 | 348 | 28.95172 | 0.038856 |
| GOTERM_ | GO:0061551~trigeminal ganglion development | 3 | 0.854701 | 0.004086 | 348 | 28.95172 | 0.038856 |
| GOTERM_ | GO:0002381~immunoglobulin production involved in immunoglobulin mediated immune res | 3 | 0.854701 | 0.004086 | 348 | 28.95172 | 0.038856 |
| GOTERM_ | GO:0050691~regulation of defense response to virus by host | 3 | 0.854701 | 0.004086 | 348 | 28.95172 | 0.038856 |
| GOTERM_ | GO:0051964~negative regulation of synapse assembly | 3 | 0.854701 | 0.004086 | 348 | 28.95172 | 0.038856 |
| GOTERM_ | GO:1901166~neural crest cell migration involved in autonomic nervous system development | 3 | 0.854701 | 0.004086 | 348 | 28.95172 | 0.038856 |
| GOTERM_ | GO:0060754~positive regulation of mast cell chemotaxis | 3 | 0.854701 | 0.006045 | 348 | 24.12644 | 0.053532 |
| GOTERM_ | GO:0060137~maternal process involved in parturition | 3 | 0.854701 | 0.006045 | 348 | 24.12644 | 0.053532 |
| GOTERM_ | GO:0030031~cell projection assembly | 3 | 0.854701 | 0.006045 | 348 | 24.12644 | 0.053532 |
| GOTERM_ | GO:0048841~regulation of axon extension involved in axon guidance | 3 | 0.854701 | 0.008348 | 348 | 20.6798 | 0.069635 |
| GOTERM_ | GO:0060687~regulation of branching involved in prostate gland morphogenesis | 3 | 0.854701 | 0.008348 | 348 | 20.6798 | 0.069635 |
| GOTERM_ | GO:0007435~salivary gland morphogenesis | 3 | 0.854701 | 0.008348 | 348 | 20.6798 | 0.069635 |
| GOTERM_ | GO:0060463~lung lobe morphogenesis | 3 | 0.854701 | 0.008348 | 348 | 20.6798 | 0.069635 |
| GOTERM_ | GO:0090277~positive regulation of peptide hormone secretion | 3 | 0.854701 | 0.01098 | 348 | 18.09483 | 0.086285 |
| GOTERM_ | GO:0002438~acute inflammatory response to antigenic stimulus | 3 | 0.854701 | 0.01098 | 348 | 18.09483 | 0.086285 |
| GOTERM_ | GO:0090037~positive regulation of protein kinase C signaling | 3 | 0.854701 | 0.01098 | 348 | 18.09483 | 0.086285 |
| GOTERM_ | GO:0035234~ectopic germ cell programmed cell death | 3 | 0.854701 | 0.01098 | 348 | 18.09483 | 0.086285 |
| GOTERM_ | GO:0042119~neutrophil activation | 3 | 0.854701 | 0.01098 | 348 | 18.09483 | 0.086285 |
| GOTERM_ | GO:0045410~positive regulation of interleukin-6 biosynthetic process | 3 | 0.854701 | 0.01098 | 348 | 18.09483 | 0.086285 |
| GOTERM_ | GO:0034128~negative regulation of MyD88-independent toll-like receptor signaling pathway | 3 | 0.854701 | 0.01098 | 348 | 18.09483 | 0.086285 |
| GOTERM_ | GO:0048762~mesenchymal cell differentiation | 3 | 0.854701 | 0.01098 | 348 | 18.09483 | 0.086285 |
| GOTERM_ | GO:0050778~positive regulation of immune response | 3 | 0.854701 | 0.013926 | 348 | 16.08429 | 0.101594 |
| GOTERM_ | GO:0050764~regulation of phagocytosis | 3 | 0.854701 | 0.013926 | 348 | 16.08429 | 0.101594 |
| GOTERM_ | GO:0016331~morphogenesis of embryonic epithelium | 3 | 0.854701 | 0.013926 | 348 | 16.08429 | 0.101594 |

| | | | | | | | |
|---|---|---|---|---|---|---|---|
| GOTERM_ | GO:0061549~sympathetic ganglion development | 3 | 0.854701 | 0.013926 | 348 | 16.08429 | 0.101594 |
| GOTERM_ | GO:0035456~response to interferon-beta | 3 | 0.854701 | 0.013926 | 348 | 16.08429 | 0.101594 |
| GOTERM_ | GO:0060670~branching involved in labyrinthine layer morphogenesis | 3 | 0.854701 | 0.013926 | 348 | 16.08429 | 0.101594 |
| GOTERM_ | GO:0002675~positive regulation of acute inflammatory response | 3 | 0.854701 | 0.017171 | 348 | 14.47586 | 0.117884 |
| GOTERM_ | GO:0042116~macrophage activation | 3 | 0.854701 | 0.017171 | 348 | 14.47586 | 0.117884 |
| GOTERM_ | GO:0010884~positive regulation of lipid storage | 3 | 0.854701 | 0.017171 | 348 | 14.47586 | 0.117884 |
| GOTERM_ | GO:0032725~positive regulation of granulocyte macrophage colony-stimulating factor produ | 3 | 0.854701 | 0.017171 | 348 | 14.47586 | 0.117884 |
| GOTERM_ | GO:2000406~positive regulation of T cell migration | 3 | 0.854701 | 0.017171 | 348 | 14.47586 | 0.117884 |
| GOTERM_ | GO:0010820~positive regulation of T cell chemotaxis | 3 | 0.854701 | 0.017171 | 348 | 14.47586 | 0.117884 |
| GOTERM_ | GO:0003149~membranous septum morphogenesis | 3 | 0.854701 | 0.017171 | 348 | 14.47586 | 0.117884 |
| GOTERM_ | GO:0090084~negative regulation of inclusion body assembly | 3 | 0.854701 | 0.017171 | 348 | 14.47586 | 0.117884 |
| GOTERM_ | GO:0045080~positive regulation of chemokine biosynthetic process | 3 | 0.854701 | 0.017171 | 348 | 14.47586 | 0.117884 |
| GOTERM_ | GO:0050707~regulation of cytokine secretion | 3 | 0.854701 | 0.020704 | 348 | 13.15987 | 0.135293 |
| GOTERM_ | GO:0042535~positive regulation of tumor necrosis factor biosynthetic process | 3 | 0.854701 | 0.020704 | 348 | 13.15987 | 0.135293 |
| GOTERM_ | GO:0002446~neutrophil mediated immunity | 3 | 0.854701 | 0.020704 | 348 | 13.15987 | 0.135293 |
| GOTERM_ | GO:0051024~positive regulation of immunoglobulin secretion | 3 | 0.854701 | 0.020704 | 348 | 13.15987 | 0.135293 |
| GOTERM_ | GO:0060445~branching involved in salivary gland morphogenesis | 3 | 0.854701 | 0.020704 | 348 | 13.15987 | 0.135293 |
| GOTERM_ | GO:0032495~response to muramyl dipeptide | 3 | 0.854701 | 0.020704 | 348 | 13.15987 | 0.135293 |
| GOTERM_ | GO:0003181~atrioventricular valve morphogenesis | 3 | 0.854701 | 0.020704 | 348 | 13.15987 | 0.135293 |
| GOTERM_ | GO:0002523~leukocyte migration involved in inflammatory response | 3 | 0.854701 | 0.020704 | 348 | 13.15987 | 0.135293 |
| GOTERM_ | GO:0034116~positive regulation of heterotypic cell-cell adhesion | 3 | 0.854701 | 0.020704 | 348 | 13.15987 | 0.135293 |
| GOTERM_ | GO:0001946~lymphangiogenesis | 3 | 0.854701 | 0.020704 | 348 | 13.15987 | 0.135293 |
| GOTERM_ | GO:0010447~response to acidic pH | 3 | 0.854701 | 0.024509 | 348 | 12.06322 | 0.152032 |
| GOTERM_ | GO:0045078~positive regulation of interferon-gamma biosynthetic process | 3 | 0.854701 | 0.024509 | 348 | 12.06322 | 0.152032 |
| GOTERM_ | GO:0043032~positive regulation of macrophage activation | 3 | 0.854701 | 0.024509 | 348 | 12.06322 | 0.152032 |
| GOTERM_ | GO:0045779~negative regulation of bone resorption | 3 | 0.854701 | 0.024509 | 348 | 12.06322 | 0.152032 |
| GOTERM_ | GO:0030728~ovulation | 3 | 0.854701 | 0.024509 | 348 | 12.06322 | 0.152032 |
| GOTERM_ | GO:0050921~positive regulation of chemotaxis | 3 | 0.854701 | 0.024509 | 348 | 12.06322 | 0.152032 |
| GOTERM_ | GO:0045086~positive regulation of interleukin-2 biosynthetic process | 3 | 0.854701 | 0.024509 | 348 | 12.06322 | 0.152032 |
| GOTERM_ | GO:0071498~cellular response to fluid shear stress | 3 | 0.854701 | 0.024509 | 348 | 12.06322 | 0.152032 |
| GOTERM_ | GO:0050777~negative regulation of immune response | 3 | 0.854701 | 0.024509 | 348 | 12.06322 | 0.152032 |
| GOTERM_ | GO:0060394~negative regulation of pathway-restricted SMAD protein phosphorylation | 3 | 0.854701 | 0.024509 | 348 | 12.06322 | 0.152032 |
| GOTERM_ | GO:0045778~positive regulation of ossification | 3 | 0.854701 | 0.024509 | 348 | 12.06322 | 0.152032 |
| GOTERM_ | GO:0045780~positive regulation of bone resorption | 3 | 0.854701 | 0.028575 | 348 | 11.13528 | 0.172016 |
| GOTERM_ | GO:0035810~positive regulation of urine volume | 3 | 0.854701 | 0.028575 | 348 | 11.13528 | 0.172016 |
| GOTERM_ | GO:2000573~positive regulation of DNA biosynthetic process | 3 | 0.854701 | 0.028575 | 348 | 11.13528 | 0.172016 |
| GOTERM_ | GO:0006809~nitric oxide biosynthetic process | 3 | 0.854701 | 0.028575 | 348 | 11.13528 | 0.172016 |
| GOTERM_ | GO:0003215~cardiac right ventricle morphogenesis | 3 | 0.854701 | 0.028575 | 348 | 11.13528 | 0.172016 |
| GOTERM_ | GO:0045651~positive regulation of macrophage differentiation | 3 | 0.854701 | 0.028575 | 348 | 11.13528 | 0.172016 |
| GOTERM_ | GO:0032026~response to magnesium ion | 3 | 0.854701 | 0.028575 | 348 | 11.13528 | 0.172016 |
| GOTERM_ | GO:0002756~MyD88-independent toll-like receptor signaling pathway | 3 | 0.854701 | 0.028575 | 348 | 11.13528 | 0.172016 |
| GOTERM_ | GO:0001516~prostaglandin biosynthetic process | 3 | 0.854701 | 0.03289 | 348 | 10.3399 | 0.193694 |
| GOTERM_ | GO:0048009~insulin-like growth factor receptor signaling pathway | 3 | 0.854701 | 0.03289 | 348 | 10.3399 | 0.193694 |

| | | | | | | | |
|---|---|---|---|---|---|---|---|
| GOTERM_ | GO:0043537~negative regulation of blood vessel endothelial cell migration | 3 | 0.854701 | 0.03289 | 348 | 10.3399 | 0.193694 |
| GOTERM_ | GO:0070498~interleukin-1-mediated signaling pathway | 3 | 0.854701 | 0.03289 | 348 | 10.3399 | 0.193694 |
| GOTERM_ | GO:0033627~cell adhesion mediated by integrin | 3 | 0.854701 | 0.037441 | 348 | 9.650575 | 0.216325 |
| GOTERM_ | GO:0030502~negative regulation of bone mineralization | 3 | 0.854701 | 0.037441 | 348 | 9.650575 | 0.216325 |
| GOTERM_ | GO:0048485~sympathetic nervous system development | 3 | 0.854701 | 0.037441 | 348 | 9.650575 | 0.216325 |
| GOTERM_ | GO:0048011~neurotrophin TRK receptor signaling pathway | 3 | 0.854701 | 0.042217 | 348 | 9.047414 | 0.228268 |
| GOTERM_ | GO:0090280~positive regulation of calcium ion import | 3 | 0.854701 | 0.042217 | 348 | 9.047414 | 0.228268 |
| GOTERM_ | GO:0010613~positive regulation of cardiac muscle hypertrophy | 3 | 0.854701 | 0.042217 | 348 | 9.047414 | 0.228268 |
| GOTERM_ | GO:0048566~embryonic digestive tract development | 3 | 0.854701 | 0.042217 | 348 | 9.047414 | 0.228268 |
| GOTERM_ | GO:0051895~negative regulation of focal adhesion assembly | 3 | 0.854701 | 0.042217 | 348 | 9.047414 | 0.228268 |
| GOTERM_ | GO:0003222~ventricular trabecula myocardium morphogenesis | 3 | 0.854701 | 0.042217 | 348 | 9.047414 | 0.228268 |
| GOTERM_ | GO:0048535~lymph node development | 3 | 0.854701 | 0.047206 | 348 | 8.515213 | 0.249181 |
| GOTERM_ | GO:0042523~positive regulation of tyrosine phosphorylation of Stat5 protein | 3 | 0.854701 | 0.047206 | 348 | 8.515213 | 0.249181 |
| GOTERM_ | GO:0010460~positive regulation of heart rate | 3 | 0.854701 | 0.047206 | 348 | 8.515213 | 0.249181 |
| GOTERM_ | GO:0050870~positive regulation of T cell activation | 3 | 0.854701 | 0.052398 | 348 | 8.042146 | 0.27189 |
| GOTERM_ | GO:0007250~activation of NF-kappaB-inducing kinase activity | 3 | 0.854701 | 0.052398 | 348 | 8.042146 | 0.27189 |
| GOTERM_ | GO:0035066~positive regulation of histone acetylation | 3 | 0.854701 | 0.052398 | 348 | 8.042146 | 0.27189 |
| GOTERM_ | GO:0045807~positive regulation of endocytosis | 3 | 0.854701 | 0.052398 | 348 | 8.042146 | 0.27189 |
| GOTERM_ | GO:0070266~necroptotic process | 3 | 0.854701 | 0.052398 | 348 | 8.042146 | 0.27189 |
| GOTERM_ | GO:0045089~positive regulation of innate immune response | 3 | 0.854701 | 0.052398 | 348 | 8.042146 | 0.27189 |
| GOTERM_ | GO:0032332~positive regulation of chondrocyte differentiation | 3 | 0.854701 | 0.057782 | 348 | 7.618875 | 0.293305 |
| GOTERM_ | GO:0043011~myeloid dendritic cell differentiation | 3 | 0.854701 | 0.057782 | 348 | 7.618875 | 0.293305 |
| GOTERM_ | GO:0071375~cellular response to peptide hormone stimulus | 3 | 0.854701 | 0.063349 | 348 | 7.237931 | 0.302984 |
| GOTERM_ | GO:0035584~calcium-mediated signaling using intracellular calcium source | 3 | 0.854701 | 0.063349 | 348 | 7.237931 | 0.302984 |
| GOTERM_ | GO:0001892~embryonic placenta development | 3 | 0.854701 | 0.063349 | 348 | 7.237931 | 0.302984 |
| GOTERM_ | GO:1902895~positive regulation of pri-miRNA transcription from RNA polymerase II prom | 3 | 0.854701 | 0.063349 | 348 | 7.237931 | 0.302984 |
| GOTERM_ | GO:0035902~response to immobilization stress | 3 | 0.854701 | 0.069088 | 348 | 6.893268 | 0.326761 |
| GOTERM_ | GO:0042730~fibrinolysis | 3 | 0.854701 | 0.069088 | 348 | 6.893268 | 0.326761 |
| GOTERM_ | GO:0034113~heterotypic cell-cell adhesion | 3 | 0.854701 | 0.07499 | 348 | 6.579937 | 0.349746 |
| GOTERM_ | GO:0001502~cartilage condensation | 3 | 0.854701 | 0.07499 | 348 | 6.579937 | 0.349746 |
| GOTERM_ | GO:0032967~positive regulation of collagen biosynthetic process | 3 | 0.854701 | 0.081046 | 348 | 6.293853 | 0.351824 |
| GOTERM_ | GO:0071398~cellular response to fatty acid | 3 | 0.854701 | 0.081046 | 348 | 6.293853 | 0.351824 |
| GOTERM_ | GO:0009617~response to bacterium | 3 | 0.854701 | 0.081046 | 348 | 6.293853 | 0.351824 |
| GOTERM_ | GO:0070371~ERK1 and ERK2 cascade | 3 | 0.854701 | 0.087247 | 348 | 6.031609 | 0.376735 |
| GOTERM_ | GO:0051602~response to electrical stimulus | 3 | 0.854701 | 0.087247 | 348 | 6.031609 | 0.376735 |
| GOTERM_ | GO:0045671~negative regulation of osteoclast differentiation | 3 | 0.854701 | 0.087247 | 348 | 6.031609 | 0.376735 |
| GOTERM_ | GO:0070372~regulation of ERK1 and ERK2 cascade | 3 | 0.854701 | 0.093584 | 348 | 5.790345 | 0.399163 |
| GOTERM_ | GO:0051090~regulation of sequence-specific DNA binding transcription factor activity | 3 | 0.854701 | 0.093584 | 348 | 5.790345 | 0.399163 |
| GOTERM_ | GO:0042495~detection of triacyl bacterial lipopeptide | 2 | 0.569801 | 0.040903 | 348 | 48.25287 | 0.225153 |
| GOTERM_ | GO:0034344~regulation of type III interferon production | 2 | 0.569801 | 0.040903 | 348 | 48.25287 | 0.225153 |
| GOTERM_ | GO:1902380~positive regulation of endoribonuclease activity | 2 | 0.569801 | 0.040903 | 348 | 48.25287 | 0.225153 |
| GOTERM_ | GO:0002730~regulation of dendritic cell cytokine production | 2 | 0.569801 | 0.040903 | 348 | 48.25287 | 0.225153 |
| GOTERM_ | GO:0090024~negative regulation of neutrophil chemotaxis | 2 | 0.569801 | 0.040903 | 348 | 48.25287 | 0.225153 |

| | | | | | | | |
|---|---|---|---|---|---|---|---|
| GOTERM_ | GO:0001912~positive regulation of leukocyte mediated cytotoxicity | 2 | 0.569801 | 0.040903 | 348 | 48.25287 | 0.225153 |
| GOTERM_ | GO:0035408~histone H3-T6 phosphorylation | 2 | 0.569801 | 0.040903 | 348 | 48.25287 | 0.225153 |
| GOTERM_ | GO:0021784~postganglionic parasympathetic fiber development | 2 | 0.569801 | 0.040903 | 348 | 48.25287 | 0.225153 |
| GOTERM_ | GO:0090291~negative regulation of osteoclast proliferation | 2 | 0.569801 | 0.040903 | 348 | 48.25287 | 0.225153 |
| GOTERM_ | GO:0070483~detection of hypoxia | 2 | 0.569801 | 0.040903 | 348 | 48.25287 | 0.225153 |
| GOTERM_ | GO:0001550~ovarian cumulus expansion | 2 | 0.569801 | 0.040903 | 348 | 48.25287 | 0.225153 |
| GOTERM_ | GO:0042704~uterine wall breakdown | 2 | 0.569801 | 0.040903 | 348 | 48.25287 | 0.225153 |
| GOTERM_ | GO:0021649~vestibulocochlear nerve structural organization | 2 | 0.569801 | 0.040903 | 348 | 48.25287 | 0.225153 |
| GOTERM_ | GO:1904722~positive regulation of mRNA endonucleolytic cleavage involved in unfolded pr | 2 | 0.569801 | 0.040903 | 348 | 48.25287 | 0.225153 |
| GOTERM_ | GO:0010512~negative regulation of phosphatidylinositol biosynthetic process | 2 | 0.569801 | 0.040903 | 348 | 48.25287 | 0.225153 |
| GOTERM_ | GO:2000538~positive regulation of B cell chemotaxis | 2 | 0.569801 | 0.040903 | 348 | 48.25287 | 0.225153 |
| GOTERM_ | GO:1904835~dorsal root ganglion morphogenesis | 2 | 0.569801 | 0.040903 | 348 | 48.25287 | 0.225153 |
| GOTERM_ | GO:0072126~positive regulation of glomerular mesangial cell proliferation | 2 | 0.569801 | 0.060725 | 348 | 32.16858 | 0.293305 |
| GOTERM_ | GO:0060128~corticotropin hormone secreting cell differentiation | 2 | 0.569801 | 0.060725 | 348 | 32.16858 | 0.293305 |
| GOTERM_ | GO:0035793~positive regulation of metanephric mesenchymal cell migration by platelet-deri | 2 | 0.569801 | 0.060725 | 348 | 32.16858 | 0.293305 |
| GOTERM_ | GO:0045726~positive regulation of integrin biosynthetic process | 2 | 0.569801 | 0.060725 | 348 | 32.16858 | 0.293305 |
| GOTERM_ | GO:0045061~thymic T cell selection | 2 | 0.569801 | 0.060725 | 348 | 32.16858 | 0.293305 |
| GOTERM_ | GO:0002282~microglial cell activation involved in immune response | 2 | 0.569801 | 0.060725 | 348 | 32.16858 | 0.293305 |
| GOTERM_ | GO:0039530~MDA-5 signaling pathway | 2 | 0.569801 | 0.060725 | 348 | 32.16858 | 0.293305 |
| GOTERM_ | GO:0051795~positive regulation of catagen | 2 | 0.569801 | 0.060725 | 348 | 32.16858 | 0.293305 |
| GOTERM_ | GO:0010829~negative regulation of glucose transport | 2 | 0.569801 | 0.060725 | 348 | 32.16858 | 0.293305 |
| GOTERM_ | GO:0060585~positive regulation of prostaglandin-endoperoxide synthase activity | 2 | 0.569801 | 0.060725 | 348 | 32.16858 | 0.293305 |
| GOTERM_ | GO:0097374~sensory neuron axon guidance | 2 | 0.569801 | 0.060725 | 348 | 32.16858 | 0.293305 |
| GOTERM_ | GO:2000391~positive regulation of neutrophil extravasation | 2 | 0.569801 | 0.060725 | 348 | 32.16858 | 0.293305 |
| GOTERM_ | GO:0090500~endocardial cushion to mesenchymal transition | 2 | 0.569801 | 0.060725 | 348 | 32.16858 | 0.293305 |
| GOTERM_ | GO:1903659~regulation of complement-dependent cytotoxicity | 2 | 0.569801 | 0.060725 | 348 | 32.16858 | 0.293305 |
| GOTERM_ | GO:0002291~T cell activation via T cell receptor contact with antigen bound to MHC molec | 2 | 0.569801 | 0.060725 | 348 | 32.16858 | 0.293305 |
| GOTERM_ | GO:0061364~apoptotic process involved in luteolysis | 2 | 0.569801 | 0.060725 | 348 | 32.16858 | 0.293305 |
| GOTERM_ | GO:0002158~osteoclast proliferation | 2 | 0.569801 | 0.060725 | 348 | 32.16858 | 0.293305 |
| GOTERM_ | GO:2001179~regulation of interleukin-10 secretion | 2 | 0.569801 | 0.060725 | 348 | 32.16858 | 0.293305 |
| GOTERM_ | GO:0003274~endocardial cushion fusion | 2 | 0.569801 | 0.060725 | 348 | 32.16858 | 0.293305 |
| GOTERM_ | GO:0034140~negative regulation of toll-like receptor 3 signaling pathway | 2 | 0.569801 | 0.060725 | 348 | 32.16858 | 0.293305 |
| GOTERM_ | GO:0060978~angiogenesis involved in coronary vascular morphogenesis | 2 | 0.569801 | 0.060725 | 348 | 32.16858 | 0.293305 |
| GOTERM_ | GO:0070487~monocyte aggregation | 2 | 0.569801 | 0.060725 | 348 | 32.16858 | 0.293305 |
| GOTERM_ | GO:0001660~fever generation | 2 | 0.569801 | 0.060725 | 348 | 32.16858 | 0.293305 |
| GOTERM_ | GO:0045079~negative regulation of chemokine biosynthetic process | 2 | 0.569801 | 0.060725 | 348 | 32.16858 | 0.293305 |
| GOTERM_ | GO:2000726~negative regulation of cardiac muscle cell differentiation | 2 | 0.569801 | 0.060725 | 348 | 32.16858 | 0.293305 |
| GOTERM_ | GO:0002693~positive regulation of cellular extravasation | 2 | 0.569801 | 0.060725 | 348 | 32.16858 | 0.293305 |
| GOTERM_ | GO:0033688~regulation of osteoblast proliferation | 2 | 0.569801 | 0.060725 | 348 | 32.16858 | 0.293305 |
| GOTERM_ | GO:0039528~cytoplasmic pattern recognition receptor signaling pathway in response to virus | 2 | 0.569801 | 0.060725 | 348 | 32.16858 | 0.293305 |
| GOTERM_ | GO:0021834~chemorepulsion involved in embryonic olfactory bulb interneuron precursor m | 2 | 0.569801 | 0.060725 | 348 | 32.16858 | 0.293305 |
| GOTERM_ | GO:0038165~oncostatin-M-mediated signaling pathway | 2 | 0.569801 | 0.080138 | 348 | 24.12644 | 0.349746 |
| GOTERM_ | GO:2000676~positive regulation of type B pancreatic cell apoptotic process | 2 | 0.569801 | 0.080138 | 348 | 24.12644 | 0.349746 |

| | | | | | | | |
|---|---|---|---|---|---|---|---|
| GOTERM_ | GO:0016199~axon midline choice point recognition | 2 | 0.569801 | 0.080138 | 348 | 24.12644 | 0.349746 |
| GOTERM_ | GO:0003104~positive regulation of glomerular filtration | 2 | 0.569801 | 0.080138 | 348 | 24.12644 | 0.349746 |
| GOTERM_ | GO:0060355~positive regulation of cell adhesion molecule production | 2 | 0.569801 | 0.080138 | 348 | 24.12644 | 0.349746 |
| GOTERM_ | GO:0002826~negative regulation of T-helper 1 type immune response | 2 | 0.569801 | 0.080138 | 348 | 24.12644 | 0.349746 |
| GOTERM_ | GO:0071726~cellular response to diacyl bacterial lipopeptide | 2 | 0.569801 | 0.080138 | 348 | 24.12644 | 0.349746 |
| GOTERM_ | GO:0032732~positive regulation of interleukin-1 production | 2 | 0.569801 | 0.080138 | 348 | 24.12644 | 0.349746 |
| GOTERM_ | GO:2000660~negative regulation of interleukin-1-mediated signaling pathway | 2 | 0.569801 | 0.080138 | 348 | 24.12644 | 0.349746 |
| GOTERM_ | GO:0045415~negative regulation of interleukin-8 biosynthetic process | 2 | 0.569801 | 0.080138 | 348 | 24.12644 | 0.349746 |
| GOTERM_ | GO:0038124~toll-like receptor TLR6:TLR2 signaling pathway | 2 | 0.569801 | 0.080138 | 348 | 24.12644 | 0.349746 |
| GOTERM_ | GO:0061041~regulation of wound healing | 2 | 0.569801 | 0.080138 | 348 | 24.12644 | 0.349746 |
| GOTERM_ | GO:0035655~interleukin-18-mediated signaling pathway | 2 | 0.569801 | 0.080138 | 348 | 24.12644 | 0.349746 |
| GOTERM_ | GO:0060686~negative regulation of prostatic bud formation | 2 | 0.569801 | 0.080138 | 348 | 24.12644 | 0.349746 |
| GOTERM_ | GO:0043152~induction of bacterial agglutination | 2 | 0.569801 | 0.080138 | 348 | 24.12644 | 0.349746 |
| GOTERM_ | GO:0060571~morphogenesis of an epithelial fold | 2 | 0.569801 | 0.080138 | 348 | 24.12644 | 0.349746 |
| GOTERM_ | GO:0032673~regulation of interleukin-4 production | 2 | 0.569801 | 0.080138 | 348 | 24.12644 | 0.349746 |
| GOTERM_ | GO:0010574~regulation of vascular endothelial growth factor production | 2 | 0.569801 | 0.080138 | 348 | 24.12644 | 0.349746 |
| GOTERM_ | GO:0060708~spongiotrophoblast differentiation | 2 | 0.569801 | 0.080138 | 348 | 24.12644 | 0.349746 |
| GOTERM_ | GO:0060485~mesenchyme development | 2 | 0.569801 | 0.080138 | 348 | 24.12644 | 0.349746 |
| GOTERM_ | GO:1903142~positive regulation of establishment of endothelial barrier | 2 | 0.569801 | 0.080138 | 348 | 24.12644 | 0.349746 |
| GOTERM_ | GO:0032369~negative regulation of lipid transport | 2 | 0.569801 | 0.080138 | 348 | 24.12644 | 0.349746 |
| GOTERM_ | GO:0072108~positive regulation of mesenchymal to epithelial transition involved in metaner | 2 | 0.569801 | 0.080138 | 348 | 24.12644 | 0.349746 |
| GOTERM_ | GO:0045906~negative regulation of vasoconstriction | 2 | 0.569801 | 0.080138 | 348 | 24.12644 | 0.349746 |
| GOTERM_ | GO:0002725~negative regulation of T cell cytokine production | 2 | 0.569801 | 0.080138 | 348 | 24.12644 | 0.349746 |
| GOTERM_ | GO:0060750~epithelial cell proliferation involved in mammary gland duct elongation | 2 | 0.569801 | 0.080138 | 348 | 24.12644 | 0.349746 |
| GOTERM_ | GO:0060841~venous blood vessel development | 2 | 0.569801 | 0.080138 | 348 | 24.12644 | 0.349746 |
| GOTERM_ | GO:0060129~thyroid-stimulating hormone-secreting cell differentiation | 2 | 0.569801 | 0.080138 | 348 | 24.12644 | 0.349746 |
| GOTERM_ | GO:0070858~negative regulation of bile acid biosynthetic process | 2 | 0.569801 | 0.080138 | 348 | 24.12644 | 0.349746 |
| GOTERM_ | GO:2000110~negative regulation of macrophage apoptotic process | 2 | 0.569801 | 0.080138 | 348 | 24.12644 | 0.349746 |
| GOTERM_ | GO:0070370~cellular heat acclimation | 2 | 0.569801 | 0.080138 | 348 | 24.12644 | 0.349746 |
| GOTERM_ | GO:0021891~olfactory bulb interneuron development | 2 | 0.569801 | 0.080138 | 348 | 24.12644 | 0.349746 |
| GOTERM_ | GO:0048608~reproductive structure development | 2 | 0.569801 | 0.080138 | 348 | 24.12644 | 0.349746 |
| GOTERM_ | GO:0050847~progesterone receptor signaling pathway | 2 | 0.569801 | 0.080138 | 348 | 24.12644 | 0.349746 |
| GOTERM_ | GO:1903553~positive regulation of extracellular exosome assembly | 2 | 0.569801 | 0.080138 | 348 | 24.12644 | 0.349746 |
| GOTERM_ | GO:0042482~positive regulation of odontogenesis | 2 | 0.569801 | 0.080138 | 348 | 24.12644 | 0.349746 |
| GOTERM_ | GO:0018119~peptidyl-cysteine S-nitrosylation | 2 | 0.569801 | 0.080138 | 348 | 24.12644 | 0.349746 |
| GOTERM_ | GO:0014826~vein smooth muscle contraction | 2 | 0.569801 | 0.080138 | 348 | 24.12644 | 0.349746 |
| GOTERM_ | GO:0045986~negative regulation of smooth muscle contraction | 2 | 0.569801 | 0.080138 | 348 | 24.12644 | 0.349746 |
| GOTERM_ | GO:0010828~positive regulation of glucose transport | 2 | 0.569801 | 0.099151 | 348 | 19.30115 | 0.403503 |
| GOTERM_ | GO:0070100~negative regulation of chemokine-mediated signaling pathway | 2 | 0.569801 | 0.099151 | 348 | 19.30115 | 0.403503 |
| GOTERM_ | GO:0048562~embryonic organ morphogenesis | 2 | 0.569801 | 0.099151 | 348 | 19.30115 | 0.403503 |
| GOTERM_ | GO:0045063~T-helper 1 cell differentiation | 2 | 0.569801 | 0.099151 | 348 | 19.30115 | 0.403503 |
| GOTERM_ | GO:0003100~regulation of systemic arterial blood pressure by endothelin | 2 | 0.569801 | 0.099151 | 348 | 19.30115 | 0.403503 |
| GOTERM_ | GO:0071801~regulation of podosome assembly | 2 | 0.569801 | 0.099151 | 348 | 19.30115 | 0.403503 |

| | | | | | | | |
|---|---|---|---|---|---|---|---|
| GOTERM_ | GO:0030823~regulation of cGMP metabolic process | 2 | 0.569801 | 0.099151 | 348 | 19.30115 | 0.403503 |
| GOTERM_ | GO:0060744~mammary gland branching involved in thelarche | 2 | 0.569801 | 0.099151 | 348 | 19.30115 | 0.403503 |
| GOTERM_ | GO:0022614~membrane to membrane docking | 2 | 0.569801 | 0.099151 | 348 | 19.30115 | 0.403503 |
| GOTERM_ | GO:0060836~lymphatic endothelial cell differentiation | 2 | 0.569801 | 0.099151 | 348 | 19.30115 | 0.403503 |
| GOTERM_ | GO:0060157~urinary bladder development | 2 | 0.569801 | 0.099151 | 348 | 19.30115 | 0.403503 |
| GOTERM_ | GO:0090027~negative regulation of monocyte chemotaxis | 2 | 0.569801 | 0.099151 | 348 | 19.30115 | 0.403503 |
| GOTERM_ | GO:0010936~negative regulation of macrophage cytokine production | 2 | 0.569801 | 0.099151 | 348 | 19.30115 | 0.403503 |
| GOTERM_ | GO:0060385~axonogenesis involved in innervation | 2 | 0.569801 | 0.099151 | 348 | 19.30115 | 0.403503 |
| GOTERM_ | GO:0042092~type 2 immune response | 2 | 0.569801 | 0.099151 | 348 | 19.30115 | 0.403503 |
| GOTERM_ | GO:0060676~ureteric bud formation | 2 | 0.569801 | 0.099151 | 348 | 19.30115 | 0.403503 |
| GOTERM_ | GO:0031622~positive regulation of fever generation | 2 | 0.569801 | 0.099151 | 348 | 19.30115 | 0.403503 |
| GOTERM_ | GO:0042531~positive regulation of tyrosine phosphorylation of STAT protein | 2 | 0.569801 | 0.099151 | 348 | 19.30115 | 0.403503 |
| GOTERM_ | GO:0034392~negative regulation of smooth muscle cell apoptotic process | 2 | 0.569801 | 0.099151 | 348 | 19.30115 | 0.403503 |
| GOTERM_ | GO:0097029~mature conventional dendritic cell differentiation | 2 | 0.569801 | 0.099151 | 348 | 19.30115 | 0.403503 |
| GOTERM_ | GO:0070120~ciliary neurotrophic factor-mediated signaling pathway | 2 | 0.569801 | 0.099151 | 348 | 19.30115 | 0.403503 |
| GOTERM_ | GO:0014910~regulation of smooth muscle cell migration | 2 | 0.569801 | 0.099151 | 348 | 19.30115 | 0.403503 |
| GOTERM_ | GO:0042743~hydrogen peroxide metabolic process | 2 | 0.569801 | 0.099151 | 348 | 19.30115 | 0.403503 |
| GOTERM_ | GO:0034123~positive regulation of toll-like receptor signaling pathway | 2 | 0.569801 | 0.099151 | 348 | 19.30115 | 0.403503 |
