# Supplementary material for: SNCA correlates with immune infiltration and serves as a prognostic biomarker in lung adenocarcinoma
Source: BMC Cancer. 2022 Apr 14;22:406. doi: 10.1186/s12885-022-09289-7 (PMC9009002; doi:10.1186/s12885-022-09289-7)
Supplement: Supplementary file 7 — Additional file 7. [file 12885_2022_9289_MOESM7_ESM.pdf]

#node1	node2	node1_string_id	node2_string_id	neighborhood_on_chromosome	gene_fusion	phylogenetic_cooccurrence	homology	coexpression	experimentally_determined_interaction	database_annotated	automated_textmining	combined_score
ACKR3	C5	9606.ENSP00000272928	9606.ENSP00000223642	0	0	0	0	0	0	0.9	0.053	0.901
ACKR3	CXCL6	9606.ENSP00000272928	9606.ENSP00000226317	0	0	0	0	0	0	0.9	0.309	0.927
ACKR3	OPRD1	9606.ENSP00000272928	9606.ENSP00000234961	0	0	0	0.681	0	0	0.9	0.189	0.904
ACKR3	C3	9606.ENSP00000272928	9606.ENSP00000245907	0	0	0	0	0	0	0.9	0	0.9
ACKR3	CCR7	9606.ENSP00000272928	9606.ENSP00000246657	0	0	0	0.759	0	0	0.9	0.577	0.913
ACKR3	KNG1	9606.ENSP00000272928	9606.ENSP00000265023	0	0	0	0	0	0	0.9	0.061	0.902
ACKR3	GAL	9606.ENSP00000272928	9606.ENSP00000265643	0	0	0	0	0	0	0.9	0.079	0.903
ACKR3	SSTR2	9606.ENSP00000272928	9606.ENSP00000350198	0	0	0	0.717	0	0	0.9	0.074	0.9
ACKR3	CORT	9606.ENSP00000272928	9606.ENSP00000366248	0	0	0	0	0	0	0.9	0	0.9
ACKR3	SAA1	9606.ENSP00000272928	9606.ENSP00000384906	0	0	0	0	0	0	0.9	0.079	0.903
ACKR3	APLN	9606.ENSP00000272928	9606.ENSP00000391800	0	0	0	0	0	0	0.9	0.111	0.907
ACKR3	S1PR2	9606.ENSP00000272928	9606.ENSP00000466933	0	0	0	0	0	0	0.9	0.116	0.907
ACKR3	S1PR1	9606.ENSP00000272928	9606.ENSP00000305416	0	0	0	0	0.077	0	0.9	0.129	0.912
ACKR3	CXCL5	9606.ENSP00000272928	9606.ENSP00000296027	0	0	0	0	0	0	0.9	0.389	0.936
ACKR3	CXCL3	9606.ENSP00000272928	9606.ENSP00000296026	0	0	0	0	0	0	0.9	0.399	0.937
ACKR3	CXCL2	9606.ENSP00000272928	9606.ENSP00000427279	0	0	0	0	0	0	0.9	0.421	0.939
ACKR3	CCL20	9606.ENSP00000272928	9606.ENSP00000351671	0	0	0	0	0	0	0.9	0.433	0.94
ACKR3	CXCL1	9606.ENSP00000272928	9606.ENSP00000379110	0	0	0	0	0	0	0.9	0.449	0.942
ACKR3	CCL5	9606.ENSP00000272928	9606.ENSP00000474412	0	0	0	0	0	0.05	0.9	0.47	0.945
ACKR3	CXCL8	9606.ENSP00000272928	9606.ENSP00000306512	0	0	0	0	0	0	0.9	0.703	0.969
ACVR1C	TGFB3	9606.ENSP00000243349	9606.ENSP00000238682	0	0	0	0	0	0.731	0	0.268	0.794
ACVR1C	GDF11	9606.ENSP00000243349	9606.ENSP00000257868	0	0	0	0	0.054	0.292	0	0.66	0.752
ACVR1C	BMP2	9606.ENSP00000243349	9606.ENSP00000368104	0	0	0	0	0	0.685	0	0.479	0.829
ACVR1C	ACVR2B	9606.ENSP00000243349	9606.ENSP00000340361	0	0	0	0.8	0.077	0.684	0.9	0.806	0.973
ACVR1C	INHBB	9606.ENSP00000243349	9606.ENSP00000295228	0	0	0	0	0	0.651	0.9	0.626	0.985
ACVR2B	GDF11	9606.ENSP00000340361	9606.ENSP00000257868	0	0	0	0	0.061	0.55	0	0.879	0.945
ACVR2B	INHBB	9606.ENSP00000340361	9606.ENSP00000295228	0	0	0	0	0	0.502	0.9	0.536	0.974
ACVR2B	BMP7	9606.ENSP00000340361	9606.ENSP00000379204	0	0	0	0	0	0.489	0	0.678	0.828
ACVR2B	ENG	9606.ENSP00000340361	9606.ENSP00000362299	0	0	0	0	0	0.372	0.9	0.358	0.956
ACVR2B	ACVRL1	9606.ENSP00000340361	9606.ENSP00000373574	0	0	0	0.75	0.063	0.732	0.9	0.62	0.976
ACVR2B	BMPR1B	9606.ENSP00000340361	9606.ENSP00000401907	0	0	0	0.778	0.077	0.735	0.9	0.764	0.977
ACVR2B	BMP2	9606.ENSP00000340361	9606.ENSP00000368104	0	0	0	0	0	0.97	0.9	0.739	0.999
ACVRL1	TGFB3	9606.ENSP00000373574	9606.ENSP00000238682	0	0	0	0	0.061	0.492	0.9	0.268	0.96
ACVRL1	TGFBR2	9606.ENSP00000373574	9606.ENSP00000351905	0	0	0	0.759	0.09	0.596	0.9	0.537	0.965
ACVRL1	ENG	9606.ENSP00000373574	9606.ENSP00000362299	0	0	0	0	0.132	0.468	0.9	0.87	0.993
ADCYAP1	PTGER2	9606.ENSP00000483721	9606.ENSP00000245457	0	0	0	0	0	0	0.9	0.195	0.916
ADCYAP1	GLP2R	9606.ENSP00000483721	9606.ENSP00000262441	0	0	0	0.789	0	0	0.9	0.337	0.906
ADCYAP1	GPR32	9606.ENSP00000483721	9606.ENSP00000270590	0	0	0	0	0	0	0.9	0	0.9
ADCYAP1	PTH1R	9606.ENSP00000483721	9606.ENSP00000321999	0	0	0	0.82	0.098	0	0.9	0.4	0.912
ADCYAP1	VIPR1	9606.ENSP00000483721	9606.ENSP00000327246	0	0	0	0.929	0	0	0.9	0.802	0.905
ADCYAP1	GIP	9606.ENSP00000483721	9606.ENSP00000350005	0	0	0	0	0	0	0.9	0.223	0.919
ADCYAP1	VIP	9606.ENSP00000483721	9606.ENSP00000356213	0	0	0	0	0.107	0.176	0.9	0.75	0.979
ADCYAP1	INSL3	9606.ENSP00000483721	9606.ENSP00000369017	0	0	0	0	0	0	0.9	0	0.9
ADCYAP1	RLN2	9606.ENSP00000483721	9606.ENSP00000371040	0	0	0	0	0	0	0.9	0	0.9
ADCYAP1	CALCRL	9606.ENSP00000483721	9606.ENSP00000386972	0	0	0	0.717	0	0	0.9	0.451	0.912
ADCYAP1	CALCB	9606.ENSP00000483721	9606.ENSP00000433490	0	0	0	0	0	0	0.9	0	0.9
ADCYAP1	ADM	9606.ENSP00000483721	9606.ENSP00000436607	0	0	0	0	0	0	0.9	0.136	0.909
ADCYAP1	PTHLH	9606.ENSP00000483721	9606.ENSP00000441765	0	0	0	0	0.061	0	0.9	0.062	0.904
ADCYAP1	MC1R	9606.ENSP00000483721	9606.ENSP00000451605	0	0	0	0	0	0	0.9	0	0.9
ADM	PTGER2	9606.ENSP00000436607	9606.ENSP00000245457	0	0	0	0	0	0	0.9	0.057	0.901
ADM	GLP2R	9606.ENSP00000436607	9606.ENSP00000262441	0	0	0	0	0	0	0.9	0	0.9
ADM	GPR32	9606.ENSP00000436607	9606.ENSP00000270590	0	0	0	0	0	0	0.9	0	0.9
ADM	PTH1R	9606.ENSP00000436607	9606.ENSP00000321999	0	0	0	0	0	0	0.9	0.202	0.916
ADM	VIPR1	9606.ENSP00000436607	9606.ENSP00000327246	0	0	0	0	0	0	0.9	0.464	0.944
ADM	GIP	9606.ENSP00000436607	9606.ENSP00000350005	0	0	0	0	0	0	0.9	0.125	0.908
ADM	VIP	9606.ENSP00000436607	9606.ENSP00000356213	0	0	0	0	0	0	0.9	0.574	0.955
ADM	EDN1	9606.ENSP00000436607	9606.ENSP00000368683	0	0	0	0	0.065	0.379	0	0.822	0.888
ADM	INSL3	9606.ENSP00000436607	9606.ENSP00000369017	0	0	0	0	0	0	0.9	0.077	0.903
ADM	RLN2	9606.ENSP00000436607	9606.ENSP00000371040	0	0	0	0	0	0	0.9	0.091	0.905
ADM	IL6	9606.ENSP00000436607	9606.ENSP00000385675	0	0	0	0	0.098	0	0	0.695	0.713
ADM	CALCRL	9606.ENSP00000436607	9606.ENSP00000386972	0	0	0	0	0	0.294	0.9	0.958	0.996
ADM	CALCB	9606.ENSP00000436607	9606.ENSP00000433490	0	0	0	0	0	0	0.9	0.755	0.974
ADM	MC1R	9606.ENSP00000436607	9606.ENSP00000451605	0	0	0	0	0	0	0.9	0.061	0.902
ADM	PTHLH	9606.ENSP00000436607	9606.ENSP00000441765	0	0	0	0	0	0	0.9	0.265	0.923
AKT3	PIK3R3	9606.ENSP00000263826	9606.ENSP00000262741	0	0	0	0	0.062	0.085	0.9	0.55	0.956
AKT3	MAPK3	9606.ENSP00000263826	9606.ENSP00000263025	0	0	0.327	0.595	0.064	0.146	0.8	0.618	0.884
AKT3	MAP3K8	9606.ENSP00000263826	9606.ENSP00000263056	0	0	0.334	0.564	0.056	0.05	0.9	0.17	0.92
AKT3	NOS1	9606.ENSP00000263826	9606.ENSP00000477999	0	0	0	0	0.049	0.065	0.8	0.066	0.811
AKT3	NOS2	9606.ENSP00000263826	9606.ENSP00000327251	0	0	0	0	0.064	0.065	0.8	0.066	0.814
AKT3	IKBKG	9606.ENSP00000263826	9606.ENSP00000483825	0	0	0	0	0	0.129	0.8	0.132	0.835
AKT3	MAP3K14	9606.ENSP00000263826	9606.ENSP00000482657	0	0	0	0.584	0.064	0.085	0.9	0.118	0.91

AKT3	FYN	9606.ENSP00000263826	9606.ENSP00000346671	0	0	0.376	0.566	0.091	0.071	0.9	0.248	0.929
AKT3	PIK3R5	9606.ENSP00000263826	9606.ENSP00000392812	0	0	0	0	0	0	0.9	0.472	0.944
AMBN	PROC	9606.ENSP00000313809	9606.ENSP00000234071	0	0	0	0	0	0	0.9	0	0.9
AMBN	FGF23	9606.ENSP00000313809	9606.ENSP00000237837	0	0	0	0	0	0	0.9	0.161	0.912
AMBN	C3	9606.ENSP00000313809	9606.ENSP00000245907	0	0	0	0	0	0	0.9	0	0.9
AMBN	KNG1	9606.ENSP00000313809	9606.ENSP00000265023	0	0	0	0	0.057	0	0.9	0.077	0.905
AMBN	SCG2	9606.ENSP00000313809	9606.ENSP00000304133	0	0	0	0	0	0	0.9	0	0.9
AMBN	FGA	9606.ENSP00000313809	9606.ENSP00000306361	0	0	0	0	0.065	0	0.9	0.116	0.91
AMBN	SDC2	9606.ENSP00000313809	9606.ENSP00000307046	0	0	0	0	0	0	0.9	0.043	0.9
AMBN	CHGB	9606.ENSP00000313809	9606.ENSP00000368244	0	0	0	0	0	0	0.9	0	0.9
AMBN	IL6	9606.ENSP00000313809	9606.ENSP00000385675	0	0	0	0	0	0	0.9	0.047	0.9
AMBN	CSF1	9606.ENSP00000313809	9606.ENSP00000327513	0	0	0	0	0	0	0.9	0.064	0.902
AMBN	SPP1	9606.ENSP00000313809	9606.ENSP00000378517	0	0	0	0	0.063	0	0.9	0.442	0.943
ANGPTL4	RXRA	9606.ENSP00000301455	9606.ENSP00000419692	0	0	0	0	0.062	0.05	0.9	0.211	0.92
APLN	C5	9606.ENSP00000391800	9606.ENSP00000223642	0	0	0	0	0	0	0.9	0	0.9
APLN	CXCL6	9606.ENSP00000391800	9606.ENSP00000226317	0	0	0	0	0	0	0.9	0	0.9
APLN	OPRD1	9606.ENSP00000391800	9606.ENSP00000234961	0	0	0	0	0	0	0.9	0.213	0.917
APLN	C3	9606.ENSP00000391800	9606.ENSP00000245907	0	0	0	0	0	0	0.9	0	0.9
APLN	CCR7	9606.ENSP00000391800	9606.ENSP00000246657	0	0	0	0	0	0	0.9	0	0.9
APLN	KNG1	9606.ENSP00000391800	9606.ENSP00000265023	0	0	0	0	0	0	0.9	0.347	0.931
APLN	GAL	9606.ENSP00000391800	9606.ENSP00000265643	0	0	0	0	0	0	0.9	0.25	0.921
APLN	CXCL3	9606.ENSP00000391800	9606.ENSP00000296026	0	0	0	0	0	0	0.9	0	0.9
APLN	CXCL5	9606.ENSP00000391800	9606.ENSP00000296027	0	0	0	0	0	0	0.9	0	0.9
APLN	S1PR1	9606.ENSP00000391800	9606.ENSP00000305416	0	0	0	0	0.065	0	0.9	0.056	0.904
APLN	CXCL8	9606.ENSP00000391800	9606.ENSP00000306512	0	0	0	0	0	0	0.9	0.2	0.916
APLN	SSTR2	9606.ENSP00000391800	9606.ENSP00000350198	0	0	0	0	0	0	0.9	0.123	0.908
APLN	CCL20	9606.ENSP00000391800	9606.ENSP00000351671	0	0	0	0	0	0	0.9	0.046	0.9
APLN	CORT	9606.ENSP00000391800	9606.ENSP00000366248	0	0	0	0	0	0	0.9	0.076	0.903
APLN	CXCL1	9606.ENSP00000391800	9606.ENSP00000379110	0	0	0	0	0	0	0.9	0.047	0.9
APLN	SAA1	9606.ENSP00000391800	9606.ENSP00000384906	0	0	0	0	0	0	0.9	0.069	0.902
APLN	S1PR2	9606.ENSP00000391800	9606.ENSP00000466933	0	0	0	0	0	0	0.9	0	0.9
APLN	CXCL2	9606.ENSP00000391800	9606.ENSP00000427279	0	0	0	0	0	0	0.9	0	0.9
APLN	CCL5	9606.ENSP00000391800	9606.ENSP00000474412	0	0	0	0	0	0	0.9	0.067	0.902
APOH	PLTP	9606.ENSP00000205948	9606.ENSP00000417138	0	0	0	0	0	0	0.72	0.278	0.789
APOH	FGA	9606.ENSP00000205948	9606.ENSP00000306361	0	0	0	0	0.668	0.157	0	0.5	0.848
AREG	EREG	9606.ENSP00000379097	9606.ENSP00000244869	0	0	0	0	0.202	0	0	0.915	0.929
AREG	ICAM1	9606.ENSP00000379097	9606.ENSP00000264832	0	0	0	0	0.076	0.534	0	0.595	0.81
AREG	IGF1R	9606.ENSP00000379097	9606.ENSP00000268035	0	0	0	0	0	0	0.6	0.473	0.78
AREG	EGFR	9606.ENSP00000379097	9606.ENSP00000275493	0	0	0	0	0	0.379	0.9	0.919	0.994
AREG	TGFA	9606.ENSP00000379097	9606.ENSP00000295400	0	0	0	0	0.088	0	0.9	0.786	0.978
AREG	MET	9606.ENSP00000379097	9606.ENSP00000317272	0	0	0	0	0.076	0	0.6	0.44	0.774
AREG	BTC	9606.ENSP00000379097	9606.ENSP00000379092	0	0	0	0	0.083	0	0	0.884	0.889
AREG	IL6	9606.ENSP00000379097	9606.ENSP00000385675	0	0	0	0	0.096	0	0	0.698	0.716
ARG2	NOS1	9606.ENSP00000261783	9606.ENSP00000477999	0	0	0	0	0.06	0.266	0.9	0.574	0.966
ARG2	NOS2	9606.ENSP00000261783	9606.ENSP00000327251	0	0	0	0	0.06	0.086	0.9	0.81	0.981
ARTN	PLCG1	9606.ENSP00000387435	9606.ENSP00000244007	0	0	0	0	0	0	0.9	0.049	0.9
ARTN	PIK3R3	9606.ENSP00000387435	9606.ENSP00000262741	0	0	0	0	0	0	0.9	0	0.9
ARTN	NRTN	9606.ENSP00000387435	9606.ENSP00000302648	0	0	0	0.764	0.065	0	0.9	0.932	0.923
ARTN	SHC3	9606.ENSP00000387435	9606.ENSP00000364995	0	0	0	0	0	0	0.9	0.049	0.9
ARTN	PRKCA	9606.ENSP00000387435	9606.ENSP00000408695	0	0	0	0	0	0	0.9	0.08	0.904
ARTN	SHC1	9606.ENSP00000387435	9606.ENSP00000401303	0	0	0	0	0	0	0.9	0.112	0.907
ARTN	GDNF	9606.ENSP00000387435	9606.ENSP00000409007	0	0	0	0.694	0.061	0	0.9	0.899	0.928
BCL3	RELB	9606.ENSP00000164227	9606.ENSP00000221452	0	0.004	0	0	0.483	0.468	0.54	0.711	0.958
BDNF	NGFR	9606.ENSP00000414303	9606.ENSP00000172229	0	0	0	0	0	0.298	0.9	0.948	0.996
BDNF	PLCG1	9606.ENSP00000414303	9606.ENSP00000244007	0	0	0	0	0	0	0.9	0.641	0.962
BDNF	IL1B	9606.ENSP00000414303	9606.ENSP00000263341	0	0	0	0	0	0	0	0.798	0.798
BDNF	FGF2	9606.ENSP00000414303	9606.ENSP00000264498	0	0	0	0	0.11	0	0	0.906	0.913
BDNF	SHC2	9606.ENSP00000414303	9606.ENSP00000264554	0	0	0	0	0	0	0.9	0.282	0.925
BDNF	SEMA3A	9606.ENSP00000414303	9606.ENSP00000265362	0	0	0	0	0.084	0	0	0.702	0.715
BDNF	IGF1R	9606.ENSP00000414303	9606.ENSP00000268035	0	0	0	0	0	0.185	0.6	0.427	0.796
BDNF	EGFR	9606.ENSP00000414303	9606.ENSP00000275493	0	0	0	0	0.132	0	0.6	0.541	0.826
BDNF	FLT1	9606.ENSP00000414303	9606.ENSP00000282397	0	0	0	0	0	0	0.6	0.292	0.704
BDNF	MET	9606.ENSP00000414303	9606.ENSP00000317272	0	0	0	0	0.076	0.185	0.6	0.269	0.75
BDNF	FGF13	9606.ENSP00000414303	9606.ENSP00000322390	0	0	0	0	0	0	0	0.747	0.747
BDNF	NGF	9606.ENSP00000414303	9606.ENSP00000358525	0	0	0	0.841	0.1	0	0.9	0.972	0.92
BDNF	SHC3	9606.ENSP00000414303	9606.ENSP00000364995	0	0	0	0	0.088	0	0.9	0.319	0.932
BDNF	CORT	9606.ENSP00000414303	9606.ENSP00000366248	0	0	0	0	0	0	0	0.743	0.743
BDNF	NRG1	9606.ENSP00000414303	9606.ENSP00000384620	0	0	0	0	0.088	0	0	0.776	0.788
BDNF	IL6	9606.ENSP00000414303	9606.ENSP00000385675	0	0	0	0	0	0	0	0.811	0.811
BDNF	SHC1	9606.ENSP00000414303	9606.ENSP00000401303	0	0	0	0	0	0	0.9	0.553	0.953
BDNF	GDNF	9606.ENSP00000414303	9606.ENSP00000409007	0	0	0	0	0	0	0	0.957	0.958
BLNK	PIK3R3	9606.ENSP00000224337	9606.ENSP00000262741	0	0	0	0	0	0	0.9	0	0.9

BLNK	SHC1	9606.ENSP00000224337	9606.ENSP00000401303	0	0	0	0	0	0	0.9	0.292	0.926
BLNK	FYN	9606.ENSP00000224337	9606.ENSP00000346671	0	0	0	0	0	0.078	0.9	0.441	0.943
BLNK	HCK	9606.ENSP00000224337	9606.ENSP00000444986	0	0	0	0	0.158	0.078	0.9	0.441	0.95
BLNK	SRC	9606.ENSP00000224337	9606.ENSP00000362680	0	0	0	0	0	0.078	0.9	0.532	0.953
BLNK	PTPN6	9606.ENSP00000224337	9606.ENSP00000391592	0	0	0	0	0.17	0.379	0.9	0.243	0.955
BLNK	PLCG1	9606.ENSP00000224337	9606.ENSP00000244007	0	0	0	0	0.064	0.465	0.9	0.386	0.965
BLNK	SYK	9606.ENSP00000224337	9606.ENSP00000364907	0	0	0	0	0.179	0.446	0.9	0.949	0.997
BMP1	BMP2	9606.ENSP00000305714	9606.ENSP00000368104	0	0	0	0	0.063	0.33	0	0.743	0.824
BMP2	FGF2	9606.ENSP00000368104	9606.ENSP00000264498	0	0	0	0	0	0.05	0	0.845	0.847
BMP2	ENG	9606.ENSP00000368104	9606.ENSP00000362299	0	0	0	0	0.061	0.342	0	0.734	0.821
BMP2	IL6	9606.ENSP00000368104	9606.ENSP00000385675	0	0	0	0	0.065	0	0	0.714	0.722
BMP2	PTHLH	9606.ENSP00000368104	9606.ENSP00000441765	0	0	0	0	0	0	0	0.755	0.755
BMP2	SPP1	9606.ENSP00000368104	9606.ENSP00000378517	0	0	0	0	0	0	0	0.879	0.88
BMP2	BMP7	9606.ENSP00000368104	9606.ENSP00000379204	0	0	0	0.795	0.06	0.177	0.9	0.926	0.931
BMP2	GREM1	9606.ENSP00000368104	9606.ENSP00000478319	0	0	0	0	0	0.294	0.9	0.821	0.986
BMP2	BMPR1B	9606.ENSP00000368104	9606.ENSP00000401907	0	0	0	0	0	0.828	0.9	0.778	0.995
BMP7	CDH1	9606.ENSP00000379204	9606.ENSP00000261769	0	0	0	0	0.061	0.064	0	0.713	0.725
BMP7	GDF7	9606.ENSP00000379204	9606.ENSP00000272224	0	0	0	0.69	0	0.472	0.8	0.653	0.911
BMP7	CTGF	9606.ENSP00000379204	9606.ENSP00000356954	0	0	0	0	0	0	0	0.732	0.732
BMP7	GREM1	9606.ENSP00000379204	9606.ENSP00000478319	0	0	0	0	0	0	0	0.821	0.822
BMP7	BMPR1B	9606.ENSP00000379204	9606.ENSP00000401907	0	0	0	0	0	0.541	0.9	0.758	0.987
BMPR1B	TGFB3	9606.ENSP00000401907	9606.ENSP00000238682	0	0	0	0	0.061	0.731	0	0.223	0.786
BMPR1B	GDF7	9606.ENSP00000401907	9606.ENSP00000272224	0	0	0	0	0	0.292	0.8	0.428	0.912
BMPR1B	GDF6	9606.ENSP00000401907	9606.ENSP00000287020	0	0	0	0	0	0.492	0.6	0.499	0.889
BMPR1B	GREM1	9606.ENSP00000401907	9606.ENSP00000478319	0	0	0	0	0.065	0	0.9	0.51	0.95
BST2	GBP2	9606.ENSP00000252593	9606.ENSP00000359497	0	0	0	0	0.132	0	0.9	0.141	0.919
BST2	IRF5	9606.ENSP00000252593	9606.ENSP00000349770	0	0	0	0	0.076	0	0.9	0.214	0.921
BST2	HLA-B	9606.ENSP00000252593	9606.ENSP00000399168	0	0	0	0	0.129	0	0.9	0.265	0.93
BST2	PSMB8	9606.ENSP00000252593	9606.ENSP00000364016	0	0	0	0	0.302	0	0.9	0.142	0.934
BST2	ISG20	9606.ENSP00000252593	9606.ENSP00000306565	0	0	0	0	0.12	0	0.9	0.471	0.949
BST2	IRF9	9606.ENSP00000252593	9606.ENSP00000380073	0	0	0	0	0.197	0	0.9	0.447	0.951
BST2	IFITM1	9606.ENSP00000252593	9606.ENSP00000386187	0	0	0	0	0.339	0	0.9	0.542	0.967
BST2	OAS1	9606.ENSP00000252593	9606.ENSP00000388001	0	0	0	0	0.429	0	0.9	0.53	0.97
BST2	IRF7	9606.ENSP00000252593	9606.ENSP00000380697	0	0	0	0	0.411	0	0.9	0.553	0.971
BST2	MX1	9606.ENSP00000252593	9606.ENSP00000381601	0	0	0	0	0.424	0	0.9	0.632	0.976
BTC	EREG	9606.ENSP00000379092	9606.ENSP00000244869	0	0	0	0.666	0	0	0.9	0.901	0.929
BTC	EGFR	9606.ENSP00000379092	9606.ENSP00000275493	0	0	0	0	0.098	0.472	0.9	0.74	0.985
BTC	FYN	9606.ENSP00000379092	9606.ENSP00000346671	0	0	0	0	0	0	0.9	0.095	0.905
BTC	SRC	9606.ENSP00000379092	9606.ENSP00000362680	0	0	0	0	0	0	0.9	0.353	0.932
BTC	SHC1	9606.ENSP00000379092	9606.ENSP00000401303	0	0	0	0	0	0	0.9	0.325	0.929
BTC	NRG1	9606.ENSP00000379092	9606.ENSP00000384620	0	0	0	0	0	0	0.9	0.609	0.959
C3	C5	9606.ENSP00000245907	9606.ENSP00000223642	0	0	0	0.754	0.063	0.379	0.9	0.73	0.947
C3	CXCL6	9606.ENSP00000245907	9606.ENSP00000226317	0	0	0	0	0	0	0.9	0.087	0.904
C3	PROC	9606.ENSP00000245907	9606.ENSP00000234071	0	0	0	0	0.11	0.05	0.9	0.13	0.916
C3	OPRD1	9606.ENSP00000245907	9606.ENSP00000234961	0	0	0	0	0	0	0.9	0	0.9
C3	FGF23	9606.ENSP00000245907	9606.ENSP00000237837	0	0	0	0	0	0	0.9	0	0.9
C3	SCG2	9606.ENSP00000245907	9606.ENSP00000304133	0	0	0	0	0	0	0.9	0	0.9
C3	SSTR2	9606.ENSP00000245907	9606.ENSP00000350198	0	0	0	0	0	0	0.9	0	0.9
C3	CORT	9606.ENSP00000245907	9606.ENSP00000366248	0	0	0	0	0	0	0.9	0.052	0.901
C3	CHGB	9606.ENSP00000245907	9606.ENSP00000368244	0	0	0	0	0	0	0.9	0.066	0.902
C3	GAL	9606.ENSP00000245907	9606.ENSP00000265643	0	0	0	0	0	0	0.9	0.062	0.902
C3	S1PR1	9606.ENSP00000245907	9606.ENSP00000305416	0	0	0	0	0	0	0.9	0.085	0.904
C3	SDC2	9606.ENSP00000245907	9606.ENSP00000307046	0	0	0	0	0.062	0	0.9	0.062	0.904
C3	S1PR2	9606.ENSP00000245907	9606.ENSP00000466933	0	0	0	0	0	0	0.9	0.11	0.907
C3	IFITM1	9606.ENSP00000245907	9606.ENSP00000386187	0	0	0	0	0.061	0	0.9	0.104	0.908
C3	CXCL3	9606.ENSP00000245907	9606.ENSP00000296026	0	0	0	0	0.082	0	0.9	0.13	0.913
C3	CCR7	9606.ENSP00000245907	9606.ENSP00000246657	0	0	0	0	0	0	0.9	0.205	0.917
C3	CCL20	9606.ENSP00000245907	9606.ENSP00000351671	0	0	0	0	0.098	0	0.9	0.165	0.918
C3	CXCL5	9606.ENSP00000245907	9606.ENSP00000296027	0	0	0	0	0	0	0.9	0.232	0.919
C3	CXCL2	9606.ENSP00000245907	9606.ENSP00000427279	0	0	0	0	0.094	0	0.9	0.222	0.923
C3	CSF1	9606.ENSP00000245907	9606.ENSP00000327513	0	0	0	0	0.077	0	0.9	0.268	0.926
C3	SPP1	9606.ENSP00000245907	9606.ENSP00000378517	0	0	0	0	0.098	0	0.9	0.288	0.93
C3	CXCL1	9606.ENSP00000245907	9606.ENSP00000379110	0	0	0	0	0.105	0	0.9	0.297	0.931
C3	CCL5	9606.ENSP00000245907	9606.ENSP00000474412	0	0	0	0	0.063	0	0.9	0.356	0.934
C3	SAA1	9606.ENSP00000245907	9606.ENSP00000384906	0	0	0	0	0.125	0	0.9	0.329	0.936
C3	ELANE	9606.ENSP00000245907	9606.ENSP00000466090	0	0	0	0	0.061	0.05	0.9	0.436	0.943
C3	ITGB2	9606.ENSP00000245907	9606.ENSP00000380948	0	0	0	0	0.063	0.294	0.9	0.255	0.944
C3	FGA	9606.ENSP00000245907	9606.ENSP00000306361	0	0	0	0	0.217	0	0.9	0.359	0.945
C3	KNG1	9606.ENSP00000245907	9606.ENSP00000265023	0	0	0	0	0.094	0	0.9	0.54	0.954
C3	CXCL8	9606.ENSP00000245907	9606.ENSP00000306512	0	0	0	0	0.07	0	0.9	0.546	0.954
C3	IL6	9606.ENSP00000245907	9606.ENSP00000385675	0	0	0	0	0.071	0	0.9	0.59	0.958
C3	C5AR2	9606.ENSP00000245907	9606.ENSP00000472620	0	0	0	0	0.061	0.452	0.9	0.645	0.979

C5	SSTR2	9606.ENSP00000223642	9606.ENSP00000350198	0	0	0	0	0	0	0.9	0	0.9
C5	S1PR2	9606.ENSP00000223642	9606.ENSP00000466933	0	0	0	0	0	0	0.9	0.049	0.9
C5	OPRD1	9606.ENSP00000223642	9606.ENSP00000234961	0	0	0	0	0	0	0.9	0	0.9
C5	CORT	9606.ENSP00000223642	9606.ENSP00000366248	0	0	0	0	0	0	0.9	0	0.9
C5	GAL	9606.ENSP00000223642	9606.ENSP00000265643	0	0	0	0	0	0	0.9	0.052	0.901
C5	CXCL6	9606.ENSP00000223642	9606.ENSP00000226317	0	0	0	0	0	0	0.9	0.058	0.901
C5	S1PR1	9606.ENSP00000223642	9606.ENSP00000305416	0	0	0	0	0	0	0.9	0.056	0.901
C5	CCR7	9606.ENSP00000223642	9606.ENSP00000246657	0	0	0	0	0	0	0.9	0.053	0.901
C5	CXCL5	9606.ENSP00000223642	9606.ENSP00000296027	0	0	0	0	0	0	0.9	0.062	0.902
C5	CCL20	9606.ENSP00000223642	9606.ENSP00000351671	0	0	0	0	0	0	0.9	0.063	0.902
C5	CXCL3	9606.ENSP00000223642	9606.ENSP00000296026	0	0	0	0	0	0	0.9	0.096	0.905
C5	SAA1	9606.ENSP00000223642	9606.ENSP00000384906	0	0	0	0	0.061	0	0.9	0.108	0.908
C5	CXCL2	9606.ENSP00000223642	9606.ENSP00000427279	0	0	0	0	0	0	0.9	0.189	0.915
C5	CXCL1	9606.ENSP00000223642	9606.ENSP00000379110	0	0	0	0	0	0	0.9	0.239	0.92
C5	CCL5	9606.ENSP00000223642	9606.ENSP00000474412	0	0	0	0	0.054	0	0.9	0.243	0.922
C5	KNG1	9606.ENSP00000223642	9606.ENSP00000265023	0	0	0	0	0.064	0	0.9	0.25	0.923
C5	CXCL8	9606.ENSP00000223642	9606.ENSP00000306512	0	0	0	0	0	0	0.9	0.283	0.925
C5	ELANE	9606.ENSP00000223642	9606.ENSP00000466090	0	0	0	0	0.049	0.05	0.9	0.294	0.927
C5	VTN	9606.ENSP00000223642	9606.ENSP00000226218	0	0	0	0	0.096	0	0.9	0.33	0.934
C5	C5AR2	9606.ENSP00000223642	9606.ENSP00000472620	0	0	0	0	0	0.299	0.9	0.634	0.972
CALCB	PTGER2	9606.ENSP00000433490	9606.ENSP00000245457	0	0	0	0	0	0	0.9	0	0.9
CALCB	GLP2R	9606.ENSP00000433490	9606.ENSP00000262441	0	0	0	0	0	0	0.9	0	0.9
CALCB	GPR32	9606.ENSP00000433490	9606.ENSP00000270590	0	0	0	0	0	0	0.9	0	0.9
CALCB	PTH1R	9606.ENSP00000433490	9606.ENSP00000321999	0	0	0	0	0	0	0.9	0.083	0.904
CALCB	VIPR1	9606.ENSP00000433490	9606.ENSP00000327246	0	0	0	0	0	0	0.9	0	0.9
CALCB	GIP	9606.ENSP00000433490	9606.ENSP00000350005	0	0	0	0	0	0	0.9	0	0.9
CALCB	VIP	9606.ENSP00000433490	9606.ENSP00000356213	0	0	0	0	0.063	0	0.9	0.266	0.925
CALCB	INSL3	9606.ENSP00000433490	9606.ENSP00000369017	0	0	0	0	0	0	0.9	0.186	0.915
CALCB	RLN2	9606.ENSP00000433490	9606.ENSP00000371040	0	0	0	0	0	0	0.9	0.053	0.901
CALCB	CALCRL	9606.ENSP00000433490	9606.ENSP00000386972	0	0	0	0	0.062	0	0.9	0.712	0.97
CALCB	MC1R	9606.ENSP00000433490	9606.ENSP00000451605	0	0	0	0	0	0	0.9	0	0.9
CALCB	PTHLH	9606.ENSP00000433490	9606.ENSP00000441765	0	0	0	0	0	0	0.9	0.055	0.901
CALCRL	PTGER2	9606.ENSP00000386972	9606.ENSP00000245457	0	0	0	0	0	0	0.9	0.064	0.902
CALCRL	GLP2R	9606.ENSP00000386972	9606.ENSP00000262441	0	0	0	0.741	0	0	0.9	0.436	0.91
CALCRL	GPR32	9606.ENSP00000386972	9606.ENSP00000270590	0	0	0	0	0	0	0.9	0	0.9
CALCRL	PTH1R	9606.ENSP00000386972	9606.ENSP00000321999	0	0	0	0.798	0	0	0.9	0.447	0.908
CALCRL	VIPR1	9606.ENSP00000386972	9606.ENSP00000327246	0	0	0	0.763	0	0	0.9	0.487	0.911
CALCRL	GIP	9606.ENSP00000386972	9606.ENSP00000350005	0	0	0	0	0	0	0.9	0.198	0.916
CALCRL	VIP	9606.ENSP00000386972	9606.ENSP00000356213	0	0	0	0	0	0.176	0.9	0.419	0.947
CALCRL	INSL3	9606.ENSP00000386972	9606.ENSP00000369017	0	0	0	0	0	0	0.9	0	0.9
CALCRL	RLN2	9606.ENSP00000386972	9606.ENSP00000371040	0	0	0	0	0	0	0.9	0	0.9
CALCRL	MC1R	9606.ENSP00000386972	9606.ENSP00000451605	0	0	0	0	0	0	0.9	0.118	0.908
CALCRL	PTHLH	9606.ENSP00000386972	9606.ENSP00000441765	0	0	0	0	0	0	0.9	0.269	0.923
CARD11	PRKCB	9606.ENSP00000380150	9606.ENSP00000305355	0	0	0	0	0.095	0.456	0.9	0.42	0.967
CARD11	IKBKG	9606.ENSP00000380150	9606.ENSP00000483825	0	0	0	0	0.046	0.86	0.9	0.588	0.993
CCK	NTS	9606.ENSP00000379472	9606.ENSP00000256010	0	0	0	0	0.062	0	0.9	0.86	0.985
CCK	PIK3R3	9606.ENSP00000379472	9606.ENSP00000262741	0	0	0	0	0	0	0.9	0	0.9
CCK	KNG1	9606.ENSP00000379472	9606.ENSP00000265023	0	0	0	0	0	0	0.9	0.517	0.949
CCK	GAL	9606.ENSP00000379472	9606.ENSP00000265643	0	0	0	0	0	0	0	0.738	0.738
CCK	PTGER1	9606.ENSP00000379472	9606.ENSP00000292513	0	0	0	0	0	0	0.9	0.074	0.903
CCK	PROK2	9606.ENSP00000379472	9606.ENSP00000295619	0	0	0	0	0	0	0.9	0.12	0.908
CCK	F2RL1	9606.ENSP00000379472	9606.ENSP00000296677	0	0	0	0	0	0	0.9	0.077	0.903
CCK	OXTR	9606.ENSP00000379472	9606.ENSP00000324270	0	0	0	0	0	0	0.9	0.526	0.95
CCK	GIP	9606.ENSP00000379472	9606.ENSP00000350005	0	0	0	0	0	0	0	0.739	0.739
CCK	VIP	9606.ENSP00000379472	9606.ENSP00000356213	0	0	0	0	0.231	0	0	0.859	0.887
CCK	XCL1	9606.ENSP00000379472	9606.ENSP00000356792	0	0	0	0	0	0	0.9	0	0.9
CCK	PTGFR	9606.ENSP00000379472	9606.ENSP00000359793	0	0	0	0	0	0	0.9	0.043	0.9
CCK	EDN2	9606.ENSP00000379472	9606.ENSP00000361668	0	0	0	0	0	0	0.9	0.086	0.904
CCK	EDN1	9606.ENSP00000379472	9606.ENSP00000368683	0	0	0	0	0	0	0.9	0.244	0.921
CCK	SAA1	9606.ENSP00000379472	9606.ENSP00000384906	0	0	0	0	0	0	0.9	0	0.9
CCK	CYSLTR1	9606.ENSP00000379472	9606.ENSP00000478492	0	0	0	0	0	0	0.9	0.041	0.9
CCL2	TNFRSF1	9606.ENSP00000225831	9606.ENSP00000162749	0	0	0	0	0.086	0	0	0.769	0.78
CCL2	IL1A	9606.ENSP00000225831	9606.ENSP00000263339	0	0	0	0	0.091	0	0	0.684	0.7
CCL2	LIF	9606.ENSP00000225831	9606.ENSP00000249075	0	0	0	0	0.098	0	0	0.681	0.7
CCL2	TLR3	9606.ENSP00000225831	9606.ENSP00000296795	0	0	0	0	0.055	0	0	0.703	0.708
CCL2	CXCL6	9606.ENSP00000225831	9606.ENSP00000226317	0	0	0	0	0.201	0	0	0.665	0.721
CCL2	OLR1	9606.ENSP00000225831	9606.ENSP00000309124	0	0	0	0	0.084	0	0	0.718	0.73
CCL2	CXCL3	9606.ENSP00000225831	9606.ENSP00000296026	0	0	0	0	0.165	0	0	0.709	0.747
CCL2	IL18	9606.ENSP00000225831	9606.ENSP00000280357	0	0	0	0	0	0	0	0.749	0.749
CCL2	SOCS3	9606.ENSP00000225831	9606.ENSP00000330341	0	0	0	0	0.126	0	0	0.734	0.758
CCL2	IL15	9606.ENSP00000225831	9606.ENSP00000296545	0	0	0	0	0.129	0	0	0.737	0.761
CCL2	IL33	9606.ENSP00000225831	9606.ENSP00000370842	0	0	0	0	0.088	0	0	0.771	0.784

CCL2	CCL20	9606.ENSP00000225831	9606.ENSP00000351671	0	0	0	0	0.107	0	0	0.78	0.795
CCL2	SPP1	9606.ENSP00000225831	9606.ENSP00000378517	0	0	0	0	0.118	0	0	0.792	0.809
CCL2	TLR4	9606.ENSP00000225831	9606.ENSP00000363089	0	0	0	0	0.096	0	0	0.8	0.812
CCL2	CXCL5	9606.ENSP00000225831	9606.ENSP00000296027	0	0	0	0	0.184	0	0	0.782	0.815
CCL2	CTGF	9606.ENSP00000225831	9606.ENSP00000356954	0	0	0	0	0.104	0	0	0.811	0.824
CCL2	CSF1	9606.ENSP00000225831	9606.ENSP00000327513	0	0	0	0	0.119	0	0	0.83	0.844
CCL2	TLR2	9606.ENSP00000225831	9606.ENSP00000260010	0	0	0	0	0.085	0	0	0.836	0.844
CCL2	PTGS2	9606.ENSP00000225831	9606.ENSP00000356438	0	0	0	0	0.117	0	0	0.847	0.859
CCL2	CCR7	9606.ENSP00000225831	9606.ENSP00000246657	0	0	0	0	0.061	0	0.6	0.662	0.862
CCL2	CXCL1	9606.ENSP00000225831	9606.ENSP00000379110	0	0	0	0	0.21	0	0	0.881	0.902
CCL2	CXCL2	9606.ENSP00000225831	9606.ENSP00000427279	0	0	0	0	0.193	0	0	0.885	0.903
CCL2	ICAM1	9606.ENSP00000225831	9606.ENSP00000264832	0	0	0	0	0.149	0	0	0.919	0.929
CCL2	VCAM1	9606.ENSP00000225831	9606.ENSP00000294728	0	0	0	0	0.206	0	0	0.927	0.94
CCL2	IL1B	9606.ENSP00000225831	9606.ENSP00000263341	0	0	0	0	0.126	0	0	0.945	0.95
CCL2	CXCL8	9606.ENSP00000225831	9606.ENSP00000306512	0	0	0	0	0.278	0.667	0	0.925	0.98
CCL2	IL6	9606.ENSP00000225831	9606.ENSP00000385675	0	0	0	0	0.267	0	0	0.977	0.982
CCL20	CXCL6	9606.ENSP00000351671	9606.ENSP00000226317	0	0	0	0	0.12	0	0.9	0.617	0.963
CCL20	OPRD1	9606.ENSP00000351671	9606.ENSP00000234961	0	0	0	0	0	0	0.9	0	0.9
CCL20	CCR7	9606.ENSP00000351671	9606.ENSP00000246657	0	0	0	0	0.065	0	0.9	0.673	0.966
CCL20	TLR2	9606.ENSP00000351671	9606.ENSP00000260010	0	0	0	0	0.063	0	0	0.724	0.731
CCL20	IL1A	9606.ENSP00000351671	9606.ENSP00000263339	0	0	0	0	0.365	0	0	0.578	0.721
CCL20	IL1B	9606.ENSP00000351671	9606.ENSP00000263341	0	0	0	0	0.603	0	0	0.79	0.913
CCL20	KNG1	9606.ENSP00000351671	9606.ENSP00000265023	0	0	0	0	0	0	0.9	0.081	0.904
CCL20	GAL	9606.ENSP00000351671	9606.ENSP00000265643	0	0	0	0	0	0	0.9	0	0.9
CCL20	CXCL3	9606.ENSP00000351671	9606.ENSP00000296026	0	0	0	0	0.308	0	0.9	0.706	0.977
CCL20	CXCL5	9606.ENSP00000351671	9606.ENSP00000296027	0	0	0	0	0.138	0.274	0.9	0.722	0.98
CCL20	S1PR1	9606.ENSP00000351671	9606.ENSP00000305416	0	0	0	0	0	0	0.9	0.243	0.921
CCL20	CXCL8	9606.ENSP00000351671	9606.ENSP00000306512	0	0	0	0	0.552	0	0.9	0.758	0.988
CCL20	SSTR2	9606.ENSP00000351671	9606.ENSP00000350198	0	0	0	0	0	0	0.9	0	0.9
CCL20	IL6	9606.ENSP00000351671	9606.ENSP00000385675	0	0	0	0	0.245	0	0	0.839	0.873
CCL20	CORT	9606.ENSP00000351671	9606.ENSP00000366248	0	0	0	0	0	0	0.9	0	0.9
CCL20	S1PR2	9606.ENSP00000351671	9606.ENSP00000466933	0	0	0	0	0	0	0.9	0.082	0.904
CCL20	SAA1	9606.ENSP00000351671	9606.ENSP00000384906	0	0	0	0	0.107	0	0.9	0.303	0.932
CCL20	CCL5	9606.ENSP00000351671	9606.ENSP00000474412	0	0	0	0	0.133	0.219	0.9	0.738	0.979
CCL20	CXCL2	9606.ENSP00000351671	9606.ENSP00000427279	0	0	0	0	0.248	0	0.9	0.749	0.979
CCL20	CXCL1	9606.ENSP00000351671	9606.ENSP00000379110	0	0	0	0	0.553	0	0.9	0.759	0.988
CCL5	CXCL6	9606.ENSP00000474412	9606.ENSP00000226317	0	0	0	0	0.063	0.215	0.9	0.589	0.965
CCL5	OPRD1	9606.ENSP00000474412	9606.ENSP00000234961	0	0	0	0	0	0.05	0.9	0.205	0.917
CCL5	CCR7	9606.ENSP00000474412	9606.ENSP00000246657	0	0	0	0	0.184	0	0.9	0.678	0.971
CCL5	TLR2	9606.ENSP00000474412	9606.ENSP00000260010	0	0	0	0	0.137	0	0	0.7	0.73
CCL5	IL1B	9606.ENSP00000474412	9606.ENSP00000263341	0	0	0	0	0.13	0	0	0.867	0.88
CCL5	ICAM1	9606.ENSP00000474412	9606.ENSP00000264832	0	0	0	0	0.08	0	0	0.84	0.847
CCL5	KNG1	9606.ENSP00000474412	9606.ENSP00000265023	0	0	0	0	0	0	0.9	0.284	0.925
CCL5	GAL	9606.ENSP00000474412	9606.ENSP00000265643	0	0	0	0	0	0	0.9	0.097	0.905
CCL5	IL18	9606.ENSP00000474412	9606.ENSP00000280357	0	0	0	0	0.061	0	0	0.711	0.717
CCL5	CXCL3	9606.ENSP00000474412	9606.ENSP00000296026	0	0	0	0	0.063	0.069	0.9	0.669	0.967
CCL5	CXCL5	9606.ENSP00000474412	9606.ENSP00000296027	0	0	0	0	0.063	0.069	0.9	0.753	0.975
CCL5	IL15	9606.ENSP00000474412	9606.ENSP00000296545	0	0	0	0	0.086	0	0	0.774	0.785
CCL5	TLR3	9606.ENSP00000474412	9606.ENSP00000296795	0	0	0	0	0	0	0	0.705	0.705
CCL5	S1PR1	9606.ENSP00000474412	9606.ENSP00000305416	0	0	0	0	0	0	0.9	0.256	0.922
CCL5	CXCL8	9606.ENSP00000474412	9606.ENSP00000306512	0	0	0	0	0.064	0.397	0.9	0.904	0.993
CCL5	SSTR2	9606.ENSP00000474412	9606.ENSP00000350198	0	0	0	0	0	0.05	0.9	0.044	0.901
CCL5	SDC4	9606.ENSP00000474412	9606.ENSP00000361818	0	0	0	0	0.061	0.379	0.9	0.211	0.947
CCL5	TLR4	9606.ENSP00000474412	9606.ENSP00000363089	0	0	0	0	0.061	0	0	0.828	0.832
CCL5	CORT	9606.ENSP00000474412	9606.ENSP00000366248	0	0	0	0	0	0	0.9	0.23	0.919
CCL5	SDC1	9606.ENSP00000474412	9606.ENSP00000370542	0	0	0	0	0.06	0.379	0.9	0.286	0.952
CCL5	CXCL1	9606.ENSP00000474412	9606.ENSP00000379110	0	0	0	0	0.063	0.069	0.9	0.867	0.986
CCL5	SAA1	9606.ENSP00000474412	9606.ENSP00000384906	0	0	0	0	0.058	0	0.9	0.366	0.935
CCL5	IL6	9606.ENSP00000474412	9606.ENSP00000385675	0	0	0	0	0.064	0	0	0.932	0.934
CCL5	CXCL2	9606.ENSP00000474412	9606.ENSP00000427279	0	0	0	0	0.063	0.228	0.9	0.777	0.981
CCL5	S1PR2	9606.ENSP00000474412	9606.ENSP00000466933	0	0	0	0	0	0	0.9	0.211	0.917
CCR7	CXCL6	9606.ENSP00000246657	9606.ENSP00000226317	0	0	0	0	0.049	0	0.9	0.226	0.919
CCR7	OPRD1	9606.ENSP00000246657	9606.ENSP00000234961	0	0	0	0.657	0	0	0.9	0.056	0.9
CCR7	IL18	9606.ENSP00000246657	9606.ENSP00000280357	0	0	0	0	0.061	0	0	0.695	0.701
CCR7	XCL1	9606.ENSP00000246657	9606.ENSP00000356792	0	0	0	0	0.096	0	0.6	0.493	0.8
CCR7	IL6	9606.ENSP00000246657	9606.ENSP00000385675	0	0	0	0	0.071	0	0	0.796	0.803
CCR7	IL7R	9606.ENSP00000246657	9606.ENSP00000306157	0	0	0	0	0.346	0	0	0.763	0.838
CCR7	SSTR2	9606.ENSP00000246657	9606.ENSP00000350198	0	0	0	0.713	0	0	0.9	0.048	0.9
CCR7	CORT	9606.ENSP00000246657	9606.ENSP00000366248	0	0	0	0	0	0	0.9	0	0.9
CCR7	KNG1	9606.ENSP00000246657	9606.ENSP00000265023	0	0	0	0	0	0	0.9	0.053	0.901
CCR7	GAL	9606.ENSP00000246657	9606.ENSP00000265643	0	0	0	0	0	0	0.9	0.059	0.901
CCR7	SAA1	9606.ENSP00000246657	9606.ENSP00000384906	0	0	0	0	0	0	0.9	0.076	0.903

CCR7	S1PR2	9606.ENSP00000246657	9606.ENSP00000466933	0	0	0	0	0	0	0.9	0.307	0.927
CCR7	CXCL3	9606.ENSP00000246657	9606.ENSP00000296026	0	0	0	0	0	0	0.9	0.399	0.937
CCR7	CXCL5	9606.ENSP00000246657	9606.ENSP00000296027	0	0	0	0	0.049	0	0.9	0.41	0.938
CCR7	CXCL2	9606.ENSP00000246657	9606.ENSP00000427279	0	0	0	0	0	0	0.9	0.441	0.941
CCR7	CXCL1	9606.ENSP00000246657	9606.ENSP00000379110	0	0	0	0	0	0	0.9	0.51	0.948
CCR7	CXCL8	9606.ENSP00000246657	9606.ENSP00000306512	0	0	0	0	0.075	0	0.9	0.585	0.958
CCR7	S1PR1	9606.ENSP00000246657	9606.ENSP00000305416	0	0	0	0	0.089	0	0.9	0.672	0.967
CD14	TLR2	9606.ENSP00000304236	9606.ENSP00000260010	0	0	0	0	0.612	0.393	0.9	0.691	0.991
CD14	IKBKG	9606.ENSP00000304236	9606.ENSP00000483825	0	0	0	0	0.065	0	0.9	0.059	0.904
CD14	IKBKE	9606.ENSP00000304236	9606.ENSP00000464030	0	0	0	0	0.064	0	0.9	0.1	0.908
CD14	IRF7	9606.ENSP00000304236	9606.ENSP00000380697	0	0	0	0	0.119	0	0.9	0.149	0.918
CD14	TLR1	9606.ENSP00000304236	9606.ENSP00000354932	0	0	0	0	0.244	0.064	0.9	0.455	0.956
CD14	ITGB2	9606.ENSP00000304236	9606.ENSP00000380948	0	0	0	0	0.623	0.379	0.9	0.232	0.979
CD14	TLR4	9606.ENSP00000304236	9606.ENSP00000363089	0	0	0	0	0.264	0.472	0.9	0.758	0.989
CDH1	PROC	9606.ENSP00000261769	9606.ENSP00000234071	0	0	0	0	0	0	0.9	0.173	0.913
CDH1	HNF4A	9606.ENSP00000261769	9606.ENSP00000312987	0	0	0	0	0.107	0	0	0.701	0.721
CDH1	CTGF	9606.ENSP00000261769	9606.ENSP00000356954	0	0	0	0	0	0	0	0.737	0.738
CDH1	PTGS2	9606.ENSP00000261769	9606.ENSP00000356438	0	0	0	0	0	0.074	0	0.735	0.744
CDH1	WNT5A	9606.ENSP00000261769	9606.ENSP00000264634	0	0	0	0	0	0.09	0	0.745	0.759
CDH1	CXCL8	9606.ENSP00000261769	9606.ENSP00000306512	0	0	0	0	0	0	0	0.763	0.763
CDH1	IL6	9606.ENSP00000261769	9606.ENSP00000385675	0	0	0	0	0	0	0	0.788	0.788
CDH1	FGF2	9606.ENSP00000261769	9606.ENSP00000264498	0	0	0	0	0	0	0	0.795	0.795
CDH1	PRKCA	9606.ENSP00000261769	9606.ENSP00000408695	0	0	0	0	0	0	0.8	0.296	0.853
CDH1	GDNF	9606.ENSP00000261769	9606.ENSP00000409007	0	0	0	0	0	0	0.9	0.292	0.926
CDH1	MAPK3	9606.ENSP00000261769	9606.ENSP00000263025	0	0	0	0	0	0	0.9	0.678	0.966
CDH1	IGF1R	9606.ENSP00000261769	9606.ENSP00000268035	0	0	0	0	0	0.056	0.9	0.746	0.974
CDH1	MET	9606.ENSP00000261769	9606.ENSP00000317272	0	0	0	0	0.062	0.388	0.9	0.81	0.987
CDH1	SRC	9606.ENSP00000261769	9606.ENSP00000362680	0	0	0	0	0.041	0.388	0.9	0.858	0.99
CDH1	EGFR	9606.ENSP00000261769	9606.ENSP00000275493	0	0	0	0	0.062	0.513	0.9	0.917	0.995
CDH1	FYN	9606.ENSP00000261769	9606.ENSP00000346671	0	0	0	0	0	0.993	0.9	0.637	0.999
CHGB	PROC	9606.ENSP00000368244	9606.ENSP00000234071	0	0	0	0	0	0	0.9	0	0.9
CHGB	FGF23	9606.ENSP00000368244	9606.ENSP00000237837	0	0	0	0	0	0	0.9	0	0.9
CHGB	KNG1	9606.ENSP00000368244	9606.ENSP00000265023	0	0	0	0	0	0	0.9	0.14	0.91
CHGB	SCG2	9606.ENSP00000368244	9606.ENSP00000304133	0	0	0	0	0.296	0	0.9	0.898	0.992
CHGB	FGA	9606.ENSP00000368244	9606.ENSP00000306361	0	0	0	0	0	0	0.9	0.151	0.911
CHGB	SDC2	9606.ENSP00000368244	9606.ENSP00000307046	0	0	0	0	0	0	0.9	0	0.9
CHGB	CSF1	9606.ENSP00000368244	9606.ENSP00000327513	0	0	0	0	0	0	0.9	0	0.9
CHGB	IL6	9606.ENSP00000368244	9606.ENSP00000385675	0	0	0	0	0	0	0.9	0.075	0.903
CHGB	SPP1	9606.ENSP00000368244	9606.ENSP00000378517	0	0	0	0	0	0	0.9	0.128	0.909
CLCF1	JAK1	9606.ENSP00000309338	9606.ENSP00000343204	0	0	0	0	0	0.078	0.9	0.287	0.928
CLCF1	IL6ST	9606.ENSP00000309338	9606.ENSP00000370698	0	0	0	0	0.06	0.17	0.9	0.413	0.948
CLCF1	CNTFR	9606.ENSP00000309338	9606.ENSP00000368265	0	0	0	0	0	0.437	0.9	0.639	0.977
CLCF1	CRLF1	9606.ENSP00000309338	9606.ENSP00000376188	0	0	0	0	0.066	0.379	0.9	0.908	0.993
CLEC11A	KITLG	9606.ENSP00000250340	9606.ENSP00000228280	0	0	0	0	0	0.379	0	0.558	0.713
CNTFR	LIF	9606.ENSP00000368265	9606.ENSP00000249075	0	0	0	0	0	0	0.6	0.531	0.804
CNTFR	IL11	9606.ENSP00000368265	9606.ENSP00000264563	0	0	0	0	0	0	0.6	0.441	0.767
CNTFR	CTF1	9606.ENSP00000368265	9606.ENSP00000279804	0	0	0	0	0	0	0.6	0.53	0.804
CNTFR	JAK1	9606.ENSP00000368265	9606.ENSP00000343204	0	0	0	0	0	0	0.9	0.243	0.921
CNTFR	IL6	9606.ENSP00000368265	9606.ENSP00000385675	0	0	0	0	0	0	0.6	0.399	0.749
CNTFR	IL6ST	9606.ENSP00000368265	9606.ENSP00000370698	0	0	0	0.587	0	0.379	0.9	0.506	0.948
CNTFR	CRLF1	9606.ENSP00000368265	9606.ENSP00000376188	0	0	0	0.597	0.062	0.379	0.9	0.662	0.953
CORT	RELB	9606.ENSP00000366248	9606.ENSP00000221452	0	0	0	0	0	0	0	0.746	0.746
CORT	CXCL6	9606.ENSP00000366248	9606.ENSP00000226317	0	0	0	0	0	0	0.9	0	0.9
CORT	OPRD1	9606.ENSP00000366248	9606.ENSP00000234961	0	0	0	0	0	0	0.9	0.27	0.923
CORT	KNG1	9606.ENSP00000366248	9606.ENSP00000265023	0	0	0	0	0	0	0.9	0	0.9
CORT	GAL	9606.ENSP00000366248	9606.ENSP00000265643	0	0	0	0	0	0	0.9	0.25	0.921
CORT	CXCL3	9606.ENSP00000366248	9606.ENSP00000296026	0	0	0	0	0	0	0.9	0	0.9
CORT	CXCL5	9606.ENSP00000366248	9606.ENSP00000296027	0	0	0	0	0	0	0.9	0.046	0.9
CORT	S1PR1	9606.ENSP00000366248	9606.ENSP00000305416	0	0	0	0	0	0	0.9	0	0.9
CORT	CXCL8	9606.ENSP00000366248	9606.ENSP00000306512	0	0	0	0	0	0	0.9	0.218	0.918
CORT	SSTR2	9606.ENSP00000366248	9606.ENSP00000350198	0	0	0	0	0	0.305	0.9	0.59	0.969
CORT	S1PR2	9606.ENSP00000366248	9606.ENSP00000466933	0	0	0	0	0	0	0.9	0	0.9
CORT	CXCL2	9606.ENSP00000366248	9606.ENSP00000427279	0	0	0	0	0	0	0.9	0	0.9
CORT	CXCL1	9606.ENSP00000366248	9606.ENSP00000379110	0	0	0	0	0	0	0.9	0.185	0.915
CORT	SAA1	9606.ENSP00000366248	9606.ENSP00000384906	0	0	0	0	0	0	0.9	0.252	0.922
CRABP2	RXRA	9606.ENSP00000482841	9606.ENSP00000419692	0	0	0	0	0	0.064	0.9	0.432	0.942
CRLF1	JAK1	9606.ENSP00000376188	9606.ENSP00000343204	0	0	0	0	0	0.072	0.9	0.292	0.928
CRLF1	IL6ST	9606.ENSP00000376188	9606.ENSP00000370698	0	0	0	0.667	0	0	0.9	0.106	0.902
CSF1	PROC	9606.ENSP00000327513	9606.ENSP00000234071	0	0	0	0	0	0	0.9	0	0.9
CSF1	FGF23	9606.ENSP00000327513	9606.ENSP00000237837	0	0	0	0	0	0	0.9	0.153	0.911
CSF1	FLT3	9606.ENSP00000327513	9606.ENSP00000241453	0	0	0	0	0	0.176	0.6	0.641	0.871
CSF1	ITGAV	9606.ENSP00000327513	9606.ENSP00000261023	0	0	0	0	0.088	0	0.9	0.328	0.933

CSF1	FLT4	9606.ENSP00000327513	9606.ENSP00000261937	0	0	0	0	0.061	0.176	0.6	0.269	0.743
CSF1	IL1B	9606.ENSP00000327513	9606.ENSP00000263341	0	0	0	0	0.083	0	0	0.787	0.796
CSF1	KNG1	9606.ENSP00000327513	9606.ENSP00000265023	0	0	0	0	0	0	0.9	0.095	0.905
CSF1	EGFR	9606.ENSP00000327513	9606.ENSP00000275493	0	0	0	0	0.076	0	0.6	0.478	0.79
CSF1	FLT1	9606.ENSP00000327513	9606.ENSP00000282397	0	0	0	0	0.061	0.176	0.6	0.448	0.806
CSF1	SCG2	9606.ENSP00000327513	9606.ENSP00000304133	0	0	0	0	0	0	0.9	0	0.9
CSF1	FGA	9606.ENSP00000327513	9606.ENSP00000306361	0	0	0	0	0	0	0.9	0.082	0.904
CSF1	SDC2	9606.ENSP00000327513	9606.ENSP00000307046	0	0	0	0	0.062	0	0.9	0.058	0.903
CSF1	MET	9606.ENSP00000327513	9606.ENSP00000317272	0	0	0	0	0.062	0	0.6	0.267	0.7
CSF1	SPP1	9606.ENSP00000327513	9606.ENSP00000378517	0	0	0	0	0.071	0	0.9	0.511	0.95
CSF1	IL6	9606.ENSP00000327513	9606.ENSP00000385675	0	0	0	0	0.117	0	0.9	0.751	0.976
CSF2RA	PIK3R3	9606.ENSP00000394227	9606.ENSP00000262741	0	0	0	0	0	0	0.9	0	0.9
CSF2RA	IL15	9606.ENSP00000394227	9606.ENSP00000296545	0	0	0	0	0.189	0	0.6	0.201	0.718
CSF2RA	JAK1	9606.ENSP00000394227	9606.ENSP00000343204	0	0	0	0	0	0.05	0.9	0.25	0.922
CSF2RA	SFTPD	9606.ENSP00000394227	9606.ENSP00000361366	0	0	0	0	0.057	0	0.9	0.233	0.921
CSF2RA	SYK	9606.ENSP00000394227	9606.ENSP00000364907	0	0	0	0	0.119	0.05	0.9	0.076	0.912
CSF2RA	IL6	9606.ENSP00000394227	9606.ENSP00000385675	0	0	0	0	0	0	0.6	0.387	0.744
CSF2RA	PTPN6	9606.ENSP00000394227	9606.ENSP00000391592	0	0	0	0	0.097	0	0.9	0	0.905
CSF2RA	SHC1	9606.ENSP00000394227	9606.ENSP00000401303	0	0	0	0	0	0	0.9	0.104	0.906
CSF2RA	INPP5D	9606.ENSP00000394227	9606.ENSP00000405338	0	0	0	0	0.088	0	0.9	0.083	0.909
CSPG5	SDC2	9606.ENSP00000373244	9606.ENSP00000307046	0	0	0	0	0	0	0.9	0.059	0.901
CSPG5	SDC4	9606.ENSP00000373244	9606.ENSP00000361818	0	0	0	0	0	0	0.9	0.299	0.926
CSPG5	SDC1	9606.ENSP00000373244	9606.ENSP00000370542	0	0	0	0	0	0	0.9	0.162	0.912
CTF1	LIF	9606.ENSP00000279804	9606.ENSP00000249075	0	0	0	0	0	0	0.9	0.803	0.979
CTF1	IL11	9606.ENSP00000279804	9606.ENSP00000264563	0	0	0	0	0	0	0	0.755	0.755
CTF1	OSMR	9606.ENSP00000279804	9606.ENSP00000274276	0	0	0	0	0	0.129	0.6	0.577	0.839
CTF1	IL6	9606.ENSP00000279804	9606.ENSP00000385675	0	0	0	0	0	0	0	0.802	0.802
CTF1	SHC1	9606.ENSP00000279804	9606.ENSP00000401303	0	0	0	0	0	0	0.9	0.08	0.904
CTF1	JAK1	9606.ENSP00000279804	9606.ENSP00000343204	0	0	0	0	0	0	0.9	0.428	0.94
CTF1	IL6ST	9606.ENSP00000279804	9606.ENSP00000370698	0	0	0	0	0	0.379	0.9	0.444	0.962
CTGF	TGFB3	9606.ENSP00000356954	9606.ENSP00000238682	0	0	0	0	0.064	0.379	0.9	0.517	0.968
CTGF	THBS1	9606.ENSP00000356954	9606.ENSP00000260356	0	0	0	0	0.19	0	0	0.692	0.74
CTGF	FGF2	9606.ENSP00000356954	9606.ENSP00000264498	0	0	0	0	0.138	0	0	0.78	0.803
CTGF	EGFR	9606.ENSP00000356954	9606.ENSP00000275493	0	0	0	0	0.214	0.294	0	0.718	0.83
CTGF	TGFBR2	9606.ENSP00000356954	9606.ENSP00000351905	0	0	0	0	0.079	0	0.9	0.51	0.95
CTGF	TGFB2	9606.ENSP00000356954	9606.ENSP00000355896	0	0	0	0	0.154	0.379	0	0.59	0.765
CTGF	PTHLH	9606.ENSP00000356954	9606.ENSP00000441765	0	0	0	0	0.088	0	0	0.69	0.706
CTGF	IL6	9606.ENSP00000356954	9606.ENSP00000385675	0	0	0	0	0.094	0	0	0.795	0.807
CTGF	EDN1	9606.ENSP00000356954	9606.ENSP00000368683	0	0	0	0	0.105	0	0	0.852	0.862
CTGF	ITGB2	9606.ENSP00000356954	9606.ENSP00000380948	0	0	0	0	0	0	0.9	0.285	0.925
CTSB	HLA-DQA	9606.ENSP00000345672	9606.ENSP00000339398	0	0	0	0	0	0	0.9	0.231	0.919
CTSB	PLAU	9606.ENSP00000345672	9606.ENSP00000361850	0	0	0	0	0.077	0.379	0	0.553	0.721
CTSB	CTSS	9606.ENSP00000345672	9606.ENSP00000357981	0	0	0.223	0.675	0.103	0	0.8	0.77	0.868
CTSB	HLA-DRB	9606.ENSP00000345672	9606.ENSP00000364114	0	0	0	0	0.062	0	0.9	0.087	0.906
CTSB	HLA-DQB	9606.ENSP00000345672	9606.ENSP00000364080	0	0	0	0	0.062	0	0.9	0.104	0.908
CTSB	HLA-DPA	9606.ENSP00000345672	9606.ENSP00000393566	0	0	0	0	0	0	0.9	0.129	0.909
CTSB	HLA-DPB	9606.ENSP00000345672	9606.ENSP00000408146	0	0	0	0	0.062	0	0.9	0.126	0.91
CTSB	HLA-DRB	9606.ENSP00000345672	9606.ENSP00000353099	0	0	0	0	0.062	0	0.9	0.138	0.912
CTSS	HLA-DQA	9606.ENSP00000357981	9606.ENSP00000339398	0	0	0	0	0.202	0	0.9	0.195	0.93
CTSS	HLA-DRB	9606.ENSP00000357981	9606.ENSP00000353099	0	0	0	0	0.264	0	0.9	0.198	0.935
CTSS	ITGB2	9606.ENSP00000357981	9606.ENSP00000380948	0	0	0	0	0.747	0	0	0.399	0.841
CTSS	PTPN6	9606.ENSP00000357981	9606.ENSP00000391592	0	0	0	0	0.156	0	0.9	0.089	0.916
CTSS	HLA-DRB	9606.ENSP00000357981	9606.ENSP00000364114	0	0	0	0	0.166	0	0.9	0.13	0.921
CTSS	CXCL1	9606.ENSP00000357981	9606.ENSP00000379110	0	0	0	0	0.095	0	0.9	0.237	0.924
CTSS	HLA-DPB	9606.ENSP00000357981	9606.ENSP00000408146	0	0	0	0	0.17	0	0.9	0.178	0.925
CTSS	HLA-DPA	9606.ENSP00000357981	9606.ENSP00000393566	0	0	0	0	0.248	0	0.9	0.106	0.926
CTSS	HLA-DQB	9606.ENSP00000357981	9606.ENSP00000364080	0	0	0	0	0.233	0	0.9	0.21	0.934
CXCL1	CXCL6	9606.ENSP00000379110	9606.ENSP00000226317	0	0	0	0.893	0.647	0.667	0.9	0.778	0.988
CXCL1	OPRD1	9606.ENSP00000379110	9606.ENSP00000234961	0	0	0	0	0	0	0.9	0.07	0.903
CXCL1	TLR2	9606.ENSP00000379110	9606.ENSP00000260010	0	0	0	0	0.133	0	0	0.796	0.816
CXCL1	IL1A	9606.ENSP00000379110	9606.ENSP00000263339	0	0	0	0	0.295	0	0	0.652	0.744
CXCL1	IL1B	9606.ENSP00000379110	9606.ENSP00000263341	0	0	0	0	0.601	0	0	0.888	0.953
CXCL1	ICAM1	9606.ENSP00000379110	9606.ENSP00000264832	0	0	0	0	0.223	0	0	0.7	0.757
CXCL1	KNG1	9606.ENSP00000379110	9606.ENSP00000265023	0	0	0	0	0	0	0.9	0.212	0.917
CXCL1	GAL	9606.ENSP00000379110	9606.ENSP00000265643	0	0	0	0	0	0	0.9	0.156	0.912
CXCL1	IL18	9606.ENSP00000379110	9606.ENSP00000280357	0	0	0	0	0.083	0	0	0.747	0.758
CXCL1	CXCL3	9606.ENSP00000379110	9606.ENSP00000296026	0	0	0	0.979	0.674	0	0.9	0.792	0.966
CXCL1	CXCL5	9606.ENSP00000379110	9606.ENSP00000296027	0	0	0	0.907	0.397	0.667	0.9	0.887	0.979
CXCL1	S1PR1	9606.ENSP00000379110	9606.ENSP00000305416	0	0	0	0	0	0	0.9	0.146	0.91
CXCL1	CXCL8	9606.ENSP00000379110	9606.ENSP00000306512	0	0	0	0.878	0.842	0	0.9	0.918	0.985
CXCL1	SLPI	9606.ENSP00000379110	9606.ENSP00000342082	0	0	0	0	0.108	0	0.9	0.33	0.935
CXCL1	SSTR2	9606.ENSP00000379110	9606.ENSP00000350198	0	0	0	0	0	0	0.9	0.05	0.9

CXCL1	PTGS2	9606.ENSP00000379110	9606.ENSP00000356438	0	0	0	0	0.549	0	0	0.753	0.884
CXCL1	LCN2	9606.ENSP00000379110	9606.ENSP00000362108	0	0	0	0	0.146	0	0.9	0.562	0.959
CXCL1	TLR4	9606.ENSP00000379110	9606.ENSP00000363089	0	0	0	0	0.107	0	0	0.742	0.76
CXCL1	PTPN6	9606.ENSP00000379110	9606.ENSP00000391592	0	0	0	0	0	0	0.9	0.069	0.902
CXCL1	S1PR2	9606.ENSP00000379110	9606.ENSP00000466933	0	0	0	0	0.06	0	0.9	0.1	0.908
CXCL1	ELANE	9606.ENSP00000379110	9606.ENSP00000466090	0	0	0	0	0	0	0.9	0.45	0.942
CXCL1	SAA1	9606.ENSP00000379110	9606.ENSP00000384906	0	0	0	0	0.118	0	0.9	0.416	0.944
CXCL1	IL6	9606.ENSP00000379110	9606.ENSP00000385675	0	0	0	0	0.563	0	0	0.942	0.974
CXCL1	CXCL2	9606.ENSP00000379110	9606.ENSP00000427279	0	0	0	0.979	0.809	0	0.9	0.934	0.98
CXCL2	CXCL6	9606.ENSP00000427279	9606.ENSP00000226317	0	0	0	0.875	0.316	0.55	0.9	0.797	0.969
CXCL2	OPRD1	9606.ENSP00000427279	9606.ENSP00000234961	0	0	0	0	0	0	0.9	0.096	0.905
CXCL2	IL1B	9606.ENSP00000427279	9606.ENSP00000263341	0	0	0	0	0.592	0	0	0.781	0.906
CXCL2	KNG1	9606.ENSP00000427279	9606.ENSP00000265023	0	0	0	0	0	0	0.9	0.113	0.907
CXCL2	GAL	9606.ENSP00000427279	9606.ENSP00000265643	0	0	0	0	0	0	0.9	0.106	0.906
CXCL2	CXCL3	9606.ENSP00000427279	9606.ENSP00000296026	0	0	0	0.979	0.705	0	0.9	0.834	0.969
CXCL2	CXCL5	9606.ENSP00000427279	9606.ENSP00000296027	0	0	0	0.898	0.319	0.473	0.9	0.833	0.964
CXCL2	S1PR1	9606.ENSP00000427279	9606.ENSP00000305416	0	0	0	0	0	0	0.9	0.093	0.905
CXCL2	CXCL8	9606.ENSP00000427279	9606.ENSP00000306512	0	0	0	0.874	0.824	0	0.9	0.767	0.983
CXCL2	SSTR2	9606.ENSP00000427279	9606.ENSP00000350198	0	0	0	0	0	0	0.9	0.05	0.9
CXCL2	PTGS2	9606.ENSP00000427279	9606.ENSP00000356438	0	0	0	0	0.538	0	0	0.588	0.802
CXCL2	TLR4	9606.ENSP00000427279	9606.ENSP00000363089	0	0	0	0	0.096	0	0	0.723	0.739
CXCL2	SAA1	9606.ENSP00000427279	9606.ENSP00000384906	0	0	0	0	0.108	0	0.9	0.291	0.931
CXCL2	IL6	9606.ENSP00000427279	9606.ENSP00000385675	0	0	0	0	0.631	0	0	0.825	0.932
CXCL2	S1PR2	9606.ENSP00000427279	9606.ENSP00000466933	0	0	0	0	0.06	0	0.9	0.116	0.909
CXCL3	CXCL6	9606.ENSP00000296026	9606.ENSP00000226317	0	0	0	0.884	0.329	0.234	0.9	0.838	0.949
CXCL3	OPRD1	9606.ENSP00000296026	9606.ENSP00000234961	0	0	0	0	0	0	0.9	0	0.9
CXCL3	IL1B	9606.ENSP00000296026	9606.ENSP00000263341	0	0	0	0	0.354	0	0	0.69	0.791
CXCL3	KNG1	9606.ENSP00000296026	9606.ENSP00000265023	0	0	0	0	0	0	0.9	0.047	0.9
CXCL3	GAL	9606.ENSP00000296026	9606.ENSP00000265643	0	0	0	0	0	0	0.9	0.062	0.902
CXCL3	IL6	9606.ENSP00000296026	9606.ENSP00000385675	0	0	0	0	0.276	0	0	0.725	0.793
CXCL3	SSTR2	9606.ENSP00000296026	9606.ENSP00000350198	0	0	0	0	0	0	0.9	0.05	0.9
CXCL3	S1PR2	9606.ENSP00000296026	9606.ENSP00000466933	0	0	0	0	0.06	0	0.9	0	0.901
CXCL3	S1PR1	9606.ENSP00000296026	9606.ENSP00000305416	0	0	0	0	0	0	0.9	0.073	0.903
CXCL3	SAA1	9606.ENSP00000296026	9606.ENSP00000384906	0	0	0	0	0.087	0	0.9	0.259	0.926
CXCL3	CXCL8	9606.ENSP00000296026	9606.ENSP00000306512	0	0	0	0.865	0.626	0	0.9	0.708	0.964
CXCL3	CXCL5	9606.ENSP00000296026	9606.ENSP00000296027	0	0	0	0.902	0.409	0.667	0.9	0.838	0.98
CXCL5	CXCL6	9606.ENSP00000296027	9606.ENSP00000226317	0	0	0	0.974	0.233	0	0.9	0.8	0.921
CXCL5	OPRD1	9606.ENSP00000296027	9606.ENSP00000234961	0	0	0	0	0	0	0.9	0	0.9
CXCL5	IL1B	9606.ENSP00000296027	9606.ENSP00000263341	0	0	0	0	0.19	0	0	0.84	0.865
CXCL5	KNG1	9606.ENSP00000296027	9606.ENSP00000265023	0	0	0	0	0	0	0.9	0.164	0.912
CXCL5	GAL	9606.ENSP00000296027	9606.ENSP00000265643	0	0	0	0	0	0	0.9	0.041	0.9
CXCL5	IL6	9606.ENSP00000296027	9606.ENSP00000385675	0	0	0	0	0.227	0	0	0.798	0.837
CXCL5	SSTR2	9606.ENSP00000296027	9606.ENSP00000350198	0	0	0	0	0	0	0.9	0	0.9
CXCL5	S1PR2	9606.ENSP00000296027	9606.ENSP00000466933	0	0	0	0	0	0	0.9	0.049	0.9
CXCL5	S1PR1	9606.ENSP00000296027	9606.ENSP00000305416	0	0	0	0	0	0	0.9	0.105	0.906
CXCL5	SAA1	9606.ENSP00000296027	9606.ENSP00000384906	0	0	0	0	0.064	0	0.9	0.358	0.934
CXCL5	CXCL8	9606.ENSP00000296027	9606.ENSP00000306512	0	0	0	0.85	0.369	0	0.9	0.869	0.942
CXCL6	IL6	9606.ENSP00000226317	9606.ENSP00000385675	0	0	0	0	0.193	0	0	0.7	0.747
CXCL6	SSTR2	9606.ENSP00000226317	9606.ENSP00000350198	0	0	0	0	0	0	0.9	0	0.9
CXCL6	S1PR2	9606.ENSP00000226317	9606.ENSP00000466933	0	0	0	0	0	0	0.9	0	0.9
CXCL6	KNG1	9606.ENSP00000226317	9606.ENSP00000265023	0	0	0	0	0	0	0.9	0	0.9
CXCL6	S1PR1	9606.ENSP00000226317	9606.ENSP00000305416	0	0	0	0	0	0	0.9	0	0.9
CXCL6	GAL	9606.ENSP00000226317	9606.ENSP00000265643	0	0	0	0	0	0	0.9	0	0.9
CXCL6	OPRD1	9606.ENSP00000226317	9606.ENSP00000234961	0	0	0	0	0	0	0.9	0	0.9
CXCL6	SAA1	9606.ENSP00000226317	9606.ENSP00000384906	0	0	0	0	0.092	0	0.9	0.225	0.923
CXCL6	CXCL8	9606.ENSP00000226317	9606.ENSP00000306512	0	0	0	0.83	0.378	0	0.9	0.704	0.942
CXCL8	OPRD1	9606.ENSP00000306512	9606.ENSP00000234961	0	0	0	0	0	0	0.9	0.25	0.921
CXCL8	TLR2	9606.ENSP00000306512	9606.ENSP00000260010	0	0	0	0	0.157	0	0	0.909	0.92
CXCL8	MAPK3	9606.ENSP00000306512	9606.ENSP00000263025	0	0	0	0	0	0	0	0.817	0.817
CXCL8	IL1A	9606.ENSP00000306512	9606.ENSP00000263339	0	0	0	0	0.232	0	0	0.869	0.895
CXCL8	IL1B	9606.ENSP00000306512	9606.ENSP00000263341	0	0	0	0	0.676	0	0	0.977	0.992
CXCL8	FGF2	9606.ENSP00000306512	9606.ENSP00000264498	0	0	0	0	0	0	0	0.745	0.745
CXCL8	ICAM1	9606.ENSP00000306512	9606.ENSP00000264832	0	0	0	0	0.214	0	0	0.912	0.928
CXCL8	KNG1	9606.ENSP00000306512	9606.ENSP00000265023	0	0	0	0	0	0	0.9	0.53	0.95
CXCL8	GAL	9606.ENSP00000306512	9606.ENSP00000265643	0	0	0	0	0	0	0.9	0.174	0.913
CXCL8	EGFR	9606.ENSP00000306512	9606.ENSP00000275493	0	0	0	0	0	0	0	0.817	0.817
CXCL8	IL18	9606.ENSP00000306512	9606.ENSP00000280357	0	0	0	0	0.089	0	0	0.831	0.84
CXCL8	VCAM1	9606.ENSP00000306512	9606.ENSP00000294728	0	0	0	0	0.061	0	0	0.793	0.798
CXCL8	IL15	9606.ENSP00000306512	9606.ENSP00000296545	0	0	0	0	0.056	0	0	0.799	0.802
CXCL8	TLR3	9606.ENSP00000306512	9606.ENSP00000296795	0	0	0	0	0.055	0	0	0.858	0.86
CXCL8	S1PR1	9606.ENSP00000306512	9606.ENSP00000305416	0	0	0	0	0	0	0.9	0.266	0.923
CXCL8	MUC5AC	9606.ENSP00000306512	9606.ENSP00000485659	0	0	0	0	0	0	0	0.724	0.724

CXCL8	IL32	9606.ENSP00000306512	9606.ENSP00000432218	0	0	0	0	0.076	0	0	0.738	0.748
CXCL8	SRC	9606.ENSP00000306512	9606.ENSP00000362680	0	0	0	0	0	0	0	0.753	0.753
CXCL8	TNFAIP3	9606.ENSP00000306512	9606.ENSP00000481570	0	0	0	0	0.537	0	0	0.496	0.757
CXCL8	TLR1	9606.ENSP00000306512	9606.ENSP00000354932	0	0	0	0	0.139	0	0	0.733	0.76
CXCL8	ELANE	9606.ENSP00000306512	9606.ENSP00000466090	0	0	0	0	0.065	0	0	0.836	0.84
CXCL8	IL33	9606.ENSP00000306512	9606.ENSP00000370842	0	0	0	0	0.061	0	0	0.841	0.845
CXCL8	SSTR2	9606.ENSP00000306512	9606.ENSP00000350198	0	0	0	0	0	0	0.9	0.087	0.904
CXCL8	SDC2	9606.ENSP00000306512	9606.ENSP00000307046	0	0	0	0	0	0	0.9	0.217	0.918
CXCL8	S1PR2	9606.ENSP00000306512	9606.ENSP00000466933	0	0	0	0	0	0	0.9	0.22	0.918
CXCL8	TLR4	9606.ENSP00000306512	9606.ENSP00000363089	0	0	0	0	0.116	0	0	0.924	0.93
CXCL8	HCK	9606.ENSP00000306512	9606.ENSP00000444986	0	0	0	0	0.117	0	0.9	0.345	0.937
CXCL8	SAA1	9606.ENSP00000306512	9606.ENSP00000384906	0	0	0	0	0.091	0	0.9	0.548	0.955
CXCL8	PTGS2	9606.ENSP00000306512	9606.ENSP00000356438	0	0	0	0	0.753	0	0	0.839	0.958
CXCL8	IL6	9606.ENSP00000306512	9606.ENSP00000385675	0	0	0	0	0.581	0	0	0.966	0.985
CYSLTR1	NTS	9606.ENSP00000478492	9606.ENSP00000256010	0	0	0	0	0	0	0.9	0.096	0.905
CYSLTR1	PIK3R3	9606.ENSP00000478492	9606.ENSP00000262741	0	0	0	0	0	0	0.9	0	0.9
CYSLTR1	KNG1	9606.ENSP00000478492	9606.ENSP00000265023	0	0	0	0	0	0	0.9	0.217	0.918
CYSLTR1	PTGER1	9606.ENSP00000478492	9606.ENSP00000292513	0	0	0	0	0	0	0.9	0.442	0.941
CYSLTR1	PROK2	9606.ENSP00000478492	9606.ENSP00000295619	0	0	0	0	0	0	0.9	0.047	0.9
CYSLTR1	F2RL1	9606.ENSP00000478492	9606.ENSP00000296677	0	0	0	0.73	0	0	0.9	0.115	0.902
CYSLTR1	OXTR	9606.ENSP00000478492	9606.ENSP00000324270	0	0	0	0.587	0	0	0.9	0	0.9
CYSLTR1	XCL1	9606.ENSP00000478492	9606.ENSP00000356792	0	0	0	0	0	0	0.9	0	0.9
CYSLTR1	PTGFR	9606.ENSP00000478492	9606.ENSP00000359793	0	0	0	0	0	0	0.9	0.163	0.912
CYSLTR1	EDN2	9606.ENSP00000478492	9606.ENSP00000361668	0	0	0	0	0	0	0.9	0	0.9
CYSLTR1	EDN1	9606.ENSP00000478492	9606.ENSP00000368683	0	0	0	0	0	0	0.9	0.108	0.906
CYSLTR1	SAA1	9606.ENSP00000478492	9606.ENSP00000384906	0	0	0	0	0	0	0.9	0	0.9
DDX58	PLCG1	9606.ENSP00000369213	9606.ENSP00000244007	0	0	0	0	0	0	0.9	0.042	0.9
DDX58	TLR2	9606.ENSP00000369213	9606.ENSP00000260010	0	0	0	0	0.096	0	0	0.798	0.809
DDX58	MAPK3	9606.ENSP00000369213	9606.ENSP00000263025	0	0	0	0	0	0	0.9	0.255	0.922
DDX58	IFIH1	9606.ENSP00000369213	9606.ENSP00000263642	0	0	0	0.752	0.783	0	0.9	0.929	0.982
DDX58	TLR3	9606.ENSP00000369213	9606.ENSP00000296795	0	0	0	0	0.114	0	0	0.949	0.953
DDX58	JAK1	9606.ENSP00000369213	9606.ENSP00000343204	0	0	0	0	0.05	0.053	0.9	0.515	0.95
DDX58	TLR1	9606.ENSP00000369213	9606.ENSP00000354932	0	0	0	0	0.095	0	0	0.832	0.841
DDX58	TLR4	9606.ENSP00000369213	9606.ENSP00000363089	0	0	0	0	0.085	0	0	0.797	0.806
DDX58	IRF9	9606.ENSP00000369213	9606.ENSP00000380073	0	0	0	0	0.426	0	0	0.671	0.803
DDX58	OAS1	9606.ENSP00000369213	9606.ENSP00000388001	0	0	0	0	0.782	0	0	0.669	0.924
DDX58	IKBKG	9606.ENSP00000369213	9606.ENSP00000483825	0	0	0	0	0	0.305	0.9	0.57	0.967
DDX58	IKBKE	9606.ENSP00000369213	9606.ENSP00000464030	0	0	0	0	0.089	0.394	0.9	0.646	0.977
DDX58	MX1	9606.ENSP00000369213	9606.ENSP00000381601	0	0	0	0	0.783	0	0.9	0.738	0.993
DDX58	IRF7	9606.ENSP00000369213	9606.ENSP00000380697	0	0	0	0	0.642	0	0.9	0.895	0.995
DES	TPM2	9606.ENSP00000363071	9606.ENSP00000367542	0	0	0	0	0.156	0	0.9	0.058	0.913
DKK1	WNT5A	9606.ENSP00000363081	9606.ENSP00000264634	0	0	0	0	0.098	0.174	0	0.832	0.864
DKK1	PTHLH	9606.ENSP00000363081	9606.ENSP00000441765	0	0	0	0	0.12	0	0	0.717	0.741
DMBT1	SFTPD	9606.ENSP00000357905	9606.ENSP00000361366	0	0	0	0	0.063	0.379	0.9	0.819	0.988
EBI3	JAK1	9606.ENSP00000221847	9606.ENSP00000343204	0	0	0	0	0.061	0	0.9	0.062	0.904
EBI3	IL6ST	9606.ENSP00000221847	9606.ENSP00000370698	0	0	0	0	0	0	0.9	0.15	0.911
EDN1	PLCG1	9606.ENSP00000368683	9606.ENSP00000244007	0	0	0	0	0	0	0.9	0.059	0.901
EDN1	NTS	9606.ENSP00000368683	9606.ENSP00000256010	0	0	0	0	0	0	0.9	0.364	0.933
EDN1	PIK3R3	9606.ENSP00000368683	9606.ENSP00000262741	0	0	0	0	0	0	0.9	0.229	0.919
EDN1	MAPK3	9606.ENSP00000368683	9606.ENSP00000263025	0	0	0	0	0	0	0.9	0.664	0.964
EDN1	IL1B	9606.ENSP00000368683	9606.ENSP00000263341	0	0	0	0	0.062	0	0	0.725	0.731
EDN1	ICAM1	9606.ENSP00000368683	9606.ENSP00000264832	0	0	0	0	0.065	0	0	0.801	0.807
EDN1	KNG1	9606.ENSP00000368683	9606.ENSP00000265023	0	0	0	0	0	0	0.9	0.731	0.971
EDN1	EGFR	9606.ENSP00000368683	9606.ENSP00000275493	0	0	0	0	0.098	0	0.9	0.482	0.949
EDN1	FLT1	9606.ENSP00000368683	9606.ENSP00000282397	0	0	0	0	0.061	0	0	0.693	0.7
EDN1	PTGER1	9606.ENSP00000368683	9606.ENSP00000292513	0	0	0	0	0	0.157	0.9	0.245	0.93
EDN1	VCAM1	9606.ENSP00000368683	9606.ENSP00000294728	0	0	0	0	0	0	0	0.82	0.82
EDN1	PROK2	9606.ENSP00000368683	9606.ENSP00000295619	0	0	0	0	0	0	0.9	0.046	0.9
EDN1	F2RL1	9606.ENSP00000368683	9606.ENSP00000296677	0	0	0	0	0	0	0.9	0.229	0.919
EDN1	OXTR	9606.ENSP00000368683	9606.ENSP00000324270	0	0	0	0	0	0.157	0.9	0.168	0.923
EDN1	NOS2	9606.ENSP00000368683	9606.ENSP00000327251	0	0	0	0	0.049	0	0	0.709	0.712
EDN1	PTGS2	9606.ENSP00000368683	9606.ENSP00000356438	0	0	0	0	0.089	0	0	0.786	0.796
EDN1	XCL1	9606.ENSP00000368683	9606.ENSP00000356792	0	0	0	0	0	0	0.9	0.111	0.907
EDN1	PTGFR	9606.ENSP00000368683	9606.ENSP00000359793	0	0	0	0	0	0.157	0.9	0.194	0.926
EDN1	EDN2	9606.ENSP00000368683	9606.ENSP00000361668	0	0	0	0.8	0	0.809	0.9	0.921	0.983
EDN1	SRC	9606.ENSP00000368683	9606.ENSP00000362680	0	0	0	0	0.06	0	0.9	0.722	0.971
EDN1	PGF	9606.ENSP00000368683	9606.ENSP00000451040	0	0	0	0	0	0	0	0.765	0.765
EDN1	IL6	9606.ENSP00000368683	9606.ENSP00000385675	0	0	0	0	0.088	0	0	0.852	0.859
EDN1	SAA1	9606.ENSP00000368683	9606.ENSP00000384906	0	0	0	0	0	0	0.9	0.105	0.906
EDN1	HCK	9606.ENSP00000368683	9606.ENSP00000444986	0	0	0	0	0	0	0.9	0.196	0.916
EDN2	NTS	9606.ENSP00000361668	9606.ENSP00000256010	0	0	0	0	0.061	0	0.9	0.053	0.903
EDN2	PIK3R3	9606.ENSP00000361668	9606.ENSP00000262741	0	0	0	0	0	0	0.9	0.043	0.9

EDN2	KNG1	9606.ENSP00000361668	9606.ENSP00000265023	0	0	0	0	0	0	0.9	0.297	0.926
EDN2	PTGER1	9606.ENSP00000361668	9606.ENSP00000292513	0	0	0	0	0	0.157	0.9	0.139	0.921
EDN2	PROK2	9606.ENSP00000361668	9606.ENSP00000295619	0	0	0	0	0	0	0.9	0.204	0.916
EDN2	F2RL1	9606.ENSP00000361668	9606.ENSP00000296677	0	0	0	0	0	0	0.9	0.05	0.9
EDN2	OXTR	9606.ENSP00000361668	9606.ENSP00000324270	0	0	0	0	0	0.157	0.9	0	0.912
EDN2	XCL1	9606.ENSP00000361668	9606.ENSP00000356792	0	0	0	0	0	0	0.9	0	0.9
EDN2	PTGFR	9606.ENSP00000361668	9606.ENSP00000359793	0	0	0	0	0	0.157	0.9	0.154	0.922
EDN2	SAA1	9606.ENSP00000361668	9606.ENSP00000384906	0	0	0	0	0	0	0.9	0	0.9
EGFR	KITLG	9606.ENSP00000275493	9606.ENSP00000228280	0	0	0	0	0.197	0.381	0.8	0.511	0.944
EGFR	PLCG1	9606.ENSP00000275493	9606.ENSP00000244007	0	0	0	0	0.06	0.778	0.9	0.77	0.994
EGFR	EREG	9606.ENSP00000275493	9606.ENSP00000244869	0	0	0	0	0	0.87	0.9	0.861	0.998
EGFR	LIF	9606.ENSP00000275493	9606.ENSP00000249075	0	0	0	0	0.098	0	0.6	0.354	0.746
EGFR	JAG1	9606.ENSP00000275493	9606.ENSP00000254958	0	0	0	0	0.121	0.134	0	0.713	0.762
EGFR	PIK3R3	9606.ENSP00000275493	9606.ENSP00000262741	0	0	0	0	0	0.558	0.8	0.398	0.942
EGFR	MAPK3	9606.ENSP00000275493	9606.ENSP00000263025	0	0	0	0.58	0	0.185	0.9	0.886	0.946
EGFR	FGF2	9606.ENSP00000275493	9606.ENSP00000264498	0	0	0	0	0.098	0	0.6	0.824	0.931
EGFR	SHC2	9606.ENSP00000275493	9606.ENSP00000264554	0	0	0	0	0.076	0.471	0.8	0.365	0.929
EGFR	IL11	9606.ENSP00000275493	9606.ENSP00000264563	0	0	0	0	0.041	0	0.6	0.356	0.731
EGFR	WNT5A	9606.ENSP00000275493	9606.ENSP00000264634	0	0	0	0	0.14	0.195	0.9	0.525	0.962
EGFR	MET	9606.ENSP00000275493	9606.ENSP00000317272	0	0	0	0.58	0.229	0.418	0	0.933	0.715
EGFR	IL15	9606.ENSP00000275493	9606.ENSP00000296545	0	0	0	0	0	0	0.6	0.348	0.728
EGFR	SDC1	9606.ENSP00000275493	9606.ENSP00000370542	0	0	0	0	0.176	0	0	0.693	0.737
EGFR	FGF13	9606.ENSP00000275493	9606.ENSP00000322390	0	0	0	0	0	0	0	0.742	0.742
EGFR	FGF5	9606.ENSP00000275493	9606.ENSP00000311697	0	0	0	0	0	0	0.6	0.4	0.749
EGFR	ESR2	9606.ENSP00000275493	9606.ENSP00000343925	0	0	0	0	0	0.077	0	0.739	0.749
EGFR	FIGF	9606.ENSP00000275493	9606.ENSP00000297904	0	0	0	0	0	0	0.6	0.421	0.758
EGFR	PDGFD	9606.ENSP00000275493	9606.ENSP00000376865	0	0	0	0	0.049	0	0.6	0.429	0.763
EGFR	PGF	9606.ENSP00000275493	9606.ENSP00000451040	0	0	0	0	0	0	0.6	0.473	0.78
EGFR	TLR4	9606.ENSP00000275493	9606.ENSP00000363089	0	0	0	0	0	0.321	0	0.695	0.784
EGFR	MUC5AC	9606.ENSP00000275493	9606.ENSP00000485659	0	0	0	0	0	0.112	0	0.772	0.789
EGFR	FGF9	9606.ENSP00000275493	9606.ENSP00000371790	0	0	0	0	0.052	0	0.6	0.503	0.795
EGFR	FGF19	9606.ENSP00000275493	9606.ENSP00000294312	0	0	0	0	0	0	0.6	0.519	0.799
EGFR	PDGFA	9606.ENSP00000275493	9606.ENSP00000346508	0	0	0	0	0	0.131	0.6	0.526	0.82
EGFR	NGF	9606.ENSP00000275493	9606.ENSP00000358525	0	0	0	0	0.098	0	0.6	0.558	0.826
EGFR	VEGFC	9606.ENSP00000275493	9606.ENSP00000480043	0	0	0	0	0.175	0	0.6	0.519	0.827
EGFR	PDGFB	9606.ENSP00000275493	9606.ENSP00000330382	0	0	0	0	0	0.185	0.6	0.596	0.856
EGFR	NEDD4	9606.ENSP00000275493	9606.ENSP00000424827	0	0	0	0	0.055	0.401	0.6	0.491	0.869
EGFR	PTGS2	9606.ENSP00000275493	9606.ENSP00000356438	0	0	0	0	0.061	0	0	0.869	0.872
EGFR	IL7R	9606.ENSP00000275493	9606.ENSP00000306157	0	0	0	0	0	0	0.9	0.098	0.906
EGFR	IL6	9606.ENSP00000275493	9606.ENSP00000385675	0	0	0	0	0	0	0.6	0.811	0.921
EGFR	SHC3	9606.ENSP00000275493	9606.ENSP00000364995	0	0	0	0	0.098	0.522	0.8	0.337	0.935
EGFR	FYN	9606.ENSP00000275493	9606.ENSP00000346671	0	0	0	0.676	0	0.185	0.9	0.777	0.936
EGFR	PRKCB	9606.ENSP00000275493	9606.ENSP00000305355	0	0	0	0.553	0.049	0.403	0.9	0.304	0.945
EGFR	PLAU	9606.ENSP00000275493	9606.ENSP00000361850	0	0	0	0	0.088	0	0.9	0.513	0.951
EGFR	PLAUR	9606.ENSP00000275493	9606.ENSP00000339328	0	0	0	0	0	0	0.9	0.561	0.954
EGFR	PTK2B	9606.ENSP00000275493	9606.ENSP00000380638	0	0	0	0.603	0.063	0.467	0.9	0.563	0.957
EGFR	PRKCA	9606.ENSP00000275493	9606.ENSP00000408695	0	0	0	0.557	0.049	0.44	0.9	0.676	0.959
EGFR	SRC	9606.ENSP00000275493	9606.ENSP00000362680	0	0	0	0.671	0	0.484	0.9	0.954	0.963
EGFR	JAK1	9606.ENSP00000275493	9606.ENSP00000343204	0	0	0	0.569	0	0.573	0.9	0.597	0.966
EGFR	HCK	9606.ENSP00000275493	9606.ENSP00000444986	0	0	0	0.679	0	0.701	0.9	0.722	0.975
EGFR	NRG1	9606.ENSP00000275493	9606.ENSP00000384620	0	0	0	0	0.076	0.591	0.9	0.874	0.994
EGFR	TGFA	9606.ENSP00000275493	9606.ENSP00000295400	0	0	0	0	0.065	0.873	0.9	0.874	0.998
EGFR	SHC1	9606.ENSP00000275493	9606.ENSP00000401303	0	0	0	0	0.069	0.9	0.9	0.895	0.998
ELANE	ICAM1	9606.ENSP00000466090	9606.ENSP00000264832	0	0	0	0	0	0	0	0.729	0.729
ELANE	PLAUR	9606.ENSP00000466090	9606.ENSP00000339328	0	0	0	0	0	0.133	0.9	0.163	0.921
ELANE	SLPI	9606.ENSP00000466090	9606.ENSP00000342082	0	0	0	0	0.061	0.87	0.9	0.833	0.997
ELANE	PTGS2	9606.ENSP00000466090	9606.ENSP00000356438	0	0	0	0	0	0.061	0	0.7	0.707
ELANE	LCN2	9606.ENSP00000466090	9606.ENSP00000362108	0	0	0	0	0.12	0	0.9	0.442	0.946
ELANE	IL6	9606.ENSP00000466090	9606.ENSP00000385675	0	0	0	0	0	0	0	0.793	0.793
ELANE	PTPN6	9606.ENSP00000466090	9606.ENSP00000391592	0	0	0	0	0.069	0	0.9	0	0.902
ELANE	MUC5AC	9606.ENSP00000466090	9606.ENSP00000485659	0	0	0	0	0	0	0	0.756	0.756
ENG	TGFB3	9606.ENSP00000362299	9606.ENSP00000238682	0	0	0	0	0.076	0.379	0.9	0.479	0.966
ENG	MAPK3	9606.ENSP00000362299	9606.ENSP00000263025	0	0	0	0	0	0	0.9	0.387	0.936
ENG	FLT1	9606.ENSP00000362299	9606.ENSP00000282397	0	0	0	0	0.063	0	0	0.705	0.711
ENG	VCAM1	9606.ENSP00000362299	9606.ENSP00000294728	0	0	0	0	0.076	0	0	0.73	0.739
ENG	TGFBR2	9606.ENSP00000362299	9606.ENSP00000351905	0	0	0	0	0.173	0.468	0.9	0.469	0.973
ENG	PGF	9606.ENSP00000362299	9606.ENSP00000451040	0	0	0	0	0	0	0	0.716	0.716
EREG	PTGS2	9606.ENSP00000244869	9606.ENSP00000356438	0	0	0	0	0.097	0	0	0.707	0.724
EREG	IGF1R	9606.ENSP00000244869	9606.ENSP00000268035	0	0	0	0	0	0	0.6	0.358	0.732
EREG	MET	9606.ENSP00000244869	9606.ENSP00000317272	0	0	0	0	0.061	0	0.6	0.376	0.745
EREG	FYN	9606.ENSP00000244869	9606.ENSP00000346671	0	0	0	0	0.061	0	0.9	0.08	0.906
EREG	SHC1	9606.ENSP00000244869	9606.ENSP00000401303	0	0	0	0	0	0	0.9	0.291	0.926

EREG	SRC	9606.ENSP00000244869	9606.ENSP00000362680	0	0	0	0	0.061	0	0.9	0.397	0.938
EREG	NRG1	9606.ENSP00000244869	9606.ENSP00000384620	0	0	0	0	0.061	0	0.9	0.593	0.958
ESR2	PIK3R3	9606.ENSP00000343925	9606.ENSP00000262741	0	0	0	0	0	0.081	0.9	0.046	0.904
ESR2	MAPK3	9606.ENSP00000343925	9606.ENSP00000263025	0	0	0	0	0	0.073	0.8	0.523	0.903
ESR2	NR0B1	9606.ENSP00000343925	9606.ENSP00000368253	0	0	0	0	0	0.379	0.9	0.33	0.954
ESR2	SRC	9606.ENSP00000343925	9606.ENSP00000362680	0	0	0	0	0	0.407	0.9	0.459	0.965
F2RL1	NTS	9606.ENSP00000296677	9606.ENSP00000256010	0	0	0	0	0	0	0.9	0.099	0.906
F2RL1	PIK3R3	9606.ENSP00000296677	9606.ENSP00000262741	0	0	0	0	0	0	0.9	0.063	0.902
F2RL1	KNG1	9606.ENSP00000296677	9606.ENSP00000265023	0	0	0	0	0	0	0.9	0.291	0.926
F2RL1	PTGER1	9606.ENSP00000296677	9606.ENSP00000292513	0	0	0	0	0	0	0.9	0.298	0.926
F2RL1	PROK2	9606.ENSP00000296677	9606.ENSP00000295619	0	0	0	0	0	0	0.9	0.061	0.902
F2RL1	TLR4	9606.ENSP00000296677	9606.ENSP00000363089	0	0	0	0	0	0.379	0	0.543	0.704
F2RL1	S1PR1	9606.ENSP00000296677	9606.ENSP00000305416	0	0	0	0	0	0	0	0.755	0.755
F2RL1	XCL1	9606.ENSP00000296677	9606.ENSP00000356792	0	0	0	0	0	0	0.9	0	0.9
F2RL1	SAA1	9606.ENSP00000296677	9606.ENSP00000384906	0	0	0	0	0.06	0	0.9	0.049	0.902
F2RL1	PTGFR	9606.ENSP00000296677	9606.ENSP00000359793	0	0	0	0	0	0	0.9	0.082	0.904
F2RL1	OXTR	9606.ENSP00000296677	9606.ENSP00000324270	0	0	0	0.566	0	0	0.9	0.172	0.905
FGA	PROC	9606.ENSP00000306361	9606.ENSP00000234071	0	0	0	0	0.586	0.157	0.9	0.265	0.97
FGA	FGF23	9606.ENSP00000306361	9606.ENSP00000237837	0	0	0	0	0.061	0	0.9	0	0.902
FGA	TGFB3	9606.ENSP00000306361	9606.ENSP00000238682	0	0	0	0	0	0	0.9	0.295	0.926
FGA	THBS1	9606.ENSP00000306361	9606.ENSP00000260356	0	0	0	0	0	0.379	0.9	0.25	0.949
FGA	ITGAV	9606.ENSP00000306361	9606.ENSP00000261023	0	0	0	0	0	0	0.9	0.254	0.922
FGA	MAPK3	9606.ENSP00000306361	9606.ENSP00000263025	0	0	0	0	0	0	0.9	0.216	0.918
FGA	KNG1	9606.ENSP00000306361	9606.ENSP00000265023	0	0	0	0	0.296	0	0.9	0.329	0.948
FGA	FIGF	9606.ENSP00000306361	9606.ENSP00000297904	0	0	0	0	0	0	0.9	0.041	0.9
FGA	SCG2	9606.ENSP00000306361	9606.ENSP00000304133	0	0	0	0	0.062	0	0.9	0	0.902
FGA	NR1H4	9606.ENSP00000306361	9606.ENSP00000447149	0	0	0	0	0.643	0.05	0	0.207	0.707
FGA	VEGFC	9606.ENSP00000306361	9606.ENSP00000480043	0	0	0	0	0	0	0.9	0	0.9
FGA	SDC2	9606.ENSP00000306361	9606.ENSP00000307046	0	0	0	0	0.061	0	0.9	0.052	0.903
FGA	TLR4	9606.ENSP00000306361	9606.ENSP00000363089	0	0	0	0	0	0.056	0.9	0.079	0.905
FGA	SPP1	9606.ENSP00000306361	9606.ENSP00000378517	0	0	0	0	0.076	0	0.9	0.091	0.908
FGA	PLAUR	9606.ENSP00000306361	9606.ENSP00000339328	0	0	0	0	0	0	0.9	0.157	0.912
FGA	TMSB4X	9606.ENSP00000306361	9606.ENSP00000370010	0	0	0	0	0	0	0.9	0.186	0.915
FGA	SRC	9606.ENSP00000306361	9606.ENSP00000362680	0	0	0	0	0.062	0.054	0.9	0.218	0.921
FGA	IL6	9606.ENSP00000306361	9606.ENSP00000385675	0	0	0	0	0	0	0.9	0.25	0.921
FGA	TGFB2	9606.ENSP00000306361	9606.ENSP00000355896	0	0	0	0	0	0	0.9	0.257	0.922
FGA	PDGFB	9606.ENSP00000306361	9606.ENSP00000330382	0	0	0	0	0	0	0.9	0.286	0.925
FGA	ITGB2	9606.ENSP00000306361	9606.ENSP00000380948	0	0	0	0	0.082	0	0.9	0.254	0.925
FGA	SYK	9606.ENSP00000306361	9606.ENSP00000364907	0	0	0	0	0.083	0.054	0.9	0.254	0.926
FGA	PLAU	9606.ENSP00000306361	9606.ENSP00000361850	0	0	0	0	0	0.157	0.9	0.411	0.946
FGF11	FGFR2	9606.ENSP00000293829	9606.ENSP00000410294	0	0	0	0	0	0.267	0	0.673	0.75
FGF12	FGFR2	9606.ENSP00000413496	9606.ENSP00000410294	0	0	0	0	0.076	0.267	0	0.704	0.782
FGF13	VEGFC	9606.ENSP00000322390	9606.ENSP00000480043	0	0	0	0	0	0	0	0.705	0.705
FGF13	NGF	9606.ENSP00000322390	9606.ENSP00000358525	0	0	0	0	0	0	0	0.736	0.737
FGF13	FGFR3	9606.ENSP00000322390	9606.ENSP00000339824	0	0	0	0	0	0.267	0	0.739	0.8
FGF13	FGFR2	9606.ENSP00000322390	9606.ENSP00000410294	0	0	0	0	0.061	0.267	0	0.874	0.906
FGF14	FGFR2	9606.ENSP00000365301	9606.ENSP00000410294	0	0	0	0	0	0.267	0	0.647	0.73
FGF19	FGF23	9606.ENSP00000294312	9606.ENSP00000237837	0	0	0	0.701	0	0	0.9	0.736	0.921
FGF19	FLT3	9606.ENSP00000294312	9606.ENSP00000241453	0	0	0	0	0	0.267	0.6	0.228	0.754
FGF19	PLCG1	9606.ENSP00000294312	9606.ENSP00000244007	0	0	0	0	0	0.052	0.9	0.054	0.902
FGF19	FLT4	9606.ENSP00000294312	9606.ENSP00000261937	0	0	0	0	0	0.267	0.6	0.268	0.766
FGF19	MAPK3	9606.ENSP00000294312	9606.ENSP00000263025	0	0	0	0	0	0	0.9	0.464	0.944
FGF19	FGF2	9606.ENSP00000294312	9606.ENSP00000264498	0	0	0	0.641	0	0	0.9	0.756	0.926
FGF19	IGF1R	9606.ENSP00000294312	9606.ENSP00000268035	0	0	0	0	0	0.05	0.6	0.292	0.707
FGF19	FLT1	9606.ENSP00000294312	9606.ENSP00000282397	0	0	0	0	0	0.267	0.6	0.406	0.81
FGF19	MET	9606.ENSP00000294312	9606.ENSP00000317272	0	0	0	0	0	0.05	0.6	0.306	0.713
FGF19	NR1H4	9606.ENSP00000294312	9606.ENSP00000447149	0	0	0	0	0	0.379	0	0.848	0.901
FGF19	SDC2	9606.ENSP00000294312	9606.ENSP00000307046	0	0	0	0	0	0	0.9	0.08	0.904
FGF19	SDC1	9606.ENSP00000294312	9606.ENSP00000370542	0	0	0	0	0.051	0	0.9	0.249	0.922
FGF19	FGF9	9606.ENSP00000294312	9606.ENSP00000371790	0	0	0	0	0	0	0.9	0.429	0.94
FGF19	FGFR3	9606.ENSP00000294312	9606.ENSP00000339824	0	0	0	0	0	0.267	0.9	0.597	0.967
FGF19	FGFR2	9606.ENSP00000294312	9606.ENSP00000410294	0	0	0	0	0	0.267	0.9	0.709	0.976
FGF2	NGFR	9606.ENSP00000264498	9606.ENSP00000172229	0	0	0	0	0	0	0.6	0.535	0.806
FGF2	VTN	9606.ENSP00000264498	9606.ENSP00000226218	0	0	0	0	0	0.305	0	0.649	0.746
FGF2	KITLG	9606.ENSP00000264498	9606.ENSP00000228280	0	0	0	0	0.078	0	0	0.69	0.702
FGF2	FGF23	9606.ENSP00000264498	9606.ENSP00000237837	0	0	0	0.597	0	0	0.9	0.693	0.927
FGF2	TGFB3	9606.ENSP00000264498	9606.ENSP00000238682	0	0	0	0	0.062	0.05	0	0.724	0.733
FGF2	FLT3	9606.ENSP00000264498	9606.ENSP00000241453	0	0	0	0	0	0.267	0.6	0.482	0.834
FGF2	PLCG1	9606.ENSP00000264498	9606.ENSP00000244007	0	0	0	0	0	0.052	0.9	0.259	0.923
FGF2	LIF	9606.ENSP00000264498	9606.ENSP00000249075	0	0	0	0	0	0	0	0.786	0.786
FGF2	THBS1	9606.ENSP00000264498	9606.ENSP00000260356	0	0	0	0	0.061	0.379	0	0.796	0.871
FGF2	ITGAV	9606.ENSP00000264498	9606.ENSP00000261023	0	0	0	0	0.071	0	0.9	0.349	0.934

FGF2	FLT4	9606.ENSP00000264498	9606.ENSP00000261937	0	0	0	0	0	0.267	0.6	0.593	0.87
FGF2	MAPK3	9606.ENSP00000264498	9606.ENSP00000263025	0	0	0	0	0	0	0.9	0.715	0.97
FGF2	IL1B	9606.ENSP00000264498	9606.ENSP00000263341	0	0	0	0	0	0	0	0.815	0.815
FGF2	PGF	9606.ENSP00000264498	9606.ENSP00000451040	0	0	0	0	0	0	0	0.71	0.71
FGF2	ICAM1	9606.ENSP00000264498	9606.ENSP00000264832	0	0	0	0	0	0	0	0.738	0.739
FGF2	PTGS2	9606.ENSP00000264498	9606.ENSP00000356438	0	0	0	0	0.057	0	0	0.736	0.74
FGF2	SPP1	9606.ENSP00000264498	9606.ENSP00000378517	0	0	0	0	0	0	0	0.768	0.768
FGF2	NRP1	9606.ENSP00000264498	9606.ENSP00000265371	0	0	0	0	0.059	0.305	0	0.677	0.77
FGF2	MET	9606.ENSP00000264498	9606.ENSP00000317272	0	0	0	0	0.069	0.05	0.6	0.568	0.826
FGF2	VEGFC	9606.ENSP00000264498	9606.ENSP00000480043	0	0	0	0	0.088	0	0	0.825	0.834
FGF2	IL6	9606.ENSP00000264498	9606.ENSP00000385675	0	0	0	0	0.061	0	0	0.854	0.857
FGF2	GDNF	9606.ENSP00000264498	9606.ENSP00000409007	0	0	0	0	0.061	0	0	0.882	0.885
FGF2	IGF1R	9606.ENSP00000264498	9606.ENSP00000268035	0	0	0	0	0	0.05	0.6	0.726	0.886
FGF2	NGF	9606.ENSP00000264498	9606.ENSP00000358525	0	0	0	0	0.104	0	0	0.893	0.9
FGF2	FLT1	9606.ENSP00000264498	9606.ENSP00000282397	0	0	0	0	0.063	0.267	0.6	0.708	0.909
FGF2	FGF9	9606.ENSP00000264498	9606.ENSP00000371790	0	0	0	0.742	0	0	0.9	0.789	0.92
FGF2	JAK1	9606.ENSP00000264498	9606.ENSP00000343204	0	0	0	0	0	0.05	0.9	0.295	0.927
FGF2	SDC2	9606.ENSP00000264498	9606.ENSP00000307046	0	0	0	0	0.077	0.305	0.9	0.654	0.974
FGF2	SDC4	9606.ENSP00000264498	9606.ENSP00000361818	0	0	0	0	0	0.305	0.9	0.73	0.979
FGF2	SDC1	9606.ENSP00000264498	9606.ENSP00000370542	0	0	0	0	0	0.305	0.9	0.755	0.981
FGF2	FGFR3	9606.ENSP00000264498	9606.ENSP00000339824	0	0	0	0	0	0.794	0.9	0.695	0.993
FGF2	FGFR2	9606.ENSP00000264498	9606.ENSP00000410294	0	0	0	0	0	0.863	0.9	0.885	0.998
FGF23	PROC	9606.ENSP00000237837	9606.ENSP00000234071	0	0	0	0	0	0	0.9	0	0.9
FGF23	FLT3	9606.ENSP00000237837	9606.ENSP00000241453	0	0	0	0	0	0.267	0.6	0.062	0.701
FGF23	FLT4	9606.ENSP00000237837	9606.ENSP00000261937	0	0	0	0	0	0.267	0.6	0.085	0.708
FGF23	FLT1	9606.ENSP00000237837	9606.ENSP00000282397	0	0	0	0	0	0.267	0.6	0.16	0.732
FGF23	SDC2	9606.ENSP00000237837	9606.ENSP00000307046	0	0	0	0	0	0	0.9	0.05	0.9
FGF23	SCG2	9606.ENSP00000237837	9606.ENSP00000304133	0	0	0	0	0	0	0.9	0	0.9
FGF23	PLCG1	9606.ENSP00000237837	9606.ENSP00000244007	0	0	0	0	0	0.052	0.9	0	0.901
FGF23	KNG1	9606.ENSP00000237837	9606.ENSP00000265023	0	0	0	0	0	0	0.9	0.064	0.902
FGF23	SDC1	9606.ENSP00000237837	9606.ENSP00000370542	0	0	0	0	0	0	0.9	0.079	0.904
FGF23	PTK2B	9606.ENSP00000237837	9606.ENSP00000380638	0	0	0	0	0	0	0.9	0.133	0.909
FGF23	MAPK3	9606.ENSP00000237837	9606.ENSP00000263025	0	0	0	0	0	0	0.9	0.404	0.937
FGF23	FGF9	9606.ENSP00000237837	9606.ENSP00000371790	0	0	0	0	0	0	0.9	0.479	0.945
FGF23	SPP1	9606.ENSP00000237837	9606.ENSP00000378517	0	0	0	0	0	0	0.9	0.627	0.961
FGF23	IL6	9606.ENSP00000237837	9606.ENSP00000385675	0	0	0	0	0	0	0.9	0.651	0.963
FGF23	FGFR2	9606.ENSP00000237837	9606.ENSP00000410294	0	0	0	0	0.049	0.65	0.9	0.595	0.984
FGF23	FGFR3	9606.ENSP00000237837	9606.ENSP00000339824	0	0	0	0	0.049	0.549	0.9	0.695	0.985
FGF5	FLT3	9606.ENSP00000311697	9606.ENSP00000241453	0	0	0	0	0	0.267	0.6	0.063	0.701
FGF5	FLT4	9606.ENSP00000311697	9606.ENSP00000261937	0	0	0	0	0	0.267	0.6	0.223	0.752
FGF5	FLT1	9606.ENSP00000311697	9606.ENSP00000282397	0	0	0	0	0	0.267	0.6	0.372	0.8
FGF5	FGFR3	9606.ENSP00000311697	9606.ENSP00000339824	0	0	0	0	0	0.48	0.6	0.496	0.886
FGF5	FGFR2	9606.ENSP00000311697	9606.ENSP00000410294	0	0	0	0	0	0.627	0.6	0.663	0.945
FGF9	FLT3	9606.ENSP00000371790	9606.ENSP00000241453	0	0	0	0	0	0.267	0.6	0.118	0.718
FGF9	PLCG1	9606.ENSP00000371790	9606.ENSP00000244007	0	0	0	0	0.055	0.052	0.9	0.043	0.902
FGF9	FLT4	9606.ENSP00000371790	9606.ENSP00000261937	0	0	0	0	0	0.267	0.6	0.278	0.77
FGF9	MAPK3	9606.ENSP00000371790	9606.ENSP00000263025	0	0	0	0	0.041	0	0.9	0.434	0.941
FGF9	IGF1R	9606.ENSP00000371790	9606.ENSP00000268035	0	0	0	0	0	0.05	0.6	0.288	0.706
FGF9	FLT1	9606.ENSP00000371790	9606.ENSP00000282397	0	0	0	0	0	0.267	0.6	0.433	0.819
FGF9	FGFR3	9606.ENSP00000371790	9606.ENSP00000339824	0	0	0	0	0.063	0.725	0.9	0.733	0.992
FGF9	FGFR2	9606.ENSP00000371790	9606.ENSP00000410294	0	0	0	0	0.063	0.751	0.9	0.735	0.993
FGFR2	KITLG	9606.ENSP00000410294	9606.ENSP00000228280	0	0	0	0	0	0.087	0.6	0.246	0.7
FGFR2	PLCG1	9606.ENSP00000410294	9606.ENSP00000244007	0	0	0	0	0.054	0.979	0.9	0.196	0.998
FGFR2	MAPK3	9606.ENSP00000410294	9606.ENSP00000263025	0	0	0	0.584	0	0.084	0.9	0.635	0.929
FGFR2	SHC2	9606.ENSP00000410294	9606.ENSP00000264554	0	0	0	0	0.071	0.157	0.8	0.126	0.844
FGFR2	SDC2	9606.ENSP00000410294	9606.ENSP00000307046	0	0	0	0	0	0.119	0.9	0.189	0.922
FGFR2	PDGFB	9606.ENSP00000410294	9606.ENSP00000330382	0	0	0	0	0.055	0.185	0.6	0.392	0.787
FGFR2	FGFR3	9606.ENSP00000410294	9606.ENSP00000339824	0	0	0	0.966	0.156	0.613	0.9	0.908	0.965
FGFR2	JAK1	9606.ENSP00000410294	9606.ENSP00000343204	0	0	0	0.606	0	0.096	0.8	0.213	0.824
FGFR2	PDGFA	9606.ENSP00000410294	9606.ENSP00000346508	0	0	0	0	0	0.185	0.6	0.374	0.778
FGFR2	SRC	9606.ENSP00000410294	9606.ENSP00000362680	0	0	0	0.706	0.062	0.465	0.9	0.533	0.953
FGFR2	SHC3	9606.ENSP00000410294	9606.ENSP00000364995	0	0	0	0	0.06	0.157	0.8	0.126	0.842
FGFR2	SDC1	9606.ENSP00000410294	9606.ENSP00000370542	0	0	0	0	0.06	0	0.9	0.179	0.916
FGFR2	SHC1	9606.ENSP00000410294	9606.ENSP00000401303	0	0	0	0	0	0.454	0.9	0.269	0.956
FGFR2	VEGFC	9606.ENSP00000410294	9606.ENSP00000480043	0	0	0	0	0	0.174	0.6	0.287	0.744
FGFR3	PLCG1	9606.ENSP00000339824	9606.ENSP00000244007	0	0	0	0	0.077	0.711	0.9	0.282	0.978
FGFR3	PIK3R3	9606.ENSP00000339824	9606.ENSP00000262741	0	0	0	0	0	0.097	0.6	0.386	0.758
FGFR3	MAPK3	9606.ENSP00000339824	9606.ENSP00000263025	0	0	0	0.58	0	0.084	0.9	0.622	0.928
FGFR3	SHC2	9606.ENSP00000339824	9606.ENSP00000264554	0	0	0	0	0.111	0.157	0.8	0.114	0.849
FGFR3	SDC2	9606.ENSP00000339824	9606.ENSP00000307046	0	0	0	0	0	0.184	0.9	0.136	0.923
FGFR3	PDGFB	9606.ENSP00000339824	9606.ENSP00000330382	0	0	0	0	0.055	0.185	0.6	0.262	0.742
FGFR3	VEGFC	9606.ENSP00000339824	9606.ENSP00000480043	0	0	0	0	0.05	0.174	0.6	0.275	0.742

FGFR3	PDGFA	9606.ENSP00000339824	9606.ENSP00000346508	0	0	0	0	0	0.185	0.6	0.36	0.773
FGFR3	JAK1	9606.ENSP00000339824	9606.ENSP00000343204	0	0	0	0.605	0	0.096	0.8	0.254	0.828
FGFR3	SHC3	9606.ENSP00000339824	9606.ENSP00000364995	0	0	0	0	0.07	0.157	0.8	0.114	0.842
FGFR3	SRC	9606.ENSP00000339824	9606.ENSP00000362680	0	0	0	0.702	0.062	0.175	0.9	0.502	0.927
FGFR3	SHC1	9606.ENSP00000339824	9606.ENSP00000401303	0	0	0	0	0	0.157	0.9	0.233	0.929
FGFR3	SDC1	9606.ENSP00000339824	9606.ENSP00000370542	0	0	0	0	0.076	0	0.9	0.44	0.943
FGFR3	PTK2B	9606.ENSP00000339824	9606.ENSP00000380638	0	0	0	0.619	0	0.454	0.9	0.202	0.946
FIGF	TGFB3	9606.ENSP00000297904	9606.ENSP00000238682	0	0	0	0	0.061	0	0.9	0.168	0.915
FIGF	FLT3	9606.ENSP00000297904	9606.ENSP00000241453	0	0	0	0	0.062	0.08	0.6	0.492	0.801
FIGF	THBS1	9606.ENSP00000297904	9606.ENSP00000260356	0	0	0	0	0.061	0	0.9	0.625	0.961
FIGF	FLT4	9606.ENSP00000297904	9606.ENSP00000261937	0	0	0	0	0	0.438	0.9	0.958	0.997
FIGF	KNG1	9606.ENSP00000297904	9606.ENSP00000265023	0	0	0	0	0	0	0.9	0.096	0.905
FIGF	IGF1R	9606.ENSP00000297904	9606.ENSP00000268035	0	0	0	0	0	0	0.6	0.297	0.706
FIGF	FLT1	9606.ENSP00000297904	9606.ENSP00000282397	0	0	0	0	0	0.132	0.6	0.829	0.935
FIGF	MET	9606.ENSP00000297904	9606.ENSP00000317272	0	0	0	0	0	0	0.6	0.307	0.71
FIGF	TMSB4X	9606.ENSP00000297904	9606.ENSP00000370010	0	0	0	0	0	0	0.9	0.042	0.9
FIGF	SHC1	9606.ENSP00000297904	9606.ENSP00000401303	0	0	0	0	0	0	0.9	0.153	0.911
FIGF	VEGFC	9606.ENSP00000297904	9606.ENSP00000480043	0	0	0	0.873	0	0	0.9	0.962	0.912
FIGF	TGFB2	9606.ENSP00000297904	9606.ENSP00000355896	0	0	0	0	0	0	0.9	0.194	0.915
FIGF	PGF	9606.ENSP00000297904	9606.ENSP00000451040	0	0	0	0.708	0	0	0.9	0.919	0.926
FIGF	PDGFB	9606.ENSP00000297904	9606.ENSP00000330382	0	0	0	0	0	0	0.9	0.448	0.942
FLT1	KITLG	9606.ENSP00000282397	9606.ENSP00000228280	0	0	0	0	0.061	0.087	0.6	0.575	0.834
FLT1	PLCG1	9606.ENSP00000282397	9606.ENSP00000244007	0	0	0	0	0	0.791	0.9	0.234	0.982
FLT1	FLT4	9606.ENSP00000282397	9606.ENSP00000261937	0	0	0	0.889	0.062	0.732	0.9	0.902	0.975
FLT1	SHC2	9606.ENSP00000282397	9606.ENSP00000264554	0	0	0	0	0	0.474	0.9	0.139	0.95
FLT1	NRP1	9606.ENSP00000282397	9606.ENSP00000265371	0	0	0	0	0.062	0.716	0.9	0.848	0.995
FLT1	TGFA	9606.ENSP00000282397	9606.ENSP00000295400	0	0	0	0	0.061	0	0.6	0.286	0.708
FLT1	NGF	9606.ENSP00000282397	9606.ENSP00000358525	0	0	0	0	0	0	0.6	0.31	0.712
FLT1	HCK	9606.ENSP00000282397	9606.ENSP00000444986	0	0	0	0.689	0	0.699	0	0.224	0.717
FLT1	PDGFD	9606.ENSP00000282397	9606.ENSP00000376865	0	0	0	0	0.061	0	0.6	0.5	0.795
FLT1	PDGFA	9606.ENSP00000282397	9606.ENSP00000346508	0	0	0	0	0	0.185	0.6	0.482	0.816
FLT1	PDGFB	9606.ENSP00000282397	9606.ENSP00000330382	0	0	0	0	0.062	0.393	0.6	0.694	0.921
FLT1	SRC	9606.ENSP00000282397	9606.ENSP00000362680	0	0	0	0.657	0.058	0.161	0.9	0.569	0.93
FLT1	SHC1	9606.ENSP00000282397	9606.ENSP00000401303	0	0	0	0	0	0.418	0.9	0.274	0.954
FLT1	VEGFC	9606.ENSP00000282397	9606.ENSP00000480043	0	0	0	0	0.049	0.185	0.6	0.906	0.967
FLT1	NRP2	9606.ENSP00000282397	9606.ENSP00000353582	0	0	0	0	0	0.521	0.9	0.78	0.988
FLT1	PGF	9606.ENSP00000282397	9606.ENSP00000451040	0	0	0	0	0	0.877	0.9	0.958	0.999
FLT3	KITLG	9606.ENSP00000241453	9606.ENSP00000228280	0	0	0	0	0	0.087	0.6	0.869	0.948
FLT3	SHC1	9606.ENSP00000241453	9606.ENSP00000401303	0	0	0	0	0	0.157	0.6	0.258	0.727
FLT3	PGF	9606.ENSP00000241453	9606.ENSP00000451040	0	0	0	0	0	0.058	0.6	0.348	0.732
FLT3	PDGFA	9606.ENSP00000241453	9606.ENSP00000346508	0	0	0	0	0	0.185	0.6	0.254	0.735
FLT3	PDGFB	9606.ENSP00000241453	9606.ENSP00000330382	0	0	0	0	0	0.185	0.6	0.278	0.744
FLT3	VEGFC	9606.ENSP00000241453	9606.ENSP00000480043	0	0	0	0	0	0.174	0.6	0.348	0.765
FLT3	PIK3R3	9606.ENSP00000241453	9606.ENSP00000262741	0	0	0	0	0	0.097	0.8	0.052	0.813
FLT4	KITLG	9606.ENSP00000261937	9606.ENSP00000228280	0	0	0	0	0.061	0.087	0.6	0.473	0.795
FLT4	PLCG1	9606.ENSP00000261937	9606.ENSP00000244007	0	0	0	0	0.061	0.374	0.9	0.251	0.95
FLT4	PDGFD	9606.ENSP00000261937	9606.ENSP00000376865	0	0	0	0	0.062	0	0.6	0.295	0.712
FLT4	SHC3	9606.ENSP00000261937	9606.ENSP00000364995	0	0	0	0	0	0.474	0.6	0.05	0.782
FLT4	PDGFA	9606.ENSP00000261937	9606.ENSP00000346508	0	0	0	0	0	0.185	0.6	0.44	0.801
FLT4	PGF	9606.ENSP00000261937	9606.ENSP00000451040	0	0	0	0	0	0.08	0.6	0.777	0.91
FLT4	PDGFB	9606.ENSP00000261937	9606.ENSP00000330382	0	0	0	0	0.061	0.393	0.6	0.66	0.912
FLT4	NRP1	9606.ENSP00000261937	9606.ENSP00000265371	0	0	0	0	0.062	0.732	0	0.7	0.918
FLT4	SRC	9606.ENSP00000261937	9606.ENSP00000362680	0	0	0	0.655	0.058	0.161	0.9	0.689	0.934
FLT4	SHC1	9606.ENSP00000261937	9606.ENSP00000401303	0	0	0	0	0.061	0.474	0.9	0.235	0.957
FLT4	NRP2	9606.ENSP00000261937	9606.ENSP00000353582	0	0	0	0	0.061	0.261	0.9	0.818	0.985
FLT4	VEGFC	9606.ENSP00000261937	9606.ENSP00000480043	0	0	0	0	0.062	0.889	0.9	0.977	0.999
FYN	PLCG1	9606.ENSP00000346671	9606.ENSP00000244007	0	0	0	0.553	0.061	0.472	0.9	0.786	0.964
FYN	RAC2	9606.ENSP00000346671	9606.ENSP00000249071	0	0	0	0	0	0.16	0.8	0.456	0.9
FYN	ITGAV	9606.ENSP00000346671	9606.ENSP00000261023	0	0	0	0	0	0.084	0.9	0.205	0.92
FYN	PIK3R3	9606.ENSP00000346671	9606.ENSP00000262741	0	0	0	0	0.055	0.535	0.9	0.177	0.959
FYN	SHC2	9606.ENSP00000346671	9606.ENSP00000264554	0	0	0	0	0	0.26	0.8	0.158	0.864
FYN	SEMA3A	9606.ENSP00000346671	9606.ENSP00000265362	0	0	0	0	0	0.059	0.9	0.359	0.934
FYN	NRP1	9606.ENSP00000346671	9606.ENSP00000265371	0	0	0	0	0	0	0.9	0.507	0.948
FYN	RAC3	9606.ENSP00000346671	9606.ENSP00000304283	0	0	0	0	0	0.16	0.8	0.372	0.885
FYN	S1PR1	9606.ENSP00000346671	9606.ENSP00000305416	0	0	0	0	0.076	0.166	0.9	0.143	0.925
FYN	PDGFB	9606.ENSP00000346671	9606.ENSP00000330382	0	0	0	0	0	0	0.9	0.146	0.91
FYN	MAPT	9606.ENSP00000346671	9606.ENSP00000340820	0	0	0	0	0	0.379	0	0.75	0.839
FYN	JAK1	9606.ENSP00000346671	9606.ENSP00000343204	0	0	0	0.653	0.059	0.093	0.9	0.427	0.92
FYN	SHC3	9606.ENSP00000346671	9606.ENSP00000364995	0	0	0	0	0.061	0.26	0.8	0.203	0.874
FYN	HCK	9606.ENSP00000346671	9606.ENSP00000444986	0	0	0.444	0.958	0	0	0.9	0.849	0.905
FYN	HLA-DRB	9606.ENSP00000346671	9606.ENSP00000353099	0	0	0	0	0	0	0.9	0.111	0.907
FYN	PIK3R5	9606.ENSP00000346671	9606.ENSP00000392812	0	0	0	0	0.062	0	0.9	0.139	0.912

FYN	PLXNA4	9606.ENSP00000346671	9606.ENSP00000352882	0	0	0	0	0.086	0.085	0.9	0.169	0.921
FYN	NRG1	9606.ENSP00000346671	9606.ENSP00000384620	0	0	0	0	0	0	0.9	0.275	0.924
FYN	PTPN6	9606.ENSP00000346671	9606.ENSP00000391592	0	0	0	0	0.062	0.162	0.9	0.39	0.945
FYN	SYK	9606.ENSP00000346671	9606.ENSP00000364907	0	0	0	0.73	0.061	0.393	0.9	0.814	0.951
FYN	PTK2B	9606.ENSP00000346671	9606.ENSP00000380638	0	0	0	0.714	0.062	0.521	0.9	0.842	0.962
FYN	SHC1	9606.ENSP00000346671	9606.ENSP00000401303	0	0	0	0	0	0.52	0.9	0.658	0.982
FYN	SRC	9606.ENSP00000346671	9606.ENSP00000362680	0	0	0.445	0.971	0	0.981	0.9	0.913	0.998
GAL	OPRD1	9606.ENSP00000265643	9606.ENSP00000234961	0	0	0	0	0	0	0.9	0.473	0.945
GAL	NTS	9606.ENSP00000265643	9606.ENSP00000256010	0	0	0	0	0.166	0	0	0.721	0.757
GAL	KNG1	9606.ENSP00000265643	9606.ENSP00000265023	0	0	0	0	0	0	0.9	0.447	0.942
GAL	VIP	9606.ENSP00000265643	9606.ENSP00000356213	0	0	0	0	0.062	0	0	0.733	0.738
GAL	SAA1	9606.ENSP00000265643	9606.ENSP00000384906	0	0	0	0	0	0	0.9	0	0.9
GAL	S1PR1	9606.ENSP00000265643	9606.ENSP00000305416	0	0	0	0	0	0	0.9	0	0.9
GAL	S1PR2	9606.ENSP00000265643	9606.ENSP00000466933	0	0	0	0	0	0	0.9	0.058	0.901
GAL	SSTR2	9606.ENSP00000265643	9606.ENSP00000350198	0	0	0	0	0	0	0.9	0.352	0.932
GBP2	ICAM1	9606.ENSP00000359497	9606.ENSP00000264832	0	0	0	0	0.149	0	0.9	0.22	0.927
GBP2	VCAM1	9606.ENSP00000359497	9606.ENSP00000294728	0	0	0	0	0.151	0	0.9	0.124	0.919
GBP2	ISG20	9606.ENSP00000359497	9606.ENSP00000306565	0	0	0	0	0.124	0	0.9	0.479	0.95
GBP2	HLA-DQA	9606.ENSP00000359497	9606.ENSP00000339398	0	0	0	0	0.063	0	0.9	0.119	0.91
GBP2	IRF5	9606.ENSP00000359497	9606.ENSP00000349770	0	0	0	0	0.088	0	0.9	0.2	0.92
GBP2	HLA-DRB	9606.ENSP00000359497	9606.ENSP00000353099	0	0	0	0	0.159	0	0.9	0.11	0.918
GBP2	HLA-DRB	9606.ENSP00000359497	9606.ENSP00000364114	0	0	0	0	0.064	0	0.9	0.068	0.905
GBP2	HLA-DPB	9606.ENSP00000359497	9606.ENSP00000408146	0	0	0	0	0.108	0	0.9	0.055	0.908
GBP2	HLA-DQB	9606.ENSP00000359497	9606.ENSP00000364080	0	0	0	0	0.098	0	0.9	0.068	0.908
GBP2	HLA-B	9606.ENSP00000359497	9606.ENSP00000399168	0	0	0	0	0.213	0	0.9	0.046	0.918
GBP2	IFI30	9606.ENSP00000359497	9606.ENSP00000384886	0	0	0	0	0.128	0	0.9	0.161	0.92
GBP2	HLA-DPA	9606.ENSP00000359497	9606.ENSP00000393566	0	0	0	0	0.17	0	0.9	0.161	0.924
GBP2	IFITM1	9606.ENSP00000359497	9606.ENSP00000386187	0	0	0	0	0.137	0	0.9	0.422	0.945
GBP2	OAS1	9606.ENSP00000359497	9606.ENSP00000388001	0	0	0	0	0.154	0	0.9	0.477	0.951
GBP2	IRF9	9606.ENSP00000359497	9606.ENSP00000380073	0	0	0	0	0.204	0	0.9	0.445	0.952
GBP2	PSMB8	9606.ENSP00000359497	9606.ENSP00000364016	0	0	0	0	0.266	0	0.9	0.402	0.952
GBP2	MX1	9606.ENSP00000359497	9606.ENSP00000381601	0	0	0	0	0.245	0	0.9	0.518	0.96
GBP2	IRF7	9606.ENSP00000359497	9606.ENSP00000380697	0	0	0	0	0.221	0.189	0.9	0.518	0.965
GDF15	IL6	9606.ENSP00000252809	9606.ENSP00000385675	0	0	0	0	0	0	0	0.719	0.719
GDNF	PLCG1	9606.ENSP00000409007	9606.ENSP00000244007	0	0	0	0	0	0	0.9	0.073	0.903
GDNF	PIK3R3	9606.ENSP00000409007	9606.ENSP00000262741	0	0	0	0	0	0	0.9	0	0.9
GDNF	NRTN	9606.ENSP00000409007	9606.ENSP00000302648	0	0	0	0.804	0	0	0.9	0.936	0.918
GDNF	NGF	9606.ENSP00000409007	9606.ENSP00000358525	0	0	0	0	0.062	0	0	0.901	0.903
GDNF	SRC	9606.ENSP00000409007	9606.ENSP00000362680	0	0	0	0	0.061	0	0.9	0.41	0.939
GDNF	SHC3	9606.ENSP00000409007	9606.ENSP00000364995	0	0	0	0	0	0	0.9	0.138	0.91
GDNF	SHC1	9606.ENSP00000409007	9606.ENSP00000401303	0	0	0	0	0	0	0.9	0.311	0.928
GDNF	PRKCA	9606.ENSP00000409007	9606.ENSP00000408695	0	0	0	0	0	0	0.9	0.124	0.908
GHR	PLCG1	9606.ENSP00000483403	9606.ENSP00000244007	0	0	0	0	0.061	0.336	0.9	0.088	0.935
GHR	MAPK3	9606.ENSP00000483403	9606.ENSP00000263025	0	0	0	0	0.061	0	0.9	0.227	0.921
GHR	SOCS3	9606.ENSP00000483403	9606.ENSP00000330341	0	0	0	0	0	0.324	0.9	0.644	0.973
GHR	JAK1	9606.ENSP00000483403	9606.ENSP00000343204	0	0	0	0	0	0.437	0.8	0.171	0.898
GHR	PTPN6	9606.ENSP00000483403	9606.ENSP00000391592	0	0	0	0	0	0.336	0.9	0.043	0.93
GIP	PTGER2	9606.ENSP00000350005	9606.ENSP00000245457	0	0	0	0	0	0	0.9	0	0.9
GIP	GLP2R	9606.ENSP00000350005	9606.ENSP00000262441	0	0	0	0	0	0	0.9	0.35	0.932
GIP	GPR32	9606.ENSP00000350005	9606.ENSP00000270590	0	0	0	0	0	0	0.9	0	0.9
GIP	PCSK1	9606.ENSP00000350005	9606.ENSP00000308024	0	0	0	0	0	0	0.9	0.316	0.928
GIP	PTH1R	9606.ENSP00000350005	9606.ENSP00000321999	0	0	0	0	0	0	0.9	0.222	0.918
GIP	VIPR1	9606.ENSP00000350005	9606.ENSP00000327246	0	0	0	0	0	0	0.9	0.311	0.928
GIP	RLN2	9606.ENSP00000350005	9606.ENSP00000371040	0	0	0	0	0	0	0.9	0	0.9
GIP	INSL3	9606.ENSP00000350005	9606.ENSP00000369017	0	0	0	0	0	0	0.9	0	0.9
GIP	MC1R	9606.ENSP00000350005	9606.ENSP00000451605	0	0	0	0	0	0	0.9	0.055	0.901
GIP	PTHLH	9606.ENSP00000350005	9606.ENSP00000441765	0	0	0	0	0	0	0.9	0.215	0.918
GIP	VIP	9606.ENSP00000350005	9606.ENSP00000356213	0	0	0	0	0	0	0.9	0.591	0.957
GLP2R	PTGER2	9606.ENSP00000262441	9606.ENSP00000245457	0	0	0	0	0.061	0	0.9	0	0.902
GLP2R	RLN2	9606.ENSP00000262441	9606.ENSP00000371040	0	0	0	0	0	0	0.9	0	0.9
GLP2R	GPR32	9606.ENSP00000262441	9606.ENSP00000270590	0	0	0	0	0	0	0.9	0	0.9
GLP2R	INSL3	9606.ENSP00000262441	9606.ENSP00000369017	0	0	0	0	0	0	0.9	0	0.9
GLP2R	MC1R	9606.ENSP00000262441	9606.ENSP00000451605	0	0	0	0	0	0	0.9	0.073	0.903
GLP2R	PTHLH	9606.ENSP00000262441	9606.ENSP00000441765	0	0	0	0	0	0	0.9	0.101	0.906
GLP2R	VIPR1	9606.ENSP00000262441	9606.ENSP00000327246	0	0	0	0.839	0.057	0	0.9	0.423	0.907
GLP2R	PTH1R	9606.ENSP00000262441	9606.ENSP00000321999	0	0	0	0.792	0.056	0	0.9	0.473	0.91
GLP2R	VIP	9606.ENSP00000262441	9606.ENSP00000356213	0	0	0	0	0.07	0.176	0.9	0.302	0.939
GPR32	PTGER2	9606.ENSP00000270590	9606.ENSP00000245457	0	0	0	0	0	0	0.9	0.09	0.905
GPR32	PTH1R	9606.ENSP00000270590	9606.ENSP00000321999	0	0	0	0	0	0	0.9	0	0.9
GPR32	MC1R	9606.ENSP00000270590	9606.ENSP00000451605	0	0	0	0	0	0	0.9	0	0.9
GPR32	PTHLH	9606.ENSP00000270590	9606.ENSP00000441765	0	0	0	0	0	0	0.9	0	0.9
GPR32	INSL3	9606.ENSP00000270590	9606.ENSP00000369017	0	0	0	0	0	0	0.9	0	0.9

GPR32	RLN2	9606.ENSP00000270590	9606.ENSP00000371040	0	0	0	0	0	0	0.9	0	0.9
GPR32	VIP	9606.ENSP00000270590	9606.ENSP00000356213	0	0	0	0	0	0	0.9	0.108	0.906
GPR32	VIPR1	9606.ENSP00000270590	9606.ENSP00000327246	0	0	0	0	0	0	0.9	0.123	0.908
GREM1	TGFB2	9606.ENSP00000478319	9606.ENSP00000355896	0	0	0	0	0	0	0	0.707	0.707
HCK	PLCG1	9606.ENSP00000444986	9606.ENSP00000244007	0	0	0	0	0.078	0.728	0.9	0.29	0.979
HCK	TLR2	9606.ENSP00000444986	9606.ENSP00000260010	0	0	0	0	0.576	0.123	0	0.297	0.716
HCK	PIK3R3	9606.ENSP00000444986	9606.ENSP00000262741	0	0	0	0	0	0.535	0.9	0.078	0.953
HCK	SHC2	9606.ENSP00000444986	9606.ENSP00000264554	0	0	0	0	0	0.26	0.6	0.181	0.736
HCK	S1PR1	9606.ENSP00000444986	9606.ENSP00000305416	0	0	0	0	0.078	0.091	0.9	0	0.908
HCK	PDGFB	9606.ENSP00000444986	9606.ENSP00000330382	0	0	0	0	0	0	0.9	0.089	0.905
HCK	JAK1	9606.ENSP00000444986	9606.ENSP00000343204	0	0	0	0.685	0	0	0.9	0.316	0.909
HCK	SRC	9606.ENSP00000444986	9606.ENSP00000362680	0	0	0.444	0.954	0	0.379	0.9	0.792	0.938
HCK	SYK	9606.ENSP00000444986	9606.ENSP00000364907	0	0	0	0.736	0.303	0.064	0.9	0.684	0.941
HCK	SHC3	9606.ENSP00000444986	9606.ENSP00000364995	0	0	0	0	0	0.26	0.6	0.161	0.729
HCK	IL6ST	9606.ENSP00000444986	9606.ENSP00000370698	0	0	0	0	0	0.388	0.9	0.084	0.939
HCK	PTK2B	9606.ENSP00000444986	9606.ENSP00000380638	0	0	0	0.746	0.116	0.125	0.9	0.51	0.926
HCK	ITGB2	9606.ENSP00000444986	9606.ENSP00000380948	0	0	0	0	0.708	0.184	0.9	0.235	0.979
HCK	IL6	9606.ENSP00000444986	9606.ENSP00000385675	0	0	0	0	0.061	0	0.9	0.441	0.942
HCK	SHC1	9606.ENSP00000444986	9606.ENSP00000401303	0	0	0	0	0	0.26	0.6	0.327	0.783
HDAC1	THBS1	9606.ENSP00000362649	9606.ENSP00000260356	0	0	0	0	0	0	0.9	0.19	0.915
HDAC1	RXRA	9606.ENSP00000362649	9606.ENSP00000419692	0	0	0	0	0.145	0.05	0.9	0.14	0.92
HFE	HLA-B	9606.ENSP00000417404	9606.ENSP00000399168	0	0	0	0.852	0	0.689	0	0.518	0.711
HFE	TFR2	9606.ENSP00000417404	9606.ENSP00000420525	0	0	0	0	0.061	0.379	0.72	0.968	0.994
HLA-B	HLA-DOA	9606.ENSP00000399168	9606.ENSP00000229829	0	0	0	0	0	0	0.9	0.584	0.956
HLA-B	ICAM1	9606.ENSP00000399168	9606.ENSP00000264832	0	0	0	0	0.102	0	0.9	0.28	0.929
HLA-B	TAPBPL	9606.ENSP00000399168	9606.ENSP00000266556	0	0	0	0	0.144	0.348	0	0.582	0.746
HLA-B	VCAM1	9606.ENSP00000399168	9606.ENSP00000294728	0	0	0	0	0.057	0	0.9	0.185	0.916
HLA-B	ISG20	9606.ENSP00000399168	9606.ENSP00000306565	0	0	0	0	0.076	0	0.9	0.108	0.91
HLA-B	HLA-DQA	9606.ENSP00000399168	9606.ENSP00000339398	0	0	0	0.596	0	0	0.9	0.737	0.929
HLA-B	IRF5	9606.ENSP00000399168	9606.ENSP00000349770	0	0	0	0	0.098	0	0.9	0.162	0.917
HLA-B	HLA-DRB	9606.ENSP00000399168	9606.ENSP00000353099	0	0	0	0.621	0.163	0	0.9	0.896	0.942
HLA-B	PSMB8	9606.ENSP00000399168	9606.ENSP00000364016	0	0	0	0	0.328	0	0.9	0.559	0.967
HLA-B	HLA-DQB	9606.ENSP00000399168	9606.ENSP00000364080	0	0	0	0.629	0.096	0	0.9	0.847	0.935
HLA-B	HLA-DRB	9606.ENSP00000399168	9606.ENSP00000364114	0	0	0	0.611	0.051	0	0.9	0.655	0.925
HLA-B	IRF9	9606.ENSP00000399168	9606.ENSP00000380073	0	0	0	0	0.107	0	0.9	0.151	0.917
HLA-B	IRF7	9606.ENSP00000399168	9606.ENSP00000380697	0	0	0	0	0.159	0	0.9	0.273	0.933
HLA-B	MX1	9606.ENSP00000399168	9606.ENSP00000381601	0	0	0	0	0.096	0	0.9	0.212	0.922
HLA-B	IFI30	9606.ENSP00000399168	9606.ENSP00000384886	0	0	0	0	0.135	0	0.9	0.154	0.92
HLA-B	IFITM1	9606.ENSP00000399168	9606.ENSP00000386187	0	0	0	0	0.087	0	0.9	0.211	0.921
HLA-B	OAS1	9606.ENSP00000399168	9606.ENSP00000388001	0	0	0	0	0.104	0	0.9	0.193	0.921
HLA-B	HLA-DPA	9606.ENSP00000399168	9606.ENSP00000393566	0	0	0	0.6	0.213	0	0.9	0.674	0.939
HLA-B	HLA-DPB	9606.ENSP00000399168	9606.ENSP00000408146	0	0	0	0.639	0.111	0	0.9	0.799	0.933
HLA-DOA	HLA-DQA	9606.ENSP00000229829	9606.ENSP00000339398	0	0	0	0.954	0.173	0	0.9	0.738	0.916
HLA-DOA	HLA-DPA	9606.ENSP00000229829	9606.ENSP00000393566	0	0	0	0.951	0.181	0	0.9	0.825	0.918
HLA-DOA	HLA-DRB	9606.ENSP00000229829	9606.ENSP00000364114	0	0	0	0.668	0.079	0.195	0.9	0.556	0.933
HLA-DOA	HLA-DRB	9606.ENSP00000229829	9606.ENSP00000353099	0	0	0	0.654	0.174	0.195	0.9	0.666	0.944
HLA-DOA	HLA-DPB	9606.ENSP00000229829	9606.ENSP00000408146	0	0	0	0.686	0.219	0.195	0.9	0.683	0.946
HLA-DOA	HLA-DQB	9606.ENSP00000229829	9606.ENSP00000364080	0	0	0	0.658	0.215	0.195	0.9	0.653	0.946
HLA-DPA	PLCG1	9606.ENSP00000393566	9606.ENSP00000244007	0	0	0	0	0	0	0.9	0	0.9
HLA-DPA	ICAM1	9606.ENSP00000393566	9606.ENSP00000264832	0	0	0	0	0.069	0	0.9	0.162	0.915
HLA-DPA	VCAM1	9606.ENSP00000393566	9606.ENSP00000294728	0	0	0	0	0.07	0	0.9	0.112	0.91
HLA-DPA	HLA-DQA	9606.ENSP00000393566	9606.ENSP00000339398	0	0	0	0.953	0.363	0	0.9	0.832	0.936
HLA-DPA	IRF5	9606.ENSP00000393566	9606.ENSP00000349770	0	0	0	0	0.129	0	0.9	0.085	0.913
HLA-DPA	HLA-DRB	9606.ENSP00000393566	9606.ENSP00000353099	0	0	0	0.648	0.561	0.297	0.9	0.749	0.975
HLA-DPA	HLA-DQB	9606.ENSP00000393566	9606.ENSP00000364080	0	0	0	0.653	0.353	0.297	0.9	0.822	0.964
HLA-DPA	HLA-DRB	9606.ENSP00000393566	9606.ENSP00000364114	0	0	0	0.651	0.205	0.297	0.9	0.644	0.952
HLA-DPA	IRF9	9606.ENSP00000393566	9606.ENSP00000380073	0	0	0	0	0.095	0	0.9	0.129	0.914
HLA-DPA	IRF7	9606.ENSP00000393566	9606.ENSP00000380697	0	0	0	0	0.111	0	0.9	0.119	0.914
HLA-DPA	IFI30	9606.ENSP00000393566	9606.ENSP00000384886	0	0	0	0	0.133	0	0.9	0.081	0.913
HLA-DPA	OAS1	9606.ENSP00000393566	9606.ENSP00000388001	0	0	0	0	0.076	0	0.9	0.107	0.91
HLA-DPA	PTPN6	9606.ENSP00000393566	9606.ENSP00000391592	0	0	0	0	0.139	0	0.9	0.046	0.91
HLA-DPA	HLA-DPB	9606.ENSP00000393566	9606.ENSP00000408146	0	0	0	0.68	0.435	0.899	0.9	0.895	0.995
HLA-DPB	PLCG1	9606.ENSP00000408146	9606.ENSP00000244007	0	0	0	0	0	0	0.9	0	0.9
HLA-DPB	ICAM1	9606.ENSP00000408146	9606.ENSP00000264832	0	0	0	0	0	0	0.9	0.231	0.919
HLA-DPB	VCAM1	9606.ENSP00000408146	9606.ENSP00000294728	0	0	0	0	0.086	0	0.9	0.209	0.921
HLA-DPB	HLA-DQA	9606.ENSP00000408146	9606.ENSP00000339398	0	0	0	0.644	0.174	0.195	0.9	0.897	0.95
HLA-DPB	IRF5	9606.ENSP00000408146	9606.ENSP00000349770	0	0	0	0	0.106	0	0.9	0.29	0.93
HLA-DPB	HLA-DRB	9606.ENSP00000408146	9606.ENSP00000353099	0	0	0	0.962	0.347	0	0.9	0.871	0.934
HLA-DPB	HLA-DQB	9606.ENSP00000408146	9606.ENSP00000364080	0	0	0	0.962	0.273	0	0.9	0.894	0.926
HLA-DPB	HLA-DRB	9606.ENSP00000408146	9606.ENSP00000364114	0	0	0	0.958	0.098	0	0.9	0.677	0.908
HLA-DPB	IRF9	9606.ENSP00000408146	9606.ENSP00000380073	0	0	0	0	0.069	0	0.9	0.08	0.906
HLA-DPB	IRF7	9606.ENSP00000408146	9606.ENSP00000380697	0	0	0	0	0.091	0	0.9	0.09	0.91

HLA-DPB	IFI30	9606.ENSP00000408146	9606.ENSP00000384886	0	0	0	0	0.112	0	0.9	0.15	0.918
HLA-DPB	OAS1	9606.ENSP00000408146	9606.ENSP00000388001	0	0	0	0	0.069	0	0.9	0.055	0.904
HLA-DPB	PTPN6	9606.ENSP00000408146	9606.ENSP00000391592	0	0	0	0	0.096	0	0.9	0.057	0.907
HLA-DQA	PLCG1	9606.ENSP00000339398	9606.ENSP00000244007	0	0	0	0	0	0	0.9	0	0.9
HLA-DQA	ICAM1	9606.ENSP00000339398	9606.ENSP00000264832	0	0	0	0	0.061	0	0.9	0.415	0.94
HLA-DQA	VCAM1	9606.ENSP00000339398	9606.ENSP00000294728	0	0	0	0	0	0	0.9	0.286	0.925
HLA-DQA	PTPN6	9606.ENSP00000339398	9606.ENSP00000391592	0	0	0	0	0.06	0	0.9	0.053	0.903
HLA-DQA	IRF9	9606.ENSP00000339398	9606.ENSP00000380073	0	0	0	0	0.056	0	0.9	0.149	0.912
HLA-DQA	IRF7	9606.ENSP00000339398	9606.ENSP00000380697	0	0	0	0	0.063	0	0.9	0.223	0.92
HLA-DQA	OAS1	9606.ENSP00000339398	9606.ENSP00000388001	0	0	0	0	0.069	0	0.9	0.282	0.927
HLA-DQA	IFI30	9606.ENSP00000339398	9606.ENSP00000384886	0	0	0	0	0.085	0	0.9	0.303	0.93
HLA-DQA	IRF5	9606.ENSP00000339398	9606.ENSP00000349770	0	0	0	0	0.09	0	0.9	0.296	0.93
HLA-DQA	HLA-DRB	9606.ENSP00000339398	9606.ENSP00000364114	0	0	0	0.655	0.193	0.347	0.9	0.715	0.956
HLA-DQA	HLA-DRB	9606.ENSP00000339398	9606.ENSP00000353099	0	0	0	0.645	0.237	0.347	0.9	0.944	0.964
HLA-DQA	HLA-DQB	9606.ENSP00000339398	9606.ENSP00000364080	0	0	0	0.641	0.546	0.938	0.9	0.944	0.998
HLA-DQB	PLCG1	9606.ENSP00000364080	9606.ENSP00000244007	0	0	0	0	0.063	0	0.9	0	0.902
HLA-DQB	ICAM1	9606.ENSP00000364080	9606.ENSP00000264832	0	0	0	0	0	0	0.9	0.257	0.922
HLA-DQB	VCAM1	9606.ENSP00000364080	9606.ENSP00000294728	0	0	0	0	0.097	0	0.9	0.186	0.92
HLA-DQB	IRF5	9606.ENSP00000364080	9606.ENSP00000349770	0	0	0	0	0.119	0	0.9	0.4	0.942
HLA-DQB	HLA-DRB	9606.ENSP00000364080	9606.ENSP00000353099	0	0	0	0.963	0.478	0	0.9	0.932	0.947
HLA-DQB	IRF9	9606.ENSP00000364080	9606.ENSP00000380073	0	0	0	0	0.069	0	0.9	0.046	0.903
HLA-DQB	PTPN6	9606.ENSP00000364080	9606.ENSP00000391592	0	0	0	0	0.121	0	0.9	0.064	0.91
HLA-DQB	IRF7	9606.ENSP00000364080	9606.ENSP00000380697	0	0	0	0	0.085	0	0.9	0.126	0.913
HLA-DQB	OAS1	9606.ENSP00000364080	9606.ENSP00000388001	0	0	0	0	0.076	0	0.9	0.211	0.92
HLA-DQB	IFI30	9606.ENSP00000364080	9606.ENSP00000384886	0	0	0	0	0.119	0	0.9	0.177	0.921
HLA-DQB	HLA-DRB	9606.ENSP00000364080	9606.ENSP00000364114	0	0	0	0.963	0.311	0	0.9	0.769	0.93
HLA-DRB	TNFRSF1(	9606.ENSP00000353099	9606.ENSP00000221132	0	0	0	0	0	0	0	0.74	0.74
HLA-DRB	PLCG1	9606.ENSP00000353099	9606.ENSP00000244007	0	0	0	0	0.063	0	0.9	0	0.902
HLA-DRB	ICAM1	9606.ENSP00000353099	9606.ENSP00000264832	0	0	0	0	0.063	0	0.9	0.252	0.923
HLA-DRB	VCAM1	9606.ENSP00000353099	9606.ENSP00000294728	0	0	0	0	0.097	0	0.9	0.185	0.919
HLA-DRB	IRF5	9606.ENSP00000353099	9606.ENSP00000349770	0	0	0	0	0.145	0	0.9	0.473	0.951
HLA-DRB	SHC1	9606.ENSP00000353099	9606.ENSP00000401303	0	0	0	0	0	0	0.9	0	0.9
HLA-DRB	IRF9	9606.ENSP00000353099	9606.ENSP00000380073	0	0	0	0	0.076	0	0.9	0.07	0.906
HLA-DRB	IRF7	9606.ENSP00000353099	9606.ENSP00000380697	0	0	0	0	0.108	0	0.9	0.138	0.916
HLA-DRB	OAS1	9606.ENSP00000353099	9606.ENSP00000388001	0	0	0	0	0.076	0	0.9	0.198	0.919
HLA-DRB	IFI30	9606.ENSP00000353099	9606.ENSP00000384886	0	0	0	0	0.137	0	0.9	0.163	0.921
HLA-DRB	PTPN6	9606.ENSP00000353099	9606.ENSP00000391592	0	0	0	0	0.173	0	0.9	0.185	0.926
HLA-DRB	HLA-DRB	9606.ENSP00000353099	9606.ENSP00000364114	0	0	0	0.983	0.289	0.748	0.9	0.73	0.98
HLA-DRB	PLCG1	9606.ENSP00000364114	9606.ENSP00000244007	0	0	0	0	0.063	0	0.9	0	0.902
HLA-DRB	ICAM1	9606.ENSP00000364114	9606.ENSP00000264832	0	0	0	0	0	0	0.9	0.271	0.923
HLA-DRB	VCAM1	9606.ENSP00000364114	9606.ENSP00000294728	0	0	0	0	0.097	0	0.9	0.189	0.92
HLA-DRB	IRF5	9606.ENSP00000364114	9606.ENSP00000349770	0	0	0	0	0.062	0	0.9	0.116	0.909
HLA-DRB	IRF9	9606.ENSP00000364114	9606.ENSP00000380073	0	0	0	0	0	0	0.9	0.058	0.901
HLA-DRB	OAS1	9606.ENSP00000364114	9606.ENSP00000388001	0	0	0	0	0	0	0.9	0.064	0.902
HLA-DRB	PTPN6	9606.ENSP00000364114	9606.ENSP00000391592	0	0	0	0	0.085	0	0.9	0	0.904
HLA-DRB	IRF7	9606.ENSP00000364114	9606.ENSP00000380697	0	0	0	0	0.061	0	0.9	0.064	0.904
HLA-DRB	IFI30	9606.ENSP00000364114	9606.ENSP00000384886	0	0	0	0	0.079	0	0.9	0.102	0.91
HNF4A	MAPK3	9606.ENSP00000312987	9606.ENSP00000263025	0	0	0	0	0	0.526	0	0.479	0.742
HSPA1A	TLR2	9606.ENSP00000364802	9606.ENSP00000260010	0	0	0	0	0	0.05	0.8	0.517	0.9
HSPA1A	MAPK3	9606.ENSP00000364802	9606.ENSP00000263025	0	0	0	0	0	0.185	0.9	0.362	0.943
HSPA1A	S100A1	9606.ENSP00000364802	9606.ENSP00000292169	0	0	0	0	0.066	0.734	0	0.237	0.793
HSPA1A	NR3C2	9606.ENSP00000364802	9606.ENSP00000350815	0	0	0	0	0	0.109	0.9	0.253	0.927
HSPA1A	HSPA1B	9606.ENSP00000364802	9606.ENSP00000364801	0	0	0.449	0.988	0.139	0.8	0.9	0.539	0.981
HSPA1A	IL6	9606.ENSP00000364802	9606.ENSP00000385675	0	0	0	0	0	0	0	0.713	0.713
HSPA1A	HSPA1L	9606.ENSP00000364802	9606.ENSP00000364805	0	0	0.449	0.984	0.078	0.387	0.9	0.788	0.939
HSPA1A	HSPA2	9606.ENSP00000364802	9606.ENSP00000378199	0	0	0.449	0.982	0	0.809	0.9	0.626	0.98
HSPA1B	S100A1	9606.ENSP00000364801	9606.ENSP00000292169	0	0	0	0	0.046	0.734	0	0	0.735
HSPA1B	NR3C2	9606.ENSP00000364801	9606.ENSP00000350815	0	0	0	0	0	0.109	0.9	0.118	0.914
HSPA1B	HSPA2	9606.ENSP00000364801	9606.ENSP00000378199	0	0	0.449	0.982	0	0	0.9	0.478	0.901
HSPA1B	HSPA1L	9606.ENSP00000364801	9606.ENSP00000364805	0	0	0.449	0.984	0	0	0.9	0.669	0.901
HSPA1L	TLR2	9606.ENSP00000364805	9606.ENSP00000260010	0	0	0	0	0	0.05	0.8	0.156	0.825
HSPA1L	S100A1	9606.ENSP00000364805	9606.ENSP00000292169	0	0	0	0	0	0.734	0	0.231	0.786
HSPA1L	NR3C2	9606.ENSP00000364805	9606.ENSP00000350815	0	0	0	0	0.059	0.109	0.9	0.118	0.916
HSPA1L	HSPA2	9606.ENSP00000364805	9606.ENSP00000378199	0	0	0.449	0.981	0.061	0	0.9	0.727	0.904
HSPA2	TLR2	9606.ENSP00000378199	9606.ENSP00000260010	0	0	0	0	0	0.05	0.8	0.114	0.817
HSPA2	S100A1	9606.ENSP00000378199	9606.ENSP00000292169	0	0	0	0	0.071	0.527	0	0.406	0.716
HSPA2	NR3C2	9606.ENSP00000378199	9606.ENSP00000350815	0	0	0	0	0	0.109	0.9	0.101	0.912
ICAM1	PLCG1	9606.ENSP00000264832	9606.ENSP00000244007	0	0	0	0	0	0	0.8	0.222	0.837
ICAM1	IL1A	9606.ENSP00000264832	9606.ENSP00000263339	0	0	0	0	0.113	0	0	0.801	0.816
ICAM1	IL1B	9606.ENSP00000264832	9606.ENSP00000263341	0	0	0	0	0.154	0	0	0.916	0.926
ICAM1	PTGS2	9606.ENSP00000264832	9606.ENSP00000356438	0	0	0	0	0.123	0	0	0.677	0.705
ICAM1	SPP1	9606.ENSP00000264832	9606.ENSP00000378517	0	0	0	0	0	0	0	0.719	0.719

ICAM1	SRC	9606.ENSP00000264832	9606.ENSP00000362680	0	0	0	0	0	0	0	0.73	0.731
ICAM1	IL15	9606.ENSP00000264832	9606.ENSP00000296545	0	0	0	0	0.055	0	0	0.743	0.747
ICAM1	IL18	9606.ENSP00000264832	9606.ENSP00000280357	0	0	0	0	0.061	0	0	0.821	0.825
ICAM1	TLR4	9606.ENSP00000264832	9606.ENSP00000363089	0	0	0	0	0.083	0	0	0.829	0.836
ICAM1	OAS1	9606.ENSP00000264832	9606.ENSP00000388001	0	0	0	0	0.082	0	0.9	0.161	0.916
ICAM1	IFI30	9606.ENSP00000264832	9606.ENSP00000384886	0	0	0	0	0.126	0	0.9	0.138	0.918
ICAM1	IRF9	9606.ENSP00000264832	9606.ENSP00000380073	0	0	0	0	0.069	0	0.9	0.231	0.922
ICAM1	IRF5	9606.ENSP00000264832	9606.ENSP00000349770	0	0	0	0	0.071	0	0.9	0.25	0.924
ICAM1	IRF7	9606.ENSP00000264832	9606.ENSP00000380697	0	0	0	0	0.097	0	0.9	0.344	0.935
ICAM1	VCAM1	9606.ENSP00000264832	9606.ENSP00000294728	0	0	0	0	0.069	0	0	0.938	0.94
ICAM1	IL6	9606.ENSP00000264832	9606.ENSP00000385675	0	0	0	0	0.152	0	0	0.947	0.953
ICAM1	ITGB2	9606.ENSP00000264832	9606.ENSP00000380948	0	0	0	0	0.113	0.379	0.9	0.829	0.989
ICAM1	ITGAL	9606.ENSP00000264832	9606.ENSP00000349252	0	0	0	0	0.09	0.887	0.9	0.932	0.999
IFI30	VCAM1	9606.ENSP00000384886	9606.ENSP00000294728	0	0	0	0	0	0	0.9	0.085	0.904
IFI30	IRF5	9606.ENSP00000384886	9606.ENSP00000349770	0	0	0	0	0.153	0	0.9	0.215	0.927
IFI30	IRF9	9606.ENSP00000384886	9606.ENSP00000380073	0	0	0	0	0.078	0	0.9	0.214	0.921
IFI30	IRF7	9606.ENSP00000384886	9606.ENSP00000380697	0	0	0	0	0.096	0	0.9	0.293	0.93
IFI30	OAS1	9606.ENSP00000384886	9606.ENSP00000388001	0	0	0	0	0.139	0	0.9	0.323	0.936
IFIH1	TLR2	9606.ENSP00000263642	9606.ENSP00000260010	0	0	0	0	0.136	0	0	0.71	0.738
IFIH1	TLR4	9606.ENSP00000263642	9606.ENSP00000363089	0	0	0	0	0.096	0	0	0.71	0.726
IFIH1	TLR1	9606.ENSP00000263642	9606.ENSP00000354932	0	0	0	0	0.197	0	0	0.71	0.757
IFIH1	IRF9	9606.ENSP00000263642	9606.ENSP00000380073	0	0	0	0	0.382	0	0	0.651	0.775
IFIH1	OAS1	9606.ENSP00000263642	9606.ENSP00000388001	0	0	0	0	0.644	0	0	0.669	0.877
IFIH1	TLR3	9606.ENSP00000263642	9606.ENSP00000296795	0	0	0	0	0.161	0	0	0.893	0.906
IFIH1	IKBKG	9606.ENSP00000263642	9606.ENSP00000483825	0	0	0	0	0.065	0	0.9	0.475	0.946
IFIH1	MX1	9606.ENSP00000263642	9606.ENSP00000381601	0	0	0	0	0.804	0	0	0.735	0.946
IFIH1	IKBKE	9606.ENSP00000263642	9606.ENSP00000464030	0	0	0	0	0.116	0.435	0.9	0.646	0.979
IFIH1	IRF7	9606.ENSP00000263642	9606.ENSP00000380697	0	0	0	0	0.425	0	0.9	0.893	0.993
IFITM1	ISG20	9606.ENSP00000386187	9606.ENSP00000306565	0	0	0	0	0.314	0	0.9	0.589	0.969
IFITM1	IRF5	9606.ENSP00000386187	9606.ENSP00000349770	0	0	0	0	0	0	0.9	0.303	0.927
IFITM1	PSMB8	9606.ENSP00000386187	9606.ENSP00000364016	0	0	0	0	0.11	0	0.9	0.402	0.942
IFITM1	IRF9	9606.ENSP00000386187	9606.ENSP00000380073	0	0	0	0	0.34	0	0.9	0.567	0.969
IFITM1	IRF7	9606.ENSP00000386187	9606.ENSP00000380697	0	0	0	0	0.331	0	0.9	0.617	0.972
IFITM1	MX1	9606.ENSP00000386187	9606.ENSP00000381601	0	0	0	0	0.746	0	0.9	0.697	0.991
IFITM1	OAS1	9606.ENSP00000386187	9606.ENSP00000388001	0	0	0	0	0.319	0	0.9	0.669	0.975
IFNLR1	JAK1	9606.ENSP00000327824	9606.ENSP00000343204	0	0	0	0	0	0.87	0.9	0.492	0.992
IGF1R	KITLG	9606.ENSP00000268035	9606.ENSP00000228280	0	0	0	0	0	0	0.6	0.39	0.745
IGF1R	PLCG1	9606.ENSP00000268035	9606.ENSP00000244007	0	0	0	0	0.061	0.185	0.8	0.198	0.86
IGF1R	ITGAV	9606.ENSP00000268035	9606.ENSP00000261023	0	0	0	0	0	0.084	0.9	0.18	0.918
IGF1R	PIK3R3	9606.ENSP00000268035	9606.ENSP00000262741	0	0	0	0	0	0.593	0.8	0.904	0.991
IGF1R	MAPK3	9606.ENSP00000268035	9606.ENSP00000263025	0	0	0	0.568	0	0.146	0.9	0.727	0.938
IGF1R	SHC2	9606.ENSP00000268035	9606.ENSP00000264554	0	0	0	0	0	0.26	0.8	0.113	0.857
IGF1R	PGF	9606.ENSP00000268035	9606.ENSP00000451040	0	0	0	0	0	0	0.6	0.287	0.702
IGF1R	TGFA	9606.ENSP00000268035	9606.ENSP00000295400	0	0	0	0	0	0	0.6	0.355	0.73
IGF1R	PDGFA	9606.ENSP00000268035	9606.ENSP00000346508	0	0	0	0	0	0	0.6	0.355	0.73
IGF1R	PDGFB	9606.ENSP00000268035	9606.ENSP00000330382	0	0	0	0	0	0	0.6	0.36	0.733
IGF1R	NGF	9606.ENSP00000268035	9606.ENSP00000358525	0	0	0	0	0	0.185	0.6	0.371	0.777
IGF1R	NEDD4	9606.ENSP00000268035	9606.ENSP00000424827	0	0	0	0	0.061	0.414	0.6	0.371	0.843
IGF1R	VEGFC	9606.ENSP00000268035	9606.ENSP00000480043	0	0	0	0	0	0	0.6	0.626	0.844
IGF1R	SHC3	9606.ENSP00000268035	9606.ENSP00000364995	0	0	0	0	0	0.26	0.8	0.181	0.868
IGF1R	JAK1	9606.ENSP00000268035	9606.ENSP00000343204	0	0	0	0.562	0	0.352	0.8	0.36	0.884
IGF1R	SRC	9606.ENSP00000268035	9606.ENSP00000362680	0	0	0	0.711	0	0.408	0.9	0.835	0.953
IGF1R	SHC1	9606.ENSP00000268035	9606.ENSP00000401303	0	0	0	0	0	0.52	0.9	0.672	0.982
IKBKE	TLR3	9606.ENSP00000464030	9606.ENSP00000296795	0	0	0	0	0.062	0.123	0.9	0.592	0.961
IKBKE	TLR4	9606.ENSP00000464030	9606.ENSP00000363089	0	0	0	0	0.085	0.123	0.9	0.538	0.957
IKBKE	IRF7	9606.ENSP00000464030	9606.ENSP00000380697	0	0	0	0	0.119	0.319	0.9	0.685	0.978
IKBKE	IKBKG	9606.ENSP00000464030	9606.ENSP00000483825	0	0	0	0	0.069	0.435	0.8	0.507	0.941
IKBKE	TNFAIP3	9606.ENSP00000464030	9606.ENSP00000481570	0	0	0	0	0.118	0.406	0.9	0.348	0.961
IKBKG	TNFRSF1/	9606.ENSP00000483825	9606.ENSP00000162749	0	0	0	0	0	0.441	0.9	0.669	0.979
IKBKG	NGFR	9606.ENSP00000483825	9606.ENSP00000172229	0	0	0	0	0	0	0.9	0.185	0.915
IKBKG	TNFRSF1(	9606.ENSP00000483825	9606.ENSP00000221132	0	0	0	0	0	0	0.9	0.302	0.927
IKBKG	RELB	9606.ENSP00000483825	9606.ENSP00000221452	0	0	0	0	0.085	0	0	0.765	0.776
IKBKG	TNFSF10	9606.ENSP00000483825	9606.ENSP00000241261	0	0	0	0	0	0	0.9	0.291	0.926
IKBKG	TLR2	9606.ENSP00000483825	9606.ENSP00000260010	0	0	0	0	0	0.124	0.9	0.562	0.958
IKBKG	MAP3K8	9606.ENSP00000483825	9606.ENSP00000263056	0	0	0	0	0	0	0.9	0.384	0.935
IKBKG	TLR3	9606.ENSP00000483825	9606.ENSP00000296795	0	0	0	0	0	0.764	0.9	0.588	0.989
IKBKG	PRKCB	9606.ENSP00000483825	9606.ENSP00000305355	0	0	0	0	0	0.167	0.9	0.148	0.922
IKBKG	TLR1	9606.ENSP00000483825	9606.ENSP00000354932	0	0	0	0	0	0.124	0.9	0.556	0.957
IKBKG	NGF	9606.ENSP00000483825	9606.ENSP00000358525	0	0	0	0	0	0	0.9	0.194	0.915
IKBKG	TLR4	9606.ENSP00000483825	9606.ENSP00000363089	0	0	0	0	0	0.809	0.9	0.586	0.991
IKBKG	IRF7	9606.ENSP00000483825	9606.ENSP00000380697	0	0	0	0	0.096	0.263	0.8	0.477	0.921
IKBKG	PRKCA	9606.ENSP00000483825	9606.ENSP00000408695	0	0	0	0	0	0	0.9	0.073	0.903

IKBKG	TNFAIP3	9606.ENSP00000483825	9606.ENSP00000481570	0	0	0	0	0.061	0.445	0.9	0.546	0.973
IKBKG	MAP3K14	9606.ENSP00000483825	9606.ENSP00000482657	0	0	0	0	0	0.832	0.9	0.466	0.99
IL11	KITLG	9606.ENSP00000264563	9606.ENSP00000228280	0	0	0	0	0	0	0	0.709	0.709
IL11	LIF	9606.ENSP00000264563	9606.ENSP00000249075	0	0	0	0	0.076	0	0	0.789	0.797
IL11	LEPR	9606.ENSP00000264563	9606.ENSP00000330393	0	0	0	0	0	0.123	0.6	0.24	0.71
IL11	IL13RA1	9606.ENSP00000264563	9606.ENSP00000360730	0	0	0	0	0	0	0.6	0.539	0.807
IL11	OSMR	9606.ENSP00000264563	9606.ENSP00000274276	0	0	0	0	0.069	0	0.6	0.55	0.817
IL11	IL6	9606.ENSP00000264563	9606.ENSP00000385675	0	0	0	0	0.067	0	0	0.883	0.887
IL11	JAK1	9606.ENSP00000264563	9606.ENSP00000343204	0	0	0	0	0	0	0.9	0.53	0.95
IL11	IL6ST	9606.ENSP00000264563	9606.ENSP00000370698	0	0	0	0	0	0.472	0.9	0.646	0.979
IL11	IL11RA	9606.ENSP00000264563	9606.ENSP00000450565	0	0	0	0	0	0.472	0.9	0.72	0.983
IL11RA	JAK1	9606.ENSP00000450565	9606.ENSP00000343204	0	0	0	0	0	0	0.9	0.209	0.917
IL11RA	IL6ST	9606.ENSP00000450565	9606.ENSP00000370698	0	0	0	0.562	0.059	0.299	0.9	0.572	0.945
IL11RA	IL6	9606.ENSP00000450565	9606.ENSP00000385675	0	0	0	0	0	0	0.6	0.349	0.728
IL13RA1	IL15	9606.ENSP00000360730	9606.ENSP00000296545	0	0	0	0	0.062	0	0.6	0.341	0.731
IL13RA1	JAK1	9606.ENSP00000360730	9606.ENSP00000343204	0	0	0	0	0.051	0	0.9	0.577	0.956
IL13RA1	IL6	9606.ENSP00000360730	9606.ENSP00000385675	0	0	0	0	0.062	0	0.6	0.31	0.718
IL15	IL9R	9606.ENSP00000296545	9606.ENSP00000244174	0	0	0	0	0.08	0	0.6	0.359	0.743
IL15	IL1B	9606.ENSP00000296545	9606.ENSP00000263341	0	0	0	0	0.063	0	0	0.794	0.799
IL15	IL18	9606.ENSP00000296545	9606.ENSP00000280357	0	0	0	0	0.061	0	0	0.897	0.9
IL15	TSLP	9606.ENSP00000296545	9606.ENSP00000339804	0	0	0	0	0	0	0	0.726	0.726
IL15	TNFRSF9	9606.ENSP00000296545	9606.ENSP00000478699	0	0	0	0	0.064	0	0	0.804	0.809
IL15	IL21R	9606.ENSP00000296545	9606.ENSP00000338010	0	0	0	0	0.08	0	0.6	0.615	0.846
IL15	IL6	9606.ENSP00000296545	9606.ENSP00000385675	0	0	0	0	0.062	0	0	0.88	0.883
IL15	SHC1	9606.ENSP00000296545	9606.ENSP00000401303	0	0	0	0	0	0	0.9	0.283	0.925
IL15	IL7R	9606.ENSP00000296545	9606.ENSP00000306157	0	0	0	0	0.11	0	0.6	0.812	0.927
IL15	JAK1	9606.ENSP00000296545	9606.ENSP00000343204	0	0	0	0	0.061	0	0.9	0.592	0.958
IL18	TLR2	9606.ENSP00000280357	9606.ENSP00000260010	0	0	0	0	0.069	0	0	0.763	0.77
IL18	MAPK3	9606.ENSP00000280357	9606.ENSP00000263025	0	0	0	0	0	0	0.9	0.477	0.945
IL18	IL1A	9606.ENSP00000280357	9606.ENSP00000263339	0	0	0	0	0.061	0	0.9	0.668	0.966
IL18	IL1B	9606.ENSP00000280357	9606.ENSP00000263341	0	0	0	0	0.095	0	0.9	0.921	0.992
IL18	TLR1	9606.ENSP00000280357	9606.ENSP00000354932	0	0	0	0	0.078	0	0	0.71	0.721
IL18	IL32	9606.ENSP00000280357	9606.ENSP00000432218	0	0	0	0	0	0	0	0.722	0.722
IL18	IL1R1	9606.ENSP00000280357	9606.ENSP00000386380	0	0	0	0	0	0	0	0.732	0.732
IL18	TLR3	9606.ENSP00000280357	9606.ENSP00000296795	0	0	0	0	0.061	0	0	0.732	0.737
IL18	LCN2	9606.ENSP00000280357	9606.ENSP00000362108	0	0	0	0	0.076	0	0	0.731	0.74
IL18	VCAM1	9606.ENSP00000280357	9606.ENSP00000294728	0	0	0	0	0.061	0	0	0.757	0.763
IL18	IL33	9606.ENSP00000280357	9606.ENSP00000370842	0	0	0	0	0.061	0	0	0.868	0.871
IL18	IL6	9606.ENSP00000280357	9606.ENSP00000385675	0	0	0	0	0	0	0	0.872	0.872
IL18	TLR4	9606.ENSP00000280357	9606.ENSP00000363089	0	0	0	0	0.062	0	0	0.87	0.873
IL1A	TLR2	9606.ENSP00000263339	9606.ENSP00000260010	0	0	0	0	0.084	0	0	0.706	0.72
IL1A	VCAM1	9606.ENSP00000263339	9606.ENSP00000294728	0	0	0	0	0.061	0	0	0.735	0.741
IL1A	PTGS2	9606.ENSP00000263339	9606.ENSP00000356438	0	0	0	0	0.213	0	0	0.772	0.813
IL1A	IL6	9606.ENSP00000263339	9606.ENSP00000385675	0	0	0	0	0.375	0	0	0.918	0.946
IL1A	IL1R2	9606.ENSP00000263339	9606.ENSP00000330959	0	0	0	0	0.067	0.544	0.9	0.559	0.978
IL1A	IL1R1	9606.ENSP00000263339	9606.ENSP00000386380	0	0	0	0	0.061	0.457	0.9	0.826	0.989
IL1A	IL1B	9606.ENSP00000263339	9606.ENSP00000263341	0	0	0	0	0.753	0	0.9	0.921	0.997
IL1B	LIF	9606.ENSP00000263341	9606.ENSP00000249075	0	0	0	0	0.069	0	0	0.695	0.703
IL1B	TLR2	9606.ENSP00000263341	9606.ENSP00000260010	0	0	0	0	0.279	0	0	0.802	0.851
IL1B	IL32	9606.ENSP00000263341	9606.ENSP00000432218	0	0	0	0	0.065	0	0	0.694	0.702
IL1B	SPP1	9606.ENSP00000263341	9606.ENSP00000378517	0	0	0	0	0.061	0	0	0.708	0.714
IL1B	TLR3	9606.ENSP00000263341	9606.ENSP00000296795	0	0	0	0	0.061	0	0	0.711	0.717
IL1B	LCN2	9606.ENSP00000263341	9606.ENSP00000362108	0	0	0	0	0.097	0	0	0.707	0.724
IL1B	TLR1	9606.ENSP00000263341	9606.ENSP00000354932	0	0	0	0	0.226	0	0	0.672	0.736
IL1B	SOCS3	9606.ENSP00000263341	9606.ENSP00000330341	0	0	0	0	0.219	0	0	0.733	0.783
IL1B	NGF	9606.ENSP00000263341	9606.ENSP00000358525	0	0	0	0	0	0	0	0.787	0.787
IL1B	IL33	9606.ENSP00000263341	9606.ENSP00000370842	0	0	0	0	0	0	0	0.81	0.81
IL1B	NOS2	9606.ENSP00000263341	9606.ENSP00000327251	0	0	0	0	0.066	0	0	0.845	0.85
IL1B	VCAM1	9606.ENSP00000263341	9606.ENSP00000294728	0	0	0	0	0.062	0	0	0.865	0.868
IL1B	TLR4	9606.ENSP00000263341	9606.ENSP00000363089	0	0	0	0	0.16	0	0	0.913	0.925
IL1B	PTGS2	9606.ENSP00000263341	9606.ENSP00000356438	0	0	0	0	0.561	0	0	0.943	0.974
IL1B	IL6	9606.ENSP00000263341	9606.ENSP00000385675	0	0	0	0	0.43	0	0	0.973	0.984
IL1B	IL1R2	9606.ENSP00000263341	9606.ENSP00000330959	0	0	0	0	0.197	0.872	0.9	0.791	0.997
IL1B	IL1R1	9606.ENSP00000263341	9606.ENSP00000386380	0	0	0	0	0.076	0.875	0.9	0.893	0.998
IL1R1	TNFRSF1A	9606.ENSP00000386380	9606.ENSP00000162749	0	0	0	0	0.082	0	0	0.727	0.739
IL1R1	TLR2	9606.ENSP00000386380	9606.ENSP00000260010	0	0	0	0	0.084	0	0	0.79	0.799
IL1R1	TLR3	9606.ENSP00000386380	9606.ENSP00000296795	0	0	0	0	0	0	0	0.734	0.734
IL1R1	IL1R2	9606.ENSP00000386380	9606.ENSP00000330959	0	0	0	0.682	0.114	0	0.8	0.754	0.859
IL1R1	TLR1	9606.ENSP00000386380	9606.ENSP00000354932	0	0	0	0	0.069	0	0	0.709	0.717
IL1R1	TLR4	9606.ENSP00000386380	9606.ENSP00000363089	0	0	0	0	0.083	0	0	0.798	0.807
IL1R1	IL6	9606.ENSP00000386380	9606.ENSP00000385675	0	0	0	0	0.098	0	0	0.835	0.846
IL20RB	IL22RA1	9606.ENSP00000328133	9606.ENSP00000270800	0	0	0	0	0	0.32	0.9	0.8	0.985

IL20RB	JAK1	9606.ENSP00000328133	9606.ENSP00000343204	0	0	0	0	0	0	0.9	0.222	0.918
IL21R	IL6	9606.ENSP00000338010	9606.ENSP00000385675	0	0	0	0	0	0	0.6	0.519	0.799
IL21R	JAK1	9606.ENSP00000338010	9606.ENSP00000343204	0	0	0	0	0.062	0.379	0.9	0.441	0.963
IL22RA1	TSLP	9606.ENSP00000270800	9606.ENSP00000339804	0	0	0	0	0	0	0.6	0.291	0.704
IL22RA1	IL6	9606.ENSP00000270800	9606.ENSP00000385675	0	0	0	0	0.06	0	0.6	0.473	0.784
IL22RA1	JAK1	9606.ENSP00000270800	9606.ENSP00000343204	0	0	0	0	0	0	0.9	0.455	0.943
IL31RA	OSMR	9606.ENSP00000415900	9606.ENSP00000274276	0	0	0	0.566	0.062	0	0.9	0.76	0.934
IL31RA	JAK1	9606.ENSP00000415900	9606.ENSP00000343204	0	0	0	0	0	0	0.9	0.369	0.934
IL33	TLR2	9606.ENSP00000370842	9606.ENSP00000260010	0	0	0	0	0.061	0	0	0.754	0.76
IL33	TLR3	9606.ENSP00000370842	9606.ENSP00000296795	0	0	0	0	0.061	0	0	0.743	0.748
IL33	TSLP	9606.ENSP00000370842	9606.ENSP00000339804	0	0	0	0	0	0	0	0.934	0.934
IL33	TLR4	9606.ENSP00000370842	9606.ENSP00000363089	0	0	0	0	0.083	0	0	0.787	0.796
IL33	IL6	9606.ENSP00000370842	9606.ENSP00000385675	0	0	0	0	0	0	0	0.832	0.832
IL6	TNFRSF1	9606.ENSP00000385675	9606.ENSP00000162749	0	0	0	0	0	0	0	0.84	0.84
IL6	KITLG	9606.ENSP00000385675	9606.ENSP00000228280	0	0	0	0	0	0	0	0.768	0.768
IL6	PROC	9606.ENSP00000385675	9606.ENSP00000234071	0	0	0	0	0	0	0.9	0.238	0.92
IL6	LIF	9606.ENSP00000385675	9606.ENSP00000249075	0	0	0	0	0.178	0	0	0.768	0.801
IL6	TLR2	9606.ENSP00000385675	9606.ENSP00000260010	0	0	0	0	0.064	0	0	0.942	0.944
IL6	MAPK3	9606.ENSP00000385675	9606.ENSP00000263025	0	0	0	0	0	0	0.9	0.771	0.976
IL6	WNT5A	9606.ENSP00000385675	9606.ENSP00000264634	0	0	0	0	0.076	0	0	0.733	0.743
IL6	KNG1	9606.ENSP00000385675	9606.ENSP00000265023	0	0	0	0	0	0	0.9	0.754	0.974
IL6	OSMR	9606.ENSP00000385675	9606.ENSP00000274276	0	0	0	0	0.095	0	0.6	0.529	0.814
IL6	VCAM1	9606.ENSP00000385675	9606.ENSP00000294728	0	0	0	0	0.083	0	0	0.891	0.896
IL6	TLR3	9606.ENSP00000385675	9606.ENSP00000296795	0	0	0	0	0	0	0	0.901	0.902
IL6	SCG2	9606.ENSP00000385675	9606.ENSP00000304133	0	0	0	0	0	0	0.9	0.124	0.908
IL6	IL7R	9606.ENSP00000385675	9606.ENSP00000306157	0	0	0	0	0.076	0	0.6	0.724	0.889
IL6	SDC2	9606.ENSP00000385675	9606.ENSP00000307046	0	0	0	0	0	0	0.9	0.106	0.906
IL6	NOS2	9606.ENSP00000385675	9606.ENSP00000327251	0	0	0	0	0.063	0	0	0.735	0.741
IL6	SOCS3	9606.ENSP00000385675	9606.ENSP00000330341	0	0	0	0	0.207	0	0.9	0.917	0.992
IL6	LEPR	9606.ENSP00000385675	9606.ENSP00000330393	0	0	0	0	0	0.064	0.6	0.599	0.836
IL6	JAK1	9606.ENSP00000385675	9606.ENSP00000343204	0	0	0	0	0.061	0	0.9	0.696	0.968
IL6	TLR1	9606.ENSP00000385675	9606.ENSP00000354932	0	0	0	0	0	0	0	0.801	0.801
IL6	PTGS2	9606.ENSP00000385675	9606.ENSP00000356438	0	0	0	0	0.314	0	0	0.922	0.945
IL6	SRC	9606.ENSP00000385675	9606.ENSP00000362680	0	0	0	0	0	0	0	0.793	0.793
IL6	TLR4	9606.ENSP00000385675	9606.ENSP00000363089	0	0	0	0	0.082	0	0	0.956	0.959
IL6	SDC1	9606.ENSP00000385675	9606.ENSP00000370542	0	0	0	0	0	0	0	0.778	0.778
IL6	IL6ST	9606.ENSP00000385675	9606.ENSP00000370698	0	0	0	0	0.052	0.875	0.9	0.774	0.997
IL6	SPP1	9606.ENSP00000385675	9606.ENSP00000378517	0	0	0	0	0.06	0	0.9	0.793	0.978
IL6	IRF7	9606.ENSP00000385675	9606.ENSP00000380697	0	0	0	0	0.063	0	0	0.79	0.795
IL6	SAA1	9606.ENSP00000385675	9606.ENSP00000384906	0	0	0	0	0.097	0	0	0.793	0.805
IL6	TNFRSF9	9606.ENSP00000385675	9606.ENSP00000478699	0	0	0	0	0.061	0	0	0.7	0.706
IL6	PGF	9606.ENSP00000385675	9606.ENSP00000451040	0	0	0	0	0	0	0	0.738	0.738
IL6	VEGFC	9606.ENSP00000385675	9606.ENSP00000480043	0	0	0	0	0.111	0	0	0.74	0.759
IL6	PTHLH	9606.ENSP00000385675	9606.ENSP00000441765	0	0	0	0	0.065	0.379	0	0.73	0.83
IL6	SHC1	9606.ENSP00000385675	9606.ENSP00000401303	0	0	0	0	0	0	0.9	0.322	0.929
IL6ST	LIF	9606.ENSP00000370698	9606.ENSP00000249075	0	0	0	0	0	0.87	0.9	0.527	0.993
IL6ST	OSMR	9606.ENSP00000370698	9606.ENSP00000274276	0	0	0	0.567	0.095	0.408	0.9	0.622	0.957
IL6ST	SOCS3	9606.ENSP00000370698	9606.ENSP00000330341	0	0	0	0	0.065	0.472	0.9	0.433	0.968
IL6ST	JAK1	9606.ENSP00000370698	9606.ENSP00000343204	0	0	0	0	0.09	0.421	0.9	0.556	0.973
IL7R	PIK3R3	9606.ENSP00000306157	9606.ENSP00000262741	0	0	0	0	0	0	0.9	0	0.9
IL7R	WNT5A	9606.ENSP00000306157	9606.ENSP00000264634	0	0	0	0	0	0	0.9	0.058	0.901
IL7R	JAK1	9606.ENSP00000306157	9606.ENSP00000343204	0	0	0	0	0.06	0.472	0.9	0.389	0.965
IL7R	TSLP	9606.ENSP00000306157	9606.ENSP00000339804	0	0	0	0	0	0.899	0.9	0.623	0.995
IL9R	TSLP	9606.ENSP00000244174	9606.ENSP00000339804	0	0	0	0	0	0	0.6	0.402	0.75
IL9R	JAK1	9606.ENSP00000244174	9606.ENSP00000343204	0	0	0	0	0	0.379	0.9	0.354	0.956
INPP5D	PLCG1	9606.ENSP00000405338	9606.ENSP00000244007	0	0	0	0	0.064	0.46	0	0.58	0.77
INPP5D	PRKCB	9606.ENSP00000405338	9606.ENSP00000305355	0	0	0	0	0.098	0	0.9	0.109	0.912
INPP5D	MET	9606.ENSP00000405338	9606.ENSP00000317272	0	0	0	0	0	0.4	0.9	0.119	0.942
INPP5D	JAK1	9606.ENSP00000405338	9606.ENSP00000343204	0	0	0	0	0	0.525	0.9	0.164	0.956
INPP5D	PTPN6	9606.ENSP00000405338	9606.ENSP00000391592	0	0	0	0	0.159	0.067	0.9	0.5	0.955
INPP5D	SHC1	9606.ENSP00000405338	9606.ENSP00000401303	0	0	0	0	0.063	0.52	0.9	0.533	0.976
INSL3	INSL4	9606.ENSP00000369017	9606.ENSP00000239316	0	0	0	0	0	0	0	0.745	0.745
INSL3	PTGER2	9606.ENSP00000369017	9606.ENSP00000245457	0	0	0	0	0	0	0.9	0	0.9
INSL3	PTH1R	9606.ENSP00000369017	9606.ENSP00000321999	0	0	0	0	0	0	0.9	0	0.9
INSL3	VIPR1	9606.ENSP00000369017	9606.ENSP00000327246	0	0	0	0	0	0	0.9	0	0.9
INSL3	VIP	9606.ENSP00000369017	9606.ENSP00000356213	0	0	0	0	0.049	0	0.9	0.041	0.9
INSL3	MC1R	9606.ENSP00000369017	9606.ENSP00000451605	0	0	0	0	0	0	0.9	0	0.9
INSL3	PTHLH	9606.ENSP00000369017	9606.ENSP00000441765	0	0	0	0	0	0	0.9	0.041	0.9
INSL3	RLN2	9606.ENSP00000369017	9606.ENSP00000371040	0	0	0	0	0	0	0.9	0.672	0.965
INSL4	RLN2	9606.ENSP00000239316	9606.ENSP00000371040	0	0	0	0	0	0	0	0.725	0.725
IRF5	VCAM1	9606.ENSP00000349770	9606.ENSP00000294728	0	0	0	0	0	0	0.9	0.197	0.916
IRF5	ISG20	9606.ENSP00000349770	9606.ENSP00000306565	0	0	0	0	0.07	0	0.9	0.216	0.92

IRF5	PSMB8	9606.ENSP00000349770	9606.ENSP00000364016	0	0	0	0	0.091	0	0.9	0.123	0.913
IRF5	IRF9	9606.ENSP00000349770	9606.ENSP00000380073	0	0	0	0.693	0.069	0	0.9	0.628	0.921
IRF5	IRF7	9606.ENSP00000349770	9606.ENSP00000380697	0	0	0	0.678	0.116	0	0.9	0.83	0.932
IRF5	OAS1	9606.ENSP00000349770	9606.ENSP00000388001	0	0	0	0	0.088	0	0.9	0.441	0.944
IRF5	MX1	9606.ENSP00000349770	9606.ENSP00000381601	0	0	0	0	0.089	0	0.9	0.558	0.956
IRF7	TLR2	9606.ENSP00000380697	9606.ENSP00000260010	0	0	0	0	0.117	0	0	0.702	0.726
IRF7	VCAM1	9606.ENSP00000380697	9606.ENSP00000294728	0	0	0	0	0.062	0	0.9	0.213	0.919
IRF7	TLR3	9606.ENSP00000380697	9606.ENSP00000296795	0	0	0	0	0.064	0	0.9	0.841	0.983
IRF7	ISG20	9606.ENSP00000380697	9606.ENSP00000306565	0	0	0	0	0.314	0	0.9	0.652	0.974
IRF7	TLR4	9606.ENSP00000380697	9606.ENSP00000363089	0	0	0	0	0.084	0	0.9	0.708	0.97
IRF7	PSMB8	9606.ENSP00000380697	9606.ENSP00000364016	0	0	0	0	0.236	0	0.9	0.294	0.941
IRF7	SPP1	9606.ENSP00000380697	9606.ENSP00000378517	0	0	0	0	0	0	0.8	0.286	0.851
IRF7	IRF9	9606.ENSP00000380697	9606.ENSP00000380073	0	0	0	0.633	0.651	0	0.9	0.87	0.975
IRF7	OAS1	9606.ENSP00000380697	9606.ENSP00000388001	0	0	0	0	0.688	0	0.9	0.678	0.989
IRF7	MX1	9606.ENSP00000380697	9606.ENSP00000381601	0	0	0	0	0.816	0	0.9	0.797	0.995
IRF9	VCAM1	9606.ENSP00000380073	9606.ENSP00000294728	0	0	0	0	0	0	0.9	0.081	0.904
IRF9	ISG20	9606.ENSP00000380073	9606.ENSP00000306565	0	0	0	0	0.151	0	0.9	0.53	0.956
IRF9	JAK1	9606.ENSP00000380073	9606.ENSP00000343204	0	0	0	0	0	0	0.9	0.766	0.975
IRF9	PSMB8	9606.ENSP00000380073	9606.ENSP00000364016	0	0	0	0	0.141	0	0.9	0.413	0.945
IRF9	OAS1	9606.ENSP00000380073	9606.ENSP00000388001	0	0	0	0	0.559	0	0.9	0.67	0.984
IRF9	MX1	9606.ENSP00000380073	9606.ENSP00000381601	0	0	0	0	0.786	0	0.9	0.711	0.993
ISG20	PSMB8	9606.ENSP00000306565	9606.ENSP00000364016	0	0	0	0	0.097	0	0.9	0.228	0.924
ISG20	OAS1	9606.ENSP00000306565	9606.ENSP00000388001	0	0	0	0	0.229	0	0.9	0.668	0.972
ISG20	MX1	9606.ENSP00000306565	9606.ENSP00000381601	0	0	0	0	0.328	0	0.9	0.676	0.976
ITGAL	ITGAV	9606.ENSP00000349252	9606.ENSP00000261023	0	0	0	0.56	0.054	0	0.9	0.492	0.921
ITGAL	OLR1	9606.ENSP00000349252	9606.ENSP00000309124	0	0	0	0	0.061	0	0.9	0.087	0.906
ITGAL	PLAUR	9606.ENSP00000349252	9606.ENSP00000339328	0	0	0	0	0.061	0	0.9	0.187	0.916
ITGAL	PTK2B	9606.ENSP00000349252	9606.ENSP00000380638	0	0	0	0	0.107	0	0.8	0.211	0.846
ITGAL	PLAU	9606.ENSP00000349252	9606.ENSP00000361850	0	0	0	0	0	0	0.9	0.089	0.905
ITGAL	ITGB2	9606.ENSP00000349252	9606.ENSP00000380948	0	0	0	0	0.418	0.892	0.9	0.866	0.999
ITGAV	VTN	9606.ENSP00000261023	9606.ENSP00000226218	0	0	0	0	0	0.336	0.9	0.869	0.99
ITGAV	TGFB3	9606.ENSP00000261023	9606.ENSP00000238682	0	0	0	0	0.049	0.405	0.9	0.235	0.95
ITGAV	THBS1	9606.ENSP00000261023	9606.ENSP00000260356	0	0	0	0	0.079	0.462	0.6	0.409	0.867
ITGAV	MAP3K14	9606.ENSP00000261023	9606.ENSP00000482657	0	0	0	0	0	0	0.9	0.048	0.9
ITGAV	OLR1	9606.ENSP00000261023	9606.ENSP00000309124	0	0	0	0	0	0	0.9	0.11	0.907
ITGAV	PTK2B	9606.ENSP00000261023	9606.ENSP00000380638	0	0	0	0	0.061	0	0.9	0.155	0.913
ITGAV	PDGFB	9606.ENSP00000261023	9606.ENSP00000330382	0	0	0	0	0.061	0	0.9	0.189	0.917
ITGAV	SDC1	9606.ENSP00000261023	9606.ENSP00000370542	0	0	0	0	0.076	0	0.9	0.189	0.918
ITGAV	SDC4	9606.ENSP00000261023	9606.ENSP00000361818	0	0	0	0	0.076	0	0.9	0.19	0.918
ITGAV	TGFBR2	9606.ENSP00000261023	9606.ENSP00000351905	0	0	0	0	0.078	0.084	0.9	0.251	0.928
ITGAV	SYK	9606.ENSP00000261023	9606.ENSP00000364907	0	0	0	0	0	0.161	0.9	0.222	0.929
ITGAV	PLAU	9606.ENSP00000261023	9606.ENSP00000361850	0	0	0	0	0.07	0	0.9	0.322	0.931
ITGAV	PLAUR	9606.ENSP00000261023	9606.ENSP00000339328	0	0	0	0	0	0	0.9	0.42	0.939
ITGAV	SRC	9606.ENSP00000261023	9606.ENSP00000362680	0	0	0	0	0	0.084	0.9	0.426	0.942
ITGAV	MAPK3	9606.ENSP00000261023	9606.ENSP00000263025	0	0	0	0	0.062	0.32	0.9	0.278	0.947
ITGAV	SPP1	9606.ENSP00000261023	9606.ENSP00000378517	0	0	0	0	0.078	0.379	0.9	0.555	0.971
ITGAV	ITGB2	9606.ENSP00000261023	9606.ENSP00000380948	0	0	0	0	0.062	0.382	0.9	0.56	0.971
ITGB2	PROC	9606.ENSP00000380948	9606.ENSP00000234071	0	0	0	0	0.081	0	0.9	0	0.904
ITGB2	RAC2	9606.ENSP00000380948	9606.ENSP00000249071	0	0	0	0	0.641	0.069	0	0.434	0.794
ITGB2	TLR2	9606.ENSP00000380948	9606.ENSP00000260010	0	0	0	0	0.552	0.062	0	0.353	0.704
ITGB2	KNG1	9606.ENSP00000380948	9606.ENSP00000265023	0	0	0	0	0.061	0.379	0.9	0.123	0.942
ITGB2	VCAM1	9606.ENSP00000380948	9606.ENSP00000294728	0	0	0	0	0.444	0	0.9	0.63	0.977
ITGB2	OLR1	9606.ENSP00000380948	9606.ENSP00000309124	0	0	0	0	0.052	0	0.9	0.211	0.918
ITGB2	PLAUR	9606.ENSP00000380948	9606.ENSP00000339328	0	0	0	0	0.073	0.379	0.9	0.153	0.944
ITGB2	PLAU	9606.ENSP00000380948	9606.ENSP00000361850	0	0	0	0	0.063	0	0.9	0.198	0.918
ITGB2	SRC	9606.ENSP00000380948	9606.ENSP00000362680	0	0	0	0	0.063	0.093	0.6	0.321	0.738
ITGB2	SYK	9606.ENSP00000380948	9606.ENSP00000364907	0	0	0	0	0.34	0.404	0	0.377	0.734
ITGB2	PTK2B	9606.ENSP00000380948	9606.ENSP00000380638	0	0	0	0	0.216	0.522	0.8	0.288	0.939
JAG1	JAG2	9606.ENSP00000254958	9606.ENSP00000328169	0	0	0	0.951	0.062	0	0.9	0.942	0.906
JAK1	TNFRSF1/	9606.ENSP00000343204	9606.ENSP00000162749	0	0	0	0	0	0.384	0	0.594	0.739
JAK1	PLCG1	9606.ENSP00000343204	9606.ENSP00000244007	0	0	0	0	0.061	0.422	0.9	0.252	0.953
JAK1	LIF	9606.ENSP00000343204	9606.ENSP00000249075	0	0	0	0	0	0	0.9	0.53	0.95
JAK1	PIK3R3	9606.ENSP00000343204	9606.ENSP00000262741	0	0	0	0	0	0.087	0.9	0.136	0.914
JAK1	MAPK3	9606.ENSP00000343204	9606.ENSP00000263025	0	0	0	0.6	0.113	0.146	0.9	0.601	0.936
JAK1	OSMR	9606.ENSP00000343204	9606.ENSP00000274276	0	0	0	0	0	0.379	0.9	0.444	0.962
JAK1	PRKCB	9606.ENSP00000343204	9606.ENSP00000305355	0	0	0	0.55	0	0.073	0.9	0.125	0.907
JAK1	MET	9606.ENSP00000343204	9606.ENSP00000317272	0	0	0	0.567	0.063	0.085	0.8	0.31	0.836
JAK1	SOCS3	9606.ENSP00000343204	9606.ENSP00000330341	0	0	0	0	0.082	0.457	0.9	0.709	0.983
JAK1	LEPR	9606.ENSP00000343204	9606.ENSP00000330393	0	0	0	0	0	0.132	0.6	0.275	0.726
JAK1	SRC	9606.ENSP00000343204	9606.ENSP00000362680	0	0	0	0.664	0.059	0.093	0.8	0.683	0.856
JAK1	PDGFA	9606.ENSP00000343204	9606.ENSP00000346508	0	0	0	0	0	0	0.9	0.079	0.903
JAK1	SYK	9606.ENSP00000343204	9606.ENSP00000364907	0	0	0	0.643	0	0.321	0.9	0.491	0.941

JAK1	PTK2B	9606.ENSP00000343204	9606.ENSP00000380638	0	0	0	0.582	0.061	0.433	0.9	0.348	0.949
JAK1	SHC1	9606.ENSP00000343204	9606.ENSP00000401303	0	0	0	0	0.061	0.26	0.9	0.43	0.955
JAK1	MX1	9606.ENSP00000343204	9606.ENSP00000381601	0	0	0	0	0	0.064	0.9	0.565	0.955
JAK1	PTPN6	9606.ENSP00000343204	9606.ENSP00000391592	0	0	0	0	0.062	0.457	0.9	0.427	0.966
KITLG	NGFR	9606.ENSP00000228280	9606.ENSP00000172229	0	0	0	0	0	0	0.6	0.324	0.718
KITLG	MET	9606.ENSP00000228280	9606.ENSP00000317272	0	0	0	0	0.1	0	0.6	0.417	0.771
KITLG	PTPN6	9606.ENSP00000228280	9606.ENSP00000391592	0	0	0	0	0.054	0	0.9	0.163	0.913
KITLG	SHC1	9606.ENSP00000228280	9606.ENSP00000401303	0	0	0	0	0	0	0.9	0.325	0.929
KNG1	VTN	9606.ENSP00000265023	9606.ENSP00000226218	0	0	0	0	0.147	0.379	0	0.512	0.719
KNG1	PROC	9606.ENSP00000265023	9606.ENSP00000234071	0	0	0	0	0.124	0	0.9	0.205	0.924
KNG1	OPRD1	9606.ENSP00000265023	9606.ENSP00000234961	0	0	0	0	0	0	0.9	0.587	0.956
KNG1	TGFB3	9606.ENSP00000265023	9606.ENSP00000238682	0	0	0	0	0	0	0.9	0.104	0.906
KNG1	NTS	9606.ENSP00000265023	9606.ENSP00000256010	0	0	0	0	0	0	0.9	0.723	0.971
KNG1	THBS1	9606.ENSP00000265023	9606.ENSP00000260356	0	0	0	0	0	0.379	0.9	0.221	0.947
KNG1	PIK3R3	9606.ENSP00000265023	9606.ENSP00000262741	0	0	0	0	0	0	0.9	0	0.9
KNG1	PTGS2	9606.ENSP00000265023	9606.ENSP00000356438	0	0	0	0	0	0	0	0.731	0.731
KNG1	NGF	9606.ENSP00000265023	9606.ENSP00000358525	0	0	0	0	0	0	0	0.802	0.802
KNG1	XCL1	9606.ENSP00000265023	9606.ENSP00000356792	0	0	0	0	0	0	0.9	0	0.9
KNG1	TGFB2	9606.ENSP00000265023	9606.ENSP00000355896	0	0	0	0	0	0	0.9	0.064	0.902
KNG1	PTGFR	9606.ENSP00000265023	9606.ENSP00000359793	0	0	0	0	0.052	0	0.9	0.074	0.904
KNG1	TMSB4X	9606.ENSP00000265023	9606.ENSP00000370010	0	0	0	0	0	0	0.9	0.081	0.904
KNG1	SDC2	9606.ENSP00000265023	9606.ENSP00000307046	0	0	0	0	0.063	0	0.9	0.073	0.905
KNG1	S1PR1	9606.ENSP00000265023	9606.ENSP00000305416	0	0	0	0	0	0	0.9	0.116	0.907
KNG1	PROK2	9606.ENSP00000265023	9606.ENSP00000295619	0	0	0	0	0	0	0.9	0.123	0.908
KNG1	S1PR2	9606.ENSP00000265023	9606.ENSP00000466933	0	0	0	0	0	0	0.9	0.126	0.908
KNG1	VEGFC	9606.ENSP00000265023	9606.ENSP00000480043	0	0	0	0	0	0	0.9	0.144	0.91
KNG1	PDGFB	9606.ENSP00000265023	9606.ENSP00000330382	0	0	0	0	0	0	0.9	0.144	0.91
KNG1	SCG2	9606.ENSP00000265023	9606.ENSP00000304133	0	0	0	0	0	0	0.9	0.156	0.911
KNG1	OXTR	9606.ENSP00000265023	9606.ENSP00000324270	0	0	0	0	0	0	0.9	0.164	0.912
KNG1	SAA1	9606.ENSP00000265023	9606.ENSP00000384906	0	0	0	0	0.061	0	0.9	0.187	0.916
KNG1	SSTR2	9606.ENSP00000265023	9606.ENSP00000350198	0	0	0	0	0	0	0.9	0.249	0.921
KNG1	SPP1	9606.ENSP00000265023	9606.ENSP00000378517	0	0	0	0	0	0	0.9	0.267	0.923
KNG1	PTGER1	9606.ENSP00000265023	9606.ENSP00000292513	0	0	0	0	0	0	0.9	0.388	0.936
LCN2	SLPI	9606.ENSP00000362108	9606.ENSP00000342082	0	0	0	0	0.342	0	0.9	0.641	0.974
LCN2	PTPN6	9606.ENSP00000362108	9606.ENSP00000391592	0	0	0	0	0.069	0	0.9	0	0.902
LEPR	LIF	9606.ENSP00000330393	9606.ENSP00000249075	0	0	0	0	0	0	0.6	0.289	0.703
LEPR	SOCS3	9606.ENSP00000330393	9606.ENSP00000330341	0	0	0	0	0	0.465	0.9	0.838	0.99
LIF	OSMR	9606.ENSP00000249075	9606.ENSP00000274276	0	0	0	0	0.098	0.129	0.6	0.551	0.84
LIF	SHC1	9606.ENSP00000249075	9606.ENSP00000401303	0	0	0	0	0.076	0	0.9	0.118	0.911
LTB	PSMD5	9606.ENSP00000410481	9606.ENSP00000210313	0	0	0	0	0	0	0.9	0	0.9
LTB	PSME2	9606.ENSP00000410481	9606.ENSP00000216802	0	0	0	0	0.061	0	0.9	0	0.902
LTB	LTBR	9606.ENSP00000410481	9606.ENSP00000228918	0	0	0	0	0	0.882	0.9	0.69	0.996
LTB	TNFRSF1	9606.ENSP00000410481	9606.ENSP00000291232	0	0	0	0	0.159	0	0.9	0.187	0.925
LTB	TNFSF12	9606.ENSP00000410481	9606.ENSP00000293825	0	0	0	0	0.076	0	0.9	0.268	0.926
LTB	TNFRSF1	9606.ENSP00000410481	9606.ENSP00000326737	0	0	0	0	0	0	0.9	0.043	0.9
LTB	PSMB8	9606.ENSP00000410481	9606.ENSP00000364016	0	0	0	0	0.138	0	0.9	0.054	0.911
LTB	MAP3K14	9606.ENSP00000410481	9606.ENSP00000482657	0	0	0	0	0.061	0	0.9	0.351	0.933
LTBR	TNFRSF1	9606.ENSP00000228918	9606.ENSP00000162749	0	0	0	0.582	0.699	0	0	0.657	0.78
LTBR	PSMD5	9606.ENSP00000228918	9606.ENSP00000210313	0	0	0	0	0	0	0.9	0	0.9
LTBR	PSME2	9606.ENSP00000228918	9606.ENSP00000216802	0	0	0	0	0.061	0	0.9	0	0.902
LTBR	PSMB8	9606.ENSP00000228918	9606.ENSP00000364016	0	0	0	0	0.061	0	0.9	0.098	0.907
LTBR	TNFSF12	9606.ENSP00000228918	9606.ENSP00000293825	0	0	0	0	0	0	0.9	0.287	0.925
LTBR	TNFRSF1	9606.ENSP00000228918	9606.ENSP00000326737	0	0	0	0	0.083	0	0.9	0.367	0.937
LTBR	MAP3K14	9606.ENSP00000228918	9606.ENSP00000482657	0	0	0	0	0	0	0.9	0.409	0.938
LTBR	TNFRSF1	9606.ENSP00000228918	9606.ENSP00000291232	0	0	0	0	0	0	0.9	0.44	0.941
MAP3K14	TNFRSF1	9606.ENSP00000482657	9606.ENSP00000162749	0	0	0	0	0	0	0.9	0.372	0.934
MAP3K14	PSMD5	9606.ENSP00000482657	9606.ENSP00000210313	0	0	0	0	0	0	0.9	0	0.9
MAP3K14	PSME2	9606.ENSP00000482657	9606.ENSP00000216802	0	0	0	0	0	0.138	0.9	0	0.91
MAP3K14	RELB	9606.ENSP00000482657	9606.ENSP00000221452	0	0	0	0	0.072	0	0.9	0.572	0.956
MAP3K14	MAPK3	9606.ENSP00000482657	9606.ENSP00000263025	0	0	0	0.598	0.055	0.537	0.9	0.283	0.957
MAP3K14	MAP3K8	9606.ENSP00000482657	9606.ENSP00000263056	0	0	0	0.661	0.061	0.419	0.9	0.44	0.949
MAP3K14	TNFRSF1	9606.ENSP00000482657	9606.ENSP00000291232	0	0	0	0	0	0	0.9	0.469	0.944
MAP3K14	TNFSF12	9606.ENSP00000482657	9606.ENSP00000293825	0	0	0	0	0	0	0.9	0.297	0.926
MAP3K14	TNFRSF1	9606.ENSP00000482657	9606.ENSP00000326737	0	0	0	0	0	0	0.9	0.243	0.921
MAP3K14	PSMB8	9606.ENSP00000482657	9606.ENSP00000364016	0	0	0	0	0.06	0	0.9	0.05	0.902
MAP3K14	SPP1	9606.ENSP00000482657	9606.ENSP00000378517	0	0	0	0	0	0	0.9	0	0.9
MAP3K8	NFATC2	9606.ENSP00000263056	9606.ENSP00000379330	0	0	0	0	0.065	0	0.9	0.116	0.91
MAPK3	TNFSF10	9606.ENSP00000263025	9606.ENSP00000241261	0	0	0	0	0	0	0.9	0.359	0.933
MAPK3	PLCG1	9606.ENSP00000263025	9606.ENSP00000244007	0	0	0	0	0	0.126	0.9	0.58	0.96
MAPK3	PTGS2	9606.ENSP00000263025	9606.ENSP00000356438	0	0	0	0	0	0.414	0	0.706	0.821
MAPK3	MAPT	9606.ENSP00000263025	9606.ENSP00000340820	0	0	0	0	0	0	0.8	0.448	0.884
MAPK3	NOS1	9606.ENSP00000263025	9606.ENSP00000477999	0	0	0	0	0.05	0	0.8	0.45	0.886

MAPK3	PTPN6	9606.ENSP00000263025	9606.ENSP00000391592	0	0	0	0	0.057	0.26	0.8	0.302	0.889
MAPK3	SAA1	9606.ENSP00000263025	9606.ENSP00000384906	0	0	0	0	0	0	0.9	0.126	0.908
MAPK3	SDC2	9606.ENSP00000263025	9606.ENSP00000307046	0	0	0	0	0	0	0.9	0.157	0.912
MAPK3	MX1	9606.ENSP00000263025	9606.ENSP00000381601	0	0	0	0	0.06	0	0.9	0.236	0.921
MAPK3	SDC4	9606.ENSP00000263025	9606.ENSP00000361818	0	0	0	0	0	0	0.9	0.312	0.928
MAPK3	PRKCB	9606.ENSP00000263025	9606.ENSP00000305355	0	0	0	0.587	0.056	0.158	0.9	0.492	0.93
MAPK3	MET	9606.ENSP00000263025	9606.ENSP00000317272	0	0	0	0.582	0.059	0.146	0.9	0.637	0.935
MAPK3	SDC1	9606.ENSP00000263025	9606.ENSP00000370542	0	0	0	0	0.064	0	0.9	0.397	0.938
MAPK3	RXRA	9606.ENSP00000263025	9606.ENSP00000419692	0	0	0	0	0	0.363	0.9	0.117	0.938
MAPK3	S1PR1	9606.ENSP00000263025	9606.ENSP00000305416	0	0	0	0	0	0	0.9	0.416	0.939
MAPK3	PRKCA	9606.ENSP00000263025	9606.ENSP00000408695	0	0	0	0.588	0.056	0.261	0.9	0.627	0.943
MAPK3	S1PR2	9606.ENSP00000263025	9606.ENSP00000466933	0	0	0	0	0.077	0	0.9	0.483	0.948
MAPK3	NRG1	9606.ENSP00000263025	9606.ENSP00000384620	0	0	0	0	0	0.126	0.9	0.47	0.949
MAPK3	SRC	9606.ENSP00000263025	9606.ENSP00000362680	0	0	0.308	0.611	0.061	0.416	0.9	0.867	0.964
MAPK3	NGF	9606.ENSP00000263025	9606.ENSP00000358525	0	0	0	0	0	0	0.9	0.678	0.966
MAPK3	SHC1	9606.ENSP00000263025	9606.ENSP00000401303	0	0	0	0	0.062	0.084	0.9	0.716	0.972
MC1R	PTGER2	9606.ENSP00000451605	9606.ENSP00000245457	0	0	0	0	0	0	0.9	0.041	0.9
MC1R	PTH1R	9606.ENSP00000451605	9606.ENSP00000321999	0	0	0	0	0	0	0.9	0.111	0.907
MC1R	VIPR1	9606.ENSP00000451605	9606.ENSP00000327246	0	0	0	0	0	0	0.9	0.112	0.907
MC1R	VIP	9606.ENSP00000451605	9606.ENSP00000356213	0	0	0	0	0	0	0.9	0.147	0.911
MC1R	RLN2	9606.ENSP00000451605	9606.ENSP00000371040	0	0	0	0	0	0	0.9	0	0.9
MC1R	PTHLH	9606.ENSP00000451605	9606.ENSP00000441765	0	0	0	0	0	0	0.9	0.071	0.903
MDK	SDC4	9606.ENSP00000385451	9606.ENSP00000361818	0	0	0	0	0	0.379	0.9	0.255	0.949
MET	PLCG1	9606.ENSP00000317272	9606.ENSP00000244007	0	0	0	0	0	0.472	0.9	0.244	0.956
MET	SHC2	9606.ENSP00000317272	9606.ENSP00000264554	0	0	0	0	0	0.318	0.8	0.103	0.867
MET	TGFA	9606.ENSP00000317272	9606.ENSP00000295400	0	0	0	0	0.076	0	0.6	0.619	0.847
MET	PDGFA	9606.ENSP00000317272	9606.ENSP00000346508	0	0	0	0	0	0	0.6	0.295	0.705
MET	PDGFB	9606.ENSP00000317272	9606.ENSP00000330382	0	0	0	0	0	0	0.6	0.354	0.73
MET	PGF	9606.ENSP00000317272	9606.ENSP00000451040	0	0	0	0	0	0	0.6	0.364	0.734
MET	NGF	9606.ENSP00000317272	9606.ENSP00000358525	0	0	0	0	0.072	0.185	0.6	0.268	0.749
MET	VEGFC	9606.ENSP00000317272	9606.ENSP00000480043	0	0	0	0	0.112	0	0.6	0.441	0.784
MET	SHC3	9606.ENSP00000317272	9606.ENSP00000364995	0	0	0	0	0	0.318	0.8	0.098	0.866
MET	SDC1	9606.ENSP00000317272	9606.ENSP00000370542	0	0	0	0	0.138	0	0.9	0.318	0.936
MET	PLAUR	9606.ENSP00000317272	9606.ENSP00000339328	0	0	0	0	0.061	0	0.9	0.451	0.943
MET	SRC	9606.ENSP00000317272	9606.ENSP00000362680	0	0	0	0.684	0.063	0.407	0.9	0.795	0.954
MET	SHC1	9606.ENSP00000317272	9606.ENSP00000401303	0	0	0	0	0.062	0.52	0.9	0.447	0.971
MX1	PLCG1	9606.ENSP00000381601	9606.ENSP00000244007	0	0	0	0	0	0	0.9	0.08	0.904
MX1	TLR3	9606.ENSP00000381601	9606.ENSP00000296795	0	0	0	0	0.342	0.087	0	0.703	0.806
MX1	PSMB8	9606.ENSP00000381601	9606.ENSP00000364016	0	0	0	0	0.1	0.058	0.9	0.427	0.944
MX1	OAS1	9606.ENSP00000381601	9606.ENSP00000388001	0	0	0	0	0.877	0	0.9	0.837	0.997
NEDD4	SOCS3	9606.ENSP00000424827	9606.ENSP00000330341	0	0	0	0	0	0	0.9	0.102	0.906
NFATC2	NFATC4	9606.ENSP00000379330	9606.ENSP00000388910	0	0	0	0.778	0	0	0.8	0.834	0.836
NFKBIZ	TNFAIP3	9606.ENSP00000325663	9606.ENSP00000481570	0	0	0	0	0.331	0.148	0	0.541	0.716
NGF	NGFR	9606.ENSP00000358525	9606.ENSP00000172229	0	0	0	0	0	0.545	0.9	0.977	0.998
NGF	PLCG1	9606.ENSP00000358525	9606.ENSP00000244007	0	0	0	0	0	0	0.9	0.452	0.942
NGF	NTS	9606.ENSP00000358525	9606.ENSP00000256010	0	0	0	0	0	0	0	0.705	0.705
NGF	SHC2	9606.ENSP00000358525	9606.ENSP00000264554	0	0	0	0	0	0	0.9	0.206	0.917
NGF	SHC3	9606.ENSP00000358525	9606.ENSP00000364995	0	0	0	0	0.065	0	0.9	0.362	0.935
NGF	SHC1	9606.ENSP00000358525	9606.ENSP00000401303	0	0	0	0	0	0	0.9	0.73	0.971
NGFR	PIK3R3	9606.ENSP00000172229	9606.ENSP00000262741	0	0	0	0	0	0	0.8	0.043	0.8
NGFR	PLCG1	9606.ENSP00000172229	9606.ENSP00000244007	0	0	0	0	0	0	0.9	0.221	0.918
NGFR	SHC1	9606.ENSP00000172229	9606.ENSP00000401303	0	0	0	0	0	0.379	0.9	0.399	0.959
NOS1	PRKCB	9606.ENSP00000477999	9606.ENSP00000305355	0	0	0	0	0.061	0	0.9	0.122	0.91
NOS1	NOS2	9606.ENSP00000477999	9606.ENSP00000327251	0	0	0.438	0.95	0	0	0.8	0.812	0.812
NOS1	PRKCA	9606.ENSP00000477999	9606.ENSP00000408695	0	0	0	0	0.061	0.305	0.9	0.131	0.935
NOS2	TLR4	9606.ENSP00000327251	9606.ENSP00000363089	0	0	0	0	0.064	0	0	0.791	0.796
NOS2	PTGS2	9606.ENSP00000327251	9606.ENSP00000356438	0	0	0	0	0.063	0.299	0	0.868	0.906
NOS2	RXRA	9606.ENSP00000327251	9606.ENSP00000419692	0	0	0	0	0	0.091	0.9	0.087	0.909
NOS2	SRC	9606.ENSP00000327251	9606.ENSP00000362680	0	0	0	0	0.064	0.394	0.9	0.6	0.974
NOX1	RAC2	9606.ENSP00000362057	9606.ENSP00000249071	0	0	0	0	0.066	0.127	0.8	0.585	0.923
NOX1	RAC3	9606.ENSP00000362057	9606.ENSP00000304283	0	0	0	0	0.066	0.127	0.8	0.088	0.831
NPPC	NPR3	9606.ENSP00000387159	9606.ENSP00000265074	0	0	0	0	0	0.72	0	0.897	0.97
NPR3	OSTN	9606.ENSP00000265074	9606.ENSP00000342356	0	0	0	0	0	0	0	0.751	0.751
NR0B1	NR5A2	9606.ENSP00000368253	9606.ENSP00000356331	0	0	0	0.565	0.062	0.865	0	0.799	0.913
NR1H2	RXRA	9606.ENSP00000253727	9606.ENSP00000419692	0	0	0	0.694	0.061	0.825	0.9	0.713	0.985
NR1H4	RXRA	9606.ENSP00000447149	9606.ENSP00000419692	0	0	0	0.668	0.062	0.904	0.9	0.745	0.992
NRG1	SRC	9606.ENSP00000384620	9606.ENSP00000362680	0	0	0	0	0	0	0.9	0.438	0.941
NRG1	SHC1	9606.ENSP00000384620	9606.ENSP00000401303	0	0	0	0	0	0	0.9	0.243	0.921
NRP1	SEMA3G	9606.ENSP00000265371	9606.ENSP00000231721	0	0	0	0	0.061	0.195	0.8	0.826	0.97
NRP1	SEMA6A	9606.ENSP00000265371	9606.ENSP00000257414	0	0	0	0	0.061	0.195	0	0.828	0.859
NRP1	PLXNC1	9606.ENSP00000265371	9606.ENSP00000258526	0	0	0	0	0.064	0.261	0	0.632	0.723
NRP1	SEMA3C	9606.ENSP00000265371	9606.ENSP00000265361	0	0	0	0	0.088	0.478	0.9	0.839	0.991

NRP1	SEMA3A	9606.ENSP00000265371	9606.ENSP00000265362	0	0	0	0	0.061	0.841	0.9	0.949	0.999
NRP1	VEGFC	9606.ENSP00000265371	9606.ENSP00000480043	0	0	0	0	0.1	0.381	0	0.667	0.798
NRP1	NRP2	9606.ENSP00000265371	9606.ENSP00000353582	0	0	0	0.924	0.098	0.379	0.72	0.789	0.839
NRP1	SEMA6C	9606.ENSP00000265371	9606.ENSP00000357909	0	0	0	0	0	0.195	0	0.818	0.847
NRP1	SEMA4B	9606.ENSP00000265371	9606.ENSP00000394720	0	0	0	0	0	0.195	0	0.818	0.847
NRP1	SEMA5B	9606.ENSP00000265371	9606.ENSP00000389588	0	0	0	0	0.055	0.195	0	0.818	0.849
NRP1	SEMA6D	9606.ENSP00000265371	9606.ENSP00000324857	0	0	0	0	0.061	0.195	0	0.818	0.851
NRP1	SEMA4A	9606.ENSP00000265371	9606.ENSP00000357268	0	0	0	0	0	0.195	0	0.842	0.868
NRP1	PGF	9606.ENSP00000265371	9606.ENSP00000451040	0	0	0	0	0	0.379	0	0.809	0.876
NRP1	SEMA3D	9606.ENSP00000265371	9606.ENSP00000284136	0	0	0	0	0.061	0.195	0.8	0.831	0.971
NRP1	PLXNA4	9606.ENSP00000265371	9606.ENSP00000352882	0	0	0	0	0	0.372	0.9	0.677	0.977
NRP1	SEMA3E	9606.ENSP00000265371	9606.ENSP00000303212	0	0	0	0	0	0.195	0.9	0.83	0.985
NRP2	SEMA3G	9606.ENSP00000353582	9606.ENSP00000231721	0	0	0	0	0	0.164	0	0.843	0.863
NRP2	SEMA6A	9606.ENSP00000353582	9606.ENSP00000257414	0	0	0	0	0	0.164	0	0.836	0.857
NRP2	SEMA3C	9606.ENSP00000353582	9606.ENSP00000265361	0	0	0	0	0	0.458	0	0.839	0.909
NRP2	SEMA3A	9606.ENSP00000353582	9606.ENSP00000265362	0	0	0	0	0.098	0.164	0	0.89	0.91
NRP2	SEMA3D	9606.ENSP00000353582	9606.ENSP00000284136	0	0	0	0	0	0.164	0	0.827	0.849
NRP2	SEMA3E	9606.ENSP00000353582	9606.ENSP00000303212	0	0	0	0	0	0.164	0	0.837	0.858
NRP2	SEMA6D	9606.ENSP00000353582	9606.ENSP00000324857	0	0	0	0	0.065	0.164	0	0.826	0.852
NRP2	PLXNA4	9606.ENSP00000353582	9606.ENSP00000352882	0	0	0	0	0	0.185	0.72	0.766	0.941
NRP2	PGF	9606.ENSP00000353582	9606.ENSP00000451040	0	0	0	0	0	0.379	0	0.577	0.726
NRP2	SEMA4A	9606.ENSP00000353582	9606.ENSP00000357268	0	0	0	0	0	0.164	0	0.825	0.847
NRP2	SEMA6C	9606.ENSP00000353582	9606.ENSP00000357909	0	0	0	0	0	0.164	0	0.825	0.847
NRP2	SEMA5B	9606.ENSP00000353582	9606.ENSP00000389588	0	0	0	0	0	0.164	0	0.826	0.848
NRP2	SEMA4B	9606.ENSP00000353582	9606.ENSP00000394720	0	0	0	0	0	0.164	0	0.826	0.848
NRP2	VEGFC	9606.ENSP00000353582	9606.ENSP00000480043	0	0	0	0	0.077	0.381	0.9	0.831	0.989
NRTN	PLCG1	9606.ENSP00000302648	9606.ENSP00000244007	0	0	0	0	0	0	0.9	0	0.9
NRTN	PIK3R3	9606.ENSP00000302648	9606.ENSP00000262741	0	0	0	0	0	0	0.9	0	0.9
NRTN	SHC3	9606.ENSP00000302648	9606.ENSP00000364995	0	0	0	0	0	0	0.9	0.048	0.9
NRTN	PRKCA	9606.ENSP00000302648	9606.ENSP00000408695	0	0	0	0	0	0	0.9	0.059	0.901
NRTN	SHC1	9606.ENSP00000302648	9606.ENSP00000401303	0	0	0	0	0	0	0.9	0.206	0.917
NTS	VIP	9606.ENSP00000256010	9606.ENSP00000356213	0	0	0	0	0.062	0	0	0.745	0.751
NTS	XCL1	9606.ENSP00000256010	9606.ENSP00000356792	0	0	0	0	0	0	0.9	0	0.9
NTS	PIK3R3	9606.ENSP00000256010	9606.ENSP00000262741	0	0	0	0	0	0	0.9	0.041	0.9
NTS	SAA1	9606.ENSP00000256010	9606.ENSP00000384906	0	0	0	0	0	0	0.9	0.074	0.903
NTS	PROK2	9606.ENSP00000256010	9606.ENSP00000295619	0	0	0	0	0	0	0.9	0.164	0.912
NTS	PTGFR	9606.ENSP00000256010	9606.ENSP00000359793	0	0	0	0	0	0.263	0.9	0.093	0.927
NTS	PTGER1	9606.ENSP00000256010	9606.ENSP00000292513	0	0	0	0	0	0.263	0.9	0.104	0.928
NTS	OXTR	9606.ENSP00000256010	9606.ENSP00000324270	0	0	0	0	0	0.263	0.9	0.299	0.943
OAS1	VCAM1	9606.ENSP00000388001	9606.ENSP00000294728	0	0	0	0	0	0	0.9	0.09	0.905
OAS1	PSMB8	9606.ENSP00000388001	9606.ENSP00000364016	0	0	0	0	0.116	0	0.9	0.328	0.935
OLR1	PLAU	9606.ENSP00000309124	9606.ENSP00000361850	0	0	0	0	0	0	0.9	0.111	0.907
OLR1	PLAUR	9606.ENSP00000309124	9606.ENSP00000339328	0	0	0	0	0.071	0	0.9	0.122	0.911
OPRD1	SAA1	9606.ENSP00000234961	9606.ENSP00000384906	0	0	0	0	0	0	0.9	0	0.9
OPRD1	S1PR2	9606.ENSP00000234961	9606.ENSP00000466933	0	0	0	0	0	0	0.9	0.064	0.902
OPRD1	S1PR1	9606.ENSP00000234961	9606.ENSP00000305416	0	0	0	0.587	0	0	0.9	0.227	0.908
OPRD1	SSTR2	9606.ENSP00000234961	9606.ENSP00000350198	0	0	0	0.865	0.142	0	0.9	0.45	0.915
OXTR	PIK3R3	9606.ENSP00000324270	9606.ENSP00000262741	0	0	0	0	0	0.057	0.9	0	0.901
OXTR	PTGER1	9606.ENSP00000324270	9606.ENSP00000292513	0	0	0	0	0	0	0.9	0.146	0.911
OXTR	PROK2	9606.ENSP00000324270	9606.ENSP00000295619	0	0	0	0	0	0	0.9	0	0.9
OXTR	SAA1	9606.ENSP00000324270	9606.ENSP00000384906	0	0	0	0	0	0	0.9	0	0.9
OXTR	XCL1	9606.ENSP00000324270	9606.ENSP00000356792	0	0	0	0	0	0.059	0.9	0	0.901
OXTR	PTGFR	9606.ENSP00000324270	9606.ENSP00000359793	0	0	0	0	0	0	0.9	0.483	0.946
PAK7	RAC2	9606.ENSP00000367686	9606.ENSP00000249071	0	0	0	0	0.061	0.735	0.6	0.224	0.912
PAK7	RAC3	9606.ENSP00000367686	9606.ENSP00000304283	0	0	0	0	0.061	0.735	0.6	0.311	0.922
PAK7	SRC	9606.ENSP00000367686	9606.ENSP00000362680	0	0	0	0.576	0.058	0.157	0.9	0.199	0.919
PAK7	ROBO2	9606.ENSP00000367686	9606.ENSP00000417335	0	0	0	0	0.063	0.106	0.8	0.106	0.83
PAK7	SLIT2	9606.ENSP00000367686	9606.ENSP00000422591	0	0	0	0	0.062	0.063	0.9	0.088	0.909
PDGFA	PLCG1	9606.ENSP00000346508	9606.ENSP00000244007	0	0	0	0	0	0.379	0.9	0.237	0.948
PDGFA	TGFA	9606.ENSP00000346508	9606.ENSP00000295400	0	0	0	0	0	0	0.8	0.446	0.884
PDGFA	PDGFB	9606.ENSP00000346508	9606.ENSP00000330382	0	0	0	0.876	0.061	0.305	0.9	0.902	0.936
PDGFA	SHC1	9606.ENSP00000346508	9606.ENSP00000401303	0	0	0	0	0	0	0.9	0.239	0.92
PDGFA	PDGFD	9606.ENSP00000346508	9606.ENSP00000376865	0	0	0	0	0	0	0.8	0.816	0.961
PDGFB	TGFB3	9606.ENSP00000330382	9606.ENSP00000238682	0	0	0	0	0.082	0	0.9	0.318	0.931
PDGFB	PLCG1	9606.ENSP00000330382	9606.ENSP00000244007	0	0	0	0	0	0	0.9	0.221	0.918
PDGFB	THBS1	9606.ENSP00000330382	9606.ENSP00000260356	0	0	0	0	0.049	0.379	0.9	0.399	0.959
PDGFB	TGFA	9606.ENSP00000330382	9606.ENSP00000295400	0	0	0	0	0.076	0	0.8	0.398	0.879
PDGFB	S1PR1	9606.ENSP00000330382	9606.ENSP00000305416	0	0	0	0	0	0	0.9	0.229	0.919
PDGFB	TMSB4X	9606.ENSP00000330382	9606.ENSP00000370010	0	0	0	0	0	0	0.9	0.061	0.902
PDGFB	SHC1	9606.ENSP00000330382	9606.ENSP00000401303	0	0	0	0	0	0	0.9	0.198	0.916
PDGFB	SPP1	9606.ENSP00000330382	9606.ENSP00000378517	0	0	0	0	0.069	0	0.9	0.287	0.927
PDGFB	TGFB2	9606.ENSP00000330382	9606.ENSP00000355896	0	0	0	0	0.07	0	0.9	0.42	0.941

PDGFB	SRC	9606.ENSP00000330382	9606.ENSP00000362680	0	0	0	0	0.076	0	0.9	0.473	0.947
PDGFB	VEGFC	9606.ENSP00000330382	9606.ENSP00000480043	0	0	0	0	0	0	0.9	0.564	0.954
PDGFB	PDGFD	9606.ENSP00000330382	9606.ENSP00000376865	0	0	0	0	0.061	0	0.9	0.785	0.978
PDGFD	TGFA	9606.ENSP00000376865	9606.ENSP00000295400	0	0	0	0	0	0	0.8	0.165	0.825
PDGFD	PLAUR	9606.ENSP00000376865	9606.ENSP00000339328	0	0	0	0	0	0	0.9	0.178	0.914
PDGFD	PLAU	9606.ENSP00000376865	9606.ENSP00000361850	0	0	0	0	0	0	0.9	0.214	0.918
PGF	PLCG1	9606.ENSP00000451040	9606.ENSP00000244007	0	0	0	0	0	0	0.9	0.069	0.902
PGF	PTGFR	9606.ENSP00000451040	9606.ENSP00000359793	0	0	0	0	0	0	0	0.746	0.746
PGF	VEGFC	9606.ENSP00000451040	9606.ENSP00000480043	0	0	0	0.69	0	0	0.9	0.905	0.927
PIK3R3	PLCG1	9606.ENSP00000262741	9606.ENSP00000244007	0	0	0	0.556	0	0	0.65	0.393	0.707
PIK3R3	RAC2	9606.ENSP00000262741	9606.ENSP00000249071	0	0	0	0	0	0.086	0.9	0.113	0.911
PIK3R3	PROK2	9606.ENSP00000262741	9606.ENSP00000295619	0	0	0	0	0	0	0.9	0	0.9
PIK3R3	XCL1	9606.ENSP00000262741	9606.ENSP00000356792	0	0	0	0	0	0	0.9	0	0.9
PIK3R3	SAA1	9606.ENSP00000262741	9606.ENSP00000384906	0	0	0	0	0	0	0.9	0	0.9
PIK3R3	PTGFR	9606.ENSP00000262741	9606.ENSP00000359793	0	0	0	0	0	0.057	0.9	0	0.901
PIK3R3	PTGER1	9606.ENSP00000262741	9606.ENSP00000292513	0	0	0	0	0	0.057	0.9	0	0.901
PIK3R3	SYK	9606.ENSP00000262741	9606.ENSP00000364907	0	0	0	0	0	0.085	0.9	0.134	0.913
PIK3R3	SHC1	9606.ENSP00000262741	9606.ENSP00000401303	0	0	0	0	0	0.064	0.9	0.188	0.917
PIK3R3	SRC	9606.ENSP00000262741	9606.ENSP00000362680	0	0	0	0.556	0	0.535	0.9	0.315	0.957
PIK3R3	PIK3R5	9606.ENSP00000262741	9606.ENSP00000392812	0	0	0	0	0	0	0.9	0.657	0.964
PIK3R5	SYK	9606.ENSP00000392812	9606.ENSP00000364907	0	0	0	0	0.182	0	0.9	0.22	0.93
PLAU	VTN	9606.ENSP00000361850	9606.ENSP00000226218	0	0	0	0	0	0	0.9	0.669	0.965
PLAU	PLAUR	9606.ENSP00000361850	9606.ENSP00000339328	0	0	0	0	0.221	0.723	0.9	0.967	0.999
PLAU	SRC	9606.ENSP00000361850	9606.ENSP00000362680	0	0	0	0	0.069	0.05	0.9	0.443	0.944
PLAUR	VTN	9606.ENSP00000339328	9606.ENSP00000226218	0	0	0	0	0	0.87	0.9	0.871	0.998
PLAUR	SRC	9606.ENSP00000339328	9606.ENSP00000362680	0	0	0	0	0	0	0.9	0.495	0.947
PLCG1	SHC1	9606.ENSP00000244007	9606.ENSP00000401303	0	0	0	0	0	0.379	0	0.623	0.756
PLCG1	SDC2	9606.ENSP00000244007	9606.ENSP00000307046	0	0	0	0	0	0	0.9	0.049	0.9
PLCG1	S1PR1	9606.ENSP00000244007	9606.ENSP00000305416	0	0	0	0	0	0	0.9	0.054	0.901
PLCG1	PRKCB	9606.ENSP00000244007	9606.ENSP00000305355	0	0	0	0	0.062	0.082	0.9	0.356	0.937
PLCG1	PRKCA	9606.ENSP00000244007	9606.ENSP00000408695	0	0	0	0	0.062	0.082	0.9	0.479	0.949
PLCG1	SRC	9606.ENSP00000244007	9606.ENSP00000362680	0	0	0	0.55	0.061	0.472	0.9	0.798	0.965
PLCG1	SYK	9606.ENSP00000244007	9606.ENSP00000364907	0	0	0	0.556	0.063	0.974	0.9	0.786	0.998
PLTP	SAA1	9606.ENSP00000417138	9606.ENSP00000384906	0	0	0	0	0	0	0.54	0.377	0.701
PLXNA4	SEMA3G	9606.ENSP00000352882	9606.ENSP00000231721	0	0	0	0	0.076	0.188	0.6	0.899	0.966
PLXNA4	RAC2	9606.ENSP00000352882	9606.ENSP00000249071	0	0	0	0	0	0.264	0.6	0.231	0.753
PLXNA4	SEMA6A	9606.ENSP00000352882	9606.ENSP00000257414	0	0	0	0	0.061	0.38	0.9	0.926	0.995
PLXNA4	SEMA3C	9606.ENSP00000352882	9606.ENSP00000265361	0	0	0	0	0	0.188	0.6	0.899	0.964
PLXNA4	SEMA3A	9606.ENSP00000352882	9606.ENSP00000265362	0	0	0	0	0	0.365	0.9	0.905	0.993
PLXNA4	SEMA3D	9606.ENSP00000352882	9606.ENSP00000284136	0	0	0	0	0	0.188	0.6	0.899	0.964
PLXNA4	SEMA3E	9606.ENSP00000352882	9606.ENSP00000303212	0	0	0	0	0.061	0.188	0.6	0.899	0.965
PLXNA4	RAC3	9606.ENSP00000352882	9606.ENSP00000304283	0	0	0	0	0.061	0.264	0.6	0.234	0.759
PLXNA4	SEMA6C	9606.ENSP00000352882	9606.ENSP00000357909	0	0	0	0	0.09	0.38	0	0.913	0.947
PLXNC1	SEMA6A	9606.ENSP00000258526	9606.ENSP00000257414	0	0	0	0	0	0.188	0	0.899	0.915
PLXNC1	SEMA3A	9606.ENSP00000258526	9606.ENSP00000265362	0	0	0	0	0	0.188	0	0.877	0.896
PLXNC1	SEMA6C	9606.ENSP00000258526	9606.ENSP00000357909	0	0	0	0	0	0.188	0	0.877	0.896
PLXNC1	SEMA3D	9606.ENSP00000258526	9606.ENSP00000284136	0	0	0	0	0	0.188	0	0.896	0.912
PLXNC1	SEMA6D	9606.ENSP00000258526	9606.ENSP00000324857	0	0	0	0	0	0.188	0	0.896	0.912
PLXNC1	SEMA3C	9606.ENSP00000258526	9606.ENSP00000265361	0	0	0	0	0	0.188	0	0.895	0.912
PLXNC1	SEMA3E	9606.ENSP00000258526	9606.ENSP00000303212	0	0	0	0	0	0.188	0	0.897	0.914
PLXNC1	SEMA5B	9606.ENSP00000258526	9606.ENSP00000389588	0	0	0	0	0	0.188	0	0.901	0.917
PRKCA	WNT5A	9606.ENSP00000408695	9606.ENSP00000264634	0	0	0	0	0	0	0.9	0.235	0.92
PRKCA	PRKCB	9606.ENSP00000408695	9606.ENSP00000305355	0	0	0	0.981	0	0.39	0.9	0.9	0.937
PRKCA	SDC4	9606.ENSP00000408695	9606.ENSP00000361818	0	0	0	0	0	0.454	0.9	0.72	0.983
PRKCA	SRC	9606.ENSP00000408695	9606.ENSP00000362680	0	0	0	0.575	0	0.399	0.9	0.738	0.956
PRKCA	PTK2B	9606.ENSP00000408695	9606.ENSP00000380638	0	0	0	0.553	0	0	0.9	0.269	0.91
PRKCA	ROBO2	9606.ENSP00000408695	9606.ENSP00000417335	0	0	0	0	0.061	0	0.9	0	0.902
PRKCA	RXRA	9606.ENSP00000408695	9606.ENSP00000419692	0	0	0	0	0	0	0.9	0.123	0.908
PRKCB	WNT5A	9606.ENSP00000305355	9606.ENSP00000264634	0	0	0	0	0	0	0.9	0.187	0.915
PRKCB	PTK2B	9606.ENSP00000305355	9606.ENSP00000380638	0	0	0	0.552	0.123	0	0.9	0.187	0.914
PRKCB	SRC	9606.ENSP00000305355	9606.ENSP00000362680	0	0	0	0.572	0	0.073	0.9	0.323	0.915
PROC	SCG2	9606.ENSP00000234071	9606.ENSP00000304133	0	0	0	0	0	0	0.9	0	0.9
PROC	SDC2	9606.ENSP00000234071	9606.ENSP00000307046	0	0	0	0	0.06	0	0.9	0	0.901
PROC	SPP1	9606.ENSP00000234071	9606.ENSP00000378517	0	0	0	0	0	0	0.9	0.093	0.905
PROK2	PTGER1	9606.ENSP00000295619	9606.ENSP00000292513	0	0	0	0	0	0	0.9	0	0.9
PROK2	XCL1	9606.ENSP00000295619	9606.ENSP00000356792	0	0	0	0	0	0	0.9	0	0.9
PROK2	PTGFR	9606.ENSP00000295619	9606.ENSP00000359793	0	0	0	0	0	0	0.9	0	0.9
PROK2	SAA1	9606.ENSP00000295619	9606.ENSP00000384906	0	0	0	0	0	0	0.9	0.073	0.903
PSMB8	PSMD5	9606.ENSP00000364016	9606.ENSP00000210313	0	0	0	0	0.061	0.869	0.9	0.223	0.989
PSMB8	PSME2	9606.ENSP00000364016	9606.ENSP00000216802	0	0	0	0	0.264	0.299	0.9	0.685	0.981
PSMB8	RELB	9606.ENSP00000364016	9606.ENSP00000221452	0	0	0	0	0.1	0	0.9	0.187	0.92
PSMB8	TNFRSF1	9606.ENSP00000364016	9606.ENSP00000291232	0	0	0	0	0.062	0	0.9	0	0.902

PSMB8	TNFSF12	9606.ENSP00000364016	9606.ENSP00000293825	0	0	0	0	0.076	0	0.9	0	0.903
PSMB8	TNFRSF1	9606.ENSP00000364016	9606.ENSP00000326737	0	0	0	0	0	0	0.9	0.047	0.9
PSMD5	TNFSF12	9606.ENSP00000210313	9606.ENSP00000293825	0	0	0	0	0	0	0.9	0	0.9
PSMD5	TNFRSF1	9606.ENSP00000210313	9606.ENSP00000326737	0	0	0	0	0	0	0.9	0.049	0.9
PSMD5	RELB	9606.ENSP00000210313	9606.ENSP00000221452	0	0	0	0	0	0	0.9	0.074	0.903
PSMD5	TNFRSF1	9606.ENSP00000210313	9606.ENSP00000291232	0	0	0	0	0	0	0.9	0.145	0.91
PSMD5	PSME2	9606.ENSP00000210313	9606.ENSP00000216802	0	0	0	0	0.061	0	0.9	0.257	0.924
PSME2	TNFSF12	9606.ENSP00000216802	9606.ENSP00000293825	0	0	0	0	0	0	0.9	0.048	0.9
PSME2	TNFRSF1	9606.ENSP00000216802	9606.ENSP00000291232	0	0	0	0	0	0	0.9	0	0.9
PSME2	TNFRSF1	9606.ENSP00000216802	9606.ENSP00000326737	0	0	0	0	0.061	0	0.9	0	0.902
PSME2	RELB	9606.ENSP00000216802	9606.ENSP00000221452	0	0	0	0	0	0	0.9	0.095	0.905
PTGER1	PTGS2	9606.ENSP00000292513	9606.ENSP00000356438	0	0	0	0	0	0.454	0	0.794	0.883
PTGER1	SAA1	9606.ENSP00000292513	9606.ENSP00000384906	0	0	0	0	0	0	0.9	0	0.9
PTGER1	XCL1	9606.ENSP00000292513	9606.ENSP00000356792	0	0	0	0	0	0.059	0.9	0	0.901
PTGER1	PTGFR	9606.ENSP00000292513	9606.ENSP00000359793	0	0	0	0.871	0	0.379	0.9	0.623	0.94
PTGER2	PTGS2	9606.ENSP00000245457	9606.ENSP00000356438	0	0	0	0	0.098	0.056	0	0.777	0.794
PTGER2	PTHLH	9606.ENSP00000245457	9606.ENSP00000441765	0	0	0	0	0	0	0.9	0.041	0.9
PTGER2	RLN2	9606.ENSP00000245457	9606.ENSP00000371040	0	0	0	0	0	0	0.9	0	0.9
PTGER2	PTH1R	9606.ENSP00000245457	9606.ENSP00000321999	0	0	0	0	0	0	0.9	0	0.9
PTGER2	VIPR1	9606.ENSP00000245457	9606.ENSP00000327246	0	0	0	0	0	0	0.9	0	0.9
PTGER2	VIP	9606.ENSP00000245457	9606.ENSP00000356213	0	0	0	0	0	0	0.9	0.067	0.902
PTGFR	XCL1	9606.ENSP00000359793	9606.ENSP00000356792	0	0	0	0	0	0.059	0.9	0	0.901
PTGFR	SAA1	9606.ENSP00000359793	9606.ENSP00000384906	0	0	0	0	0	0	0.9	0	0.9
PTGS2	VCAM1	9606.ENSP00000356438	9606.ENSP00000294728	0	0	0	0	0.061	0	0	0.778	0.783
PTGS2	SOCS3	9606.ENSP00000356438	9606.ENSP00000330341	0	0	0	0	0.165	0	0	0.655	0.7
PTGS2	VEGFC	9606.ENSP00000356438	9606.ENSP00000480043	0	0	0	0	0.092	0	0	0.702	0.718
PTGS2	TLR4	9606.ENSP00000356438	9606.ENSP00000363089	0	0	0	0	0.14	0	0	0.816	0.835
PTH1R	RLN2	9606.ENSP00000321999	9606.ENSP00000371040	0	0	0	0	0	0	0.9	0.049	0.9
PTH1R	VIPR1	9606.ENSP00000321999	9606.ENSP00000327246	0	0	0	0.871	0	0	0.9	0.377	0.904
PTH1R	VIP	9606.ENSP00000321999	9606.ENSP00000356213	0	0	0	0	0.077	0.176	0.9	0.335	0.942
PTH1R	PTHLH	9606.ENSP00000321999	9606.ENSP00000441765	0	0	0	0	0	0.379	0.9	0.885	0.992
PTHLH	VIPR1	9606.ENSP00000441765	9606.ENSP00000327246	0	0	0	0	0	0	0.9	0.188	0.915
PTHLH	VIP	9606.ENSP00000441765	9606.ENSP00000356213	0	0	0	0	0.062	0	0.9	0.261	0.924
PTHLH	RLN2	9606.ENSP00000441765	9606.ENSP00000371040	0	0	0	0	0	0	0.9	0.067	0.902
PTK2B	VTN	9606.ENSP00000380638	9606.ENSP00000226218	0	0	0	0	0	0	0.9	0.359	0.933
PTK2B	RAC2	9606.ENSP00000380638	9606.ENSP00000249071	0	0	0	0	0.133	0	0.8	0.429	0.892
PTK2B	VCAM1	9606.ENSP00000380638	9606.ENSP00000294728	0	0	0	0	0.064	0	0.9	0.311	0.929
PTK2B	RAC3	9606.ENSP00000380638	9606.ENSP00000304283	0	0	0	0	0.087	0	0.8	0.299	0.86
PTK2B	SOCS3	9606.ENSP00000380638	9606.ENSP00000330341	0	0	0	0	0	0.387	0	0.604	0.747
PTK2B	SRC	9606.ENSP00000380638	9606.ENSP00000362680	0	0	0	0.711	0.062	0.521	0.9	0.928	0.964
PTK2B	SYK	9606.ENSP00000380638	9606.ENSP00000364907	0	0	0	0.687	0.1	0.373	0.9	0.731	0.952
PTK2B	SPP1	9606.ENSP00000380638	9606.ENSP00000378517	0	0	0	0	0	0	0.9	0.266	0.923
PTK2B	SHC1	9606.ENSP00000380638	9606.ENSP00000401303	0	0	0	0	0	0.379	0.9	0.584	0.971
PTPN6	SOCS3	9606.ENSP00000391592	9606.ENSP00000330341	0	0	0	0	0.062	0.061	0.9	0.297	0.929
PTPN6	SLPI	9606.ENSP00000391592	9606.ENSP00000342082	0	0	0	0	0.062	0	0.9	0	0.902
PTPN6	TNFRSF1	9606.ENSP00000391592	9606.ENSP00000347948	0	0	0	0	0.127	0.195	0.9	0.111	0.929
PTPN6	SRC	9606.ENSP00000391592	9606.ENSP00000362680	0	0	0	0	0.062	0.457	0	0.643	0.802
PTPN6	SYK	9606.ENSP00000391592	9606.ENSP00000364907	0	0	0	0	0.23	0.456	0.9	0.707	0.986
PTPN6	SHC1	9606.ENSP00000391592	9606.ENSP00000401303	0	0	0	0	0	0.402	0.9	0.41	0.961
RAC2	WNT5A	9606.ENSP00000249071	9606.ENSP00000264634	0	0	0	0	0	0.101	0.9	0.303	0.931
RAC2	SRC	9606.ENSP00000249071	9606.ENSP00000362680	0	0	0	0	0	0.16	0.8	0.662	0.938
RAC2	SYK	9606.ENSP00000249071	9606.ENSP00000364907	0	0	0	0	0.159	0.16	0.9	0.473	0.957
RAC2	RAC3	9606.ENSP00000249071	9606.ENSP00000304283	0	0	0.449	0.983	0	0.855	0.9	0.38	0.985
RAC3	WNT5A	9606.ENSP00000304283	9606.ENSP00000264634	0	0	0	0	0	0.101	0.9	0.182	0.92
RAC3	SRC	9606.ENSP00000304283	9606.ENSP00000362680	0	0	0	0	0.073	0.16	0.8	0.57	0.924
RAET1E	RAET1G	9606.ENSP00000349709	9606.ENSP00000356329	0	0	0	0.796	0.062	0	0.8	0.844	0.837
RAET1G	ULBP2	9606.ENSP00000356329	9606.ENSP00000356320	0	0	0	0.981	0.197	0	0.9	0.733	0.917
RBP1	SDC1	9606.ENSP00000232219	9606.ENSP00000370542	0	0	0	0	0	0	0.9	0.045	0.9
RBP1	SDC2	9606.ENSP00000232219	9606.ENSP00000307046	0	0	0	0	0	0	0.9	0	0.9
RBP1	SDC4	9606.ENSP00000232219	9606.ENSP00000361818	0	0	0	0	0	0	0.9	0.151	0.911
RBP1	RBP4	9606.ENSP00000232219	9606.ENSP00000360522	0	0	0	0	0.062	0	0.9	0.639	0.963
RLN2	VIPR1	9606.ENSP00000371040	9606.ENSP00000327246	0	0	0	0	0	0	0.9	0	0.9
RLN2	VIP	9606.ENSP00000371040	9606.ENSP00000356213	0	0	0	0	0	0	0.9	0.064	0.902
ROBO2	SLIT1	9606.ENSP00000417335	9606.ENSP00000266058	0	0	0	0	0.095	0.63	0.9	0.905	0.996
ROBO2	SLIT2	9606.ENSP00000417335	9606.ENSP00000422591	0	0	0	0	0.088	0.76	0.9	0.856	0.996
ROBO3	SLIT1	9606.ENSP00000380903	9606.ENSP00000266058	0	0	0	0	0.061	0.63	0	0.848	0.942
ROBO3	SLIT2	9606.ENSP00000380903	9606.ENSP00000422591	0	0	0	0	0.061	0.63	0	0.807	0.927
S100A1	S100P	9606.ENSP00000292169	9606.ENSP00000296370	0	0	0	0.939	0	0.777	0	0.31	0.78
S100A1	S100A3	9606.ENSP00000292169	9606.ENSP00000357702	0	0	0	0.89	0	0.768	0	0.716	0.785
S100A1	S100A2	9606.ENSP00000292169	9606.ENSP00000357697	0	0	0	0.932	0.103	0.768	0	0.782	0.794
S100A1	TLR4	9606.ENSP00000292169	9606.ENSP00000363089	0	0	0	0	0	0	0.9	0.57	0.955
S100A2	S100A3	9606.ENSP00000357697	9606.ENSP00000357702	0	0	0	0.907	0.331	0.791	0	0.73	0.863

S100A6	TMSB10	9606.ENSP00000357709	9606.ENSP00000233143	0	0	0	0	0.139	0	0	0.755	0.78
S1PR1	SSTR2	9606.ENSP00000305416	9606.ENSP00000350198	0	0	0	0.581	0	0	0.9	0	0.9
S1PR1	SAA1	9606.ENSP00000305416	9606.ENSP00000384906	0	0	0	0	0	0	0.9	0	0.9
S1PR1	SHC1	9606.ENSP00000305416	9606.ENSP00000401303	0	0	0	0	0	0	0.9	0.107	0.906
S1PR1	S1PR2	9606.ENSP00000305416	9606.ENSP00000466933	0	0	0	0.933	0	0.379	0.9	0.882	0.939
S1PR1	SRC	9606.ENSP00000305416	9606.ENSP00000362680	0	0	0	0	0	0.323	0.9	0.255	0.945
S1PR2	SSTR2	9606.ENSP00000466933	9606.ENSP00000350198	0	0	0	0.577	0	0	0.9	0.058	0.9
S1PR2	SAA1	9606.ENSP00000466933	9606.ENSP00000384906	0	0	0	0	0	0	0.9	0	0.9
SAA1	SSTR2	9606.ENSP00000384906	9606.ENSP00000350198	0	0	0	0	0	0	0.9	0.046	0.9
SAA1	XCL1	9606.ENSP00000384906	9606.ENSP00000356792	0	0	0	0	0.062	0	0.9	0.047	0.902
SCG2	SDC2	9606.ENSP00000304133	9606.ENSP00000307046	0	0	0	0	0.069	0	0.9	0	0.902
SCG2	SPP1	9606.ENSP00000304133	9606.ENSP00000378517	0	0	0	0	0.076	0	0.9	0.148	0.914
SDC1	VTN	9606.ENSP00000370542	9606.ENSP00000226218	0	0	0	0	0	0	0.9	0.419	0.939
SDC1	THBS1	9606.ENSP00000370542	9606.ENSP00000260356	0	0	0	0	0.095	0	0.9	0.297	0.93
SDC1	SDC2	9606.ENSP00000370542	9606.ENSP00000307046	0	0	0	0.727	0.071	0.305	0.9	0.873	0.946
SDC1	SDC4	9606.ENSP00000370542	9606.ENSP00000361818	0	0	0	0.693	0.111	0	0.9	0.803	0.929
SDC2	VTN	9606.ENSP00000307046	9606.ENSP00000226218	0	0	0	0	0.079	0	0.9	0.297	0.929
SDC2	SHC1	9606.ENSP00000307046	9606.ENSP00000401303	0	0	0	0	0	0	0.9	0.189	0.915
SDC2	SPP1	9606.ENSP00000307046	9606.ENSP00000378517	0	0	0	0	0.103	0	0.9	0.193	0.921
SDC2	SRC	9606.ENSP00000307046	9606.ENSP00000362680	0	0	0	0	0	0	0.9	0.272	0.924
SDC2	SDC4	9606.ENSP00000307046	9606.ENSP00000361818	0	0	0	0.824	0.065	0.305	0.9	0.876	0.94
SDC4	VTN	9606.ENSP00000361818	9606.ENSP00000226218	0	0	0	0	0	0	0.9	0.42	0.939
SDC4	THBS1	9606.ENSP00000361818	9606.ENSP00000260356	0	0	0	0	0.098	0	0.9	0.398	0.94
SEMA3A	SLIT1	9606.ENSP00000265362	9606.ENSP00000266058	0	0	0	0	0.055	0	0	0.732	0.736
SEMA3A	SLIT2	9606.ENSP00000265362	9606.ENSP00000422591	0	0	0	0	0.116	0	0	0.729	0.751
SEMA5B	THBS1	9606.ENSP00000389588	9606.ENSP00000260356	0	0	0	0.554	0	0	0.9	0.213	0.907
SHC1	TGFB3	9606.ENSP00000401303	9606.ENSP00000238682	0	0	0	0	0	0	0.9	0.189	0.915
SHC1	SHC2	9606.ENSP00000401303	9606.ENSP00000264554	0	0	0	0.922	0	0.379	0.9	0.642	0.938
SHC1	TGFBR2	9606.ENSP00000401303	9606.ENSP00000351905	0	0	0	0	0.072	0.52	0.9	0.221	0.96
SHC1	SRC	9606.ENSP00000401303	9606.ENSP00000362680	0	0	0	0.552	0.052	0.52	0.9	0.922	0.97
SHC1	SYK	9606.ENSP00000401303	9606.ENSP00000364907	0	0	0	0	0.061	0.52	0.9	0.751	0.987
SHC1	SHC3	9606.ENSP00000401303	9606.ENSP00000364995	0	0	0	0.92	0	0	0.9	0.678	0.905
SHC1	VEGFC	9606.ENSP00000401303	9606.ENSP00000480043	0	0	0	0	0.076	0	0.9	0.21	0.92
SHC2	SYK	9606.ENSP00000264554	9606.ENSP00000364907	0	0	0	0	0	0.26	0.8	0.179	0.868
SHC2	SRC	9606.ENSP00000264554	9606.ENSP00000362680	0	0	0	0	0.059	0.268	0.8	0.411	0.908
SHC2	SHC3	9606.ENSP00000264554	9606.ENSP00000364995	0	0	0	0.909	0.119	0	0.9	0.828	0.914
SHC3	SRC	9606.ENSP00000364995	9606.ENSP00000362680	0	0	0	0	0.057	0.268	0.8	0.442	0.912
SHC3	SYK	9606.ENSP00000364995	9606.ENSP00000364907	0	0	0	0	0	0.26	0.8	0.138	0.861
SLIT1	SLIT2	9606.ENSP00000266058	9606.ENSP00000422591	0	0	0	0.972	0	0	0.9	0.503	0.901
SPP1	VTN	9606.ENSP00000378517	9606.ENSP00000226218	0	0	0	0	0.062	0	0	0.718	0.724
SPP1	SYK	9606.ENSP00000378517	9606.ENSP00000364907	0	0	0	0	0.061	0.079	0.9	0.186	0.92
SRC	VTN	9606.ENSP00000362680	9606.ENSP00000226218	0	0	0	0	0	0	0.9	0.593	0.957
SRC	TLR2	9606.ENSP00000362680	9606.ENSP00000260010	0	0	0	0	0	0.123	0	0.72	0.744
SRC	VCAM1	9606.ENSP00000362680	9606.ENSP00000294728	0	0	0	0	0.055	0	0.9	0.484	0.947
SRC	TLR3	9606.ENSP00000362680	9606.ENSP00000296795	0	0	0	0	0	0.432	0	0.671	0.805
SRC	TLR4	9606.ENSP00000362680	9606.ENSP00000363089	0	0	0	0	0.062	0.551	0	0.756	0.888
SRC	SYK	9606.ENSP00000362680	9606.ENSP00000364907	0	0	0	0.729	0.054	0.393	0.9	0.913	0.953
SYK	TLR2	9606.ENSP00000364907	9606.ENSP00000260010	0	0	0	0	0.23	0.123	0	0.647	0.74
SYK	TLR3	9606.ENSP00000364907	9606.ENSP00000296795	0	0	0	0	0	0.392	0	0.558	0.72
SYK	TLR4	9606.ENSP00000364907	9606.ENSP00000363089	0	0	0	0	0.124	0.46	0	0.821	0.908
TGFB2	TGFB3	9606.ENSP00000355896	9606.ENSP00000238682	0	0	0	0.951	0.096	0.32	0.9	0.927	0.936
TGFB2	THBS1	9606.ENSP00000355896	9606.ENSP00000260356	0	0	0	0	0.08	0	0.9	0.473	0.947
TGFB2	TGFBR2	9606.ENSP00000355896	9606.ENSP00000351905	0	0	0	0	0	0.507	0.9	0.742	0.986
TGFB2	TMSB4X	9606.ENSP00000355896	9606.ENSP00000370010	0	0	0	0	0	0	0.9	0.055	0.901
TGFB2	VEGFC	9606.ENSP00000355896	9606.ENSP00000480043	0	0	0	0	0.126	0	0.9	0.295	0.933
TGFB3	TMSB4X	9606.ENSP00000238682	9606.ENSP00000370010	0	0	0	0	0	0	0.9	0.098	0.906
TGFB3	VEGFC	9606.ENSP00000238682	9606.ENSP00000480043	0	0	0	0	0.061	0	0.9	0.195	0.917
TGFB3	THBS1	9606.ENSP00000238682	9606.ENSP00000260356	0	0	0	0	0.062	0	0.9	0.338	0.932
TGFB3	TGFBR2	9606.ENSP00000238682	9606.ENSP00000351905	0	0	0	0	0.062	0.961	0.9	0.685	0.998
THBS1	TMSB4X	9606.ENSP00000260356	9606.ENSP00000370010	0	0	0	0	0	0	0.9	0.108	0.906
THBS1	VCAM1	9606.ENSP00000260356	9606.ENSP00000294728	0	0	0	0	0.062	0	0.9	0.65	0.964
THBS1	VEGFC	9606.ENSP00000260356	9606.ENSP00000480043	0	0	0	0	0.152	0	0.9	0.677	0.97
TLR1	TLR2	9606.ENSP00000354932	9606.ENSP00000260010	0	0	0	0.771	0.507	0.882	0.9	0.962	0.995
TLR2	TLR4	9606.ENSP00000260010	9606.ENSP00000363089	0	0	0	0.624	0.263	0	0.8	0.964	0.902
TLR3	TSLP	9606.ENSP00000296795	9606.ENSP00000339804	0	0	0	0	0	0	0	0.75	0.75
TMSB4X	VEGFC	9606.ENSP00000370010	9606.ENSP00000480043	0	0	0	0	0	0	0.9	0.058	0.901
TNFAIP3	TNFRSF1	9606.ENSP00000481570	9606.ENSP00000162749	0	0	0	0	0.062	0.416	0.9	0.55	0.972
TNFRSF1	TNFSF10	9606.ENSP00000221132	9606.ENSP00000241261	0	0	0	0	0	0.993	0.9	0.883	0.999
TNFRSF1	TNFRSF1	9606.ENSP00000326737	9606.ENSP00000291232	0	0	0	0	0	0	0.9	0.421	0.939
TNFRSF1	TNFSF12	9606.ENSP00000326737	9606.ENSP00000293825	0	0	0	0	0	0.379	0.9	0.939	0.995
TNFRSF1	TNFSF12	9606.ENSP00000291232	9606.ENSP00000293825	0	0	0	0	0	0	0.9	0.441	0.941
TNFRSF1	TNFSF12	9606.ENSP00000162749	9606.ENSP00000293825	0	0	0	0	0	0	0	0.742	0.742

TNFRSF1[illegible]	VCAM1	9606.ENSP00000162749	9606.ENSP00000294728	0	0	0	0	0	0	0	0.742	0.742
VIP	VIPR1	9606.ENSP00000356213	9606.ENSP00000327246	0	0	0	0	0.07	0.713	0.9	0.905	0.997
